# *In vitro* evolution of enhanced RNA replicons for immunotherapy

**DOI:** 10.1038/s41598-019-43422-0

**Published:** 2019-05-06

**Authors:** Yingzhong Li, Brian Teague, Yuan Zhang, Zhijun Su, Ely Porter, Brian Dobosh, Tyler Wagner, Darrell J. Irvine, Ron Weiss

**Affiliations:** 1 Department of Biological Engineering, Massachusetts Institute of Technology, Cambridge, Massachusetts USA; 2 Koch Institute for Integrative Cancer Research, Massachusetts Institute of Technology, Cambridge, Massachusetts USA; 3 Department of Materials Science and Engineering, Massachusetts Institute of Technology, Cambridge, Massachusetts USA; 4 Ragon Institute of Massachusetts General Hospital, Massachusetts Institute of Technology and Harvard University, Cambridge, Massachusetts USA; 5 Howard Hughes Medical Institute, Chevy Chase, Maryland USA; 6 Department of Biomedical and Pharmaceutical Sciences, College of Pharmacy, University of Rhode Island, Kingston, RI USA

**Keywords:** Synthetic biology, Cancer immunotherapy, Experimental evolution

## Abstract

Self-replicating (replicon) RNA is a promising new platform for gene therapy, but applications are still limited by short persistence of expression in most cell types and low levels of transgene expression *in vivo*. To address these shortcomings, we developed an *in vitro* evolution strategy and identified six mutations in nonstructural proteins (nsPs) of Venezuelan equine encephalitis (VEE) replicon that promoted subgenome expression in cells. Two mutations in nsP2 and nsP3 enhanced transgene expression, while three mutations in nsP3 regulated this expression. Replicons containing the most effective mutation combinations showed enhanced duration and cargo gene expression *in vivo*. In comparison to wildtype replicon, mutants expressing IL-2 injected into murine B16F10 melanoma showed 5.5-fold increase in intratumoral IL-2 and 2.1-fold increase in infiltrating CD8 T cells, resulting in significantly slowed tumor growth. Thus, these mutant replicons may be useful for improving RNA therapeutics for vaccination, cancer immunotherapy, and gene therapy.

## Introduction

Nucleic acid therapeutics have the potential to treat or cure many diseases that are difficult to address with more traditional therapies^1^. Importantly, delivery of exogenous nucleic acids to host cells allows therapeutic proteins to be produced that retain native conformations and post-translational modifications (e.g. glycosylation)^2,3^, which are often difficult to achieve with recombinant proteins. One type of nucleic acid, synthetic mRNA, is particularly attractive for its improved safety profile relative to viral or DNA-based modalities; the likelihood of genomic integration is low, and hence oncogenesis is less of a concern^4^. These advantages make synthetic mRNA a promising platform for vaccines, cancer therapeutics, and therapies that compensate for (or correct) genetic defects, among others^5^.

Unfortunately, synthetic RNAs degrade rapidly in recipient cells, limiting their therapeutic utility. A synthetic RNA’s persistence can be extended by biological modifications (such as enzymatic capping and polyadenylation) and incorporation of chemically modified nucleotides^6–8^, but even modified RNAs often remain active in cells for only several days^9^, making them unsuitable for long-term gene therapy. One alternative approach is to employ replicons derived from alphaviruses, positive-strand RNAs that encode RNA-dependent RNA polymerases which simultaneously transcribe therapeutic payloads and self-amplify the replicon on entry into the cytoplasm^10^. Specifically, therapeutic replicons are constructed by retaining the UTRs, non-structural proteins and subgenomic promoter (SGP) of the parent alphavirus, but the structural proteins in the subgenomic region are either fused with or replaced by genes of experimental or therapeutic interest. Replicons delivered as engineered viral particles or as synthetic RNA encapsulated in lipid nanoparticles have shown promise as non-viral vaccine vectors^11,12^, for expression of therapeutic agents in cancer immunotherapy^13,14^, and for correction of genetic defects such as hemophilia^15^. Motivated by these promising examples, we began exploring strategies to regulate gene expression using replicons derived from the Venezuelan Equine Encephalitis (VEE) virus^16,17^. Because replicons encode proteins necessary to copy the RNA itself, they persist in cells longer than modified synthetic RNA and can express genes in the subgenome at a high level. However, even though gene expression from the replicon is stronger and lasts longer than comparable synthetic mRNAs, expression still fades gradually^17–19^ due to the host cell’s innate immune response^20–23^.

Distinct from host mRNA, replicon RNAs encode a set of four nonstructural proteins (nsPs 1–4) that are responsible for both genome replication and transcription of “cargo” products under the subgenomic promoter. The nsPs control many facets of replicon biology and the host cell response, and thus have been a focus of both fundamental studies and engineering efforts. For example, Frolov *et al*. used replicons expressing puromycin acetyltransferase as a selectable marker and identified several mutations in nsP2 which control the cytopathicity of Sindbis virus replicons^24^. Rose *et al*. used infectious Semliki Forest viral particles to evolve replicons through long-term serial infections of *in vitro* cell cultures, and identified multiple mutations promoting high-titer production of virus-like vesicles from SFV replicons^25^.

Based on these past successes, we started with non-cytopathic VEE^26^ and designed a new *in vitro* evolution strategy that uses interferon (IFN)-competent cells and multiple long duration rounds of replicon enrichment for higher subgenome expression. In contrast to the use of IFN-deficient BHK-21 cells in previous studies, we decided to transfect replicons into IFN-competent Jurkat cells to select for replicon mutations compatible with innate immune responses of host cells, as this is more representative of our ultimate delivery targets. We chose to subject the replicons to multiple long duration rounds of growth in cell culture with selection of top performing replicons to continue growing in each successive round via fluorescence activated cell sorting based on highest subgenome expression of a fluorescent protein. Since nsPs provide a potentially powerful strategy for modifying and enhancing replicon expression *in vitro* and *in vivo*, we focused on replicon constructs with the structural proteins deleted, in order to discover possible mutations that impact the persistence of replicon RNA and the strength of gene expression from the subgenome rather than viral particle production/packaging. The objective was to develop an improved non-viral platform for delivery of therapeutic cargo as well as synthetic gene circuits for better vaccinations and cancer immunotherapies. Using our *in vitro* evolution approach, we identified six mutations in nonstructural proteins nsP2 and nsP3. Five of the six had significant impact on replicon persistence and gene expression levels. Two of the mutations increased transgene expression, while three mutations suppressed subgenomic transcription. Interestingly, some of these mutations also altered the strength of the host cell’s interferon response to the replicons. After discovery of our mutants *in vitro*, we used synthetic nanoparticle formulations for delivery of RNA^12^ *in vivo* to avoid any reliance on viral particle production. When applied in a model of cancer immunotherapy, optimized mutant replicons identified here harboring the immunoregulatory cytokine interleukin-2 significantly improved treatment of murine B16F10 melanoma. The mutant replicon sequences identified here thus improve the utility of replicon RNA as a platform for gene therapy.

## Results

### *In vitro* evolution of VEE replicons identifies mutations associated with prolonged and higher payload gene expression

**Figure 1:**
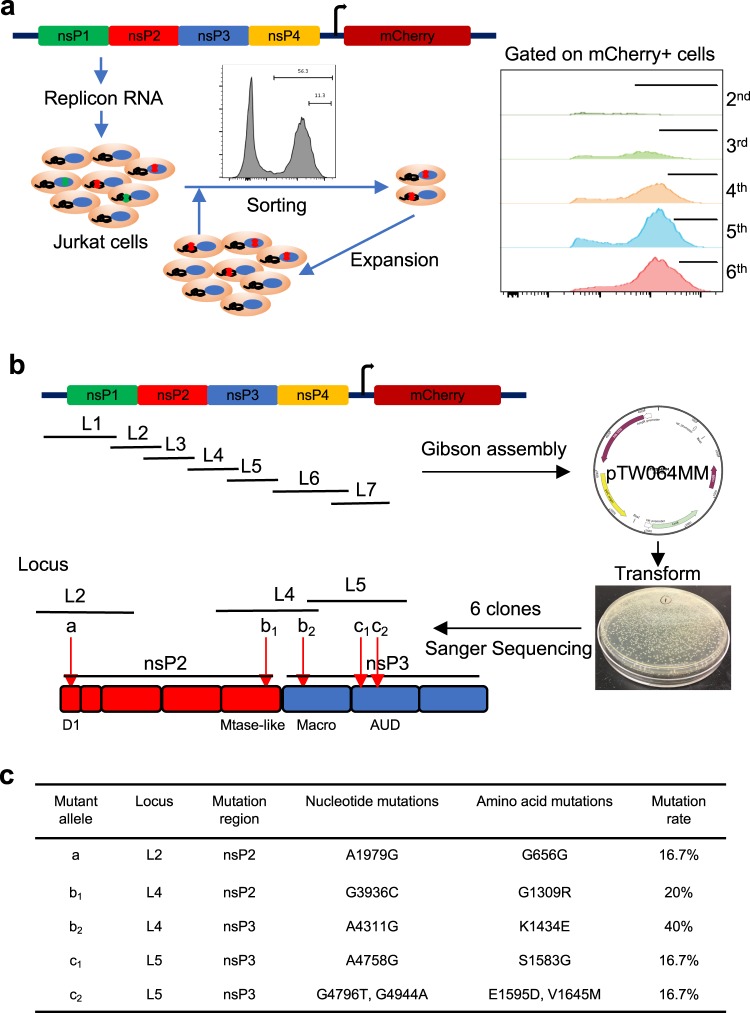
Identification of mutant replicons promoting subgenome expression by *in vitro* evolution. (**a**) Method for *in vitro* replicon evolution: Jurkat cells were transfected with replicon RNA encoding mCherry under the SGP and grown in cell culture. The top 20% of the mCherry^+^ population were sorted for approximately every 10 days during serial passaging as indicated by the flow cytometry histograms, leading to an enrichment in cells expressing high levels of the reporter gene. Cells from the 5^th^ sort were isolated for replicon sequencing. (**b**) Identification of mutations: Total RNA from mCherry positive cells was extracted and reverse transcribed to cDNA. Then, nsP1–4 and the subgenomic promoter were amplified by seven pairs of specific primers and amplicons from Loci 1–7 were engineered into plasmid DNA and transformed into *E*. *coli* for amplication. Six clones from each locus were randomly picked for Sanger Sequencing. Schematic at bottom left shows the approximate locations in nsP2 and nsP3 where point mutations were identified in 5 mutant alleles with c_2_ harboring two linked mutations. (**c**) Table describing the 5 mutant alleles identified in the screen. Mutation rates describe the percentages of mutations in the sequenced loci.

In order to identify mutations in the replicon that impact the persistence and strength of expression of payload genes under the subgenomic promoter, we designed an *in vitro* evolution strategy using VEE replicons that lack genes encoding the structural proteins. This allowed us to focus our mutational screen on the nonstructural proteins involved in RNA replication and host machinery interactions. We hypothesized that extended culture of replicon-transfected cells combined with repeated enrichment of highly-expressing cells would select for replicons bearing favorable mutations. While many replicon studies are carried out using BHK-21 cells that are deficient in interferon signaling, we employed human Jurkat cells that have a functional interferon pathway for transfection and passage of replicons, in order to potentially select for mutations limiting the host cell interferon response. VEE replicons expressing the fluorescent protein mCherry from the subgenomic region were synthesized by *in vitro* transcription (IVT) and transfected into Jurkat cells. Based on prior studies estimating RNA virus mutation rates^27,28^, we cultured the transfected cells for 60 days, selecting the top 20% of mCherry-expressing cells approximately every 10 days by fluorescence-activated cell sorting (FACS). The percentage of mCherry^hi^ cells and mean fluorescence intensity of mCherry expression increased with each sort (Fig. 1a, Supplemental Fig. 1), suggesting that mutations in either the replicon RNA or changes in the host Jurkat cells were favoring replicon persistence and stronger subgenome expression.

The percentage of mCherry-positive cells began to plateau after 6 sorts (Supplemental Fig. 1b), and thus we selected cells from the 5^th^ sort for sequencing to assess potential mutations arising in the replicon. Total RNA was isolated from the sorted Jurkat cells, reverse-transcribed to cDNA, and amplicons from seven overlapping regions covering the nonstructural proteins (loci L1–L7) were each separately cloned into plasmid vectors (Fig. 1b). Plasmids expressing the amplicon constructs were transformed into *E*. *coli*, and 6 colonies from each amplicon were isolated for Sanger sequencing. We treat each of the 7 amplified regions as a genetic locus, and each clone bearing one or more mutations as an allele at that locus. Mutations were identified in 3 of the loci, L2, L4, and L5. We named the wildtype alleles at each of these loci as *A*, *B*, and *C*, respectively, while mutant alleles were assigned lowercase letters: One recovered amplicon sequence containing a synonymous point mutation in L2 was named allele *a*; two different mutant alleles identified in L4 were named *b*_1_ and *b*_2_; and two mutant alleles in L5 were named *c*_1_ (bearing one point mutation) and *c*_2_ (containing two point mutations) (Fig. 1c). Using this nomenclature, a replicon construct is specified by which alleles it bears at these three loci. The 3 WT (*A*, *B*, *C*) and 5 mutant alleles (*a*, *b*_1_, *b*_2_, *c*_1_, *c*_2_) from these 3 loci combine to give 18 total potential variants made up of unique WT/mutant allele combinations (Supplemental Table 1). Replicon *ABC*, for example, is the wildtype replicon, while replicon *ab*_2_*C* has the mutant *a* allele at L2 and the mutant *b*_2_ allele at L4, but wildtype *C* allele at L5. As shown in Fig. 1b, the identified mutations were located in the D1/D2 and methyltransferase-like domains of nsP2 and the Macro and alphavirus unique domain (AUD) of nsP3.

### *In vitro*-selected mutations impact the intensity and longevity of expression of reporter genes under the subgenomic promoter

**Figure 2:**
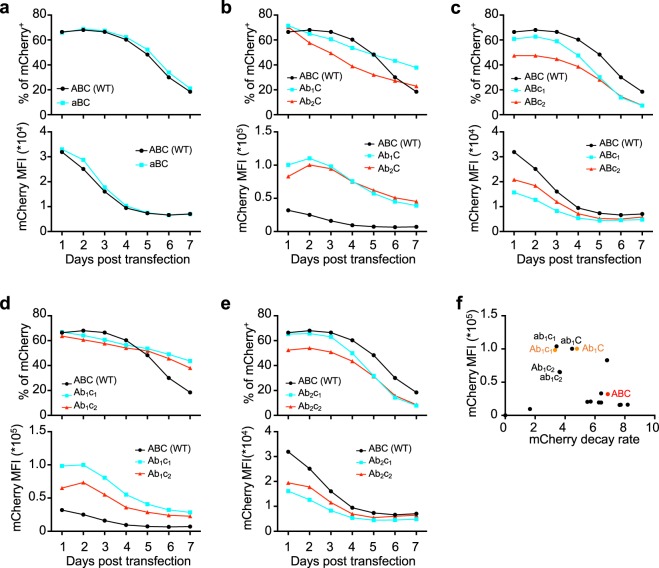
Mutations in alleles b_1_, b_2_, c_1_ and c_2_ impact the intensity and longevity of transgene expression. (**a**–**e**) Jurkat cells were transfected with WT or mutant replicons encoding mCherry as a reporter and then cultured for 7 days; mean fluorescence intensities (MFI) and the percentages of mCherry^+^ cells were tracked over time by flow cytometry. Shown are comparisons of WT with replicons containing mutant alleles (**a**) *a*, (**b**) *b*_1_ and *b*_2_, (**c**) *c*_1_ and *c*_2_, or combinations of alleles *c*_1_ and *c*_2_ with (**d**) *b*_1_ (**e**) or *b*_2_. mCherrry MFI are shown with a y-axis range of either 0–4.0 × 10^4^ or 0–1.5 × 10^5^ to highlight difference relative to WT replicon. (**f**) Scatter plot of maximal mCherry MFI vs. the rate of decay of mCherry^+^ cells for all replicon tested. mCherry decay rate is computed as percentage of mCherry^+^ cells on day 1 minus this percentage on day 7, and divided by 7. The promising mutants and WT replicons for later study are highlighted as orange and red, respectively.

To better understand the impact of these mutations on gene expression from the replicon, we built 17 synthetic replicons using all combinations of the 5 mutant alleles we identified in our screen (Supplemental Table 1), and compared the strength and duration of transgene expression to the wildtype replicon (*ABC*). Each replicon was transfected into Jurkat cells and mCherry expression was tracked for 7 days using flow cytometry. Allele *a* had minimal impact on the proportion of cells expressing replicon or the level of subgenome expression (Fig. 2a). By contrast, alleles *b*_1_ and *b*_2_ increased the intensity of mCherry expression approximately 3-fold compared to the wildtype replicon (Fig. 2b). Alleles *c*_1_ and *c*_2_ alone elicited minor changes in expression relative to the WT replicon (Fig. 2c), but when *c*_1_ was combined with allele *b*_1_, the durability of mCherry expression over time was further enhanced (Fig. 2d,e). Summarizing the kinetic data in a plot of Day 1 mCherry mean fluorescence intensity vs. the rate of mCherry expression loss over time, the most improved replicons relative to the WT *ABC* sequence are readily identified, with mutants *Ab*_1_*C* and *Ab*_1_*c*_1_ showing particularly high levels of transgene expression combined with the lowest expression decay rates (Fig. 2f).

### Alleles b_1_ and b_2_ increase replicon RNA persistence, while c_1_ and c_2_ decrease subgenomic transcription

**Figure 3:**
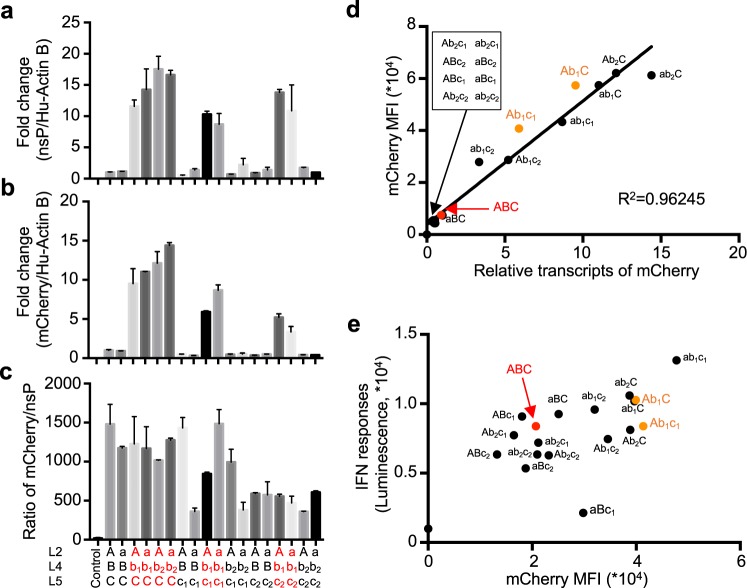
Alleles b_1_ and b_2_ enhance both subgenome and whole replicon genome RNA levels relative to WT replicons. (**a**,**b**) Jurkat cells were transfected with WT or mutant replicons and analyzed on day 5 for levels of nsP RNA (**a**; serving as a surrogate for whole genome copies) or mCherry RNA (**b**) by PCR. Shown are actin-normalized RNA levels relative to the WT *ABC* construct. (**c**) Ratios of mCherry to nsP transcripts in cells transfected with replicon RNA. (**d**) Scatter plot of mCherry MFI vs. relative mCherry transcripts in Jurkat cells on 5 day for all replicon tested. Coefficient of determination (R-squared) is indicated. (**e**) RAW-Lucia ISG reporter cells were transfected with replicons and the interferon response and mCherry fluorescence levels were observed after 24 hr via bioluminescence and flow cytometry, respectively. In (**d**,**e**), the promising mutants and WT replicons for later study are highlighted in orange and red, respectively.

During alphavirus transcription by the nsPs, both copies of the entire replicon genome and shorter transcripts of only the subgenome are produced. Thus, we next evaluated the impact of the replicon mutations on levels of intracellular subgenome vs. whole replicon genome transcripts. Relative levels of nonstructural proteins (Fig. 3a) and mCherry (Fig. 3b) transcripts in Jurkat cells transfected with all 18 replicons were measured by qPCR. Cells transfected with replicons *Ab*_1_*C*, *ab*_1_*C*, *Ab*_2_*C*, *ab*_2_*C*, *Ab*_1_*c*_1_, *ab*_1_*c*_1_, *Ab*_1_*c*_2_, and *ab*_1_*c*_2_ had 10–18 times more replicon RNA compared to cells transfected with the wildtype (*ABC*) replicon (Fig. 3a). Subgenomic RNA levels of mCherry RNA generally correlated with the nsP RNA levels. (Fig. 3b), suggesting the subgenomic expression is mainly determined by the presence of replicon RNA. Alleles *b*_1_ and *b*_2_ at locus L4 demonstrated higher overall mCherry expression (Fig. 2a), indicating that alleles *b*_1_ and *b*_2_ increase replicon RNA levels as well as mCherry subgenomic RNA level compared to wildtype allele *B*, and that alleles *c*_1_ and *c*_2_ in locus L5 suppress this effect in replicons with allele *b*_2_ but not allele *b*_1_.

All six replicons with allele *C* (including the wildtype replicon) exhibited mCherry transcript to whole replicon genome RNA ratios of 1000–1500. In contrast, the six replicons with allele *c*_2_ exhibited lower relative subgenome expression, with mCherry/nsP ratios of around 500. The six replicons carrying allele *c*_1_ had widely varying mCherry/nsP ratios, ranging from 400 to 1500, depending on the presence of other mutations (Fig. 3c). Thus, alleles *b*_1_ and *b*_2_ increased subgenome expression (Fig. 3b) by increasing overall replicon RNA in cells (Fig. 3a), rather than by enhancing subgenomic transcription (Fig. 3c). In contrast, allele *c*_2_ decreased subgenome expression (Fig. 3b) by decreasing replicon RNA as well as subgenomic transcription (Fig. 3b,c). The effects of alleles *a* and *c*_1_ on subgenomic expression are highly context-sensitive, depending on which alleles are at the other two loci.

We were interested in the mechanism that led to some mutant replicons being present in cells at levels 10 to 18-fold higher than wildtype. We reasoned that these mutations may aid the replicon in escaping the cell’s innate interferon response, which is activated by viral RNA and limits its replication^20–23^. To test this hypothesis, we transfected all 18 replicons into RAW-Lucia ISG cells, which secrete luciferase in response to interferon^29,30^. We observed that mCherry transcripts correlated with mCherry MFI (mean fluorescence intensity) in Jurkat cells (Fig. 3d) and that mCherry MFI was also correlated between Jurkat and RAW-Lucia ISG cells (Supplemental Fig. 2), suggesting that mCherry MFI indicates mCherry transcript abundance, and that replicon replication and expression dynamics are similar in these two reporter cells. We further analyzed the effects of mutant alleles on mCherry MFI (Supplemental Fig. 3), observing that in general *b*_1_ and *b*_2_ enhance expression, while *c*_1_ and *c*_2_ regulate this expression (also noted in Fig. 2). We then compared IFN responses (luminescence) versus mCherry MFI in RAW-Lucia ISG cells transfected with each of the 18 replicons (Fig. 3e). Interestingly, 5 of 6 mutant replicons with allele *c*_2_ showed decreased IFN responses, suggesting that allele *c*_2_ may reduce IFN response by restriction of subgenomic transcription (Fig. 3c). Similarly, 4 of 6 mutant replicons with allele *c*_1_ decreased IFN responses too, suggesting that allele *c*_1_ may also be involved in escaping the IFN response. Unexpectedly, 5 of 6 mutant replicons with allele *b*_2_ also showed less IFN response, which may be due to low subgenomic expression of this allele (Fig. 2e). In contrast, 4 of 6 mutant replicons with allele *b*_1_ showed higher IFN responses, which may result from stimulation of IFN responses by more replicon RNA (Fig. 3a) as well as more subgenomic transcripts (Fig. 3b). Allele *a*, on the other hand, has more complicated effects on IFN responses, depending on which other alleles are present. Taken together, in general allele *b*_1_ and *b*_2_ increase, but *c*_1_ and *c*_2_ decrease IFN response (Supplemental Fig. 4). In summary, using Jurkat cells, we successfully identified several mutant alleles with altered interactions between the replicon and host cell’s innate immune response, though mechanisms underlying these changes may be complicated.

### Mutant replicons increase gene expression magnitude and persistence *in vivo*

**Figure 4:**
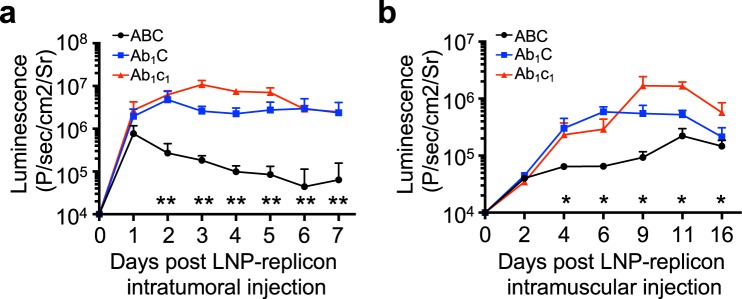
Replicon mutations impact the strength and persistence of transgene expression *in vivo*. (**a**) C57BL/6J mice (*n* = 4/group) bearing B16F10 tumors ~50 mm^2^ in size were injected intratumorally with 10 µg LNP-formulated WT or mutant replicons encoding luciferase, and bioluminescence was tracked over time. (**b**) C57BL/6 J mice (*n* = 4/group) were injected intramuscularly with 2.5 µg LNP-formulated WT or mutant replicons encoding luciferase, and bioluminescence was tracked over time. Statistics were analyzed by two-way ANOVA. * and **mean p-value < 0.05 and <0.01, respectively.

To evaluate the impact of evolved mutations on replicon expression *in vivo*, we selected two of the mutant sequences with enhanced behavior *in vitro* for further analysis and comparison to the wildtype replicon: mutants *Ab*_1_*C* and *Ab*_1_*c*_1_ (highlighted in orange in Figs 2f and 3d) because these two mutants showed higher subgenomic expression, lower mCherry loss rate, and IFN responses comparable to wildtype *ABC*. Mutant and wildtype replicons expressing luciferase from the subgenome were synthesized and encapsulated in lipid nanoparticles (LNPs) for transfection *in vivo*^12^. Localized treatment of cancer using immunotherapeutics injected intratumorally is a therapy modality of growing interest clinically, based on preclinical and clinical evidence that locally-generated T cell responses can disseminate to cause regression of distal untreated tumors^31–34^. Modeling this setting, we first injected LNPs carrying encapsulated replicon RNA intratumorally (i.t.) in C57BL/6J mice bearing B16F10 melanoma tumors. While the WT VEE replicon showed steadily decaying expression from day 1 onward, both the *Ab*_1_*C* and *Ab*_1_*c*_1_ constructs maintained expression at a level ~10-fold greater on average than WT replicon (Fig. 4a). In a separate experiment, we also injected these three replicons intramuscularly to model their use in a vaccination setting. Intramuscular expression of all 3 replicons increased over time, but climbed substantially more rapidly for the mutant replicons, which exhibited on average ~6-fold greater bioluminescence signal by day 6 (Fig. 4b). Interestingly, the *Ab*_1_*C* and *Ab*_1_*c*_1_ constructs showed relatively constant high expression over 7 days following intratumoral administration, but exhibited a continuous increase in expression muscle over the same time course. We expect these distinct kinetics of expressions likely reflect differences in replicon biology within the cell populations transfected in these two different tissues (tumor cells/immune cells vs. muscle cells).

### Enhanced anti-tumor immunity stimulated by mutant replicons

**Figure 5:**
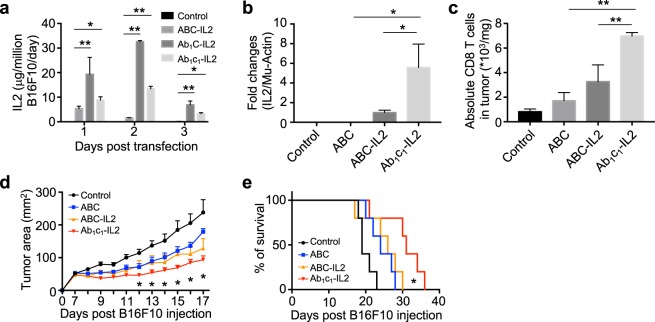
Mutant replicons encoding interleukin-2 exhibit enhanced gene expression and anti-tumor activity over WT VEE replicons. (**a**) B16F10 melanoma cells were transfected with WT or mutant replicons encoding IL-2, and secreted IL-2 present in the supernatants was measured over 3 days by ELISA. Statistics were analyzed by two-way ANOVA. * and **mean p-value < 0.05 and <0.01, respectively. (**b**,**c**) Groups of C57BL/6J mice (*n* = 4/group) bearing B16F10 tumors ~50 mm^2^ in size were injected intratumorally with 10 µg LNP-formulated IL-2-encoding replicons (*ABC*-IL2, or *Ab*_1_*c*_1_-IL2) or WT (*ABC*) control replicon with no subgenome cargo. Transcripts of IL-2 within the tumors were measured by PCR 3 days post injection (**b**), and total numbers of CD8 T cells in tumors were tallied 3 days post LNP-replicon injection (**c**). Statistics were analyzed by one-way ANOVA. * and **mean p-value < 0.05 and <0.01, respectively. (**d**,**e**) C57BL/6J mice (*n* = 5/group) were injected s.c. in the flank with 10^6^ B16F10 cells, and then received intratumoral injections of 10 µg LNP-formulated WT control or IL-2-encoding replicons on day 7 (**d**) or on days 7, 9, and 11 (**e**). Shown are average tumor growth (**d**) and overall survival (**e**). Survival statistics were analyzed by Curve Comparison. *mean p-value < 0.05.

The enhanced expression of payload genes from the SGP of the mutant replicons suggests they could be an attractive platform for gene-based immunotherapy. As a preliminary exploration of this application, we generated WT and mutant *Ab*_1_*c*_1_ replicons expressing interleukin-2 (IL-2) under the SGP. IL-2 is a potent cytokine that promotes the proliferation and effector functions of CD8^+^ T cells and natural killer cells^35^. We first evaluated secretion of IL-2 from transfected tumor cells *in vitro*. As shown in Fig. 5a, even *in vitro*, replicon expression in B16F10 tumor cells transfected with WT replicon *ABC* encoding IL-2 decayed to near baseline by 48 hr, while secreted IL-2 protein was detected over at least 3 days in culture from the mutant replicons and at much higher levels than the WT construct. To assess replicon-based IL-2 expression *in vivo*, we injected LNPs carrying replicon RNA into established B16F10 tumors and measured IL-2 transcript levels by quantitative RT-PCR 3 days post injection. As shown in Fig. 5b, tumor samples injected with Ab_1_c_1_ replicons expressed 5.5-fold more transcripts than the WT replicon. Tumors with the *Ab*_1_*c*_1_ mutant replicon showed 2.1-fold more infiltrating CD8 T cells than the wildtype ABC replicon (Fig. 5c). When a single dose of replicon was administered 7 days post tumor inoculation, the *Ab*_1_*c*_1_ mutant IL-2-expressing replicon significantly slowed tumor progression compared to either the ABC IL-2 replicon, the ABC replicon without IL-2, or the vehicle control (Fig. 5d). Further, 3 doses of *Ab*_1_*c*_1_ replicon administered at days 7, 9, and 11-day post tumor cell inoculation led to substantially prolonged overall survival compared to the other constructs (Fig. 5e). In summary, the mutations identified here improve the therapeutic potential of self-replicating RNA, with implications for cancer immunotherapy and beyond, e.g. for vaccination and gene therapy.

## Discussion

Alphavirus replicons are of substantial interest as platforms for gene therapy and vaccination, with the ability to encode therapeutic genes or antigens under the subgenomic promoter in place of the structural proteins required for replicon replication. To increase the effectiveness of replicon RNA, we developed an *in vitro* evolution (IVE) strategy to identify mutations in VEE alphavirus replicons that enhanced the strength and persistence of gene expression from the replicon’s subgenome in human and murine cells. Interferon-deficient BHK-21 cells have often been used in alphavirus studies, as type I interferons strongly restrict replicon expression^36^. We opted to instead carry out IVE in Jurkat cells that maintain an intact interferon response, in order to allow for the possibility of selecting mutants with altered interferon induction. Interestingly, although mutants were found with a lower interferon response (e.g. *aBc*_1_ and *aBc*_2_), these replicons did not provide the best performance in terms of subgenome transgene expression (Figs 2c,f and 3e).

The IVE screen identified 5 non-synonymous mutations associated with enhanced expression in Jurkat cells *in vitro*. Allele *b*_1_ carried a favorable mutation in nsP2, a non-structural protein that has previously been shown to regulate the cytopathic effect of alphavirus infection^37,38^. The mutation in allele *b*_2_ was located in the macrodomain of nsP3 but had similar effects on expression strength as allele *b*_1_, consistent with previous reports that the protease and MTase domains in nsP2 and the macrodomain and AUD in nsP3 function as a compact complex^39^. Alleles c_1_ and c_2_ carried mutations in nsP3 which is involved in subgenomic transcription^40^. These alleles suppressed expression in combination with allele *b*_2_ but enhanced the durability of expression when combined with allele *b*_1_, suggesting that replicon transcription and subgenome transcription are independent but related activities. Though our evolution methods experiment was aimed at improving replicon performance, it has raised interesting questions about the underlying mechanisms of alphavirus replication that merit further study.

Our *in vivo* results also highlight the mutant replicons’ therapeutic potential. Injected intramuscularly, a common site for vaccine administration, the mutant replicons showed more rapid and higher-level transgene expression over at least one week *in vivo*. Administered intratumorally, the best-performing mutant replicon showed strong transgene expression *in vivo* a week after administration, contrasting with rapid decay of expression from the wildtype replicon. We expect these changes in expression behavior to be of value both in many applications, but particularly for vaccine applications and cancer immunotherapy. For example, studies of the role of vaccine kinetics in humoral responses to vaccination have indicated that prolonged exposure to antigen promotes germinal center responses and increases the production of durable high-titer antibody responses in serum^41,42^. The increased magnitude and persistence of gene expression from the mutant replicons also led to increased anti-tumor efficacy in a mouse melanoma model. This like reflects the need for sustained signaling in the tumor microenvironment to overturn tumor immunosuppression and prime anti-tumor immunity. Sustained stimulation in this manner is likely important for a range of immunostimulatory cytokines and immunomodulators, making the mutant replicon platform of broad interest for cancer immunotherapy.

## Methods

### Cell lines and animals

Cell lines Jurkat (ATCC® TIB-152™), Raw-Lucia ISG (http://www.invivogen.com/raw-lucia-isg), and B16F10 (ATCC® CRL-6475™), were cultured following vendor instructions (37 °C, 5% CO_2_). Female C57BL/6J (JAX Stock No. 000664) mice 6–8 weeks of age were maintained in the animal facility at the Massachusetts Institute of Technology (MIT). All animal studies and procedures were carried out following federal, state, and local guidelines under an IACUC-approved animal protocol (the MIT Committee on Animal Care (CAC) Protocol Number: 0717-076-20).

### Constructs, *in vitro* transcription, capping/methylating for replicon RNA, and neon transfection

Wildtype VEE replicon RNA was prepared as described in Wrobleska *et al*.^17,26^. mCherry was amplified by two round PCR with the primers YL-mCherry-ClaI-F, YL-mCherry-R1, and YL-mCherry-ClaI-F, YL-mCherry-SphI-R2 (Supplementary Table 2). Fragments from the second round PCR were cloned into the VEE replicon construct^43,44^ to obtain plasmids encoding the wildtype VEE-mCherry construct.

Replicon RNAs were *in vitro* transcribed (IVT) from the templates of linearized VEE-constructs above using the MEGAscript™ T7 Transcription Kit (ThermoFisher) following the manufacturer’s instructions. Resulting replicon RNAs were capped and methylated using the ScriptCap™ m7G Capping System and ScriptCap™ 2′-O-Methyltransferase Kit (Cellscript) according to the manufacturer’s instructions. RNA purity was assessed by gel electrophoresis.

*In vitro* transfections were carried out using 1 µg RNA for per 200,000 cells using the NEON electroporation kit (ThermoFisher) following the manufacturer’s instructions.

### *In vitro* evolution screen

Jurkat cells were transfected with VEE-mCherry replicon RNA using a NEON transfection kit and the cells were cultured in 37 °C with 5% CO_2_ for 10 days. The 20% cells expressing highest levels of mCherry were then sorted using a BD Aria III sorter, and sorted cells were cultured for 10 days prior to the next sorting. We repeated sorting through 6 rounds and selected the 5^th^ round sorted cells for total RNA extraction and cDNA synthesis. Using the cDNA as template, we divided the nsP1–4 and subgenomic promoter regions into 7 overlapping loci, and each loci was amplified by 7 pairs of primers, YL-Locus-5′UTR-F1 and YL- Locus-R1, YL-Locus-F2 and YL-Locus-R2, YL-Locus-F3 and YL-Locus-R3, YL-Locus-F4 and YL-Locus-R4, YL-Locus-F5 and YL-Locus-R5, YL-Locus-F6 and YL-Locus-R6, YL-Locus-F7 and YL Locus-R7, respectively (Supplementary Table 2). The 7 amplicons were cloned into the BsaI of pTW064MM and transformed into *E*. *coli* DH5α. Six clones from each locus were picked for Sanger sequencing.

### Assembly of mutant replicons

Mutations identified in the *in vitro* evolution screen were cloned into replicons encoding mCherry to obtain mutant replicons for characterization *in vitro* and *in vivo*. For cloning mutant allele *a* into the replicon construct, plasmids L2–4 were digested by SalI and EcoRI for insertion into the wildtype replicon construct. The constructs with alleles *b*_1_ and *b*_2_ were amplified by YL-nsP2-XmaI-F and YL-nsP3-PstI-R from plasmids L4–5 (b_1_) and L4–3 (b_2_) to clone into wildtype construct (*ABC*), respectively. For cloning the mutations *c*_1_ and *c*_2_, two fragment were amplified by primers YL-nsP3-PstI-F and YL-nsP4-OL-R from the plasmids L5–2 (c_1_) and L5–4 (c_2_), and by primers YL-nsP4-OL-F and YL-nsP4-AvrII-R from wildtype replicon construct (ABC). Then the two fragments with the wildtype replicon construct (ABC) digested by PstI-F and AvrII were assembled by NEBuilder® HiFi DNA Assembly kit. Other combinations were cloned in same method as above. The cited PCR primers are defined in Supplementary Table 2.

To synthesize replicon constructs ABC-IL2-P2A-mCherry, Ab_1_C-IL2-P2A-mCherry, and Ab_1_c_1_-IL2-P2A-mCherry, the fragments with BC (wildtype), b_1_C, and b_1_c_1_ were cut from the plasmids of VEE-ABC-mCherry, VEE-Ab_1_C-mCherry, and VEE-Ab_1_c_1_-mCherry, respectively, by restriction enzymes EcoRI and PspXI. Then pYL026 were replaced by these fragments. All of the restriction enzymes were purchased from NEB.

To synthesize replicon constructs ABC-Luc, Ab_1_C-Luc, and Ab_1_c_1_-Luc, the plasmids of ABC, Ab_1_C, and Ab_1_c_1_ expressing mCherry were replaced with Luc fragment pBD059 (unpublished) between the restriction enzymes ApaI and SphI.

### Antibodies, flow cytometry, sorting, and analysis

For analysis of CD8 T cells in B16F10 melanoma, single cell suspensions were prepared and stained^45^ with fluorophore-conjugated antibopdies against CD45 (Biolegend, Cat# 103116, Clone 30-F11), CD8 (Biolegend, Cat# 100706, Clone 53–6.7), and live dye Aqua (ThermoFisher Scientific, Cat# L34957). The stained cells were mixed with counting beads (ThermoFisher Scientific, Cat# C36950) and analyzed on a BD-LSRII Fortessa analyzer. All flow cytometry data were analyzed by FlowJo and the plots were prepared using GraphPad Prism.

### RNA extraction and quantitative PCR analysis

To quantify levels of RNA transcripts, total RNA was extracted from cells or tumors transfected with replicon RNA with the mutations as indicated and reverse transcribed by a TaqManÂ® Reverse Transcription Reagents Kit (ABI Catalog No. N8080234), followed by amplification with Sybr Green Master Mix (Roche) and specific primers YL-nsP3-qPCR-F and YL-nsP3-qPCR-R, YL-mCherry-F and YL-mCherry-R, YL-huActB-qPCR-F and YL-huActB-qPCR-R (Origene, Cat# HP204660), YL-mIL2-F and YL-mIL2-R (Origene, Cat# MP206769), and detected by a Roche LightCycler 480. The Ct values were normalized with housekeeping gene human Actin B for comparison.

### Lipid nanoparticle (LNP) formulation of replicons for *in vivo* delivery

For encapsulating 10 μg replicon RNA, a lipid mixture composed of 16.9375 μl DOTAP (Avanti, Cat# 890890, 10 mg/ml), 15.965 μl DSPC (Avanti, Cat# 850365, 3 mg/ml), 18.7675 μl cholesterol (Sigma-Aldrich, Cat# C8667, 6 mg/ml), 13.6 μl DSPE-PEG2000 (Avanti Cat# 880128, 2.5 mg/ml) in a molar ratio of 40:10:48:2 was prepared in ethanol and evaporated under N_2_ till one third of the total initial volume remained. Then 10 μg replicon RNA (1 mg/ml) in 11.8 μl 0.1 M citrate buffer (PH 6.0) was added with pipetting, followed by a second addition of an additional 22 μl 0.1 M citrate buffer (PH 6.0) with pipetting. The mixture was shaken for an hour and then dialyzed against PBS for another hour at 25 °C in a 3,500 MWCO dialysis cassette. The resulting replicon-loaded lipid nanoparticles were aliquoted in appropriate dosages for intratumoral injection (10 μg/mouse) or for intramuscular injection (2.5 μg/mouse).

### *In vivo* replicon studies

For intratumoral injections, C57BL/6J mice were injected s.c. in the flank with 10^6^ B16F10 cells. Seven days post injection, melanoma tumors were intratumoraly injected with 10 μg replicon RNA in LNPs. Then tumor areas were measured at the days indicated with calipers or necropsied for flow cytometry or for total RNA extraction. To mimic vaccination, groups of C57BL/6J mice were intramuscularly injected with 2.5 μg LNP-formulated replicon RNA encoding luciferase. Then the mice were imaged using an *In Vivo* Imaging System (XENOGEN IVIS 200) at 10 minutes after subcutaneous injection of 200 uL luciferin (8 mg/mL in PBS, GoldBio Cat# LUCK-1G) near the melanoma tumor or the muscle injected with LNP-replicon.

## Supplementary information

In vitro evolution of enhanced RNA replicons for immunotherapy
