# Supplementary material for: In vitro evolution of enhanced RNA replicons for immunotherapy
Source: Sci Rep. 2019 May 6;9:6932. doi: 10.1038/s41598-019-43422-0 (PMC6502795; doi:10.1038/s41598-019-43422-0)
Supplement: Supplementary file 1 — In vitro evolution of enhanced RNA replicons for immunotherapy [file 41598_2019_43422_MOESM1_ESM.pdf]

Supplemental Information for:

***In vitro* evolution of enhanced RNA replicons for immunotherapy**

Yingzhong Li, Brian Teague, Yuan Zhang, Zhijun Su, Ely Porter, Brian Dobosh, Tyler Wagner, Darrell J. Irvine, Ron Weiss

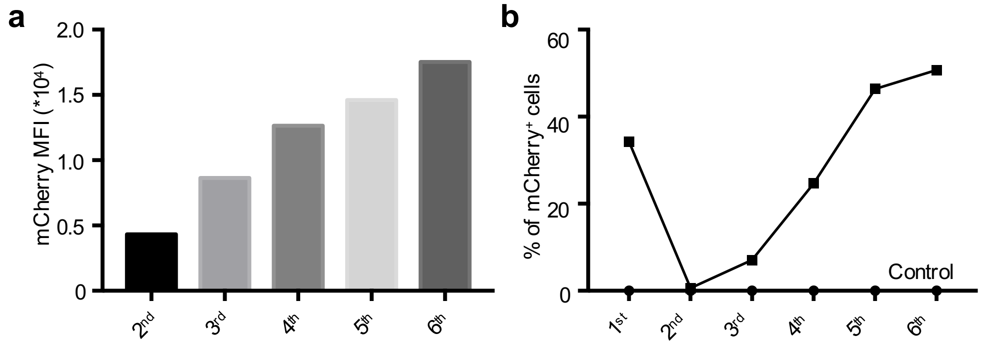

Supplemental Figure 1. *In vitro* evolution increases mCherry expression from replicon RNA. **a**-**b** mCherry MFI (**a**) and percentage of mCherry positive cells (**b**) just before each sorting.

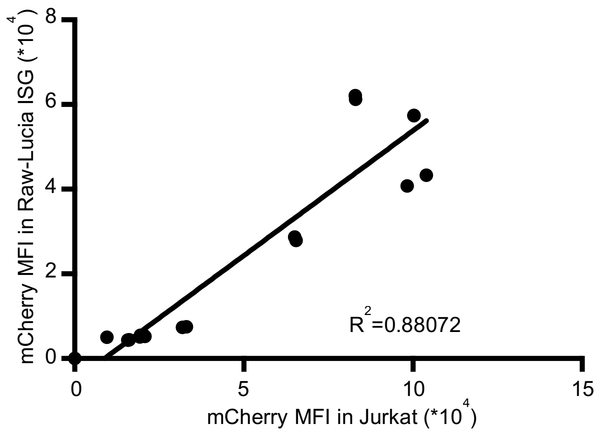

Supplemental Figure 2. (**a**) Correlation between mCherry MFI in Jurkat and in RAW-Lucia ISG cells. Jurkat and Raw-Lucia ISG cells were transfected with WT or mutant replicons encoding mCherry as a reporter. Mean fluorescence intensities (MFI) on day 1 post transfection were determined by flow cytometry. Coefficient of determination (R-squared) is indicated.

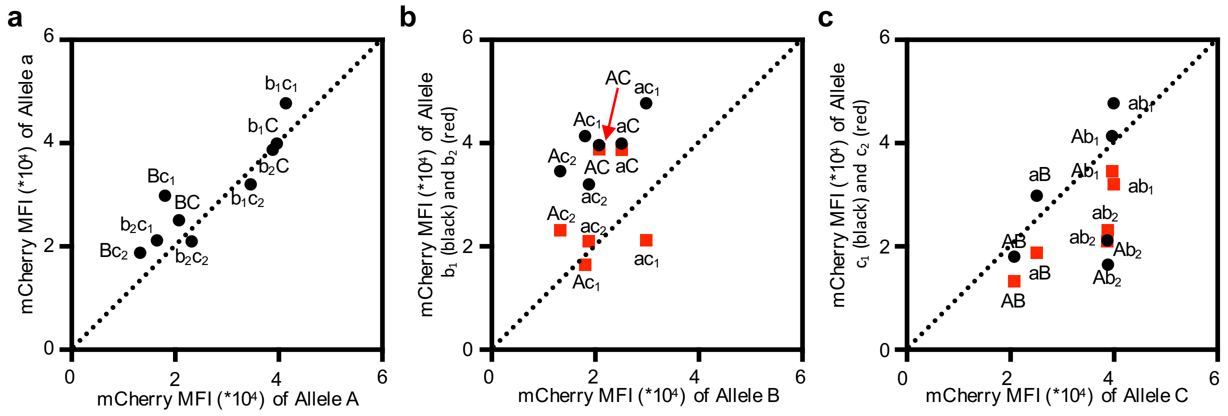

Supplemental Figure 3. Effects of different alleles on mCherry MFI in Raw-Lucia-ISG. Shown are scatter plots of wildtype replicon mCherry MFI (X-axis) versus mCherry MFI of mutant alleles (Y-axis) for (**a**) allele A vs. a, (**b**) B vs. b_1 (black) and b_2 (red), and (**c**) C vs. c_1 (black) and c_2 (red). For each plot, the X-axis is for the wildtype allele mCherry MFI and the Y-axis is for the corresponding mutant allele mCherry MFI. As each of our replicons comprised combinations of three alleles A (or a), B (or b_1, or b_2), and C (or c_1, or c_2), when comparing the effect of any allele mutation, here we only label each dot or square with the other two alleles. For instance, the dot marked as Bc_1 in the leftmost panel is positioned to indicate the comparison between the mCherry MFIs of ABc_1 and aBc_1, where the x-value of this dot is the mCherry MFI of ABc_1 and the y-value of this dot is the mCherry MFI of aBc_1. Dots or squares that are close to the dashed diagonal line indicate that the mutant allele has negligible effect on mCherry MFI in comparison to the wildtype allele. Dots or squares above and to the left of the diagonal line indicate mutant alleles that increase mCherry expression relative to wildtype. Dots or squares below and to the right of the diagonal line indicate that the allele reduces mCherry expression.

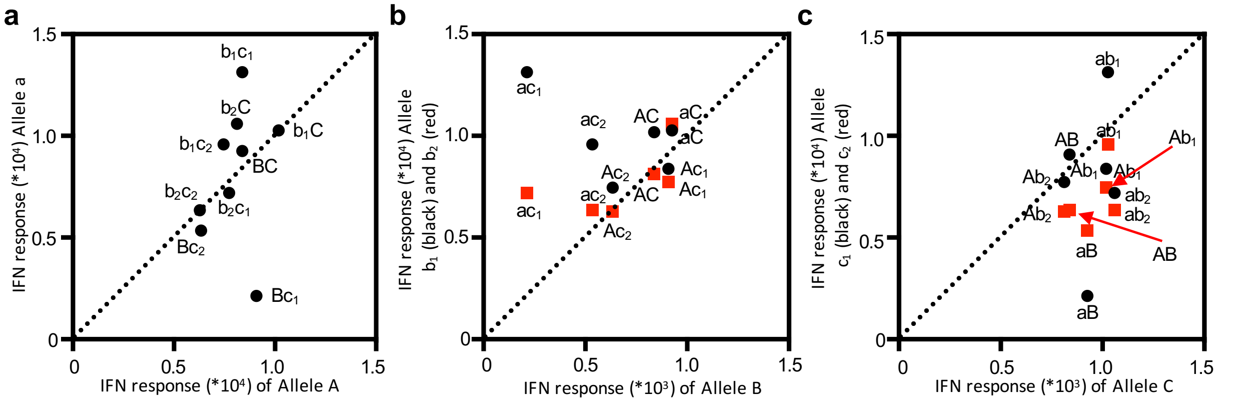

Supplemental Figure 4. Effects of different alleles on IFN responses in Raw-Lucia-ISG. Shown are scatter plots of wildtype replicon IFN response (X-axis) versus IFN response of mutant alleles (Y-axis) for (**a**) allele A vs. a, (**b**) B vs. b_1 (black) and b_2 (red), and (**c**) C vs. c_1 (black) and c_2 (red). For each plot, the X-axis is for the wildtype allele IFN response and the Y-axis is for the corresponding mutant allele IFN response. As each of our replicons comprised combinations of three alleles A (or a), B (or b_1, or b_2), and C (or c_1, or c_2), when comparing the effect of any allele mutation, here we only label each dot or square with the other two alleles. For instance, the dot marked as Bc_1 in the leftmost panel is positioned to indicate the comparison between the IFN responses of ABc_1 and aBc_1, where the x-value of this dot is the IFN response of ABc_1 and the y-value of this dot is the IFN response of aBc_1. Dots or squares that are close to the dashed diagonal line indicate that the allele has negligible effect on IFN response in comparison to the wildtype allele. Dots or squares above and to the left of the diagonal line indicate mutant alleles that increase IFN response relative to wildtype. Dots or squares below and to the right of the diagonal line indicate that the allele reduces IFN response.

Information					Combination																	
Locus	Allele	nucleotide	AA	region	ABC	aBC	Ab_1C	ab_1C	Ab_2C	ab_2C	ABc_1	aBc_1	Ab_1c_1	ab_1c_1	Ab_2c_1	ab_2c_1	ABc_2	aBc_2	Ab_1c_2	ab_1c_2	Ab_2c_2	ab_2c_2
L2	A				A		A		A		A		A		A		A		A		A	
	a	A1979G	G656G	nsP2		a		a		a		a		a		a		a		a		a
L4	B				B	B					B	B					B	B				
	b_1	G3936C	G1309R	nsP2			b_1	b_1					b_1	b_1					b_1	b_1		
	b_2	A4311G	K1434E	nsP3					b_2	b_2					b_2	b_2					b_2	b_2
L5	C				C	C	C	C	C	C												
	c_1	A4758G	S1583G	nsP3							c_1	c_1	c_1	c_1	c_1	c_1						
	c_2	G4796T/ G4944A	E1595D/ V1645M	nsP3													c_2	c_2	c_2	c_2	c_2	c_2

Supplemental Table 1. Description of wildtype alleles and mutant alleles as well as all combinations of these alleles. Locus 2 has wildtype allele A and mutant allele a. Locus 4 has wildtype allele B, mutant alleles b_1 and b_2. Locus 5 has wildtype allele C, mutant alleles c_1 and c_2. Mutant allele c_2 has two linked mutations. The nucleotide and amino acid changes of mutant alleles are indicated. The locations of mutant alleles in the nsPs are also indicated. All 18 combinations of these 8 independent alleles are shown on the right.

YL-mCherry-ClaI-F	atgggcgcgccctcagcatcgattgaattggccaccATGGTGAGCAAGGGCGAGGA
YL-mCherry-R1	TCGCCGCGAGTTCTATGTAAGCAGCTTGCCaattcCTACTTGTACAGCTCGTCCA
YL-mCherry-SphI-R2	AAATAAAATAAAAATTTTAAGGCGGCATGCCAATCGCCGCGAGTTCTATGTAA
YL-locus-5'UTR-F1	TATTGGGCGCTCTTCCGCTGGTCTCACACCATGGGCGGCGCATGAGAGAA
YL-locus-R1	TCAGTGAGCGAGGAAGCGGTCTCATTATACGACTATACGCTGGTTGAG
YL-locus-F2	TATTGGGCGCTCTTCCGCTGGTCTCACACCTGGCAACAGATGTCAGTGCG
YL-locus-R2	TCAGTGAGCGAGGAAGCGGTCTCATTATAATGATGCCAGACTTGCCTG
YL-locus-F3	TATTGGGCGCTCTTCCGCTGGTCTCACACCTACCAAGTACCAACCATAGG
YL-locus-R3	TCAGTGAGCGAGGAAGCGGTCTCATTATTTCAGCCCGTACATGTTAGG
YL-locus-F4	TATTGGGCGCTCTTCCGCTGGTCTCACACCTAATCACTGGGATAACTCCC
YL-locus-R4	TCAGTGAGCGAGGAAGCGGTCTCATTATCATCTGCATCAGTGGTGTCT
YL-locus-F5	TATTGGGCGCTCTTCCGCTGGTCTCACACCTTGAACCATTTGCTGACAGC
YL-locus-R5	TCAGTGAGCGAGGAAGCGGTCTCATTATTCCTCTCTAGTGATCACCCT
YL-locus-F6	TATTGGGCGCTCTTCCGCTGGTCTCACACCAGAACCAGCCTAGTTTCCAC
YL-locus-R6	TCAGTGAGCGAGGAAGCGGTCTCATTATAACCAGCTCTCGGTGGATTC
YL-locus-F7	TATTGGGCGCTCTTCCGCTGGTCTCACACCGCTAGCAACAGCGTATCTGT
YL-locus-R7	TCAGTGAGCGAGGAAGCGGTCTCATTATATGGCCATGTTATCCTCCTC
YL-nsP2-XmaI-F	GCGCGGCTGTTCAAGTTTTC
YL-nsP3-PstI-R	TTTCCCATTTCTTGTCCCTGCAGTATATGGCTACATCTGCATCAGTGGTGTCT
YL-nsP3-PstI-F	ATGCAGATGTAGCCATATAC
YL-nsP4-OL-R	GATCACCCTATTCACGCCTG
YL-nsP4-OL-F	CAGGCGTGAATAGGGTGATC
YL-nsP4-AvrII-R	CTTCTGCCTTCAAATAATGC
YL-nsP2-BsaI-F	TATTGGGCGCTCTTCCGCTGGTCTCACACCAGCATAGGTTATGGTTACGC
YL-nsP3-BsaI-R	TCAGTGAGCGAGGAAGCGGTCTCATTATTACGCAGGCACTTTCGGTGA
YL-nsP3-qPCR-F	ATGCCGTAGGACCAAACTTC
YL-nsP3-qPCR-R	TGGTGTCTAAAGCTGTCAGC
YL-mCherry-F	CTTCAAGGTGCACATGGAGG
YL-mCherry-R	AGGACAGCTTCAAGTAGTCG
YL-hActB-qPCR-F	CACCATTGGCAATGAGCGGTTC
YL-hActB-qPCR-R	AGGTCTTTGCGGATGTCCACGT
YL-mIL2-F	GCGGCATGTTCTGGATTTGACTC
YL-mIL2-R	CCACCACAGTTGCTGACTCATC

Supplemental Table 2. Primers used in this paper.

Plasmid Sequences

```
>ABC-mCherry
ATGGGCGGCGCATGAGAGAAGCCCAGACCAATTACCTACCCAAAATGGAGAAAGTTCACGTTGACATCGAGGAAGACAGCCCATTCCTCAGAGCTTT
GCAGCGGAGCTTCCCGCAGTTTGAGGTAGAAGCCAAGCAGGTCACTGATAATGACCATGCTAATGCCAGAGCGTTTTCGCATCTGGCTTCAAAACTG
ATCGAAACGGAGGTGGACCCATCCGACACGATCCTTGACATTGGAAGTGCGCCCGCCCGCAGAATGTATTCTAAGCACAAGTATCATTGTATCTGTC
CGATGAGATGTGCGGAAGATCCGGACAGATTGTATAAGTATGCAACTAAGCTGAAGAAAAACTGTAAGGAAATAACTGATAAGGAATTGGACAAGAA
AATGAAGGAGCTGGCCGCCGTCATGAGCGACCCTGACCTGGAAACTGAGACTATGTGCCTCCACGACGACGAGTCGTGTCGCTACGAAGGGCAAGTC
GCTGTTTACCAGGATGTATACGCGGTTGACGGACCGACAAGTCTCTATCACCAAGCCAATAAGGGAGTTAGAGTCGCCTACTGGATAGGCTTTGACA
CCACCCCTTTTATGTTTAAGAACTTGGCTGGAGCATATCCATCATACTCTACCAACTGGGCCGACGAAACCGTGTTAACGGCTCGTAACATAGGCCT
ATGCAGCTCTGACGTTATGGAGCGGTCACGTAGAGGGATGTCCATTCTTAGAAAGAAGTATTTGAAACCATCCAACAATGTTCTATTCTCTGTTGGC
TCGACCATCTACCACGAGAAGAGGGACTTACTGAGGAGCTGGCACCTGCCGTCTGTATTTCACTTACGTGGCAAGCAAAATTACACATGTCGGTGTG
AGACTATAGTTAGTTGCGACGGGTACGTCGTTAAAAGAATAGCTATCAGTCCAGGCCTGTATGGGAAGCCTTCAGGCTATGCTGCTACGATGCACCG
CGAGGGATTCTTGTGCTGCAAAGTGACAGACACATTGAACGGGGAGAGGGTCTCTTTTCCCGTGTGCACGTATGTGCCAGCTACATTGTGTGACCAA
ATGACTGGCATACTGGCAACAGATGTCAGTGCGGACGACGCGCAAAAACTGCTGGTTGGGCTCAACCAGCGTATAGTCGTCAACGGTCGCACCCAGA
GAAACACCAATACCATGAAAAATTACCTTTTGCCCGTAGTGGCCCAGGCATTTGCTAGGTGGGCAAAGGAATATAAGGAAGATCAAGAAGATGAAAG
GCCACTAGGACTACGAGATAGACAGTTAGTCATGGGGTGTTGTTGGGCTTTTAGAAGGCACAAGATAACATCTATTTATAAGCGCCCGGATACCCAA
ACCATCATCAAAGTGAACAGCGATTTCCACTCATTCGTGCTGCCCAGGATAGGCAGTAACACATTGGAGATCGGGCTGAGAACAAGAATCAGGAAAA
TGTTAGAGGAGCACAAGGAGCCGTCACCTCTCATTACCGCCGAGGACGTACAAGAAGCTAAGTGCGCAGCCGATGAGGCTAAGGAGGTGCGTGAAGC
CGAGGAGTTGCGCGCAGCTCTACCACCTTTGGCAGCTGATGTTGAGGAGCCCACTCTGGAGGCAGACGTCGACTTGATGTTACAAGAGGCTGGGGCC
GGCTCAGTGGAGACACCTCGTGGCTTGATAAAGGTTACCAGCTACGATGGCGAGGACAAGATCGGCTCTTACGCTGTGCTTTCTCCGCAGGCTGTAC
TCAAGAGTGAAAAATTATCTTGCATCCACCCTCTCGCTGAACAAGTCATAGTGATAACACACTCTGGCCGAAAAGGGCGTTATGCCGTGGAACCATA
CCATGGTAAAGTAGTGGTGCCAGAGGGACATGCAATACCCGTCCAGGACTTTCAAGCTCTGAGTGAAAGTGCCACCATTGTGTACAACGAACGTGAG
TTCGTAAACAGGTACCTGCACCATATTGCCACACATGGAGGAGCGCTGAACACTGATGAAGAATATTACAAAACTGTCAAGCCCAGCGAGCACGACG
GCGAATACCTGTACGACATCGACAGGAAACAGTGCGTCAAGAAAGAACTAGTCACTGGGCTAGGGCTCACAGGCGAGCTGGTGGATCCTCCCTTCCA
TGAATTCGCCTACGAGAGTCTGAGAACACGACCAGCCGCTCCTTACCAAGTACCAACCATAGGGGTGTATGGCGTGCCAGGATCAGGCAAGTCTGGC
ATCATTAAAAGCGCAGTCACCAAAAAAGATCTAGTGGTGAGCGCCAAGAAAGAAAACTGTGCAGAAATTATAAGGGACGTCAAGAAAATGAAAGGGC
TGGACGTCAATGCCAGAACTGTGGACTCAGTGCTCTTGAATGGATGCAAACACCCCGTAGAGACCCTGTATATTGACGAAGCTTTTGCTTGTCATGC
AGGTACTCTCAGAGCGCTCATAGCCATTATAAGACCTAAAAAGGCAGTGCTCTGCGGGGATCCCAAACAGTGCGGTTTTTTAACATGATGTGCCTG
AAAGTGCATTTTAACCACGAGATTTGCACACAAGTCTTCCACAAAAGCATCTCTCGCCGTTGCACTAAATCTGTGACTTCGGTCGTCTCAACCTTGT
TTTACGACAAAAAAATGAGAACGACGAATCCGAAAGAGACTAAGATTGTGATTGACACTACCGGCAGTACCAAACCTAAGCAGGACGATCTCATTCT
CACTTGTTTCAGAGGGTGGGTGAAGCAGTTGCAAATAGATTACAAAGGCAACGAAATAATGACGGCAGCTGCCTCTCAAGGGCTGACCCGTAAAGGT
GTGTATGCCGTTCGGTACAAGGTGAATGAAAATCCTCTGTACGCACCCACCTCAGAACATGTGAACGTCCTACTGACCCGCACGGAGGACCGCATCG
TGTGGAAAACACTAGCCGGCGACCCATGGATAAAAACACTGACTGCCAAGTACCCTGGGAATTTCACTGCCACGATAGAGGAGTGGCAAGCAGAGCA
TGATGCCATCATGAGGCACATCTTGGAGAGACCGGACCCTACCGACGTCTTCCAGAATAAGGCAAACGTGTGTTGGGCCAAGGCTTTAGTGCCGGTG
CTGAAGACCGCTGGCATAGACATGACCACTGAACAATGGAACACTGTGGATTATTTTGAAACGGACAAAGCTCACTCAGCAGAGATAGTATTGAACC
AACTATGCGTGAGGTTCTTTGGACTCGATCTGGACTCCGGTCTATTTTCTGCACCCACTGTTCCGTTATCCATTAGGAATAATCACTGGGATAACTC
CCCGTCGCCTAACATGTACGGGCTGAATAAAGAAGTGGTCCGTCAGCTCTCTCGCAGGTACCCACAACTGCCTCGGGCAGTTGCCACTGGAAGAGTC
TATGACATGAACACTGGTACACTGCGCAATTATGATCCGCGCATAAACCTAGTACCTGTAAACAGAAGACTGCCTCATGCTTTAGTCCTCCACCATA
ATGAACACCCACAGAGTGACTTTTCTTCATTCGTCAGCAAATTGAAGGGCAGAACTGTCCTGGTGGTCGGGGAAAAGTTGTCCGTCCCAGGCAAAAT
GGTTGACTGGTTGTCAGACCGGCCTGAGGCTACCTTCAGAGCTCGGCTGGATTTAGGCATCCCAGGTGATGTGCCCAAATATGACATAATATTTGTT
AATGTGAGGACCCCATATAAATACCATCACTATCAGCAGTGTGAAGACCATGCCATTAAGCTTAGCATGTTGACCAAGAAAGCTTGTCTGCATCTGA
ATCCCGGCGGAACCTGTGTCAGCATAGGTTATGGTTACGCTGACAGGGCCAGCGAAAGCATCATTGGTGCTATAGCGCGGCAGTTCAAGTTTTCCCG
GGTATGCAAACCGAAATCCTCACTTGAAGAGACGGAAGTTCTGTTTGTATTCATTGGGTACGATCGCAAGGCCCGTACGCACAATTCTTACAAGCTT
TCATCAACCTTGACCAACATTTATACAGGTTCCAGACTCCACGAAGCCGGATGTGCACCCTCATATCATGTGGTGCGAGGGGATATTGCCACGGCCA
CCGAAGGAGTGATTATAAATGCTGCTAACAGCAAAGGACAACCTGGCGGAGGGGTGTGCGGAGCGCTGTATAAGAAATTCCCGGAAAGCTTCGATTT
ACAGCCGATCGAAGTAGGAAAAGCGCGACTGGTCAAAGGTGCAGCTAAACATATCATTCATGCCGTAGGACCAAACTTCAACAAAGTTTCGGAGGTT
GAAGGTGACAAACAGTTGGCAGAGGCTTATGAGTCCATCGCTAAGATTGTCAACGATAACAATTACAAGTCAGTAGCGATTCCACTGTTGTCCACCG
GCATCTTTTCCGGGAACAAAGATCGACTAACCCAATCATTGAACCATTTGCTGACAGCTTTAGACACCACTGATGCAGATGTAGCCATATACTGCAG
GGACAAGAAATGGGAAATGACTCTCAAGGAAGCAGTGGCTAGGAGAGAAGCAGTGGAGGAGATATGCATATCCGACGACTCTTCAGTGACAGAACCT
GATGCAGAGCTGGTGAGGGTGCATCCGAAGAGTTCTTTGGCTGGAAGGAAGGGCTACAGCACAAGCGATGGCAAAACTTTCTCATATTTGGAAGGGA
CCAAGTTTCACCAGGCGGCCAAGGATATAGCAGAAATTAATGCCATGTGGCCCGTTGCAACGGAGGCCAATGAGCAGGTATGCATGTATATCCTCGG
AGAAAGCATGAGCAGTATTAGGTCGAAATGCCCCGTCGAAGAGTCGGAAGCCTCCACACCACCTAGCACGCTGCCTTGCTTGTGCATCCATGCCATG
ACTCCAGAAAGAGTACAGCGCCTAAAAGCCTCACGTCCAGAACAAATTACTGTGTGCTCATCCTTTCCATTGCCGAAGTATAGAATCACTGGTGTGC
AGAAGATCCAATGCTCCCAGCCTATATTGTTCTCACCGAAAGTGCCTGCGTATATTCATCCAAGGAAGTATCTCGTGGAAACACCACCGGTAGACGA
GACTCCGGAGCCATCGGCAGAGAACCAATCCACAGAGGGGACACCTGAACAACCACCACTTATAACCGAGGATGAGACCAGGACTAGAACGCCTGAG
CCGATCATCATCGAAGAGGAAGAAGAGGATAGCATAAGTTTGCTGTCAGATGGCCCGACCCACCAGGTGCTGCAAGTCGAGGCAGACATTCACGGGC
CGCCCTCTGTATCTAGCTCATCCTGGTCCATTCCTCATGCATCCGACTTTGATGTGGACAGTTTATCCATACTTGACACCCTGGAGGGAGCTAGCGT
GACCAGCGGGGCAACGTCAGCCGAGACTAACTCTTACTTCGCAAAGAGTATGGAGTTTCTGGCGCGACCGGTGCCTGCGCCTCGAACAGTATTCAGG
AACCCTCCACATCCCGCTCCGCGCACAAGAACACCGTCACTTGCACCCAGCAGGGCCTGCTCGAGAACCAGCCTAGTTTCCACCCCGCCAGGCGTGA
ATAGGGTGATCACTAGAGAGGAGCTCGAGGCGCTTACCCCGTCACGCACTCCTAGCAGGTCGGTCTCGAGAACCAGCCTGGTCTCCAACCCGCCAGG
CGTAAATAGGGTGATTACAAGAGAGGAGTTTGAGGCGTTCGTAGCACAACAACAATGACGGTTTGATGCGGGTGCATACATCTTTTCCTCCGACACC
GGTCAAGGGCATTTACAACAAAAATCAGTAAGGCAAACGGTGCTATCCGAAGTGGTGTTGGAGAGGACCGAATTGGAGATTTCGTATGCCCCGCGCC
TCGACCAAGAAAAAGAAGAATTACTACGCAAGAAATTACAGTTAAATCCCACACCTGCTAACAGAAGCAGATACCAGTCCAGGAAGGTGGAGAACAT
GAAAGCCATAACAGCTAGACGTATTCTGCAAGGCCTAGGGCATTATTTGAAGGCAGAAGGAAAAGTGGAGTGCTACCGAACCCTGCATCCTGTTCCT
TTGTATTCATCTAGTGTGAACCGTGCCTTTTCAAGCCCCAAGGTCGCAGTGGAAGCCTGTAACGCCATGTTGAAAGAGAACTTTCCGACTGTGGCTT
CTTACTGTATTATTCCAGAGTACGATGCCTATTTGGACATGGTTGACGGAGCTTCATGCTGCTTAGACACTGCCAGTTTTTGCCCTGCAAAGCTGCG
CAGCTTTCCAAAGAAACACTCCTATTTGGAACCCACAATACGATCGGCAGTGCCTTCAGCGATCCAGAACACGCTCCAGAACGTCCTGGCAGCTGCC
ACAAAAAGAAATTGCAATGTCACGCAAATGAGAGAATTGCCCGTATTGGATTCGGCGGCCTTTAATGTGGAATGCTTCAAGAAATATGCGTGTAATA
ATGAATATTGGGAAACGTTTAAAGAAAACCCCATCAGGCTTACTGAAGAAAACGTGGTAAATTACATTACCAAATTAAAAGGACCAAAAGCTGCTGC
TCTTTTTGCGAAGACACATAATTTGAATATGTTGCAGGACATACCAATGGACAGGTTTGTAATGGACTTAAAGAGAGACGTGAAAGTGACTCCAGGA
```

```
ACAAAACATACTGAAGAACGGCCCAAGGTACAGGTGATCCAGGCTGCCGATCCGCTAGCAACAGCGTATCTGTGCGGAATCCACCGAGAGCTGGTTA
GGAGATTAAATGCGGTCCTGCTTCCGAACATTCATACACTGTTTGATATGTCGGCTGAAGACTTTGACGCTATTATAGCCGAGCACTTCCAGCCTGG
GGATTGTGTTCTGGAAACTGACATCGCGTCGTTTGATAAAAGTGAGGACGACGCCATGGCTCTGACCGCGTTAATGATTCTGGAAGACTTAGGTGTG
GACGCAGAGCTGTTGACGCTGATTGAGGCGGCTTTCGGCGAAATTTCATCAATACATTTGCCCACTAAAACTAAATTTAAATTCGGAGCCATGATGA
AATCTGGAATGTTCCTCACACTGTTTGTGAACACAGTCATTAACATTGTAATCGCAAGCAGAGTGTTGAGAGAACGGCTAACCGGATCACCATGTGC
AGCATTCATTGGAGATGACAATATCGTGAAAGGAGTCAAATCGGACAAATTAATGGCAGACAGGTGCGCCACCTGGTTGAATATGGAAGTCAAGATT
ATAGATGCTGTGGTGGGCGAGAAAGCGCCTTATTTCTGTGGAGGGTTTATTTTGTGTGACTCCGTGACCGGCACAGCGTGCCGTGTGGCAGACCCCC
TAAAAAGGCTGTTTAAGCTTGGCAAACCTCTGGCAGCAGACGATGAACATGATGATGACAGGAGAAGGGCATTGCATGAAGAGTCAACACGCTGGAA
CCGAGTGGGTATTCTTTCAGAGCTGTGCAAGGCAGTAGAATCAAGGTATGAAACCGTAGGAACTTCCATCATAGTTATGGCCATGACTACTCTAGCT
AGCAGTGTTAAATCATTCAGCTACCTGAGAGGGGCCCCTATAACTCTCTACGGCTAACCTGAATGGACTACGACATAGTCTAGTCCGCCAAGTCTAG
catatgggcgcgccctcagcatcgattgaattggccaccATGGTGAGCAAGGGCGAGGAGGATAACATGGCCATCATCAAGGAGTTCATGCGCTTCA
AGGTGCACATGGAGGGCTCCGTGAACGGCCACGAGTTCGAGATCGAGGGCGAGGGCGAGGGCCGCCCCTACGAGGGCACCCAGACCGCCAAGCTGAA
GGTGACCAAGGGTGGCCCCCTGCCCTTCGCCTGGGACATCCTGTCCCCTCAGTTCATGTACGGCTCCAAGGCCTACGTGAAGCACCCCGCCGACATC
CCCGACTACTTGAAGCTGTCCTTCCCCGAGGGCTTCAAGTGGGAGCGCGTGATGAACTTCGAGGACGGCGGCGTGGTGACCGTGACCCAGGACTCCT
CCCTGCAGGACGGCGAGTTCATCTACAAGGTGAAGCTGCGCGGCACCAACTTCCCCTCCGACGGCCCCGTAATGCAGAAGAAGACCATGGGCTGGGA
GGCCTCCTCCGAGCGGATGTACCCCGAGGACGGCGCCCTGAAGGGCGAGATCAAGCAGAGGCTGAAGCTGAAGGACGGCGGCCACTACGACGCTGAG
GTCAAGACCACCTACAAGGCCAAGAAGCCCGTGCAGCTGCCCGGCGCCTACAACGTCAACATCAAGTTGGACATCACCTCCCACAACGAGGACTACA
CCATCGTGGAACAGTACGAACGCGCCGAGGGCCGCCACTCCACCGGCGGCATGGACGAGCTGTACAAGTAGgaattGGCAAGCTGCTTACATAGAAC
TCGCGGCGATTGGCATGCCGCCTTAAAATTTTTATTTTATTTTTCTTTTCTTTTCCGAATCGGATTTTGTTTTTAATATTTCAAAAAAAAAAAAAAA
AAAAAAAAAAACGCGTCGAGGGGAATTAATTCTTGAAGACGAAAGGGCCAGGTGGCACTTTTCGGGGAAATGTGCGCGGAACCCCTATTTGTTTATT
TTTCTAAATACATTCAAATATGTATCCGCTCATGAGACAATAACCCTGATAAATGCTTCAATAATATTGAAAAAGGAAGAGTATGAGTATTCAACAT
TTCCGTGTCGCCCTTATTCCCTTTTTTGCGGCATTTTGCCTTCCTGTTTTTGCTCACCCAGAAACGCTGGTGAAAGTAAAAGATGCTGAAGATCAGT
TGGGTGCACGAGTGGGTTACATCGAACTGGATCTCAACAGCGGTAAGATCCTTGAGAGTTTTCGCCCCGAAGAACGTTTTCCAATGATGAGCACTTT
TAAAGTTCTGCTATGTGGCGCGGTATTATCCCGTGTTGACGCCGGGCAAGAGCAACTCGGTCGCCGCATACACTATTCTCAGAATGACTTGGTTGAG
TACTCACCAGTCACAGAAAAGCATCTTACGGATGGCATGACAGTAAGAGAATTATGCAGTGCTGCCATAACCATGAGTGATAACACTGCGGCCAACT
TACTTCTGACAACGATCGGAGGACCGAAGGAGCTAACCGCTTTTTTGCACAACATGGGGGATCATGTAACTCGCCTTGATCGTTGGGAACCGGAGCT
GAATGAAGCCATACCAAACGACGAGCGTGACACCACGATGCCTGTAGCAATGGCAACAACGTTGCGCAAACTATTAACTGGCGAACTACTTACTCTA
GCTTCCCGGCAACAATTAATAGACTGGATGGAGGCGGATAAAGTTGCAGGACCACTTCTGCGCTCGGCCCTTCCGGCTGGCTGGTTTATTGCTGATA
AATCTGGAGCCGGTGAGCGTGGGTCTCGCGGTATCATTGCAGCACTGGGGCCAGATGGTAAGCCCTCCCGTATCGTAGTTATCTACACGACGGGGAG
TCAGGCAACTATGGATGAACGAAATAGACAGATCGCTGAGATAGGTGCCTCACTGATTAAGCATTGGTAACTGTCAGACCAAGTTTACTCATATATA
CTTTAGATTGATTTAAAACTTCATTTTTAATTTAAAAGGATCTAGGTGAAGATCCTTTTTGATAATCTCATGACCAAAATCCCTTAACGTGAGTTTT
CGTTCCACTGAGCGTCAGACCCCGTAGAAAAGATCAAAGGATCTTCTTGAGATCCTTTTTTTCTGCGCGTAATCTGCTGCTTGCAAACAAAAAAACC
ACCGCTACCAGCGGTGGTTTGTTTGCCGGATCAAGAGCTACCAACTCTTTTTCCGAAGGTAACTGGCTTCAGCAGAGCGCAGATACCAAATACTGTC
CTTCTAGTGTAGCCGTAGTTAGGCCACCACTTCAAGAACTCTGTAGCACCGCCTACATACCTCGCTCTGCTAATCCTGTTACCAGTGGCTGCTGCCA
GTGGCGATAAGTCGTGTCTTACCGGGTTGGACTCAAGACGATAGTTACCGGATAAGGCGCAGCGGTCGGGCTGAACGGGGGGTTCGTGCACACAGCC
CAGCTTGGAGCGAACGACCTACACCGAACTGAGATACCTACAGCGTGAGCATTGAGAAAGCGCCACGCTTCCCGAAGGGAGAAAGGCGGACAGGTAT
CCGGTAAGCGGCAGGGTCGGAACAGGAGAGCGCACGAGGGAGCTTCCAGGGGGAAACGCCTGGTATCTTTATAGTCCTGTCGGGTTTCGCCACCTCT
GACTTGAGCGTCGATTTTTGTGATGCTCGTCAGGGGGGCGGAGCCTATGGAAAAACGCCAGCAACGCGAGCTCTAATACGACTCACTATAG

>aBC-mCherry
ATGGGCGGCGCATGAGAGAAGCCCAGACCAATTACCTACCCAAAATGGAGAAAGTTCACGTTGACATCGAGGAAGACAGCCCATTCCTCAGAGCTTT
GCAGCGGAGCTTCCCGCAGTTTGAGGTAGAAGCCAAGCAGGTCACTGATAATGACCATGCTAATGCCAGAGCGTTTTCGCATCTGGCTTCAAAACTG
ATCGAAACGGAGGTGGACCCATCCGACACGATCCTTGACATTGGAAGTGCGCCCGCCCGCAGAATGTATTCTAAGCACAAGTATCATTGTATCTGTC
CGATGAGATGTGCGGAAGATCCGGACAGATTGTATAAGTATGCAACTAAGCTGAAGAAAAACTGTAAGGAAATAACTGATAAGGAATTGGACAAGAA
AATGAAGGAGCTGGCCGCCGTCATGAGCGACCCTGACCTGGAAACTGAGACTATGTGCCTCCACGACGACGAGTCGTGTCGCTACGAAGGGCAAGTC
GCTGTTTACCAGGATGTATACGCGGTTGACGGACCGACAAGTCTCTATCACCAAGCCAATAAGGGAGTTAGAGTCGCCTACTGGATAGGCTTTGACA
CCACCCCTTTTATGTTTAAGAACTTGGCTGGAGCATATCCATCATACTCTACCAACTGGGCCGACGAAACCGTGTTAACGGCTCGTAACATAGGCCT
ATGCAGCTCTGACGTTATGGAGCGGTCACGTAGAGGGATGTCCATTCTTAGAAAGAAGTATTTGAAACCATCCAACAATGTTCTATTCTCTGTTGGC
TCGACCATCTACCACGAGAAGAGGGACTTACTGAGGAGCTGGCACCTGCCGTCTGTATTTCACTTACGTGGCAAGCAAAATTACACATGTCGGTGTG
AGACTATAGTTAGTTGCGACGGGTACGTCGTTAAAAGAATAGCTATCAGTCCAGGCCTGTATGGGAAGCCTTCAGGCTATGCTGCTACGATGCACCG
CGAGGGATTCTTGTGCTGCAAAGTGACAGACACATTGAACGGGGAGAGGGTCTCTTTTCCCGTGTGCACGTATGTGCCAGCTACATTGTGTGACCAA
ATGACTGGCATACTGGCAACAGATGTCAGTGCGGACGACGCGCAAAAACTGCTGGTTGGGCTCAACCAGCGTATAGTCGTCAACGGTCGCACCCAGA
GAAACACCAATACCATGAAAAATTACCTTTTGCCCGTAGTGGCCCAGGCATTTGCTAGGTGGGCAAAGGAATATAAGGAAGATCAAGAAGATGAAAG
GCCACTAGGACTACGAGATAGACAGTTAGTCATGGGGTGTTGTTGGGCTTTTAGAAGGCACAAGATAACATCTATTTATAAGCGCCCGGATACCCAA
ACCATCATCAAAGTGAACAGCGATTTCCACTCATTCGTGCTGCCCAGGATAGGCAGTAACACATTGGAGATCGGGCTGAGAACAAGAATCAGGAAAA
TGTTAGAGGAGCACAAGGAGCCGTCACCTCTCATTACCGCCGAGGACGTACAAGAAGCTAAGTGCGCAGCCGATGAGGCTAAGGAGGTGCGTGAAGC
CGAGGAGTTGCGCGCAGCTCTACCACCTTTGGCAGCTGATGTTGAGGAGCCCACTCTGGAGGCAGACGTCGACTTGATGTTACAAGAGGCTGGGGCC
GGCTCAGTGGAGACACCTCGTGGCTTGATAAAGGTTACCAGCTACGATGGCGAGGACAAGATCGGCTCTTACGCTGTGCTTTCTCCGCAGGCTGTAC
TCAAGAGTGAAAAATTATCTTGCATCCACCCTCTCGCTGAACAAGTCATAGTGATAACACACTCTGGCCGAAAAGGGCGTTATGCCGTGGAACCATA
CCATGGTAAAGTAGTGGTGCCAGAGGGACATGCAATACCCGTCCAGGACTTTCAAGCTCTGAGTGAAAGTGCCACCATTGTGTACAACGAACGTGAG
TTCGTAAACAGGTACCTGCACCATATTGCCACACATGGGGGAGCGCTGAACACTGATGAAGAATATTACAAAACTGTCAAGCCCAGCGAGCACGACG
GCGAATACCTGTACGACATCGACAGGAAACAGTGCGTCAAGAAAGAACTAGTCACTGGGCTAGGGCTCACAGGCGAGCTGGTGGATCCTCCCTTCCA
TGAATTCGCCTACGAGAGTCTGAGAACACGACCAGCCGCTCCTTACCAAGTACCAACCATAGGGGTGTATGGCGTGCCAGGATCAGGCAAGTCTGGC
ATCATTAAAAGCGCAGTCACCAAAAAAGATCTAGTGGTGAGCGCCAAGAAAGAAAACTGTGCAGAAATTATAAGGGACGTCAAGAAAATGAAAGGGC
TGGACGTCAATGCCAGAACTGTGGACTCAGTGCTCTTGAATGGATGCAAACACCCCGTAGAGACCCTGTATATTGACGAAGCTTTTGCTTGTCATGC
AGGTACTCTCAGAGCGCTCATAGCCATTATAAGACCTAAAAAGGCAGTGCTCTGCGGGGATCCCAAACAGTGCGGTTTTTTTAACATGATGTGCCTG
AAAGTGCATTTTAACCACGAGATTTGCACACAAGTCTTCCACAAAAGCATCTCTCGCCGTTGCACTAAATCTGTGACTTCGGTCGTCTCAACCTTGT
TTTACGACAAAAAAATGAGAACGACGAATCCGAAAGAGACTAAGATTGTGATTGACACTACCGGCAGTACCAAACCTAAGCAGGACGATCTCATTCT
CACTTGTTTCAGAGGGTGGGTGAAGCAGTTGCAAATAGATTACAAAGGCAACGAAATAATGACGGCAGCTGCCTCTCAAGGGCTGACCCGTAAAGGT
GTGTATGCCGTTCGGTACAAGGTGAATGAAAATCCTCTGTACGCACCCACCTCAGAACATGTGAACGTCCTACTGACCCGCACGGAGGACCGCATCG
TGTGGAAAACACTAGCCGGCGACCCATGGATAAAAACACTGACTGCCAAGTACCCTGGGAATTTCACTGCCACGATAGAGGAGTGGCAAGCAGAGCA
TGATGCCATCATGAGGCACATCTTGGAGAGACCGGACCCTACCGACGTCTTCCAGAATAAGGCAAACGTGTGTTGGGCCAAGGCTTTAGTGCCGGTG
```

CTGAAGACCGCTGGCATAGACATGACCACTGAACAATGGAACACTGTGGATTATTTTGAAACGGACAAAGCTCACTCAGCAGAGATAGTATTGAACC
AACTATGCGTGAGGTTCTTTGGACTCGATCTGGACTCCGGTCTATTTTCTGCACCCACTGTTCCGTTATCCATTAGGAATAATCACTGGGATAACTC
CCCGTCGCCTAACATGTACGGGCTGAATAAAGAAGTGGTCCGTCAGCTCTCTCGCAGGTACCCACAACTGCCTCGGGCAGTTGCCACTGGAAGAGTC
TATGACATGAACACTGGTACACTGCGCAATTATGATCCGCGCATAAACCTAGTACCTGTAAACAGAAGACTGCCTCATGCTTTAGTCCTCCACCATA
ATGAACACCCACAGAGTGACTTTTCTTCATTCGTCAGCAAATTGAAGGGCAGAACTGTCCTGGTGGTCGGGGAAAAGTTGTCCGTCCCAGGCAAAAT
GGTTGACTGGTTGTCAGACCGGCCTGAGGCTACCTTCAGAGCTCGGCTGGATTTAGGCATCCCAGGTGATGTGCCCAAATATGACATAATATTTGTT
AATGTGAGGACCCCATATAAATACCATCACTATCAGCAGTGTGAAGACCATGCCATTAAGCTTAGCATGTTGACCAAGAAAGCTTGTCTGCATCTGA
ATCCCGGCGGAACCTGTGTCAGCATAGGTTATGGTTACGCTGACAGGGCCAGCGAAAGCATCATTGGTGCTATAGCGCGGCAGTTCAAGTTTTCCCG
GGTATGCAAACCGAAATCCTCACTTGAAGAGACGGAAGTTCTGTTTGTATTCATTGGGTACGATCGCAAGGCCCGTACGCACAATTCTTACAAGCTT
TCATCAACCTTGACCAACATTTATACAGGTTCCAGACTCCACGAAGCCGGATGTGCACCCTCATATCATGTGGTGCGAGGGGATATTGCCACGGCCA
CCGAAGGAGTGATTATAAATGCTGCTAACAGCAAAGGACAACCTGGCGGAGGGGTGTGCGGAGCGCTGTATAAGAAATTCCCGGAAAGCTTCGATTT
ACAGCCGATCGAAGTAGGAAAAGCGCGACTGGTCAAAGGTGCAGCTAAACATATCATTCATGCCGTAGGACCAAACTTCAACAAAGTTTCGGAGGTT
GAAGGTGACAAACAGTTGGCAGAGGCTTATGAGTCCATCGCTAAGATTGTCAACGATAACAATTACAAGTCAGTAGCGATTCCACTGTTGTCCACCG
GCATCTTTTCCGGGAACAAAGATCGACTAACCCAATCATTGAACCATTTGCTGACAGCTTTAGACACCACTGATGCAGATGTAGCCATATACTGCAG
GGACAAGAAATGGGAAATGACTCTCAAGGAAGCAGTGGCTAGGAGAGAAGCAGTGGAGGAGATATGCATATCCGACGACTCTTCAGTGACAGAACCT
GATGCAGAGCTGGTGAGGGTGCATCCGAAGAGTTCTTTGGCTGGAAGGAAGGGCTACAGCACAAGCGATGGCAAAACTTTCTCATATTTGGAAGGGA
CCAAGTTTCACCAGGCGGCCAAGGATATAGCAGAAATTAATGCCATGTGGCCCGTTGCAACGGAGGCCAATGAGCAGGTATGCATGTATATCCTCGG
AGAAAGCATGAGCAGTATTAGGTCGAAATGCCCCGTCGAAGAGTCGGAAGCCTCCACACCACCTAGCACGCTGCCTTGCTTGTGCATCCATGCCATG
ACTCCAGAAAGAGTACAGCGCCTAAAAGCCTCACGTCCAGAACAAATTACTGTGTGCTCATCCTTTCCATTGCCGAAGTATAGAATCACTGGTGTGC
AGAAGATCCAATGCTCCCAGCCTATATTGTTCTCACCGAAAGTGCCTGCGTATATTCATCCAAGGAAGTATCTCGTGGAAACACCACCGGTAGACGA
GACTCCGGAGCCATCGGCAGAGAACCAATCCACAGAGGGGACACCTGAACAACCACCACTTATAACCGAGGATGAGACCAGGACTAGAACGCCTGAG
CCGATCATCATCGAAGAGGAAGAAGAGGATAGCATAAGTTTGCTGTCAGATGGCCCGACCCACCAGGTGCTGCAAGTCGAGGCAGACATTCACGGGC
CGCCCTCTGTATCTAGCTCATCCTGGTCCATTCCTCATGCATCCGACTTTGATGTGGACAGTTTATCCATACTTGACACCCTGGAGGGAGCTAGCGT
GACCAGCGGGGCAACGTCAGCCGAGACTAACTCTTACTTCGCAAAGAGTATGGAGTTTCTGGCGCGACCGGTGCCTGCGCCTCGAACAGTATTCAGG
AACCCTCCACATCCCGCTCCGCGCACAAGAACACCGTCACTTGCACCCAGCAGGGCCTGCTCGAGAACCAGCCTAGTTTCCACCCCGCCAGGCGTGA
ATAGGGTGATCACTAGAGAGGAGCTCGAGGCGCTTACCCCGTCACGCACTCCTAGCAGGTCGGTCTCGAGAACCAGCCTGGTCTCCAACCCGCCAGG
CGTAAATAGGGTGATTACAAGAGAGGAGTTTGAGGCGTTCGTAGCACAACAACAATGACGGTTTGATGCGGGTGCATACATCTTTTCCTCCGACACC
GGTCAAGGGCATTTACAACAAAAATCAGTAAGGCAAACGGTGCTATCCGAAGTGGTGTTGGAGAGGACCGAATTGGAGATTTCGTATGCCCCGCGCC
TCGACCAAGAAAAAGAAGAATTACTACGCAAGAAATTACAGTTAAATCCCACACCTGCTAACAGAAGCAGATACCAGTCCAGGAAGGTGGAGAACAT
GAAAGCCATAACAGCTAGACGTATTCTGCAAGGCCTAGGGCATTATTTGAAGGCAGAAGGAAAAGTGGAGTGCTACCGAACCCTGCATCCTGTTCCT
TTGTATTCATCTAGTGTGAACCGTGCCTTTTCAAGCCCCAAGGTCGCAGTGGAAGCCTGTAACGCCATGTTGAAAGAGAACTTTCCGACTGTGGCTT
CTTACTGTATTATTCCAGAGTACGATGCCTATTTGGACATGGTTGACGGAGCTTCATGCTGCTTAGACACTGCCAGTTTTTGCCCTGCAAAGCTGCG
CAGCTTTCCAAAGAAACACTCCTATTTGGAACCCACAATACGATCGGCAGTGCCTTCAGCGATCCAGAACACGCTCCAGAACGTCCTGGCAGCTGCC
ACAAAAAGAAATTGCAATGTCACGCAAATGAGAGAATTGCCCGTATTGGATTCGGCGGCCTTTAATGTGGAATGCTTCAAGAAATATGCGTGTAATA
ATGAATATTGGGAAACGTTTAAAGAAAACCCCATCAGGCTTACTGAAGAAAACGTGGTAAATTACATTACCAAATTAAAAGGACCAAAAGCTGCTGC
TCTTTTTGCGAAGACACATAATTTGAATATGTTGCAGGACATACCAATGGACAGGTTTGTAATGGACTTAAAGAGAGACGTGAAAGTGACTCCAGGA
ACAAAACATACTGAAGAACGGCCCAAGGTACAGGTGATCCAGGCTGCCGATCCGCTAGCAACAGCGTATCTGTGCGGAATCCACCGAGAGCTGGTTA
GGAGATTAAATGCGGTCCTGCTTCCGAACATTCATACACTGTTTGATATGTCGGCTGAAGACTTTGACGCTATTATAGCCGAGCACTTCCAGCCTGG
GGATTGTGTTCTGGAAACTGACATCGCGTCGTTTGATAAAAGTGAGGACGACGCCATGGCTCTGACCGCGTTAATGATTCTGGAAGACTTAGGTGTG
GACGCAGAGCTGTTGACGCTGATTGAGGCGGCTTTCGGCGAAATTTCATCAATACATTTGCCCACTAAAACTAAATTTAAATTCGGAGCCATGATGA
AATCTGGAATGTTCCTCACACTGTTTGTGAACACAGTCATTAACATTGTAATCGCAAGCAGAGTGTTGAGAGAACGGCTAACCGGATCACCATGTGC
AGCATTCATTGGAGATGACAATATCGTGAAAGGAGTCAAATCGGACAAATTAATGGCAGACAGGTGCGCCACCTGGTTGAATATGGAAGTCAAGATT
ATAGATGCTGTGGTGGGCGAGAAAGCGCCTTATTTCTGTGGAGGGTTTATTTTGTGTGACTCCGTGACCGGCACAGCGTGCCGTGTGGCAGACCCCC
TAAAAAGGCTGTTTAAGCTTGGCAAACCTCTGGCAGCAGACGATGAACATGATGATGACAGGAGAAGGGCATTGCATGAAGAGTCAACACGCTGGAA
CCGAGTGGGTATTCTTTCAGAGCTGTGCAAGGCAGTAGAATCAAGGTATGAAACCGTAGGAACTTCCATCATAGTTATGGCCATGACTACTCTAGCT
AGCAGTGTTAAATCATTCAGCTACCTGAGAGGGGCCCCTATAACTCTCTACGGCTAACCTGAATGGACTACGACATAGTCTAGTCCGCCAAGTCTAG
catatgggcgcgccctcagcatcgattgaattggccaccATGGTGAGCAAGGGCGAGGAGGATAACATGGCCATCATCAAGGAGTTCATGCGCTTCA
AGGTGCACATGGAGGGCTCCGTGAACGGCCACGAGTTCGAGATCGAGGGCGAGGGCGAGGGCCGCCCCTACGAGGGCACCCAGACCGCCAAGCTGAA
GGTGACCAAGGGTGGCCCCCTGCCCTTCGCCTGGGACATCCTGTCCCCTCAGTTCATGTACGGCTCCAAGGCCTACGTGAAGCACCCCGCCGACATC
CCCGACTACTTGAAGCTGTCCTTCCCCGAGGGCTTCAAGTGGGAGCGCGTGATGAACTTCGAGGACGGCGGCGTGGTGACCGTGACCCAGGACTCCT
CCCTGCAGGACGGCGAGTTCATCTACAAGGTGAAGCTGCGCGGCACCAACTTCCCCTCCGACGGCCCCGTAATGCAGAAGAAGACCATGGGCTGGGA
GGCCTCCTCCGAGCGGATGTACCCCGAGGACGGCGCCCTGAAGGGCGAGATCAAGCAGAGGCTGAAGCTGAAGGACGGCGGCCACTACGACGCTGAG
GTCAAGACCACCTACAAGGCCAAGAAGCCCGTGCAGCTGCCCGGCGCCTACAACGTCAACATCAAGTTGGACATCACCTCCCACAACGAGGACTACA
CCATCGTGGAACAGTACGAACGCGCCGAGGGCCGCCACTCCACCGGCGGCATGGACGAGCTGTACAAGTAGgaattGGCAAGCTGCTTACATAGAAC
TCGCGGCGATTGGCATGCCGCCTTAAAATTTTTATTTTATTTTTCTTTTCTTTTCCGAATCGGATTTTGTTTTTAATATTTCAAAAAAAAAAAAAAA
AAAAAAAAAAACGCGTCGAGGGGAATTAATTCTTGAAGACGAAAGGGCCAGGTGGCACTTTTCGGGGAAATGTGCGCGGAACCCCTATTTGTTTATT
TTTCTAAATACATTCAAATATGTATCCGCTCATGAGACAATAACCCTGATAAATGCTTCAATAATATTGAAAAAGGAAGAGTATGAGTATTCAACAT
TTCCGTGTCGCCCTTATTCCCTTTTTTGCGGCATTTTGCCTTCCTGTTTTTGCTCACCCAGAAACGCTGGTGAAAGTAAAAGATGCTGAAGATCAGT
TGGGTGCACGAGTGGGTTACATCGAACTGGATCTCAACAGCGGTAAGATCCTTGAGAGTTTTCGCCCCGAAGAACGTTTTCCAATGATGAGCACTTT
TAAAGTTCTGCTATGTGGCGCGGTATTATCCCGTGTTGACGCCGGGCAAGAGCAACTCGGTCGCCGCATACACTATTCTCAGAATGACTTGGTTGAG
TACTCACCAGTCACAGAAAAGCATCTTACGGATGGCATGACAGTAAGAGAATTATGCAGTGCTGCCATAACCATGAGTGATAACACTGCGGCCAACT
TACTTCTGACAACGATCGGAGGACCGAAGGAGCTAACCGCTTTTTTGCACAACATGGGGGATCATGTAACTCGCCTTGATCGTTGGGAACCGGAGCT
GAATGAAGCCATACCAAACGACGAGCGTGACACCACGATGCCTGTAGCAATGGCAACAACGTTGCGCAAACTATTAACTGGCGAACTACTTACTCTA
GCTTCCCGGCAACAATTAATAGACTGGATGGAGGCGGATAAAGTTGCAGGACCACTTCTGCGCTCGGCCCTTCCGGCTGGCTGGTTTATTGCTGATA
AATCTGGAGCCGGTGAGCGTGGGTCTCGCGGTATCATTGCAGCACTGGGGCCAGATGGTAAGCCCTCCCGTATCGTAGTTATCTACACGACGGGGAG
TCAGGCAACTATGGATGAACGAAATAGACAGATCGCTGAGATAGGTGCCTCACTGATTAAGCATTGGTAACTGTCAGACCAAGTTTACTCATATATA
CTTTAGATTGATTTAAAACTTCATTTTTAATTTAAAAGGATCTAGGTGAAGATCCTTTTTGATAATCTCATGACCAAAATCCCTTAACGTGAGTTTT
CGTTCCACTGAGCGTCAGACCCCGTAGAAAAGATCAAAGGATCTTCTTGAGATCCTTTTTTTCTGCGCGTAATCTGCTGCTTGCAAACAAAAAAACC
ACCGCTACCAGCGGTGGTTTGTTTGCCGGATCAAGAGCTACCAACTCTTTTTCCGAAGGTAACTGGCTTCAGCAGAGCGCAGATACCAAATACTGTC
CTTCTAGTGTAGCCGTAGTTAGGCCACCACTTCAAGAACTCTGTAGCACCGCCTACATACCTCGCTCTGCTAATCCTGTTACCAGTGGCTGCTGCCA
GTGGCGATAAGTCGTGTCTTACCGGGTTGGACTCAAGACGATAGTTACCGGATAAGGCGCAGCGGTCGGGCTGAACGGGGGGTTCGTGCACACAGCC
CAGCTTGGAGCGAACGACCTACACCGAACTGAGATACCTACAGCGTGAGCATTGAGAAAGCGCCACGCTTCCCGAAGGGAGAAAGGCGGACAGGTAT

CCGGTAAGCGGCAGGGTCGGAACAGGAGAGCGCACGAGGGAGCTTCCAGGGGGAAACGCCTGGTATCTTTATAGTCCTGTCGGGTTTCGCCACCTCT
GACTTGAGCGTCGATTTTTGTGATGCTCGTCAGGGGGGCGGAGCCTATGGAAAAACGCCAGCAACGCGAGCTCTAATACGACTCACTATAG

>Ab1C-mCherry
ATGGGCGGCGCATGAGAGAAGCCCAGACCAATTACCTACCCAAAATGGAGAAAGTTCACGTTGACATCGAGGAAGACAGCCCATTCCTCAGAGCTTT
GCAGCGGAGCTTCCCGCAGTTTGAGGTAGAAGCCAAGCAGGTCACTGATAATGACCATGCTAATGCCAGAGCGTTTTCGCATCTGGCTTCAAAACTG
ATCGAAACGGAGGTGGACCCATCCGACACGATCCTTGACATTGGAAGTGCGCCCGCCCGCAGAATGTATTCTAAGCACAAGTATCATTGTATCTGTC
CGATGAGATGTGCGGAAGATCCGGACAGATTGTATAAGTATGCAACTAAGCTGAAGAAAAACTGTAAGGAAATAACTGATAAGGAATTGGACAAGAA
AATGAAGGAGCTGGCCGCCGTCATGAGCGACCCTGACCTGGAAACTGAGACTATGTGCCTCCACGACGACGAGTCGTGTCGCTACGAAGGGCAAGTC
GCTGTTTACCAGGATGTATACGCGGTTGACGGACCGACAAGTCTCTATCACCAAGCCAATAAGGGAGTTAGAGTCGCCTACTGGATAGGCTTTGACA
CCACCCCTTTTATGTTTAAGAACTTGGCTGGAGCATATCCATCATACTCTACCAACTGGGCCGACGAAACCGTGTTAACGGCTCGTAACATAGGCCT
ATGCAGCTCTGACGTTATGGAGCGGTCACGTAGAGGGATGTCCATTCTTAGAAAGAAGTATTTGAAACCATCCAACAATGTTCTATTCTCTGTTGGC
TCGACCATCTACCACGAGAAGAGGGACTTACTGAGGAGCTGGCACCTGCCGTCTGTATTTCACTTACGTGGCAAGCAAAATTACACATGTCGGTGTG
AGACTATAGTTAGTTGCGACGGGTACGTCGTTAAAAGAATAGCTATCAGTCCAGGCCTGTATGGGAAGCCTTCAGGCTATGCTGCTACGATGCACCG
CGAGGGATTCTTGTGCTGCAAAGTGACAGACACATTGAACGGGGAGAGGGTCTCTTTTCCCGTGTGCACGTATGTGCCAGCTACATTGTGTGACCAA
ATGACTGGCATACTGGCAACAGATGTCAGTGCGGACGACGCGCAAAAACTGCTGGTTGGGCTCAACCAGCGTATAGTCGTCAACGGTCGCACCCAGA
GAAACACCAATACCATGAAAAATTACCTTTTGCCCGTAGTGGCCCAGGCATTTGCTAGGTGGGCAAAGGAATATAAGGAAGATCAAGAAGATGAAAG
GCCACTAGGACTACGAGATAGACAGTTAGTCATGGGGTGTTGTTGGGCTTTTAGAAGGCACAAGATAACATCTATTTATAAGCGCCCGGATACCCAA
ACCATCATCAAAGTGAACAGCGATTTCCACTCATTCGTGCTGCCCAGGATAGGCAGTAACACATTGGAGATCGGGCTGAGAACAAGAATCAGGAAAA
TGTTAGAGGAGCACAAGGAGCCGTCACCTCTCATTACCGCCGAGGACGTACAAGAAGCTAAGTGCGCAGCCGATGAGGCTAAGGAGGTGCGTGAAGC
CGAGGAGTTGCGCGCAGCTCTACCACCTTTGGCAGCTGATGTTGAGGAGCCCACTCTGGAGGCAGACGTCGACTTGATGTTACAAGAGGCTGGGGCC
GGCTCAGTGGAGACACCTCGTGGCTTGATAAAGGTTACCAGCTACGATGGCGAGGACAAGATCGGCTCTTACGCTGTGCTTTCTCCGCAGGCTGTAC
TCAAGAGTGAAAAATTATCTTGCATCCACCCTCTCGCTGAACAAGTCATAGTGATAACACACTCTGGCCGAAAAGGGCGTTATGCCGTGGAACCATA
CCATGGTAAAGTAGTGGTGCCAGAGGGACATGCAATACCCGTCCAGGACTTTCAAGCTCTGAGTGAAAGTGCCACCATTGTGTACAACGAACGTGAG
TTCGTAAACAGGTACCTGCACCATATTGCCACACATGGAGGAGCGCTGAACACTGATGAAGAATATTACAAAACTGTCAAGCCCAGCGAGCACGACG
GCGAATACCTGTACGACATCGACAGGAAACAGTGCGTCAAGAAAGAACTAGTCACTGGGCTAGGGCTCACAGGCGAGCTGGTGGATCCTCCCTTCCA
TGAATTCGCCTACGAGAGTCTGAGAACACGACCAGCCGCTCCTTACCAAGTACCAACCATAGGGGTGTATGGCGTGCCAGGATCAGGCAAGTCTGGC
ATCATTAAAAGCGCAGTCACCAAAAAAGATCTAGTGGTGAGCGCCAAGAAAGAAAACTGTGCAGAAATTATAAGGGACGTCAAGAAAATGAAAGGGC
TGGACGTCAATGCCAGAACTGTGGACTCAGTGCTCTTGAATGGATGCAAACACCCCGTAGAGACCCTGTATATTGACGAAGCTTTTGCTTGTCATGC
AGGTACTCTCAGAGCGCTCATAGCCATTATAAGACCTAAAAAGGCAGTGCTCTGCGGGGATCCCAAACAGTGCGGTTTTTTTAACATGATGTGCCTG
AAAGTGCATTTTAACCACGAGATTTGCACACAAGTCTTCCACAAAAGCATCTCTCGCCGTTGCACTAAATCTGTGACTTCGGTCGTCTCAACCTTGT
TTTACGACAAAAAAATGAGAACGACGAATCCGAAAGAGACTAAGATTGTGATTGACACTACCGGCAGTACCAAACCTAAGCAGGACGATCTCATTCT
CACTTGTTTCAGAGGGTGGGTGAAGCAGTTGCAAATAGATTACAAAGGCAACGAAATAATGACGGCAGCTGCCTCTCAAGGGCTGACCCGTAAAGGT
GTGTATGCCGTTCGGTACAAGGTGAATGAAAATCCTCTGTACGCACCCACCTCAGAACATGTGAACGTCCTACTGACCCGCACGGAGGACCGCATCG
TGTGGAAAACACTAGCCGGCGACCCATGGATAAAAACACTGACTGCCAAGTACCCTGGGAATTTCACTGCCACGATAGAGGAGTGGCAAGCAGAGCA
TGATGCCATCATGAGGCACATCTTGGAGAGACCGGACCCTACCGACGTCTTCCAGAATAAGGCAAACGTGTGTTGGGCCAAGGCTTTAGTGCCGGTG
CTGAAGACCGCTGGCATAGACATGACCACTGAACAATGGAACACTGTGGATTATTTTGAAACGGACAAAGCTCACTCAGCAGAGATAGTATTGAACC
AACTATGCGTGAGGTTCTTTGGACTCGATCTGGACTCCGGTCTATTTTCTGCACCCACTGTTCCGTTATCCATTAGGAATAATCACTGGGATAACTC
CCCGTCGCCTAACATGTACGGGCTGAATAAAGAAGTGGTCCGTCAGCTCTCTCGCAGGTACCCACAACTGCCTCGGGCAGTTGCCACTGGAAGAGTC
TATGACATGAACACTGGTACACTGCGCAATTATGATCCGCGCATAAACCTAGTACCTGTAAACAGAAGACTGCCTCATGCTTTAGTCCTCCACCATA
ATGAACACCCACAGAGTGACTTTTCTTCATTCGTCAGCAAATTGAAGGGCAGAACTGTCCTGGTGGTCGGGGAAAAGTTGTCCGTCCCAGGCAAAAT
GGTTGACTGGTTGTCAGACCGGCCTGAGGCTACCTTCAGAGCTCGGCTGGATTTAGGCATCCCAGGTGATGTGCCCAAATATGACATAATATTTGTT
AATGTGAGGACCCCATATAAATACCATCACTATCAGCAGTGTGAAGACCATGCCATTAAGCTTAGCATGTTGACCAAGAAAGCTTGTCTGCATCTGA
ATCCCGGCGGAACCTGTGTCAGCATAGGTTATGGTTACGCTGACAGGGCCAGCGAAAGCATCATTGGTGCTATAGCGCGGCAGTTCAAGTTTTCCCG
GGTATGCAAACCGAAATCCTCACTTGAAGAGACGGAAGTTCTGTTTGTATTCATTCGGTACGATCGCAAGGCCCGTACGCACAATTCTTACAAGCTT
TCATCAACCTTGACCAACATTTATACAGGTTCCAGACTCCACGAAGCCGGATGTGCACCCTCATATCATGTGGTGCGAGGGGATATTGCCACGGCCA
CCGAAGGAGTGATTATAAATGCTGCTAACAGCAAAGGACAACCTGGCGGAGGGGTGTGCGGAGCGCTGTATAAGAAATTCCCGGAAAGCTTCGATTT
ACAGCCGATCGAAGTAGGAAAAGCGCGACTGGTCAAAGGTGCAGCTAAACATATCATTCATGCCGTAGGACCAAACTTCAACAAAGTTTCGGAGGTT
GAAGGTGACAAACAGTTGGCAGAGGCTTATGAGTCCATCGCTAAGATTGTCAACGATAACAATTACAAGTCAGTAGCGATTCCACTGTTGTCCACCG
GCATCTTTTCCGGGAACAAAGATCGACTAACCCAATCATTGAACCATTTGCTGACAGCTTTAGACACCACTGATGCAGATGTAGCCATATACTGCAG
GGACAAGAAATGGGAAATGACTCTCAAGGAAGCAGTGGCTAGGAGAGAAGCAGTGGAGGAGATATGCATATCCGACGACTCTTCAGTGACAGAACCT
GATGCAGAGCTGGTGAGGGTGCATCCGAAGAGTTCTTTGGCTGGAAGGAAGGGCTACAGCACAAGCGATGGCAAAACTTTCTCATATTTGGAAGGGA
CCAAGTTTCACCAGGCGGCCAAGGATATAGCAGAAATTAATGCCATGTGGCCCGTTGCAACGGAGGCCAATGAGCAGGTATGCATGTATATCCTCGG
AGAAAGCATGAGCAGTATTAGGTCGAAATGCCCCGTCGAAGAGTCGGAAGCCTCCACACCACCTAGCACGCTGCCTTGCTTGTGCATCCATGCCATG
ACTCCAGAAAGAGTACAGCGCCTAAAAGCCTCACGTCCAGAACAAATTACTGTGTGCTCATCCTTTCCATTGCCGAAGTATAGAATCACTGGTGTGC
AGAAGATCCAATGCTCCCAGCCTATATTGTTCTCACCGAAAGTGCCTGCGTATATTCATCCAAGGAAGTATCTCGTGGAAACACCACCGGTAGACGA
GACTCCGGAGCCATCGGCAGAGAACCAATCCACAGAGGGGACACCTGAACAACCACCACTTATAACCGAGGATGAGACCAGGACTAGAACGCCTGAG
CCGATCATCATCGAAGAGGAAGAAGAGGATAGCATAAGTTTGCTGTCAGATGGCCCGACCCACCAGGTGCTGCAAGTCGAGGCAGACATTCACGGGC
CGCCCTCTGTATCTAGCTCATCCTGGTCCATTCCTCATGCATCCGACTTTGATGTGGACAGTTTATCCATACTTGACACCCTGGAGGGAGCTAGCGT
GACCAGCGGGGCAACGTCAGCCGAGACTAACTCTTACTTCGCAAAGAGTATGGAGTTTCTGGCGCGACCGGTGCCTGCGCCTCGAACAGTATTCAGG
AACCCTCCACATCCCGCTCCGCGCACAAGAACACCGTCACTTGCACCCAGCAGGGCCTGCTCGAGAACCAGCCTAGTTTCCACCCCGCCAGGCGTGA
ATAGGGTGATCACTAGAGAGGAGCTCGAGGCGCTTACCCCGTCACGCACTCCTAGCAGGTCGGTCTCGAGAACCAGCCTGGTCTCCAACCCGCCAGG
CGTAAATAGGGTGATTACAAGAGAGGAGTTTGAGGCGTTCGTAGCACAACAACAATGACGGTTTGATGCGGGTGCATACATCTTTTCCTCCGACACC
GGTCAAGGGCATTTACAACAAAAATCAGTAAGGCAAACGGTGCTATCCGAAGTGGTGTTGGAGAGGACCGAATTGGAGATTTCGTATGCCCCGCGCC
TCGACCAAGAAAAAGAAGAATTACTACGCAAGAAATTACAGTTAAATCCCACACCTGCTAACAGAAGCAGATACCAGTCCAGGAAGGTGGAGAACAT
GAAAGCCATAACAGCTAGACGTATTCTGCAAGGCCTAGGGCATTATTTGAAGGCAGAAGGAAAAGTGGAGTGCTACCGAACCCTGCATCCTGTTCCT
TTGTATTCATCTAGTGTGAACCGTGCCTTTTCAAGCCCCAAGGTCGCAGTGGAAGCCTGTAACGCCATGTTGAAAGAGAACTTTCCGACTGTGGCTT
CTTACTGTATTATTCCAGAGTACGATGCCTATTTGGACATGGTTGACGGAGCTTCATGCTGCTTAGACACTGCCAGTTTTTGCCCTGCAAAGCTGCG
CAGCTTTCCAAAGAAACACTCCTATTTGGAACCCACAATACGATCGGCAGTGCCTTCAGCGATCCAGAACACGCTCCAGAACGTCCTGGCAGCTGCC
ACAAAAAGAAATTGCAATGTCACGCAAATGAGAGAATTGCCCGTATTGGATTCGGCGGCCTTTAATGTGGAATGCTTCAAGAAATATGCGTGTAATA
ATGAATATTGGGAAACGTTTAAAGAAAACCCCATCAGGCTTACTGAAGAAAACGTGGTAAATTACATTACCAAATTAAAAGGACCAAAAGCTGCTGC
TCTTTTTGCGAAGACACATAATTTGAATATGTTGCAGGACATACCAATGGACAGGTTTGTAATGGACTTAAAGAGAGACGTGAAAGTGACTCCAGGA

ACAAAACATACTGAAGAACGGCCCAAGGTACAGGTGATCCAGGCTGCCGATCCGCTAGCAACAGCGTATCTGTGCGGAATCCACCGAGAGCTGGTTA
GGAGATTAAATGCGGTCCTGCTTCCGAACATTCATACACTGTTTGATATGTCGGCTGAAGACTTTGACGCTATTATAGCCGAGCACTTCCAGCCTGG
GGATTGTGTTCTGGAAACTGACATCGCGTCGTTTGATAAAAGTGAGGACGACGCCATGGCTCTGACCGCGTTAATGATTCTGGAAGACTTAGGTGTG
GACGCAGAGCTGTTGACGCTGATTGAGGCGGCTTTCGGCGAAATTTCATCAATACATTTGCCCACTAAAACTAAATTTAAATTCGGAGCCATGATGA
AATCTGGAATGTTCCTCACACTGTTTGTGAACACAGTCATTAACATTGTAATCGCAAGCAGAGTGTTGAGAGAACGGCTAACCGGATCACCATGTGC
AGCATTCATTGGAGATGACAATATCGTGAAAGGAGTCAAATCGGACAAATTAATGGCAGACAGGTGCGCCACCTGGTTGAATATGGAAGTCAAGATT
ATAGATGCTGTGGTGGGCGAGAAAGCGCCTTATTTCTGTGGAGGGTTTATTTTGTGTGACTCCGTGACCGGCACAGCGTGCCGTGTGGCAGACCCCC
TAAAAAGGCTGTTTAAGCTTGGCAAACCTCTGGCAGCAGACGATGAACATGATGATGACAGGAGAAGGGCATTGCATGAAGAGTCAACACGCTGGAA
CCGAGTGGGTATTCTTTCAGAGCTGTGCAAGGCAGTAGAATCAAGGTATGAAACCGTAGGAACTTCCATCATAGTTATGGCCATGACTACTCTAGCT
AGCAGTGTTAAATCATTCAGCTACCTGAGAGGGGCCCCTATAACTCTCTACGGCTAACCTGAATGGACTACGACATAGTCTAGTCCGCCAAGTCTAG
catatgggcgcgccctcagcatcgattgaattggccaccATGGTGAGCAAGGGCGAGGAGGATAACATGGCCATCATCAAGGAGTTCATGCGCTTCA
AGGTGCACATGGAGGGCTCCGTGAACGGCCACGAGTTCGAGATCGAGGGCGAGGGCGAGGGCCGCCCCTACGAGGGCACCCAGACCGCCAAGCTGAA
GGTGACCAAGGGTGGCCCCCTGCCCTTCGCCTGGGACATCCTGTCCCCTCAGTTCATGTACGGCTCCAAGGCCTACGTGAAGCACCCCGCCGACATC
CCCGACTACTTGAAGCTGTCCTTCCCCGAGGGCTTCAAGTGGGAGCGCGTGATGAACTTCGAGGACGGCGGCGTGGTGACCGTGACCCAGGACTCCT
CCCTGCAGGACGGCGAGTTCATCTACAAGGTGAAGCTGCGCGGCACCAACTTCCCCTCCGACGGCCCCGTAATGCAGAAGAAGACCATGGGCTGGGA
GGCCTCCTCCGAGCGGATGTACCCCGAGGACGGCGCCCTGAAGGGCGAGATCAAGCAGAGGCTGAAGCTGAAGGACGGCGGCCACTACGACGCTGAG
GTCAAGACCACCTACAAGGCCAAGAAGCCCGTGCAGCTGCCCGGCGCCTACAACGTCAACATCAAGTTGGACATCACCTCCCACAACGAGGACTACA
CCATCGTGGAACAGTACGAACGCGCCGAGGGCCGCCACTCCACCGGCGGCATGGACGAGCTGTACAAGTAGgaattGGCAAGCTGCTTACATAGAAC
TCGCGGCGATTGGCATGCCGCCTTAAAATTTTTATTTTATTTTTCTTTTCTTTTCCGAATCGGATTTTGTTTTTAATATTTCAAAAAAAAAAAAAAA
AAAAAAAAAAACGCGTCGAGGGGAATTAATTCTTGAAGACGAAAGGGCCAGGTGGCACTTTTCGGGGAAATGTGCGCGGAACCCCTATTTGTTTATT
TTTCTAAATACATTCAAATATGTATCCGCTCATGAGACAATAACCCTGATAAATGCTTCAATAATATTGAAAAAGGAAGAGTATGAGTATTCAACAT
TTCCGTGTCGCCCTTATTCCCTTTTTTGCGGCATTTTGCCTTCCTGTTTTTGCTCACCCAGAAACGCTGGTGAAAGTAAAAGATGCTGAAGATCAGT
TGGGTGCACGAGTGGGTTACATCGAACTGGATCTCAACAGCGGTAAGATCCTTGAGAGTTTTCGCCCCGAAGAACGTTTTCCAATGATGAGCACTTT
TAAAGTTCTGCTATGTGGCGCGGTATTATCCCGTGTTGACGCCGGGCAAGAGCAACTCGGTCGCCGCATACACTATTCTCAGAATGACTTGGTTGAG
TACTCACCAGTCACAGAAAAGCATCTTACGGATGGCATGACAGTAAGAGAATTATGCAGTGCTGCCATAACCATGAGTGATAACACTGCGGCCAACT
TACTTCTGACAACGATCGGAGGACCGAAGGAGCTAACCGCTTTTTTGCACAACATGGGGGATCATGTAACTCGCCTTGATCGTTGGGAACCGGAGCT
GAATGAAGCCATACCAAACGACGAGCGTGACACCACGATGCCTGTAGCAATGGCAACAACGTTGCGCAAACTATTAACTGGCGAACTACTTACTCTA
GCTTCCCGGCAACAATTAATAGACTGGATGGAGGCGGATAAAGTTGCAGGACCACTTCTGCGCTCGGCCCTTCCGGCTGGCTGGTTTATTGCTGATA
AATCTGGAGCCGGTGAGCGTGGGTCTCGCGGTATCATTGCAGCACTGGGGCCAGATGGTAAGCCCTCCCGTATCGTAGTTATCTACACGACGGGGAG
TCAGGCAACTATGGATGAACGAAATAGACAGATCGCTGAGATAGGTGCCTCACTGATTAAGCATTGGTAACTGTCAGACCAAGTTTACTCATATATA
CTTTAGATTGATTTAAAACTTCATTTTTAATTTAAAAGGATCTAGGTGAAGATCCTTTTTGATAATCTCATGACCAAAATCCCTTAACGTGAGTTTT
CGTTCCACTGAGCGTCAGACCCCGTAGAAAAGATCAAAGGATCTTCTTGAGATCCTTTTTTTCTGCGCGTAATCTGCTGCTTGCAAACAAAAAAACC
ACCGCTACCAGCGGTGGTTTGTTTGCCGGATCAAGAGCTACCAACTCTTTTTCCGAAGGTAACTGGCTTCAGCAGAGCGCAGATACCAAATACTGTC
CTTCTAGTGTAGCCGTAGTTAGGCCACCACTTCAAGAACTCTGTAGCACCGCCTACATACCTCGCTCTGCTAATCCTGTTACCAGTGGCTGCTGCCA
GTGGCGATAAGTCGTGTCTTACCGGGTTGGACTCAAGACGATAGTTACCGGATAAGGCGCAGCGGTCGGGCTGAACGGGGGGTTCGTGCACACAGCC
CAGCTTGGAGCGAACGACCTACACCGAACTGAGATACCTACAGCGTGAGCATTGAGAAAGCGCCACGCTTCCCGAAGGGAGAAAGGCGGACAGGTAT
CCGGTAAGCGGCAGGGTCGGAACAGGAGAGCGCACGAGGGAGCTTCCAGGGGGAAACGCCTGGTATCTTTATAGTCCTGTCGGGTTTCGCCACCTCT
GACTTGAGCGTCGATTTTTGTGATGCTCGTCAGGGGGGCGGAGCCTATGGAAAAACGCCAGCAACGCGAGCTCTAATACGACTCACTATAG

>ab1C-mCherry
ATGGGCGGCGCATGAGAGAAGCCCAGACCAATTACCTACCCAAAATGGAGAAAGTTCACGTTGACATCGAGGAAGACAGCCCATTCCTCAGAGCTTT
GCAGCGGAGCTTCCCGCAGTTTGAGGTAGAAGCCAAGCAGGTCACTGATAATGACCATGCTAATGCCAGAGCGTTTTCGCATCTGGCTTCAAAACTG
ATCGAAACGGAGGTGGACCCATCCGACACGATCCTTGACATTGGAAGTGCGCCCGCCCGCAGAATGTATTCTAAGCACAAGTATCATTGTATCTGTC
CGATGAGATGTGCGGAAGATCCGGACAGATTGTATAAGTATGCAACTAAGCTGAAGAAAAACTGTAAGGAAATAACTGATAAGGAATTGGACAAGAA
AATGAAGGAGCTGGCCGCCGTCATGAGCGACCCTGACCTGGAAACTGAGACTATGTGCCTCCACGACGACGAGTCGTGTCGCTACGAAGGGCAAGTC
GCTGTTTACCAGGATGTATACGCGGTTGACGGACCGACAAGTCTCTATCACCAAGCCAATAAGGGAGTTAGAGTCGCCTACTGGATAGGCTTTGACA
CCACCCCTTTTATGTTTAAGAACTTGGCTGGAGCATATCCATCATACTCTACCAACTGGGCCGACGAAACCGTGTTAACGGCTCGTAACATAGGCCT
ATGCAGCTCTGACGTTATGGAGCGGTCACGTAGAGGGATGTCCATTCTTAGAAAGAAGTATTTGAAACCATCCAACAATGTTCTATTCTCTGTTGGC
TCGACCATCTACCACGAGAAGAGGGACTTACTGAGGAGCTGGCACCTGCCGTCTGTATTTCACTTACGTGGCAAGCAAAATTACACATGTCGGTGTG
AGACTATAGTTAGTTGCGACGGGTACGTCGTTAAAAGAATAGCTATCAGTCCAGGCCTGTATGGGAAGCCTTCAGGCTATGCTGCTACGATGCACCG
CGAGGGATTCTTGTGCTGCAAAGTGACAGACACATTGAACGGGGAGAGGGTCTCTTTTCCCGTGTGCACGTATGTGCCAGCTACATTGTGTGACCAA
ATGACTGGCATACTGGCAACAGATGTCAGTGCGGACGACGCGCAAAAACTGCTGGTTGGGCTCAACCAGCGTATAGTCGTCAACGGTCGCACCCAGA
GAAACACCAATACCATGAAAAATTACCTTTTGCCCGTAGTGGCCCAGGCATTTGCTAGGTGGGCAAAGGAATATAAGGAAGATCAAGAAGATGAAAG
GCCACTAGGACTACGAGATAGACAGTTAGTCATGGGGTGTTGTTGGGCTTTTAGAAGGCACAAGATAACATCTATTTATAAGCGCCCGGATACCCAA
ACCATCATCAAAGTGAACAGCGATTTCCACTCATTCGTGCTGCCCAGGATAGGCAGTAACACATTGGAGATCGGGCTGAGAACAAGAATCAGGAAAA
TGTTAGAGGAGCACAAGGAGCCGTCACCTCTCATTACCGCCGAGGACGTACAAGAAGCTAAGTGCGCAGCCGATGAGGCTAAGGAGGTGCGTGAAGC
CGAGGAGTTGCGCGCAGCTCTACCACCTTTGGCAGCTGATGTTGAGGAGCCCACTCTGGAGGCAGACGTCGACTTGATGTTACAAGAGGCTGGGGCC
GGCTCAGTGGAGACACCTCGTGGCTTGATAAAGGTTACCAGCTACGATGGCGAGGACAAGATCGGCTCTTACGCTGTGCTTTCTCCGCAGGCTGTAC
TCAAGAGTGAAAAATTATCTTGCATCCACCCTCTCGCTGAACAAGTCATAGTGATAACACACTCTGGCCGAAAAGGGCGTTATGCCGTGGAACCATA
CCATGGTAAAGTAGTGGTGCCAGAGGGACATGCAATACCCGTCCAGGACTTTCAAGCTCTGAGTGAAAGTGCCACCATTGTGTACAACGAACGTGAG
TTCGTAAACAGGTACCTGCACCATATTGCCACACATGGGGGAGCGCTGAACACTGATGAAGAATATTACAAAACTGTCAAGCCCAGCGAGCACGACG
GCGAATACCTGTACGACATCGACAGGAAACAGTGCGTCAAGAAAGAACTAGTCACTGGGCTAGGGCTCACAGGCGAGCTGGTGGATCCTCCCTTCCA
TGAATTCGCCTACGAGAGTCTGAGAACACGACCAGCCGCTCCTTACCAAGTACCAACCATAGGGGTGTATGGCGTGCCAGGATCAGGCAAGTCTGGC
ATCATTAAAAGCGCAGTCACCAAAAAAGATCTAGTGGTGAGCGCCAAGAAAGAAAACTGTGCAGAAATTATAAGGGACGTCAAGAAAATGAAAGGGC
TGGACGTCAATGCCAGAACTGTGGACTCAGTGCTCTTGAATGGATGCAAACACCCCGTAGAGACCCTGTATATTGACGAAGCTTTTGCTTGTCATGC
AGGTACTCTCAGAGCGCTCATAGCCATTATAAGACCTAAAAAGGCAGTGCTCTGCGGGGATCCCAAACAGTGCGGTTTTTTTAACATGATGTGCCTG
AAAGTGCATTTTAACCACGAGATTTGCACACAAGTCTTCCACAAAAGCATCTCTCGCCGTTGCACTAAATCTGTGACTTCGGTCGTCTCAACCTTGT
TTTACGACAAAAAAATGAGAACGACGAATCCGAAAGAGACTAAGATTGTGATTGACACTACCGGCAGTACCAAACCTAAGCAGGACGATCTCATTCT
CACTTGTTTCAGAGGGTGGGTGAAGCAGTTGCAAATAGATTACAAAGGCAACGAAATAATGACGGCAGCTGCCTCTCAAGGGCTGACCCGTAAAGGT
GTGTATGCCGTTCGGTACAAGGTGAATGAAAATCCTCTGTACGCACCCACCTCAGAACATGTGAACGTCCTACTGACCCGCACGGAGGACCGCATCG
TGTGGAAAACACTAGCCGGCGACCCATGGATAAAAACACTGACTGCCAAGTACCCTGGGAATTTCACTGCCACGATAGAGGAGTGGCAAGCAGAGCA
TGATGCCATCATGAGGCACATCTTGGAGAGACCGGACCCTACCGACGTCTTCCAGAATAAGGCAAACGTGTGTTGGGCCAAGGCTTTAGTGCCGGTG

CTGAAGACCGCTGGCATAGACATGACCACTGAACAATGGAACACTGTGGATTATTTTGAAACGGACAAAGCTCACTCAGCAGAGATAGTATTGAACC
AACTATGCGTGAGGTTCTTTGGACTCGATCTGGACTCCGGTCTATTTTCTGCACCCACTGTTCCGTTATCCATTAGGAATAATCACTGGGATAACTC
CCCGTCGCCTAACATGTACGGGCTGAATAAAGAAGTGGTCCGTCAGCTCTCTCGCAGGTACCCACAACTGCCTCGGGCAGTTGCCACTGGAAGAGTC
TATGACATGAACACTGGTACACTGCGCAATTATGATCCGCGCATAAACCTAGTACCTGTAAACAGAAGACTGCCTCATGCTTTAGTCCTCCACCATA
ATGAACACCCACAGAGTGACTTTTCTTCATTCGTCAGCAAATTGAAGGGCAGAACTGTCCTGGTGGTCGGGGAAAAGTTGTCCGTCCCAGGCAAAAT
GGTTGACTGGTTGTCAGACCGGCCTGAGGCTACCTTCAGAGCTCGGCTGGATTTAGGCATCCCAGGTGATGTGCCCAAATATGACATAATATTTGTT
AATGTGAGGACCCCATATAAATACCATCACTATCAGCAGTGTGAAGACCATGCCATTAAGCTTAGCATGTTGACCAAGAAAGCTTGTCTGCATCTGA
ATCCCGGCGGAACCTGTGTCAGCATAGGTTATGGTTACGCTGACAGGGCCAGCGAAAGCATCATTGGTGCTATAGCGCGGCAGTTCAAGTTTTCCCG
GGTATGCAAACCGAAATCCTCACTTGAAGAGACGGAAGTTCTGTTTGTATTCATTCGGTACGATCGCAAGGCCCGTACGCACAATTCTTACAAGCTT
TCATCAACCTTGACCAACATTTATACAGGTTCCAGACTCCACGAAGCCGGATGTGCACCCTCATATCATGTGGTGCGAGGGGATATTGCCACGGCCA
CCGAAGGAGTGATTATAAATGCTGCTAACAGCAAAGGACAACCTGGCGGAGGGGTGTGCGGAGCGCTGTATAAGAAATTCCCGGAAAGCTTCGATTT
ACAGCCGATCGAAGTAGGAAAAGCGCGACTGGTCAAAGGTGCAGCTAAACATATCATTCATGCCGTAGGACCAAACTTCAACAAAGTTTCGGAGGTT
GAAGGTGACAAACAGTTGGCAGAGGCTTATGAGTCCATCGCTAAGATTGTCAACGATAACAATTACAAGTCAGTAGCGATTCCACTGTTGTCCACCG
GCATCTTTTCCGGGAACAAAGATCGACTAACCCAATCATTGAACCATTTGCTGACAGCTTTAGACACCACTGATGCAGATGTAGCCATATACTGCAG
GGACAAGAAATGGGAAATGACTCTCAAGGAAGCAGTGGCTAGGAGAGAAGCAGTGGAGGAGATATGCATATCCGACGACTCTTCAGTGACAGAACCT
GATGCAGAGCTGGTGAGGGTGCATCCGAAGAGTTCTTTGGCTGGAAGGAAGGGCTACAGCACAAGCGATGGCAAAACTTTCTCATATTTGGAAGGGA
CCAAGTTTCACCAGGCGGCCAAGGATATAGCAGAAATTAATGCCATGTGGCCCGTTGCAACGGAGGCCAATGAGCAGGTATGCATGTATATCCTCGG
AGAAAGCATGAGCAGTATTAGGTCGAAATGCCCCGTCGAAGAGTCGGAAGCCTCCACACCACCTAGCACGCTGCCTTGCTTGTGCATCCATGCCATG
ACTCCAGAAAGAGTACAGCGCCTAAAAGCCTCACGTCCAGAACAAATTACTGTGTGCTCATCCTTTCCATTGCCGAAGTATAGAATCACTGGTGTGC
AGAAGATCCAATGCTCCCAGCCTATATTGTTCTCACCGAAAGTGCCTGCGTATATTCATCCAAGGAAGTATCTCGTGGAAACACCACCGGTAGACGA
GACTCCGGAGCCATCGGCAGAGAACCAATCCACAGAGGGGACACCTGAACAACCACCACTTATAACCGAGGATGAGACCAGGACTAGAACGCCTGAG
CCGATCATCATCGAAGAGGAAGAAGAGGATAGCATAAGTTTGCTGTCAGATGGCCCGACCCACCAGGTGCTGCAAGTCGAGGCAGACATTCACGGGC
CGCCCTCTGTATCTAGCTCATCCTGGTCCATTCCTCATGCATCCGACTTTGATGTGGACAGTTTATCCATACTTGACACCCTGGAGGGAGCTAGCGT
GACCAGCGGGGCAACGTCAGCCGAGACTAACTCTTACTTCGCAAAGAGTATGGAGTTTCTGGCGCGACCGGTGCCTGCGCCTCGAACAGTATTCAGG
AACCCTCCACATCCCGCTCCGCGCACAAGAACACCGTCACTTGCACCCAGCAGGGCCTGCTCGAGAACCAGCCTAGTTTCCACCCCGCCAGGCGTGA
ATAGGGTGATCACTAGAGAGGAGCTCGAGGCGCTTACCCCGTCACGCACTCCTAGCAGGTCGGTCTCGAGAACCAGCCTGGTCTCCAACCCGCCAGG
CGTAAATAGGGTGATTACAAGAGAGGAGTTTGAGGCGTTCGTAGCACAACAACAATGACGGTTTGATGCGGGTGCATACATCTTTTCCTCCGACACC
GGTCAAGGGCATTTACAACAAAAATCAGTAAGGCAAACGGTGCTATCCGAAGTGGTGTTGGAGAGGACCGAATTGGAGATTTCGTATGCCCCGCGCC
TCGACCAAGAAAAAGAAGAATTACTACGCAAGAAATTACAGTTAAATCCCACACCTGCTAACAGAAGCAGATACCAGTCCAGGAAGGTGGAGAACAT
GAAAGCCATAACAGCTAGACGTATTCTGCAAGGCCTAGGGCATTATTTGAAGGCAGAAGGAAAAGTGGAGTGCTACCGAACCCTGCATCCTGTTCCT
TTGTATTCATCTAGTGTGAACCGTGCCTTTTCAAGCCCCAAGGTCGCAGTGGAAGCCTGTAACGCCATGTTGAAAGAGAACTTTCCGACTGTGGCTT
CTTACTGTATTATTCCAGAGTACGATGCCTATTTGGACATGGTTGACGGAGCTTCATGCTGCTTAGACACTGCCAGTTTTTGCCCTGCAAAGCTGCG
CAGCTTTCCAAAGAAACACTCCTATTTGGAACCCACAATACGATCGGCAGTGCCTTCAGCGATCCAGAACACGCTCCAGAACGTCCTGGCAGCTGCC
ACAAAAAGAAATTGCAATGTCACGCAAATGAGAGAATTGCCCGTATTGGATTCGGCGGCCTTTAATGTGGAATGCTTCAAGAAATATGCGTGTAATA
ATGAATATTGGGAAACGTTTAAAGAAAACCCCATCAGGCTTACTGAAGAAAACGTGGTAAATTACATTACCAAATTAAAAGGACCAAAAGCTGCTGC
TCTTTTTGCGAAGACACATAATTTGAATATGTTGCAGGACATACCAATGGACAGGTTTGTAATGGACTTAAAGAGAGACGTGAAAGTGACTCCAGGA
ACAAAACATACTGAAGAACGGCCCAAGGTACAGGTGATCCAGGCTGCCGATCCGCTAGCAACAGCGTATCTGTGCGGAATCCACCGAGAGCTGGTTA
GGAGATTAAATGCGGTCCTGCTTCCGAACATTCATACACTGTTTGATATGTCGGCTGAAGACTTTGACGCTATTATAGCCGAGCACTTCCAGCCTGG
GGATTGTGTTCTGGAAACTGACATCGCGTCGTTTGATAAAAGTGAGGACGACGCCATGGCTCTGACCGCGTTAATGATTCTGGAAGACTTAGGTGTG
GACGCAGAGCTGTTGACGCTGATTGAGGCGGCTTTCGGCGAAATTTCATCAATACATTTGCCCACTAAAACTAAATTTAAATTCGGAGCCATGATGA
AATCTGGAATGTTCCTCACACTGTTTGTGAACACAGTCATTAACATTGTAATCGCAAGCAGAGTGTTGAGAGAACGGCTAACCGGATCACCATGTGC
AGCATTCATTGGAGATGACAATATCGTGAAAGGAGTCAAATCGGACAAATTAATGGCAGACAGGTGCGCCACCTGGTTGAATATGGAAGTCAAGATT
ATAGATGCTGTGGTGGGCGAGAAAGCGCCTTATTTCTGTGGAGGGTTTATTTTGTGTGACTCCGTGACCGGCACAGCGTGCCGTGTGGCAGACCCCC
TAAAAAGGCTGTTTAAGCTTGGCAAACCTCTGGCAGCAGACGATGAACATGATGATGACAGGAGAAGGGCATTGCATGAAGAGTCAACACGCTGGAA
CCGAGTGGGTATTCTTTCAGAGCTGTGCAAGGCAGTAGAATCAAGGTATGAAACCGTAGGAACTTCCATCATAGTTATGGCCATGACTACTCTAGCT
AGCAGTGTTAAATCATTCAGCTACCTGAGAGGGGCCCCTATAACTCTCTACGGCTAACCTGAATGGACTACGACATAGTCTAGTCCGCCAAGTCTAG
catatgggcgcgccctcagcatcgattgaattggccaccATGGTGAGCAAGGGCGAGGAGGATAACATGGCCATCATCAAGGAGTTCATGCGCTTCA
AGGTGCACATGGAGGGCTCCGTGAACGGCCACGAGTTCGAGATCGAGGGCGAGGGCGAGGGCCGCCCCTACGAGGGCACCCAGACCGCCAAGCTGAA
GGTGACCAAGGGTGGCCCCCTGCCCTTCGCCTGGGACATCCTGTCCCCTCAGTTCATGTACGGCTCCAAGGCCTACGTGAAGCACCCCGCCGACATC
CCCGACTACTTGAAGCTGTCCTTCCCCGAGGGCTTCAAGTGGGAGCGCGTGATGAACTTCGAGGACGGCGGCGTGGTGACCGTGACCCAGGACTCCT
CCCTGCAGGACGGCGAGTTCATCTACAAGGTGAAGCTGCGCGGCACCAACTTCCCCTCCGACGGCCCCGTAATGCAGAAGAAGACCATGGGCTGGGA
GGCCTCCTCCGAGCGGATGTACCCCGAGGACGGCGCCCTGAAGGGCGAGATCAAGCAGAGGCTGAAGCTGAAGGACGGCGGCCACTACGACGCTGAG
GTCAAGACCACCTACAAGGCCAAGAAGCCCGTGCAGCTGCCCGGCGCCTACAACGTCAACATCAAGTTGGACATCACCTCCCACAACGAGGACTACA
CCATCGTGGAACAGTACGAACGCGCCGAGGGCCGCCACTCCACCGGCGGCATGGACGAGCTGTACAAGTAGgaattGGCAAGCTGCTTACATAGAAC
TCGCGGCGATTGGCATGCCGCCTTAAAATTTTTATTTTATTTTTCTTTTCTTTTCCGAATCGGATTTTGTTTTTAATATTTCAAAAAAAAAAAAAAA
AAAAAAAAAAACGCGTCGAGGGGAATTAATTCTTGAAGACGAAAGGGCCAGGTGGCACTTTTCGGGGAAATGTGCGCGGAACCCCTATTTGTTTATT
TTTCTAAATACATTCAAATATGTATCCGCTCATGAGACAATAACCCTGATAAATGCTTCAATAATATTGAAAAAGGAAGAGTATGAGTATTCAACAT
TTCCGTGTCGCCCTTATTCCCTTTTTTGCGGCATTTTGCCTTCCTGTTTTTGCTCACCCAGAAACGCTGGTGAAAGTAAAAGATGCTGAAGATCAGT
TGGGTGCACGAGTGGGTTACATCGAACTGGATCTCAACAGCGGTAAGATCCTTGAGAGTTTTCGCCCCGAAGAACGTTTTCCAATGATGAGCACTTT
TAAAGTTCTGCTATGTGGCGCGGTATTATCCCGTGTTGACGCCGGGCAAGAGCAACTCGGTCGCCGCATACACTATTCTCAGAATGACTTGGTTGAG
TACTCACCAGTCACAGAAAAGCATCTTACGGATGGCATGACAGTAAGAGAATTATGCAGTGCTGCCATAACCATGAGTGATAACACTGCGGCCAACT
TACTTCTGACAACGATCGGAGGACCGAAGGAGCTAACCGCTTTTTTGCACAACATGGGGGATCATGTAACTCGCCTTGATCGTTGGGAACCGGAGCT
GAATGAAGCCATACCAAACGACGAGCGTGACACCACGATGCCTGTAGCAATGGCAACAACGTTGCGCAAACTATTAACTGGCGAACTACTTACTCTA
GCTTCCCGGCAACAATTAATAGACTGGATGGAGGCGGATAAAGTTGCAGGACCACTTCTGCGCTCGGCCCTTCCGGCTGGCTGGTTTATTGCTGATA
AATCTGGAGCCGGTGAGCGTGGGTCTCGCGGTATCATTGCAGCACTGGGGCCAGATGGTAAGCCCTCCCGTATCGTAGTTATCTACACGACGGGGAG
TCAGGCAACTATGGATGAACGAAATAGACAGATCGCTGAGATAGGTGCCTCACTGATTAAGCATTGGTAACTGTCAGACCAAGTTTACTCATATATA
CTTTAGATTGATTTAAAACTTCATTTTTAATTTAAAAGGATCTAGGTGAAGATCCTTTTTGATAATCTCATGACCAAAATCCCTTAACGTGAGTTTT
CGTTCCACTGAGCGTCAGACCCCGTAGAAAAGATCAAAGGATCTTCTTGAGATCCTTTTTTTCTGCGCGTAATCTGCTGCTTGCAAACAAAAAAACC
ACCGCTACCAGCGGTGGTTTGTTTGCCGGATCAAGAGCTACCAACTCTTTTTCCGAAGGTAACTGGCTTCAGCAGAGCGCAGATACCAAATACTGTC
CTTCTAGTGTAGCCGTAGTTAGGCCACCACTTCAAGAACTCTGTAGCACCGCCTACATACCTCGCTCTGCTAATCCTGTTACCAGTGGCTGCTGCCA
GTGGCGATAAGTCGTGTCTTACCGGGTTGGACTCAAGACGATAGTTACCGGATAAGGCGCAGCGGTCGGGCTGAACGGGGGGTTCGTGCACACAGCC
CAGCTTGGAGCGAACGACCTACACCGAACTGAGATACCTACAGCGTGAGCATTGAGAAAGCGCCACGCTTCCCGAAGGGAGAAAGGCGGACAGGTAT

CCGGTAAGCGGCAGGGTCGGAACAGGAGAGCGCACGAGGGAGCTTCCAGGGGGAAACGCCTGGTATCTTTATAGTCCTGTCGGGTTTCGCCACCTCT
GACTTGAGCGTCGATTTTTGTGATGCTCGTCAGGGGGGCGGAGCCTATGGAAAAACGCCAGCAACGCGAGCTCTAATACGACTCACTATAG

>Ab2C-mCherry
ATGGGCGGCGCATGAGAGAAGCCCAGACCAATTACCTACCCAAAATGGAGAAAGTTCACGTTGACATCGAGGAAGACAGCCCATTCCTCAGAGCTTT
GCAGCGGAGCTTCCCGCAGTTTGAGGTAGAAGCCAAGCAGGTCACTGATAATGACCATGCTAATGCCAGAGCGTTTTCGCATCTGGCTTCAAAACTG
ATCGAAACGGAGGTGGACCCATCCGACACGATCCTTGACATTGGAAGTGCGCCCGCCCGCAGAATGTATTCTAAGCACAAGTATCATTGTATCTGTC
CGATGAGATGTGCGGAAGATCCGGACAGATTGTATAAGTATGCAACTAAGCTGAAGAAAAACTGTAAGGAAATAACTGATAAGGAATTGGACAAGAA
AATGAAGGAGCTGGCCGCCGTCATGAGCGACCCTGACCTGGAAACTGAGACTATGTGCCTCCACGACGACGAGTCGTGTCGCTACGAAGGGCAAGTC
GCTGTTTACCAGGATGTATACGCGGTTGACGGACCGACAAGTCTCTATCACCAAGCCAATAAGGGAGTTAGAGTCGCCTACTGGATAGGCTTTGACA
CCACCCCTTTTATGTTTAAGAACTTGGCTGGAGCATATCCATCATACTCTACCAACTGGGCCGACGAAACCGTGTTAACGGCTCGTAACATAGGCCT
ATGCAGCTCTGACGTTATGGAGCGGTCACGTAGAGGGATGTCCATTCTTAGAAAGAAGTATTTGAAACCATCCAACAATGTTCTATTCTCTGTTGGC
TCGACCATCTACCACGAGAAGAGGGACTTACTGAGGAGCTGGCACCTGCCGTCTGTATTTCACTTACGTGGCAAGCAAAATTACACATGTCGGTGTG
AGACTATAGTTAGTTGCGACGGGTACGTCGTTAAAAGAATAGCTATCAGTCCAGGCCTGTATGGGAAGCCTTCAGGCTATGCTGCTACGATGCACCG
CGAGGGATTCTTGTGCTGCAAAGTGACAGACACATTGAACGGGGAGAGGGTCTCTTTTCCCGTGTGCACGTATGTGCCAGCTACATTGTGTGACCAA
ATGACTGGCATACTGGCAACAGATGTCAGTGCGGACGACGCGCAAAAACTGCTGGTTGGGCTCAACCAGCGTATAGTCGTCAACGGTCGCACCCAGA
GAAACACCAATACCATGAAAAATTACCTTTTGCCCGTAGTGGCCCAGGCATTTGCTAGGTGGGCAAAGGAATATAAGGAAGATCAAGAAGATGAAAG
GCCACTAGGACTACGAGATAGACAGTTAGTCATGGGGTGTTGTTGGGCTTTTAGAAGGCACAAGATAACATCTATTTATAAGCGCCCGGATACCCAA
ACCATCATCAAAGTGAACAGCGATTTCCACTCATTCGTGCTGCCCAGGATAGGCAGTAACACATTGGAGATCGGGCTGAGAACAAGAATCAGGAAAA
TGTTAGAGGAGCACAAGGAGCCGTCACCTCTCATTACCGCCGAGGACGTACAAGAAGCTAAGTGCGCAGCCGATGAGGCTAAGGAGGTGCGTGAAGC
CGAGGAGTTGCGCGCAGCTCTACCACCTTTGGCAGCTGATGTTGAGGAGCCCACTCTGGAGGCAGACGTCGACTTGATGTTACAAGAGGCTGGGGCC
GGCTCAGTGGAGACACCTCGTGGCTTGATAAAGGTTACCAGCTACGATGGCGAGGACAAGATCGGCTCTTACGCTGTGCTTTCTCCGCAGGCTGTAC
TCAAGAGTGAAAAATTATCTTGCATCCACCCTCTCGCTGAACAAGTCATAGTGATAACACACTCTGGCCGAAAAGGGCGTTATGCCGTGGAACCATA
CCATGGTAAAGTAGTGGTGCCAGAGGGACATGCAATACCCGTCCAGGACTTTCAAGCTCTGAGTGAAAGTGCCACCATTGTGTACAACGAACGTGAG
TTCGTAAACAGGTACCTGCACCATATTGCCACACATGGAGGAGCGCTGAACACTGATGAAGAATATTACAAAACTGTCAAGCCCAGCGAGCACGACG
GCGAATACCTGTACGACATCGACAGGAAACAGTGCGTCAAGAAAGAACTAGTCACTGGGCTAGGGCTCACAGGCGAGCTGGTGGATCCTCCCTTCCA
TGAATTCGCCTACGAGAGTCTGAGAACACGACCAGCCGCTCCTTACCAAGTACCAACCATAGGGGTGTATGGCGTGCCAGGATCAGGCAAGTCTGGC
ATCATTAAAAGCGCAGTCACCAAAAAAGATCTAGTGGTGAGCGCCAAGAAAGAAAACTGTGCAGAAATTATAAGGGACGTCAAGAAAATGAAAGGGC
TGGACGTCAATGCCAGAACTGTGGACTCAGTGCTCTTGAATGGATGCAAACACCCCGTAGAGACCCTGTATATTGACGAAGCTTTTGCTTGTCATGC
AGGTACTCTCAGAGCGCTCATAGCCATTATAAGACCTAAAAAGGCAGTGCTCTGCGGGGATCCCAAACAGTGCGGTTTTTTTAACATGATGTGCCTG
AAAGTGCATTTTAACCACGAGATTTGCACACAAGTCTTCCACAAAAGCATCTCTCGCCGTTGCACTAAATCTGTGACTTCGGTCGTCTCAACCTTGT
TTTACGACAAAAAAATGAGAACGACGAATCCGAAAGAGACTAAGATTGTGATTGACACTACCGGCAGTACCAAACCTAAGCAGGACGATCTCATTCT
CACTTGTTTCAGAGGGTGGGTGAAGCAGTTGCAAATAGATTACAAAGGCAACGAAATAATGACGGCAGCTGCCTCTCAAGGGCTGACCCGTAAAGGT
GTGTATGCCGTTCGGTACAAGGTGAATGAAAATCCTCTGTACGCACCCACCTCAGAACATGTGAACGTCCTACTGACCCGCACGGAGGACCGCATCG
TGTGGAAAACACTAGCCGGCGACCCATGGATAAAAACACTGACTGCCAAGTACCCTGGGAATTTCACTGCCACGATAGAGGAGTGGCAAGCAGAGCA
TGATGCCATCATGAGGCACATCTTGGAGAGACCGGACCCTACCGACGTCTTCCAGAATAAGGCAAACGTGTGTTGGGCCAAGGCTTTAGTGCCGGTG
CTGAAGACCGCTGGCATAGACATGACCACTGAACAATGGAACACTGTGGATTATTTTGAAACGGACAAAGCTCACTCAGCAGAGATAGTATTGAACC
AACTATGCGTGAGGTTCTTTGGACTCGATCTGGACTCCGGTCTATTTTCTGCACCCACTGTTCCGTTATCCATTAGGAATAATCACTGGGATAACTC
CCCGTCGCCTAACATGTACGGGCTGAATAAAGAAGTGGTCCGTCAGCTCTCTCGCAGGTACCCACAACTGCCTCGGGCAGTTGCCACTGGAAGAGTC
TATGACATGAACACTGGTACACTGCGCAATTATGATCCGCGCATAAACCTAGTACCTGTAAACAGAAGACTGCCTCATGCTTTAGTCCTCCACCATA
ATGAACACCCACAGAGTGACTTTTCTTCATTCGTCAGCAAATTGAAGGGCAGAACTGTCCTGGTGGTCGGGGAAAAGTTGTCCGTCCCAGGCAAAAT
GGTTGACTGGTTGTCAGACCGGCCTGAGGCTACCTTCAGAGCTCGGCTGGATTTAGGCATCCCAGGTGATGTGCCCAAATATGACATAATATTTGTT
AATGTGAGGACCCCATATAAATACCATCACTATCAGCAGTGTGAAGACCATGCCATTAAGCTTAGCATGTTGACCAAGAAAGCTTGTCTGCATCTGA
ATCCCGGCGGAACCTGTGTCAGCATAGGTTATGGTTACGCTGACAGGGCCAGCGAAAGCATCATTGGTGCTATAGCGCGGCAGTTCAAGTTTTCCCG
GGTATGCAAACCGAAATCCTCACTTGAAGAGACGGAAGTTCTGTTTGTATTCATTGGGTACGATCGCAAGGCCCGTACGCACAATTCTTACAAGCTT
TCATCAACCTTGACCAACATTTATACAGGTTCCAGACTCCACGAAGCCGGATGTGCACCCTCATATCATGTGGTGCGAGGGGATATTGCCACGGCCA
CCGAAGGAGTGATTATAAATGCTGCTAACAGCAAAGGACAACCTGGCGGAGGGGTGTGCGGAGCGCTGTATAAGAAATTCCCGGAAAGCTTCGATTT
ACAGCCGATCGAAGTAGGAAAAGCGCGACTGGTCAAAGGTGCAGCTAAACATATCATTCATGCCGTAGGACCAAACTTCAACAAAGTTTCGGAGGTT
GAAGGTGACAAACAGTTGGCAGAGGCTTATGAGTCCATCGCTGAGATTGTCAACGATAACAATTACAAGTCAGTAGCGATTCCACTGTTGTCCACCG
GCATCTTTTCCGGGAACAAAGATCGACTAACCCAATCATTGAACCATTTGCTGACAGCTTTAGACACCACTGATGCAGATGTAGCCATATACTGCAG
GGACAAGAAATGGGAAATGACTCTCAAGGAAGCAGTGGCTAGGAGAGAAGCAGTGGAGGAGATATGCATATCCGACGACTCTTCAGTGACAGAACCT
GATGCAGAGCTGGTGAGGGTGCATCCGAAGAGTTCTTTGGCTGGAAGGAAGGGCTACAGCACAAGCGATGGCAAAACTTTCTCATATTTGGAAGGGA
CCAAGTTTCACCAGGCGGCCAAGGATATAGCAGAAATTAATGCCATGTGGCCCGTTGCAACGGAGGCCAATGAGCAGGTATGCATGTATATCCTCGG
AGAAAGCATGAGCAGTATTAGGTCGAAATGCCCCGTCGAAGAGTCGGAAGCCTCCACACCACCTAGCACGCTGCCTTGCTTGTGCATCCATGCCATG
ACTCCAGAAAGAGTACAGCGCCTAAAAGCCTCACGTCCAGAACAAATTACTGTGTGCTCATCCTTTCCATTGCCGAAGTATAGAATCACTGGTGTGC
AGAAGATCCAATGCTCCCAGCCTATATTGTTCTCACCGAAAGTGCCTGCGTATATTCATCCAAGGAAGTATCTCGTGGAAACACCACCGGTAGACGA
GACTCCGGAGCCATCGGCAGAGAACCAATCCACAGAGGGGACACCTGAACAACCACCACTTATAACCGAGGATGAGACCAGGACTAGAACGCCTGAG
CCGATCATCATCGAAGAGGAAGAAGAGGATAGCATAAGTTTGCTGTCAGATGGCCCGACCCACCAGGTGCTGCAAGTCGAGGCAGACATTCACGGGC
CGCCCTCTGTATCTAGCTCATCCTGGTCCATTCCTCATGCATCCGACTTTGATGTGGACAGTTTATCCATACTTGACACCCTGGAGGGAGCTAGCGT
GACCAGCGGGGCAACGTCAGCCGAGACTAACTCTTACTTCGCAAAGAGTATGGAGTTTCTGGCGCGACCGGTGCCTGCGCCTCGAACAGTATTCAGG
AACCCTCCACATCCCGCTCCGCGCACAAGAACACCGTCACTTGCACCCAGCAGGGCCTGCTCGAGAACCAGCCTAGTTTCCACCCCGCCAGGCGTGA
ATAGGGTGATCACTAGAGAGGAGCTCGAGGCGCTTACCCCGTCACGCACTCCTAGCAGGTCGGTCTCGAGAACCAGCCTGGTCTCCAACCCGCCAGG
CGTAAATAGGGTGATTACAAGAGAGGAGTTTGAGGCGTTCGTAGCACAACAACAATGACGGTTTGATGCGGGTGCATACATCTTTTCCTCCGACACC
GGTCAAGGGCATTTACAACAAAAATCAGTAAGGCAAACGGTGCTATCCGAAGTGGTGTTGGAGAGGACCGAATTGGAGATTTCGTATGCCCCGCGCC
TCGACCAAGAAAAAGAAGAATTACTACGCAAGAAATTACAGTTAAATCCCACACCTGCTAACAGAAGCAGATACCAGTCCAGGAAGGTGGAGAACAT
GAAAGCCATAACAGCTAGACGTATTCTGCAAGGCCTAGGGCATTATTTGAAGGCAGAAGGAAAAGTGGAGTGCTACCGAACCCTGCATCCTGTTCCT
TTGTATTCATCTAGTGTGAACCGTGCCTTTTCAAGCCCCAAGGTCGCAGTGGAAGCCTGTAACGCCATGTTGAAAGAGAACTTTCCGACTGTGGCTT
CTTACTGTATTATTCCAGAGTACGATGCCTATTTGGACATGGTTGACGGAGCTTCATGCTGCTTAGACACTGCCAGTTTTTGCCCTGCAAAGCTGCG
CAGCTTTCCAAAGAAACACTCCTATTTGGAACCCACAATACGATCGGCAGTGCCTTCAGCGATCCAGAACACGCTCCAGAACGTCCTGGCAGCTGCC
ACAAAAAGAAATTGCAATGTCACGCAAATGAGAGAATTGCCCGTATTGGATTCGGCGGCCTTTAATGTGGAATGCTTCAAGAAATATGCGTGTAATA
ATGAATATTGGGAAACGTTTAAAGAAAACCCCATCAGGCTTACTGAAGAAAACGTGGTAAATTACATTACCAAATTAAAAGGACCAAAAGCTGCTGC
TCTTTTTGCGAAGACACATAATTTGAATATGTTGCAGGACATACCAATGGACAGGTTTGTAATGGACTTAAAGAGAGACGTGAAAGTGACTCCAGGA

ACAAAACATACTGAAGAACGGCCCAAGGTACAGGTGATCCAGGCTGCCGATCCGCTAGCAACAGCGTATCTGTGCGGAATCCACCGAGAGCTGGTTA
GGAGATTAAATGCGGTCCTGCTTCCGAACATTCATACACTGTTTGATATGTCGGCTGAAGACTTTGACGCTATTATAGCCGAGCACTTCCAGCCTGG
GGATTGTGTTCTGGAAACTGACATCGCGTCGTTTGATAAAAGTGAGGACGACGCCATGGCTCTGACCGCGTTAATGATTCTGGAAGACTTAGGTGTG
GACGCAGAGCTGTTGACGCTGATTGAGGCGGCTTTCGGCGAAATTTCATCAATACATTTGCCCACTAAAACTAAATTTAAATTCGGAGCCATGATGA
AATCTGGAATGTTCCTCACACTGTTTGTGAACACAGTCATTAACATTGTAATCGCAAGCAGAGTGTTGAGAGAACGGCTAACCGGATCACCATGTGC
AGCATTCATTGGAGATGACAATATCGTGAAAGGAGTCAAATCGGACAAATTAATGGCAGACAGGTGCGCCACCTGGTTGAATATGGAAGTCAAGATT
ATAGATGCTGTGGTGGGCGAGAAAGCGCCTTATTTCTGTGGAGGGTTTATTTTGTGTGACTCCGTGACCGGCACAGCGTGCCGTGTGGCAGACCCCC
TAAAAAGGCTGTTTAAGCTTGGCAAACCTCTGGCAGCAGACGATGAACATGATGATGACAGGAGAAGGGCATTGCATGAAGAGTCAACACGCTGGAA
CCGAGTGGGTATTCTTTCAGAGCTGTGCAAGGCAGTAGAATCAAGGTATGAAACCGTAGGAACTTCCATCATAGTTATGGCCATGACTACTCTAGCT
AGCAGTGTTAAATCATTCAGCTACCTGAGAGGGGCCCCTATAACTCTCTACGGCTAACCTGAATGGACTACGACATAGTCTAGTCCGCCAAGTCTAG
catatgggcgcgccctcagcatcgattgaattggccaccATGGTGAGCAAGGGCGAGGAGGATAACATGGCCATCATCAAGGAGTTCATGCGCTTCA
AGGTGCACATGGAGGGCTCCGTGAACGGCCACGAGTTCGAGATCGAGGGCGAGGGCGAGGGCCGCCCCTACGAGGGCACCCAGACCGCCAAGCTGAA
GGTGACCAAGGGTGGCCCCCTGCCCTTCGCCTGGGACATCCTGTCCCCTCAGTTCATGTACGGCTCCAAGGCCTACGTGAAGCACCCCGCCGACATC
CCCGACTACTTGAAGCTGTCCTTCCCCGAGGGCTTCAAGTGGGAGCGCGTGATGAACTTCGAGGACGGCGGCGTGGTGACCGTGACCCAGGACTCCT
CCCTGCAGGACGGCGAGTTCATCTACAAGGTGAAGCTGCGCGGCACCAACTTCCCCTCCGACGGCCCCGTAATGCAGAAGAAGACCATGGGCTGGGA
GGCCTCCTCCGAGCGGATGTACCCCGAGGACGGCGCCCTGAAGGGCGAGATCAAGCAGAGGCTGAAGCTGAAGGACGGCGGCCACTACGACGCTGAG
GTCAAGACCACCTACAAGGCCAAGAAGCCCGTGCAGCTGCCCGGCGCCTACAACGTCAACATCAAGTTGGACATCACCTCCCACAACGAGGACTACA
CCATCGTGGAACAGTACGAACGCGCCGAGGGCCGCCACTCCACCGGCGGCATGGACGAGCTGTACAAGTAGgaattGGCAAGCTGCTTACATAGAAC
TCGCGGCGATTGGCATGCCGCCTTAAAATTTTTATTTTATTTTTCTTTTCTTTTCCGAATCGGATTTTGTTTTTAATATTTCAAAAAAAAAAAAAAA
AAAAAAAAAAACGCGTCGAGGGGAATTAATTCTTGAAGACGAAAGGGCCAGGTGGCACTTTTCGGGGAAATGTGCGCGGAACCCCTATTTGTTTATT
TTTCTAAATACATTCAAATATGTATCCGCTCATGAGACAATAACCCTGATAAATGCTTCAATAATATTGAAAAAGGAAGAGTATGAGTATTCAACAT
TTCCGTGTCGCCCTTATTCCCTTTTTTGCGGCATTTTGCCTTCCTGTTTTTGCTCACCCAGAAACGCTGGTGAAAGTAAAAGATGCTGAAGATCAGT
TGGGTGCACGAGTGGGTTACATCGAACTGGATCTCAACAGCGGTAAGATCCTTGAGAGTTTTCGCCCCGAAGAACGTTTTCCAATGATGAGCACTTT
TAAAGTTCTGCTATGTGGCGCGGTATTATCCCGTGTTGACGCCGGGCAAGAGCAACTCGGTCGCCGCATACACTATTCTCAGAATGACTTGGTTGAG
TACTCACCAGTCACAGAAAAGCATCTTACGGATGGCATGACAGTAAGAGAATTATGCAGTGCTGCCATAACCATGAGTGATAACACTGCGGCCAACT
TACTTCTGACAACGATCGGAGGACCGAAGGAGCTAACCGCTTTTTTGCACAACATGGGGGATCATGTAACTCGCCTTGATCGTTGGGAACCGGAGCT
GAATGAAGCCATACCAAACGACGAGCGTGACACCACGATGCCTGTAGCAATGGCAACAACGTTGCGCAAACTATTAACTGGCGAACTACTTACTCTA
GCTTCCCGGCAACAATTAATAGACTGGATGGAGGCGGATAAAGTTGCAGGACCACTTCTGCGCTCGGCCCTTCCGGCTGGCTGGTTTATTGCTGATA
AATCTGGAGCCGGTGAGCGTGGGTCTCGCGGTATCATTGCAGCACTGGGGCCAGATGGTAAGCCCTCCCGTATCGTAGTTATCTACACGACGGGGAG
TCAGGCAACTATGGATGAACGAAATAGACAGATCGCTGAGATAGGTGCCTCACTGATTAAGCATTGGTAACTGTCAGACCAAGTTTACTCATATATA
CTTTAGATTGATTTAAAACTTCATTTTTAATTTAAAAGGATCTAGGTGAAGATCCTTTTTGATAATCTCATGACCAAAATCCCTTAACGTGAGTTTT
CGTTCCACTGAGCGTCAGACCCCGTAGAAAAGATCAAAGGATCTTCTTGAGATCCTTTTTTTCTGCGCGTAATCTGCTGCTTGCAAACAAAAAAACC
ACCGCTACCAGCGGTGGTTTGTTTGCCGGATCAAGAGCTACCAACTCTTTTTCCGAAGGTAACTGGCTTCAGCAGAGCGCAGATACCAAATACTGTC
CTTCTAGTGTAGCCGTAGTTAGGCCACCACTTCAAGAACTCTGTAGCACCGCCTACATACCTCGCTCTGCTAATCCTGTTACCAGTGGCTGCTGCCA
GTGGCGATAAGTCGTGTCTTACCGGGTTGGACTCAAGACGATAGTTACCGGATAAGGCGCAGCGGTCGGGCTGAACGGGGGGTTCGTGCACACAGCC
CAGCTTGGAGCGAACGACCTACACCGAACTGAGATACCTACAGCGTGAGCATTGAGAAAGCGCCACGCTTCCCGAAGGGAGAAAGGCGGACAGGTAT
CCGGTAAGCGGCAGGGTCGGAACAGGAGAGCGCACGAGGGAGCTTCCAGGGGGAAACGCCTGGTATCTTTATAGTCCTGTCGGGTTTCGCCACCTCT
GACTTGAGCGTCGATTTTTGTGATGCTCGTCAGGGGGGCGGAGCCTATGGAAAAACGCCAGCAACGCGAGCTCTAATACGACTCACTATAG

>ab2C-mCherry
ATGGGCGGCGCATGAGAGAAGCCCAGACCAATTACCTACCCAAAATGGAGAAAGTTCACGTTGACATCGAGGAAGACAGCCCATTCCTCAGAGCTTT
GCAGCGGAGCTTCCCGCAGTTTGAGGTAGAAGCCAAGCAGGTCACTGATAATGACCATGCTAATGCCAGAGCGTTTTCGCATCTGGCTTCAAAACTG
ATCGAAACGGAGGTGGACCCATCCGACACGATCCTTGACATTGGAAGTGCGCCCGCCCGCAGAATGTATTCTAAGCACAAGTATCATTGTATCTGTC
CGATGAGATGTGCGGAAGATCCGGACAGATTGTATAAGTATGCAACTAAGCTGAAGAAAAACTGTAAGGAAATAACTGATAAGGAATTGGACAAGAA
AATGAAGGAGCTGGCCGCCGTCATGAGCGACCCTGACCTGGAAACTGAGACTATGTGCCTCCACGACGACGAGTCGTGTCGCTACGAAGGGCAAGTC
GCTGTTTACCAGGATGTATACGCGGTTGACGGACCGACAAGTCTCTATCACCAAGCCAATAAGGGAGTTAGAGTCGCCTACTGGATAGGCTTTGACA
CCACCCCTTTTATGTTTAAGAACTTGGCTGGAGCATATCCATCATACTCTACCAACTGGGCCGACGAAACCGTGTTAACGGCTCGTAACATAGGCCT
ATGCAGCTCTGACGTTATGGAGCGGTCACGTAGAGGGATGTCCATTCTTAGAAAGAAGTATTTGAAACCATCCAACAATGTTCTATTCTCTGTTGGC
TCGACCATCTACCACGAGAAGAGGGACTTACTGAGGAGCTGGCACCTGCCGTCTGTATTTCACTTACGTGGCAAGCAAAATTACACATGTCGGTGTG
AGACTATAGTTAGTTGCGACGGGTACGTCGTTAAAAGAATAGCTATCAGTCCAGGCCTGTATGGGAAGCCTTCAGGCTATGCTGCTACGATGCACCG
CGAGGGATTCTTGTGCTGCAAAGTGACAGACACATTGAACGGGGAGAGGGTCTCTTTTCCCGTGTGCACGTATGTGCCAGCTACATTGTGTGACCAA
ATGACTGGCATACTGGCAACAGATGTCAGTGCGGACGACGCGCAAAAACTGCTGGTTGGGCTCAACCAGCGTATAGTCGTCAACGGTCGCACCCAGA
GAAACACCAATACCATGAAAAATTACCTTTTGCCCGTAGTGGCCCAGGCATTTGCTAGGTGGGCAAAGGAATATAAGGAAGATCAAGAAGATGAAAG
GCCACTAGGACTACGAGATAGACAGTTAGTCATGGGGTGTTGTTGGGCTTTTAGAAGGCACAAGATAACATCTATTTATAAGCGCCCGGATACCCAA
ACCATCATCAAAGTGAACAGCGATTTCCACTCATTCGTGCTGCCCAGGATAGGCAGTAACACATTGGAGATCGGGCTGAGAACAAGAATCAGGAAAA
TGTTAGAGGAGCACAAGGAGCCGTCACCTCTCATTACCGCCGAGGACGTACAAGAAGCTAAGTGCGCAGCCGATGAGGCTAAGGAGGTGCGTGAAGC
CGAGGAGTTGCGCGCAGCTCTACCACCTTTGGCAGCTGATGTTGAGGAGCCCACTCTGGAGGCAGACGTCGACTTGATGTTACAAGAGGCTGGGGCC
GGCTCAGTGGAGACACCTCGTGGCTTGATAAAGGTTACCAGCTACGATGGCGAGGACAAGATCGGCTCTTACGCTGTGCTTTCTCCGCAGGCTGTAC
TCAAGAGTGAAAAATTATCTTGCATCCACCCTCTCGCTGAACAAGTCATAGTGATAACACACTCTGGCCGAAAAGGGCGTTATGCCGTGGAACCATA
CCATGGTAAAGTAGTGGTGCCAGAGGGACATGCAATACCCGTCCAGGACTTTCAAGCTCTGAGTGAAAGTGCCACCATTGTGTACAACGAACGTGAG
TTCGTAAACAGGTACCTGCACCATATTGCCACACATGGGGGAGCGCTGAACACTGATGAAGAATATTACAAAACTGTCAAGCCCAGCGAGCACGACG
GCGAATACCTGTACGACATCGACAGGAAACAGTGCGTCAAGAAAGAACTAGTCACTGGGCTAGGGCTCACAGGCGAGCTGGTGGATCCTCCCTTCCA
TGAATTCGCCTACGAGAGTCTGAGAACACGACCAGCCGCTCCTTACCAAGTACCAACCATAGGGGTGTATGGCGTGCCAGGATCAGGCAAGTCTGGC
ATCATTAAAAGCGCAGTCACCAAAAAAGATCTAGTGGTGAGCGCCAAGAAAGAAAACTGTGCAGAAATTATAAGGGACGTCAAGAAAATGAAAGGGC
TGGACGTCAATGCCAGAACTGTGGACTCAGTGCTCTTGAATGGATGCAAACACCCCGTAGAGACCCTGTATATTGACGAAGCTTTTGCTTGTCATGC
AGGTACTCTCAGAGCGCTCATAGCCATTATAAGACCTAAAAAGGCAGTGCTCTGCGGGGATCCCAAACAGTGCGGTTTTTTAACATGATGTGCCTG
AAAGTGCATTTTAACCACGAGATTTGCACACAAGTCTTCCACAAAAGCATCTCTCGCCGTTGCACTAAATCTGTGACTTCGGTCGTCTCAACCTTGT
TTTACGACAAAAAAATGAGAACGACGAATCCGAAAGAGACTAAGATTGTGATTGACACTACCGGCAGTACCAAACCTAAGCAGGACGATCTCATTCT
CACTTGTTTCAGAGGGTGGGTGAAGCAGTTGCAAATAGATTACAAAGGCAACGAAATAATGACGGCAGCTGCCTCTCAAGGGCTGACCCGTAAAGGT
GTGTATGCCGTTCGGTACAAGGTGAATGAAAATCCTCTGTACGCACCCACCTCAGAACATGTGAACGTCCTACTGACCCGCACGGAGGACCGCATCG
TGTGGAAAACACTAGCCGGCGACCCATGGATAAAAACACTGACTGCCAAGTACCCTGGGAATTTCACTGCCACGATAGAGGAGTGGCAAGCAGAGCA
TGATGCCATCATGAGGCACATCTTGGAGAGACCGGACCCTACCGACGTCTTCCAGAATAAGGCAAACGTGTGTTGGGCCAAGGCTTTAGTGCCGGTG

CTGAAGACCGCTGGCATAGACATGACCACTGAACAATGGAACACTGTGGATTATTTTGAAACGGACAAAGCTCACTCAGCAGAGATAGTATTGAACC
AACTATGCGTGAGGTTCTTTGGACTCGATCTGGACTCCGGTCTATTTTCTGCACCCACTGTTCCGTTATCCATTAGGAATAATCACTGGGATAACTC
CCCGTCGCCTAACATGTACGGGCTGAATAAAGAAGTGGTCCGTCAGCTCTCTCGCAGGTACCCACAACTGCCTCGGGCAGTTGCCACTGGAAGAGTC
TATGACATGAACACTGGTACACTGCGCAATTATGATCCGCGCATAAACCTAGTACCTGTAAACAGAAGACTGCCTCATGCTTTAGTCCTCCACCATA
ATGAACACCCACAGAGTGACTTTTCTTCATTCGTCAGCAAATTGAAGGGCAGAACTGTCCTGGTGGTCGGGGAAAAGTTGTCCGTCCCAGGCAAAAT
GGTTGACTGGTTGTCAGACCGGCCTGAGGCTACCTTCAGAGCTCGGCTGGATTTAGGCATCCCAGGTGATGTGCCCAAATATGACATAATATTTGTT
AATGTGAGGACCCCATATAAATACCATCACTATCAGCAGTGTGAAGACCATGCCATTAAGCTTAGCATGTTGACCAAGAAAGCTTGTCTGCATCTGA
ATCCCGGCGGAACCTGTGTCAGCATAGGTTATGGTTACGCTGACAGGGCCAGCGAAAGCATCATTGGTGCTATAGCGCGGCAGTTCAAGTTTTCCCG
GGTATGCAAACCGAAATCCTCACTTGAAGAGACGGAAGTTCTGTTTGTATTCATTGGGTACGATCGCAAGGCCCGTACGCACAATTCTTACAAGCTT
TCATCAACCTTGACCAACATTTATACAGGTTCCAGACTCCACGAAGCCGGATGTGCACCCTCATATCATGTGGTGCGAGGGGATATTGCCACGGCCA
CCGAAGGAGTGATTATAAATGCTGCTAACAGCAAAGGACAACCTGGCGGAGGGGTGTGCGGAGCGCTGTATAAGAAATTCCCGGAAAGCTTCGATTT
ACAGCCGATCGAAGTAGGAAAAGCGCGACTGGTCAAAGGTGCAGCTAAACATATCATTCATGCCGTAGGACCAAACTTCAACAAAGTTTCGGAGGTT
GAAGGTGACAAACAGTTGGCAGAGGCTTATGAGTCCATCGCTGAGATTGTCAACGATAACAATTACAAGTCAGTAGCGATTCCACTGTTGTCCACCG
GCATCTTTTCCGGGAACAAAGATCGACTAACCCAATCATTGAACCATTTGCTGACAGCTTTAGACACCACTGATGCAGATGTAGCCATATACTGCAG
GGACAAGAAATGGGAAATGACTCTCAAGGAAGCAGTGGCTAGGAGAGAAGCAGTGGAGGAGATATGCATATCCGACGACTCTTCAGTGACAGAACCT
GATGCAGAGCTGGTGAGGGTGCATCCGAAGAGTTCTTTGGCTGGAAGGAAGGGCTACAGCACAAGCGATGGCAAAACTTTCTCATATTTGGAAGGGA
CCAAGTTTCACCAGGCGGCCAAGGATATAGCAGAAATTAATGCCATGTGGCCCGTTGCAACGGAGGCCAATGAGCAGGTATGCATGTATATCCTCGG
AGAAAGCATGAGCAGTATTAGGTCGAAATGCCCCGTCGAAGAGTCGGAAGCCTCCACACCACCTAGCACGCTGCCTTGCTTGTGCATCCATGCCATG
ACTCCAGAAAGAGTACAGCGCCTAAAAGCCTCACGTCCAGAACAAATTACTGTGTGCTCATCCTTTCCATTGCCGAAGTATAGAATCACTGGTGTGC
AGAAGATCCAATGCTCCCAGCCTATATTGTTCTCACCGAAAGTGCCTGCGTATATTCATCCAAGGAAGTATCTCGTGGAAACACCACCGGTAGACGA
GACTCCGGAGCCATCGGCAGAGAACCAATCCACAGAGGGGACACCTGAACAACCACCACTTATAACCGAGGATGAGACCAGGACTAGAACGCCTGAG
CCGATCATCATCGAAGAGGAAGAAGAGGATAGCATAAGTTTGCTGTCAGATGGCCCGACCCACCAGGTGCTGCAAGTCGAGGCAGACATTCACGGGC
CGCCCTCTGTATCTAGCTCATCCTGGTCCATTCCTCATGCATCCGACTTTGATGTGGACAGTTTATCCATACTTGACACCCTGGAGGGAGCTAGCGT
GACCAGCGGGGCAACGTCAGCCGAGACTAACTCTTACTTCGCAAAGAGTATGGAGTTTCTGGCGCGACCGGTGCCTGCGCCTCGAACAGTATTCAGG
AACCCTCCACATCCCGCTCCGCGCACAAGAACACCGTCACTTGCACCCAGCAGGGCCTGCTCGAGAACCAGCCTAGTTTCCACCCCGCCAGGCGTGA
ATAGGGTGATCACTAGAGAGGAGCTCGAGGCGCTTACCCCGTCACGCACTCCTAGCAGGTCGGTCTCGAGAACCAGCCTGGTCTCCAACCCGCCAGG
CGTAAATAGGGTGATTACAAGAGAGGAGTTTGAGGCGTTCGTAGCACAACAACAATGACGGTTTGATGCGGGTGCATACATCTTTTCCTCCGACACC
GGTCAAGGGCATTTACAACAAAAATCAGTAAGGCAAACGGTGCTATCCGAAGTGGTGTTGGAGAGGACCGAATTGGAGATTTCGTATGCCCCGCGCC
TCGACCAAGAAAAAGAAGAATTACTACGCAAGAAATTACAGTTAAATCCCACACCTGCTAACAGAAGCAGATACCAGTCCAGGAAGGTGGAGAACAT
GAAAGCCATAACAGCTAGACGTATTCTGCAAGGCCTAGGGCATTATTTGAAGGCAGAAGGAAAAGTGGAGTGCTACCGAACCCTGCATCCTGTTCCT
TTGTATTCATCTAGTGTGAACCGTGCCTTTTCAAGCCCCAAGGTCGCAGTGGAAGCCTGTAACGCCATGTTGAAAGAGAACTTTCCGACTGTGGCTT
CTTACTGTATTATTCCAGAGTACGATGCCTATTTGGACATGGTTGACGGAGCTTCATGCTGCTTAGACACTGCCAGTTTTTGCCCTGCAAAGCTGCG
CAGCTTTCCAAAGAAACACTCCTATTTGGAACCCACAATACGATCGGCAGTGCCTTCAGCGATCCAGAACACGCTCCAGAACGTCCTGGCAGCTGCC
ACAAAAAGAAATTGCAATGTCACGCAAATGAGAGAATTGCCCGTATTGGATTCGGCGGCCTTTAATGTGGAATGCTTCAAGAAATATGCGTGTAATA
ATGAATATTGGGAAACGTTTAAAGAAAACCCCATCAGGCTTACTGAAGAAAACGTGGTAAATTACATTACCAAATTAAAAGGACCAAAAGCTGCTGC
TCTTTTTGCGAAGACACATAATTTGAATATGTTGCAGGACATACCAATGGACAGGTTTGTAATGGACTTAAAGAGAGACGTGAAAGTGACTCCAGGA
ACAAAACATACTGAAGAACGGCCCAAGGTACAGGTGATCCAGGCTGCCGATCCGCTAGCAACAGCGTATCTGTGCGGAATCCACCGAGAGCTGGTTA
GGAGATTAAATGCGGTCCTGCTTCCGAACATTCATACACTGTTTGATATGTCGGCTGAAGACTTTGACGCTATTATAGCCGAGCACTTCCAGCCTGG
GGATTGTGTTCTGGAAACTGACATCGCGTCGTTTGATAAAAGTGAGGACGACGCCATGGCTCTGACCGCGTTAATGATTCTGGAAGACTTAGGTGTG
GACGCAGAGCTGTTGACGCTGATTGAGGCGGCTTTCGGCGAAATTTCATCAATACATTTGCCCACTAAAACTAAATTTAAATTCGGAGCCATGATGA
AATCTGGAATGTTCCTCACACTGTTTGTGAACACAGTCATTAACATTGTAATCGCAAGCAGAGTGTTGAGAGAACGGCTAACCGGATCACCATGTGC
AGCATTCATTGGAGATGACAATATCGTGAAAGGAGTCAAATCGGACAAATTAATGGCAGACAGGTGCGCCACCTGGTTGAATATGGAAGTCAAGATT
ATAGATGCTGTGGTGGGCGAGAAAGCGCCTTATTTCTGTGGAGGGTTTATTTTGTGTGACTCCGTGACCGGCACAGCGTGCCGTGTGGCAGACCCCC
TAAAAAGGCTGTTTAAGCTTGGCAAACCTCTGGCAGCAGACGATGAACATGATGATGACAGGAGAAGGGCATTGCATGAAGAGTCAACACGCTGGAA
CCGAGTGGGTATTCTTTCAGAGCTGTGCAAGGCAGTAGAATCAAGGTATGAAACCGTAGGAACTTCCATCATAGTTATGGCCATGACTACTCTAGCT
AGCAGTGTTAAATCATTCAGCTACCTGAGAGGGGCCCCTATAACTCTCTACGGCTAACCTGAATGGACTACGACATAGTCTAGTCCGCCAAGTCTAG
catatgggcgcgccctcagcatcgattgaattggccaccATGGTGAGCAAGGGCGAGGAGGATAACATGGCCATCATCAAGGAGTTCATGCGCTTCA
AGGTGCACATGGAGGGCTCCGTGAACGGCCACGAGTTCGAGATCGAGGGCGAGGGCGAGGGCCGCCCCTACGAGGGCACCCAGACCGCCAAGCTGAA
GGTGACCAAGGGTGGCCCCCTGCCCTTCGCCTGGGACATCCTGTCCCCTCAGTTCATGTACGGCTCCAAGGCCTACGTGAAGCACCCCGCCGACATC
CCCGACTACTTGAAGCTGTCCTTCCCCGAGGGCTTCAAGTGGGAGCGCGTGATGAACTTCGAGGACGGCGGCGTGGTGACCGTGACCCAGGACTCCT
CCCTGCAGGACGGCGAGTTCATCTACAAGGTGAAGCTGCGCGGCACCAACTTCCCCTCCGACGGCCCCGTAATGCAGAAGAAGACCATGGGCTGGGA
GGCCTCCTCCGAGCGGATGTACCCCGAGGACGGCGCCCTGAAGGGCGAGATCAAGCAGAGGCTGAAGCTGAAGGACGGCGGCCACTACGACGCTGAG
GTCAAGACCACCTACAAGGCCAAGAAGCCCGTGCAGCTGCCCGGCGCCTACAACGTCAACATCAAGTTGGACATCACCTCCCACAACGAGGACTACA
CCATCGTGGAACAGTACGAACGCGCCGAGGGCCGCCACTCCACCGGCGGCATGGACGAGCTGTACAAGTAGgaattGGCAAGCTGCTTACATAGAAC
TCGCGGCGATTGGCATGCCGCCTTAAAATTTTTATTTTATTTTTCTTTTCTTTTCCGAATCGGATTTTGTTTTTAATATTTCAAAAAAAAAAAAAAA
AAAAAAAAAAACGCGTCGAGGGGAATTAATTCTTGAAGACGAAAGGGCCAGGTGGCACTTTTCGGGGAAATGTGCGCGGAACCCCTATTTGTTTATT
TTTCTAAATACATTCAAATATGTATCCGCTCATGAGACAATAACCCTGATAAATGCTTCAATAATATTGAAAAAGGAAGAGTATGAGTATTCAACAT
TTCCGTGTCGCCCTTATTCCCTTTTTTGCGGCATTTTGCCTTCCTGTTTTTGCTCACCCAGAAACGCTGGTGAAAGTAAAAGATGCTGAAGATCAGT
TGGGTGCACGAGTGGGTTACATCGAACTGGATCTCAACAGCGGTAAGATCCTTGAGAGTTTTCGCCCCGAAGAACGTTTTCCAATGATGAGCACTTT
TAAAGTTCTGCTATGTGGCGCGGTATTATCCCGTGTTGACGCCGGGCAAGAGCAACTCGGTCGCCGCATACACTATTCTCAGAATGACTTGGTTGAG
TACTCACCAGTCACAGAAAAGCATCTTACGGATGGCATGACAGTAAGAGAATTATGCAGTGCTGCCATAACCATGAGTGATAACACTGCGGCCAACT
TACTTCTGACAACGATCGGAGGACCGAAGGAGCTAACCGCTTTTTTGCACAACATGGGGGATCATGTAACTCGCCTTGATCGTTGGGAACCGGAGCT
GAATGAAGCCATACCAAACGACGAGCGTGACACCACGATGCCTGTAGCAATGGCAACAACGTTGCGCAAACTATTAACTGGCGAACTACTTACTCTA
GCTTCCCGGCAACAATTAATAGACTGGATGGAGGCGGATAAAGTTGCAGGACCACTTCTGCGCTCGGCCCTTCCGGCTGGCTGGTTTATTGCTGATA
AATCTGGAGCCGGTGAGCGTGGGTCTCGCGGTATCATTGCAGCACTGGGGCCAGATGGTAAGCCCTCCCGTATCGTAGTTATCTACACGACGGGGAG
TCAGGCAACTATGGATGAACGAAATAGACAGATCGCTGAGATAGGTGCCTCACTGATTAAGCATTGGTAACTGTCAGACCAAGTTTACTCATATATA
CTTTAGATTGATTTAAAACTTCATTTTTAATTTAAAAGGATCTAGGTGAAGATCCTTTTTGATAATCTCATGACCAAAATCCCTTAACGTGAGTTTT
CGTTCCACTGAGCGTCAGACCCCGTAGAAAAGATCAAAGGATCTTCTTGAGATCCTTTTTTTCTGCGCGTAATCTGCTGCTTGCAAACAAAAAAACC
ACCGCTACCAGCGGTGGTTTGTTTGCCGGATCAAGAGCTACCAACTCTTTTTCCGAAGGTAACTGGCTTCAGCAGAGCGCAGATACCAAATACTGTC
CTTCTAGTGTAGCCGTAGTTAGGCCACCACTTCAAGAACTCTGTAGCACCGCCTACATACCTCGCTCTGCTAATCCTGTTACCAGTGGCTGCTGCCA
GTGGCGATAAGTCGTGTCTTACCGGGTTGGACTCAAGACGATAGTTACCGGATAAGGCGCAGCGGTCGGGCTGAACGGGGGGTTCGTGCACACAGCC
CAGCTTGGAGCGAACGACCTACACCGAACTGAGATACCTACAGCGTGAGCATTGAGAAAGCGCCACGCTTCCCGAAGGGAGAAAGGCGGACAGGTAT

CCGGTAAGCGGCAGGGTCGGAACAGGAGAGCGCACGAGGGAGCTTCCAGGGGGAAACGCCTGGTATCTTTATAGTCCTGTCGGGTTTCGCCACCTCT
GACTTGAGCGTCGATTTTTGTGATGCTCGTCAGGGGGGCGGAGCCTATGGAAAAACGCCAGCAACGCGAGCTCTAATACGACTCACTATAG

>ABc1-mCherry
ATGGGCGGCGCATGAGAGAAGCCCAGACCAATTACCTACCCAAAATGGAGAAAGTTCACGTTGACATCGAGGAAGACAGCCCATTCCTCAGAGCTTT
GCAGCGGAGCTTCCCGCAGTTTGAGGTAGAAGCCAAGCAGGTCACTGATAATGACCATGCTAATGCCAGAGCGTTTTCGCATCTGGCTTCAAAACTG
ATCGAAACGGAGGTGGACCCATCCGACACGATCCTTGACATTGGAAGTGCGCCCGCCCGCAGAATGTATTCTAAGCACAAGTATCATTGTATCTGTC
CGATGAGATGTGCGGAAGATCCGGACAGATTGTATAAGTATGCAACTAAGCTGAAGAAAAACTGTAAGGAAATAACTGATAAGGAATTGGACAAGAA
AATGAAGGAGCTGGCCGCCGTCATGAGCGACCCTGACCTGGAAACTGAGACTATGTGCCTCCACGACGACGAGTCGTGTCGCTACGAAGGGCAAGTC
GCTGTTTACCAGGATGTATACGCGGTTGACGGACCGACAAGTCTCTATCACCAAGCCAATAAGGGAGTTAGAGTCGCCTACTGGATAGGCTTTGACA
CCACCCCTTTTATGTTTAAGAACTTGGCTGGAGCATATCCATCATACTCTACCAACTGGGCCGACGAAACCGTGTTAACGGCTCGTAACATAGGCCT
ATGCAGCTCTGACGTTATGGAGCGGTCACGTAGAGGGATGTCCATTCTTAGAAAGAAGTATTTGAAACCATCCAACAATGTTCTATTCTCTGTTGGC
TCGACCATCTACCACGAGAAGAGGGACTTACTGAGGAGCTGGCACCTGCCGTCTGTATTTCACTTACGTGGCAAGCAAAATTACACATGTCGGTGTG
AGACTATAGTTAGTTGCGACGGGTACGTCGTTAAAAGAATAGCTATCAGTCCAGGCCTGTATGGGAAGCCTTCAGGCTATGCTGCTACGATGCACCG
CGAGGGATTCTTGTGCTGCAAAGTGACAGACACATTGAACGGGGAGAGGGTCTCTTTTCCCGTGTGCACGTATGTGCCAGCTACATTGTGTGACCAA
ATGACTGGCATACTGGCAACAGATGTCAGTGCGGACGACGCGCAAAAACTGCTGGTTGGGCTCAACCAGCGTATAGTCGTCAACGGTCGCACCCAGA
GAAACACCAATACCATGAAAAATTACCTTTTGCCCGTAGTGGCCCAGGCATTTGCTAGGTGGGCAAAGGAATATAAGGAAGATCAAGAAGATGAAAG
GCCACTAGGACTACGAGATAGACAGTTAGTCATGGGGTGTTGTTGGGCTTTTAGAAGGCACAAGATAACATCTATTTATAAGCGCCCGGATACCCAA
ACCATCATCAAAGTGAACAGCGATTTCCACTCATTCGTGCTGCCCAGGATAGGCAGTAACACATTGGAGATCGGGCTGAGAACAAGAATCAGGAAAA
TGTTAGAGGAGCACAAGGAGCCGTCACCTCTCATTACCGCCGAGGACGTACAAGAAGCTAAGTGCGCAGCCGATGAGGCTAAGGAGGTGCGTGAAGC
CGAGGAGTTGCGCGCAGCTCTACCACCTTTGGCAGCTGATGTTGAGGAGCCCACTCTGGAGGCAGACGTCGACTTGATGTTACAAGAGGCTGGGGCC
GGCTCAGTGGAGACACCTCGTGGCTTGATAAAGGTTACCAGCTACGATGGCGAGGACAAGATCGGCTCTTACGCTGTGCTTTCTCCGCAGGCTGTAC
TCAAGAGTGAAAAATTATCTTGCATCCACCCTCTCGCTGAACAAGTCATAGTGATAACACACTCTGGCCGAAAAGGGCGTTATGCCGTGGAACCATA
CCATGGTAAAGTAGTGGTGCCAGAGGGACATGCAATACCCGTCCAGGACTTTCAAGCTCTGAGTGAAAGTGCCACCATTGTGTACAACGAACGTGAG
TTCGTAAACAGGTACCTGCACCATATTGCCACACATGGAGGAGCGCTGAACACTGATGAAGAATATTACAAAACTGTCAAGCCCAGCGAGCACGACG
GCGAATACCTGTACGACATCGACAGGAAACAGTGCGTCAAGAAAGAACTAGTCACTGGGCTAGGGCTCACAGGCGAGCTGGTGGATCCTCCCTTCCA
TGAATTCGCCTACGAGAGTCTGAGAACACGACCAGCCGCTCCTTACCAAGTACCAACCATAGGGGTGTATGGCGTGCCAGGATCAGGCAAGTCTGGC
ATCATTAAAAGCGCAGTCACCAAAAAAGATCTAGTGGTGAGCGCCAAGAAAGAAAACTGTGCAGAAATTATAAGGGACGTCAAGAAAATGAAAGGGC
TGGACGTCAATGCCAGAACTGTGGACTCAGTGCTCTTGAATGGATGCAAACACCCCGTAGAGACCCTGTATATTGACGAAGCTTTTGCTTGTCATGC
AGGTACTCTCAGAGCGCTCATAGCCATTATAAGACCTAAAAAGGCAGTGCTCTGCGGGGATCCCAAACAGTGCGGTTTTTTTAACATGATGTGCCTG
AAAGTGCATTTTAACCACGAGATTTGCACACAAGTCTTCCACAAAAGCATCTCTCGCCGTTGCACTAAATCTGTGACTTCGGTCGTCTCAACCTTGT
TTTACGACAAAAAAATGAGAACGACGAATCCGAAAGAGACTAAGATTGTGATTGACACTACCGGCAGTACCAAACCTAAGCAGGACGATCTCATTCT
CACTTGTTTCAGAGGGTGGGTGAAGCAGTTGCAAATAGATTACAAAGGCAACGAAATAATGACGGCAGCTGCCTCTCAAGGGCTGACCCGTAAAGGT
GTGTATGCCGTTCGGTACAAGGTGAATGAAAATCCTCTGTACGCACCCACCTCAGAACATGTGAACGTCCTACTGACCCGCACGGAGGACCGCATCG
TGTGGAAAACACTAGCCGGCGACCCATGGATAAAAACACTGACTGCCAAGTACCCTGGGAATTTCACTGCCACGATAGAGGAGTGGCAAGCAGAGCA
TGATGCCATCATGAGGCACATCTTGGAGAGACCGGACCCTACCGACGTCTTCCAGAATAAGGCAAACGTGTGTTGGGCCAAGGCTTTAGTGCCGGTG
CTGAAGACCGCTGGCATAGACATGACCACTGAACAATGGAACACTGTGGATTATTTTGAAACGGACAAAGCTCACTCAGCAGAGATAGTATTGAACC
AACTATGCGTGAGGTTCTTTGGACTCGATCTGGACTCCGGTCTATTTTCTGCACCCACTGTTCCGTTATCCATTAGGAATAATCACTGGGATAACTC
CCCGTCGCCTAACATGTACGGGCTGAATAAAGAAGTGGTCCGTCAGCTCTCTCGCAGGTACCCACAACTGCCTCGGGCAGTTGCCACTGGAAGAGTC
TATGACATGAACACTGGTACACTGCGCAATTATGATCCGCGCATAAACCTAGTACCTGTAAACAGAAGACTGCCTCATGCTTTAGTCCTCCACCATA
ATGAACACCCACAGAGTGACTTTTCTTCATTCGTCAGCAAATTGAAGGGCAGAACTGTCCTGGTGGTCGGGGAAAAGTTGTCCGTCCCAGGCAAAAT
GGTTGACTGGTTGTCAGACCGGCCTGAGGCTACCTTCAGAGCTCGGCTGGATTTAGGCATCCCAGGTGATGTGCCCAAATATGACATAATATTTGTT
AATGTGAGGACCCCATATAAATACCATCACTATCAGCAGTGTGAAGACCATGCCATTAAGCTTAGCATGTTGACCAAGAAAGCTTGTCTGCATCTGA
ATCCCGGCGGAACCTGTGTCAGCATAGGTTATGGTTACGCTGACAGGGCCAGCGAAAGCATCATTGGTGCTATAGCGCGGCAGTTCAAGTTTTCCCG
GGTATGCAAACCGAAATCCTCACTTGAAGAGACGGAAGTTCTGTTTGTATTCATTGGGTACGATCGCAAGGCCCGTACGCACAATTCTTACAAGCTT
TCATCAACCTTGACCAACATTTATACAGGTTCCAGACTCCACGAAGCCGGATGTGCACCCTCATATCATGTGGTGCGAGGGGATATTGCCACGGCCA
CCGAAGGAGTGATTATAAATGCTGCTAACAGCAAAGGACAACCTGGCGGAGGGGTGTGCGGAGCGCTGTATAAGAAATTCCCGGAAAGCTTCGATTT
ACAGCCGATCGAAGTAGGAAAAGCGCGACTGGTCAAAGGTGCAGCTAAACATATCATTCATGCCGTAGGACCAAACTTCAACAAAGTTTCGGAGGTT
GAAGGTGACAAACAGTTGGCAGAGGCTTATGAGTCCATCGCTAAGATTGTCAACGATAACAATTACAAGTCAGTAGCGATTCCACTGTTGTCCACCG
GCATCTTTTCCGGGAACAAAGATCGACTAACCCAATCATTGAACCATTTGCTGACAGCTTTAGACACCACTGATGCAGATGTAGCCATATACTGCAG
GGACAAGAAATGGGAAATGACTCTCAAGGAAGCAGTGGCTAGGAGAGAAGCAGTGGAGGAGATATGCATATCCGACGACTCTTCAGTGACAGAACCT
GATGCAGAGCTGGTGAGGGTGCATCCGAAGAGTTCTTTGGCTGGAAGGAAGGGCTACAGCACAAGCGATGGCAAAACTTTCTCATATTTGGAAGGGA
CCAAGTTTCACCAGGCGGCCAAGGATATAGCAGAAATTAATGCCATGTGGCCCGTTGCAACGGAGGCCAATGAGCAGGTATGCATGTATATCCTCGG
AGAAGGCATGAGCAGTATTAGGTCGAAATGCCCCGTCGAAGAGTCGGAAGCCTCCACACCACCTAGCACGCTGCCTTGCTTGTGCATCCATGCCATG
ACTCCAGAAAGAGTACAGCGCCTAAAAGCCTCACGTCCAGAACAAATTACTGTGTGCTCATCCTTTCCATTGCCGAAGTATAGAATCACTGGTGTGC
AGAAGATCCAATGCTCCCAGCCTATATTGTTCTCACCGAAAGTGCCTGCGTATATTCATCCAAGGAAGTATCTCGTGGAAACACCACCGGTAGACGA
GACTCCGGAGCCATCGGCAGAGAACCAATCCACAGAGGGGACACCTGAACAACCACCACTTATAACCGAGGATGAGACCAGGACTAGAACGCCTGAG
CCGATCATCATCGAAGAGGAAGAAGAGGATAGCATAAGTTTGCTGTCAGATGGCCCGACCCACCAGGTGCTGCAAGTCGAGGCAGACATTCACGGGC
CGCCCTCTGTATCTAGCTCATCCTGGTCCATTCCTCATGCATCCGACTTTGATGTGGACAGTTTATCCATACTTGACACCCTGGAGGGAGCTAGCGT
GACCAGCGGGGCAACGTCAGCCGAGACTAACTCTTACTTCGCAAAGAGTATGGAGTTTCTGGCGCGACCGGTGCCTGCGCCTCGAACAGTATTCAGG
AACCCTCCACATCCCGCTCCGCGCACAAGAACACCGTCACTTGCACCCAGCAGGGCCTGCTCGAGAACCAGCCTAGTTTCCACCCCGCCAGGCGTGA
ATAGGGTGATCACTAGAGAGGAGCTCGAGGCGCTTACCCCGTCACGCACTCCTAGCAGGTCGGTCTCGAGAACCAGCCTGGTCTCCAACCCGCCAGG
CGTAAATAGGGTGATTACAAGAGAGGAGTTTGAGGCGTTCGTAGCACAACAACAATGACGGTTTGATGCGGGTGCATACATCTTTTCCTCCGACACC
GGTCAAGGGCATTTACAACAAAAATCAGTAAGGCAAACGGTGCTATCCGAAGTGGTGTTGGAGAGGACCGAATTGGAGATTTCGTATGCCCCGCGCC
TCGACCAAGAAAAAGAAGAATTACTACGCAAGAAATTACAGTTAAATCCCACACCTGCTAACAGAAGCAGATACCAGTCCAGGAAGGTGGAGAACAT
GAAAGCCATAACAGCTAGACGTATTCTGCAAGGCCTAGGGCATTATTTGAAGGCAGAAGGAAAAGTGGAGTGCTACCGAACCCTGCATCCTGTTCCT
TTGTATTCATCTAGTGTGAACCGTGCCTTTTCAAGCCCCAAGGTCGCAGTGGAAGCCTGTAACGCCATGTTGAAAGAGAACTTTCCGACTGTGGCTT
CTTACTGTATTATTCCAGAGTACGATGCCTATTTGGACATGGTTGACGGAGCTTCATGCTGCTTAGACACTGCCAGTTTTTGCCCTGCAAAGCTGCG
CAGCTTTCCAAAGAAACACTCCTATTTGGAACCCACAATACGATCGGCAGTGCCTTCAGCGATCCAGAACACGCTCCAGAACGTCCTGGCAGCTGCC
ACAAAAAGAAATTGCAATGTCACGCAAATGAGAGAATTGCCCGTATTGGATTCGGCGGCCTTTAATGTGGAATGCTTCAAGAAATATGCGTGTAATA
ATGAATATTGGGAAACGTTTAAAGAAAACCCCATCAGGCTTACTGAAGAAAACGTGGTAAATTACATTACCAAATTAAAAGGACCAAAAGCTGCTGC
TCTTTTTGCGAAGACACATAATTTGAATATGTTGCAGGACATACCAATGGACAGGTTTGTAATGGACTTAAAGAGAGACGTGAAAGTGACTCCAGGA

ACAAAACATACTGAAGAACGGCCCAAGGTACAGGTGATCCAGGCTGCCGATCCGCTAGCAACAGCGTATCTGTGCGGAATCCACCGAGAGCTGGTTA
GGAGATTAAATGCGGTCCTGCTTCCGAACATTCATACACTGTTTGATATGTCGGCTGAAGACTTTGACGCTATTATAGCCGAGCACTTCCAGCCTGG
GGATTGTGTTCTGGAAACTGACATCGCGTCGTTTGATAAAAGTGAGGACGACGCCATGGCTCTGACCGCGTTAATGATTCTGGAAGACTTAGGTGTG
GACGCAGAGCTGTTGACGCTGATTGAGGCGGCTTTCGGCGAAATTTCATCAATACATTTGCCCACTAAAACTAAATTTAAATTCGGAGCCATGATGA
AATCTGGAATGTTCCTCACACTGTTTGTGAACACAGTCATTAACATTGTAATCGCAAGCAGAGTGTTGAGAGAACGGCTAACCGGATCACCATGTGC
AGCATTCATTGGAGATGACAATATCGTGAAAGGAGTCAAATCGGACAAATTAATGGCAGACAGGTGCGCCACCTGGTTGAATATGGAAGTCAAGATT
ATAGATGCTGTGGTGGGCGAGAAAGCGCCTTATTTCTGTGGAGGGTTTATTTTGTGTGACTCCGTGACCGGCACAGCGTGCCGTGTGGCAGACCCCC
TAAAAAGGCTGTTTAAGCTTGGCAAACCTCTGGCAGCAGACGATGAACATGATGATGACAGGAGAAGGGCATTGCATGAAGAGTCAACACGCTGGAA
CCGAGTGGGTATTCTTTCAGAGCTGTGCAAGGCAGTAGAATCAAGGTATGAAACCGTAGGAACTTCCATCATAGTTATGGCCATGACTACTCTAGCT
AGCAGTGTTAAATCATTCAGCTACCTGAGAGGGGCCCCTATAACTCTCTACGGCTAACCTGAATGGACTACGACATAGTCTAGTCCGCCAAGTCTAG
catatgggcgcgccctcagcatcgattgaattggccaccATGGTGAGCAAGGGCGAGGAGGATAACATGGCCATCATCAAGGAGTTCATGCGCTTCA
AGGTGCACATGGAGGGCTCCGTGAACGGCCACGAGTTCGAGATCGAGGGCGAGGGCGAGGGCCGCCCCTACGAGGGCACCCAGACCGCCAAGCTGAA
GGTGACCAAGGGTGGCCCCCTGCCCTTCGCCTGGGACATCCTGTCCCCTCAGTTCATGTACGGCTCCAAGGCCTACGTGAAGCACCCCGCCGACATC
CCCGACTACTTGAAGCTGTCCTTCCCCGAGGGCTTCAAGTGGGAGCGCGTGATGAACTTCGAGGACGGCGGCGTGGTGACCGTGACCCAGGACTCCT
CCCTGCAGGACGGCGAGTTCATCTACAAGGTGAAGCTGCGCGGCACCAACTTCCCCTCCGACGGCCCCGTAATGCAGAAGAAGACCATGGGCTGGGA
GGCCTCCTCCGAGCGGATGTACCCCGAGGACGGCGCCCTGAAGGGCGAGATCAAGCAGAGGCTGAAGCTGAAGGACGGCGGCCACTACGACGCTGAG
GTCAAGACCACCTACAAGGCCAAGAAGCCCGTGCAGCTGCCCGGCGCCTACAACGTCAACATCAAGTTGGACATCACCTCCCACAACGAGGACTACA
CCATCGTGGAACAGTACGAACGCGCCGAGGGCCGCCACTCCACCGGCGGCATGGACGAGCTGTACAAGTAGgaattGGCAAGCTGCTTACATAGAAC
TCGCGGCGATTGGCATGCCGCCTTAAAATTTTTATTTTATTTTTCTTTTCTTTTCCGAATCGGATTTTGTTTTTAATATTTCAAAAAAAAAAAAAAA
AAAAAAAAAAACGCGTCGAGGGGAATTAATTCTTGAAGACGAAAGGGCCAGGTGGCACTTTTCGGGGAAATGTGCGCGGAACCCCTATTTGTTTATT
TTTCTAAATACATTCAAATATGTATCCGCTCATGAGACAATAACCCTGATAAATGCTTCAATAATATTGAAAAAGGAAGAGTATGAGTATTCAACAT
TTCCGTGTCGCCCTTATTCCCTTTTTTGCGGCATTTTGCCTTCCTGTTTTTGCTCACCCAGAAACGCTGGTGAAAGTAAAAGATGCTGAAGATCAGT
TGGGTGCACGAGTGGGTTACATCGAACTGGATCTCAACAGCGGTAAGATCCTTGAGAGTTTTCGCCCCGAAGAACGTTTTCCAATGATGAGCACTTT
TAAAGTTCTGCTATGTGGCGCGGTATTATCCCGTGTTGACGCCGGGCAAGAGCAACTCGGTCGCCGCATACACTATTCTCAGAATGACTTGGTTGAG
TACTCACCAGTCACAGAAAAGCATCTTACGGATGGCATGACAGTAAGAGAATTATGCAGTGCTGCCATAACCATGAGTGATAACACTGCGGCCAACT
TACTTCTGACAACGATCGGAGGACCGAAGGAGCTAACCGCTTTTTTGCACAACATGGGGGATCATGTAACTCGCCTTGATCGTTGGGAACCGGAGCT
GAATGAAGCCATACCAAACGACGAGCGTGACACCACGATGCCTGTAGCAATGGCAACAACGTTGCGCAAACTATTAACTGGCGAACTACTTACTCTA
GCTTCCCGGCAACAATTAATAGACTGGATGGAGGCGGATAAAGTTGCAGGACCACTTCTGCGCTCGGCCCTTCCGGCTGGCTGGTTTATTGCTGATA
AATCTGGAGCCGGTGAGCGTGGGTCTCGCGGTATCATTGCAGCACTGGGGCCAGATGGTAAGCCCTCCCGTATCGTAGTTATCTACACGACGGGGAG
TCAGGCAACTATGGATGAACGAAATAGACAGATCGCTGAGATAGGTGCCTCACTGATTAAGCATTGGTAACTGTCAGACCAAGTTTACTCATATATA
CTTTAGATTGATTTAAAACTTCATTTTTAATTTAAAAGGATCTAGGTGAAGATCCTTTTTGATAATCTCATGACCAAAATCCCTTAACGTGAGTTTT
CGTTCCACTGAGCGTCAGACCCCGTAGAAAAGATCAAAGGATCTTCTTGAGATCCTTTTTTTCTGCGCGTAATCTGCTGCTTGCAAACAAAAAAACC
ACCGCTACCAGCGGTGGTTTGTTTGCCGGATCAAGAGCTACCAACTCTTTTTCCGAAGGTAACTGGCTTCAGCAGAGCGCAGATACCAAATACTGTC
CTTCTAGTGTAGCCGTAGTTAGGCCACCACTTCAAGAACTCTGTAGCACCGCCTACATACCTCGCTCTGCTAATCCTGTTACCAGTGGCTGCTGCCA
GTGGCGATAAGTCGTGTCTTACCGGGTTGGACTCAAGACGATAGTTACCGGATAAGGCGCAGCGGTCGGGCTGAACGGGGGGTTCGTGCACACAGCC
CAGCTTGGAGCGAACGACCTACACCGAACTGAGATACCTACAGCGTGAGCATTGAGAAAGCGCCACGCTTCCCGAAGGGAGAAAGGCGGACAGGTAT
CCGGTAAGCGGCAGGGTCGGAACAGGAGAGCGCACGAGGGAGCTTCCAGGGGGAAACGCCTGGTATCTTTATAGTCCTGTCGGGTTTCGCCACCTCT
GACTTGAGCGTCGATTTTTGTGATGCTCGTCAGGGGGGCGGAGCCTATGGAAAAACGCCAGCAACGCGAGCTCTAATACGACTCACTATAG

>aBc1-mCherry
ATGGGCGGCGCATGAGAGAAGCCCAGACCAATTACCTACCCAAAATGGAGAAAGTTCACGTTGACATCGAGGAAGACAGCCCATTCCTCAGAGCTTT
GCAGCGGAGCTTCCCGCAGTTTGAGGTAGAAGCCAAGCAGGTCACTGATAATGACCATGCTAATGCCAGAGCGTTTTCGCATCTGGCTTCAAAACTG
ATCGAAACGGAGGTGGACCCATCCGACACGATCCTTGACATTGGAAGTGCGCCCGCCCGCAGAATGTATTCTAAGCACAAGTATCATTGTATCTGTC
CGATGAGATGTGCGGAAGATCCGGACAGATTGTATAAGTATGCAACTAAGCTGAAGAAAAACTGTAAGGAAATAACTGATAAGGAATTGGACAAGAA
AATGAAGGAGCTGGCCGCCGTCATGAGCGACCCTGACCTGGAAACTGAGACTATGTGCCTCCACGACGACGAGTCGTGTCGCTACGAAGGGCAAGTC
GCTGTTTACCAGGATGTATACGCGGTTGACGGACCGACAAGTCTCTATCACCAAGCCAATAAGGGAGTTAGAGTCGCCTACTGGATAGGCTTTGACA
CCACCCCTTTTATGTTTAAGAACTTGGCTGGAGCATATCCATCATACTCTACCAACTGGGCCGACGAAACCGTGTTAACGGCTCGTAACATAGGCCT
ATGCAGCTCTGACGTTATGGAGCGGTCACGTAGAGGGATGTCCATTCTTAGAAAGAAGTATTTGAAACCATCCAACAATGTTCTATTCTCTGTTGGC
TCGACCATCTACCACGAGAAGAGGGACTTACTGAGGAGCTGGCACCTGCCGTCTGTATTTCACTTACGTGGCAAGCAAAATTACACATGTCGGTGTG
AGACTATAGTTAGTTGCGACGGGTACGTCGTTAAAAGAATAGCTATCAGTCCAGGCCTGTATGGGAAGCCTTCAGGCTATGCTGCTACGATGCACCG
CGAGGGATTCTTGTGCTGCAAAGTGACAGACACATTGAACGGGGAGAGGGTCTCTTTTCCCGTGTGCACGTATGTGCCAGCTACATTGTGTGACCAA
ATGACTGGCATACTGGCAACAGATGTCAGTGCGGACGACGCGCAAAAACTGCTGGTTGGGCTCAACCAGCGTATAGTCGTCAACGGTCGCACCCAGA
GAAACACCAATACCATGAAAAATTACCTTTTGCCCGTAGTGGCCCAGGCATTTGCTAGGTGGGCAAAGGAATATAAGGAAGATCAAGAAGATGAAAG
GCCACTAGGACTACGAGATAGACAGTTAGTCATGGGGTGTTGTTGGGCTTTTAGAAGGCACAAGATAACATCTATTTATAAGCGCCCGGATACCCAA
ACCATCATCAAAGTGAACAGCGATTTCCACTCATTCGTGCTGCCCAGGATAGGCAGTAACACATTGGAGATCGGGCTGAGAACAAGAATCAGGAAAA
TGTTAGAGGAGCACAAGGAGCCGTCACCTCTCATTACCGCCGAGGACGTACAAGAAGCTAAGTGCGCAGCCGATGAGGCTAAGGAGGTGCGTGAAGC
CGAGGAGTTGCGCGCAGCTCTACCACCTTTGGCAGCTGATGTTGAGGAGCCCACTCTGGAGGCAGACGTCGACTTGATGTTACAAGAGGCTGGGGCC
GGCTCAGTGGAGACACCTCGTGGCTTGATAAAGGTTACCAGCTACGATGGCGAGGACAAGATCGGCTCTTACGCTGTGCTTTCTCCGCAGGCTGTAC
TCAAGAGTGAAAAATTATCTTGCATCCACCCTCTCGCTGAACAAGTCATAGTGATAACACACTCTGGCCGAAAAGGGCGTTATGCCGTGGAACCATA
CCATGGTAAAGTAGTGGTGCCAGAGGGACATGCAATACCCGTCCAGGACTTTCAAGCTCTGAGTGAAAGTGCCACCATTGTGTACAACGAACGTGAG
TTCGTAAACAGGTACCTGCACCATATTGCCACACATGGBGGAGCGCTGAACACTGATGAAGAATATTACAAAACTGTCAAGCCCAGCGAGCACGACG
GCGAATACCTGTACGACATCGACAGGAAACAGTGCGTCAAGAAAGAACTAGTCACTGGGCTAGGGCTCACAGGCGAGCTGGTGGATCCTCCCTTCCA
TGAATTCGCCTACGAGAGTCTGAGAACACGACCAGCCGCTCCTTACCAAGTACCAACCATAGGGGTGTATGGCGTGCCAGGATCAGGCAAGTCTGGC
ATCATTAAAAGCGCAGTCACCAAAAAAGATCTAGTGGTGAGCGCCAAGAAAGAAAACTGTGCAGAAATTATAAGGGACGTCAAGAAAATGAAAGGGC
TGGACGTCAATGCCAGAACTGTGGACTCAGTGCTCTTGAATGGATGCAAACACCCCGTAGAGACCCTGTATATTGACGAAGCTTTTGCTTGTCATGC
AGGTACTCTCAGAGCGCTCATAGCCATTATAAGACCTAAAAAGGCAGTGCTCTGCGGGGATCCCAAACAGTGCGGTTTTTTTAACATGATGTGCCTG
AAAGTGCATTTTAACCACGAGATTTGCACACAAGTCTTCCACAAAAGCATCTCTCGCCGTTGCACTAAATCTGTGACTTCGGTCGTCTCAACCTTGT
TTTACGACAAAAAAATGAGAACGACGAATCCGAAAGAGACTAAGATTGTGATTGACACTACCGGCAGTACCAAACCTAAGCAGGACGATCTCATTCT
CACTTGTTTCAGAGGGTGGGTGAAGCAGTTGCAAATAGATTACAAAGGCAACGAAATAATGACGGCAGCTGCCTCTCAAGGGCTGACCCGTAAAGGT
GTGTATGCCGTTCGGTACAAGGTGAATGAAAATCCTCTGTACGCACCCACCTCAGAACATGTGAACGTCCTACTGACCCGCACGGAGGACCGCATCG
TGTGGAAAACACTAGCCGGCGACCCATGGATAAAAACACTGACTGCCAAGTACCCTGGGAATTTCACTGCCACGATAGAGGAGTGGCAAGCAGAGCA
TGATGCCATCATGAGGCACATCTTGGAGAGACCGGACCCTACCGACGTCTTCCAGAATAAGGCAAACGTGTGTTGGGCCAAGGCTTTAGTGCCGGTG

CTGAAGACCGCTGGCATAGACATGACCACTGAACAATGGAACACTGTGGATTATTTTGAAACGGACAAAGCTCACTCAGCAGAGATAGTATTGAACC
AACTATGCGTGAGGTTCTTTGGACTCGATCTGGACTCCGGTCTATTTTCTGCACCCACTGTTCCGTTATCCATTAGGAATAATCACTGGGATAACTC
CCCGTCGCCTAACATGTACGGGCTGAATAAAGAAGTGGTCCGTCAGCTCTCTCGCAGGTACCCACAACTGCCTCGGGCAGTTGCCACTGGAAGAGTC
TATGACATGAACACTGGTACACTGCGCAATTATGATCCGCGCATAAACCTAGTACCTGTAAACAGAAGACTGCCTCATGCTTTAGTCCTCCACCATA
ATGAACACCCACAGAGTGACTTTTCTTCATTCGTCAGCAAATTGAAGGGCAGAACTGTCCTGGTGGTCGGGGAAAAGTTGTCCGTCCCAGGCAAAAT
GGTTGACTGGTTGTCAGACCGGCCTGAGGCTACCTTCAGAGCTCGGCTGGATTTAGGCATCCCAGGTGATGTGCCCAAATATGACATAATATTTGTT
AATGTGAGGACCCCATATAAATACCATCACTATCAGCAGTGTGAAGACCATGCCATTAAGCTTAGCATGTTGACCAAGAAAGCTTGTCTGCATCTGA
ATCCCGGCGGAACCTGTGTCAGCATAGGTTATGGTTACGCTGACAGGGCCAGCGAAAGCATCATTGGTGCTATAGCGCGGCAGTTCAAGTTTTCCCG
GGTATGCAAACCGAAATCCTCACTTGAAGAGACGGAAGTTCTGTTTGTATTCATTGGGTACGATCGCAAGGCCCGTACGCACAATTCTTACAAGCTT
TCATCAACCTTGACCAACATTTATACAGGTTCCAGACTCCACGAAGCCGGATGTGCACCCTCATATCATGTGGTGCGAGGGGATATTGCCACGGCCA
CCGAAGGAGTGATTATAAATGCTGCTAACAGCAAAGGACAACCTGGCGGAGGGGTGTGCGGAGCGCTGTATAAGAAATTCCCGGAAAGCTTCGATTT
ACAGCCGATCGAAGTAGGAAAAGCGCGACTGGTCAAAGGTGCAGCTAAACATATCATTCATGCCGTAGGACCAAACTTCAACAAAGTTTCGGAGGTT
GAAGGTGACAAACAGTTGGCAGAGGCTTATGAGTCCATCGCTAAGATTGTCAACGATAACAATTACAAGTCAGTAGCGATTCCACTGTTGTCCACCG
GCATCTTTTCCGGGAACAAAGATCGACTAACCCAATCATTGAACCATTTGCTGACAGCTTTAGACACCACTGATGCAGATGTAGCCATATACTGCAG
GGACAAGAAATGGGAAATGACTCTCAAGGAAGCAGTGGCTAGGAGAGAAGCAGTGGAGGAGATATGCATATCCGACGACTCTTCAGTGACAGAACCT
GATGCAGAGCTGGTGAGGGTGCATCCGAAGAGTTCTTTGGCTGGAAGGAAGGGCTACAGCACAAGCGATGGCAAAACTTTCTCATATTTGGAAGGGA
CCAAGTTTCACCAGGCGGCCAAGGATATAGCAGAAATTAATGCCATGTGGCCCGTTGCAACGGAGGCCAATGAGCAGGTATGCATGTATATCCTCGG
AGAAGGCATGAGCAGTATTAGGTCGAAATGCCCCGTCGAAGAGTCGGAAGCCTCCACACCACCTAGCACGCTGCCTTGCTTGTGCATCCATGCCATG
ACTCCAGAAAGAGTACAGCGCCTAAAAGCCTCACGTCCAGAACAAATTACTGTGTGCTCATCCTTTCCATTGCCGAAGTATAGAATCACTGGTGTGC
AGAAGATCCAATGCTCCCAGCCTATATTGTTCTCACCGAAAGTGCCTGCGTATATTCATCCAAGGAAGTATCTCGTGGAAACACCACCGGTAGACGA
GACTCCGGAGCCATCGGCAGAGAACCAATCCACAGAGGGGACACCTGAACAACCACCACTTATAACCGAGGATGAGACCAGGACTAGAACGCCTGAG
CCGATCATCATCGAAGAGGAAGAAGAGGATAGCATAAGTTTGCTGTCAGATGGCCCGACCCACCAGGTGCTGCAAGTCGAGGCAGACATTCACGGGC
CGCCCTCTGTATCTAGCTCATCCTGGTCCATTCCTCATGCATCCGACTTTGATGTGGACAGTTTATCCATACTTGACACCCTGGAGGGAGCTAGCGT
GACCAGCGGGGCAACGTCAGCCGAGACTAACTCTTACTTCGCAAAGAGTATGGAGTTTCTGGCGCGACCGGTGCCTGCGCCTCGAACAGTATTCAGG
AACCCTCCACATCCCGCTCCGCGCACAAGAACACCGTCACTTGCACCCAGCAGGGCCTGCTCGAGAACCAGCCTAGTTTCCACCCCGCCAGGCGTGA
ATAGGGTGATCACTAGAGAGGAGCTCGAGGCGCTTACCCCGTCACGCACTCCTAGCAGGTCGGTCTCGAGAACCAGCCTGGTCTCCAACCCGCCAGG
CGTAAATAGGGTGATTACAAGAGAGGAGTTTGAGGCGTTCGTAGCACAACAACAATGACGGTTTGATGCGGGTGCATACATCTTTTCCTCCGACACC
GGTCAAGGGCATTTACAACAAAAATCAGTAAGGCAAACGGTGCTATCCGAAGTGGTGTTGGAGAGGACCGAATTGGAGATTTCGTATGCCCCGCGCC
TCGACCAAGAAAAAGAAGAATTACTACGCAAGAAATTACAGTTAAATCCCACACCTGCTAACAGAAGCAGATACCAGTCCAGGAAGGTGGAGAACAT
GAAAGCCATAACAGCTAGACGTATTCTGCAAGGCCTAGGGCATTATTTGAAGGCAGAAGGAAAAGTGGAGTGCTACCGAACCCTGCATCCTGTTCCT
TTGTATTCATCTAGTGTGAACCGTGCCTTTTCAAGCCCCAAGGTCGCAGTGGAAGCCTGTAACGCCATGTTGAAAGAGAACTTTCCGACTGTGGCTT
CTTACTGTATTATTCCAGAGTACGATGCCTATTTGGACATGGTTGACGGAGCTTCATGCTGCTTAGACACTGCCAGTTTTTGCCCTGCAAAGCTGCG
CAGCTTTCCAAAGAAACACTCCTATTTGGAACCCACAATACGATCGGCAGTGCCTTCAGCGATCCAGAACACGCTCCAGAACGTCCTGGCAGCTGCC
ACAAAAAGAAATTGCAATGTCACGCAAATGAGAGAATTGCCCGTATTGGATTCGGCGGCCTTTAATGTGGAATGCTTCAAGAAATATGCGTGTAATA
ATGAATATTGGGAAACGTTTAAAGAAAACCCCATCAGGCTTACTGAAGAAAACGTGGTAAATTACATTACCAAATTAAAAGGACCAAAAGCTGCTGC
TCTTTTTGCGAAGACACATAATTTGAATATGTTGCAGGACATACCAATGGACAGGTTTGTAATGGACTTAAAGAGAGACGTGAAAGTGACTCCAGGA
ACAAAACATACTGAAGAACGGCCCAAGGTACAGGTGATCCAGGCTGCCGATCCGCTAGCAACAGCGTATCTGTGCGGAATCCACCGAGAGCTGGTTA
GGAGATTAAATGCGGTCCTGCTTCCGAACATTCATACACTGTTTGATATGTCGGCTGAAGACTTTGACGCTATTATAGCCGAGCACTTCCAGCCTGG
GGATTGTGTTCTGGAAACTGACATCGCGTCGTTTGATAAAAGTGAGGACGACGCCATGGCTCTGACCGCGTTAATGATTCTGGAAGACTTAGGTGTG
GACGCAGAGCTGTTGACGCTGATTGAGGCGGCTTTCGGCGAAATTTCATCAATACATTTGCCCACTAAAACTAAATTTAAATTCGGAGCCATGATGA
AATCTGGAATGTTCCTCACACTGTTTGTGAACACAGTCATTAACATTGTAATCGCAAGCAGAGTGTTGAGAGAACGGCTAACCGGATCACCATGTGC
AGCATTCATTGGAGATGACAATATCGTGAAAGGAGTCAAATCGGACAAATTAATGGCAGACAGGTGCGCCACCTGGTTGAATATGGAAGTCAAGATT
ATAGATGCTGTGGTGGGCGAGAAAGCGCCTTATTTCTGTGGAGGGTTTATTTTGTGTGACTCCGTGACCGGCACAGCGTGCCGTGTGGCAGACCCCC
TAAAAAGGCTGTTTAAGCTTGGCAAACCTCTGGCAGCAGACGATGAACATGATGATGACAGGAGAAGGGCATTGCATGAAGAGTCAACACGCTGGAA
CCGAGTGGGTATTCTTTCAGAGCTGTGCAAGGCAGTAGAATCAAGGTATGAAACCGTAGGAACTTCCATCATAGTTATGGCCATGACTACTCTAGCT
AGCAGTGTTAAATCATTCAGCTACCTGAGAGGGGCCCCTATAACTCTCTACGGCTAACCTGAATGGACTACGACATAGTCTAGTCCGCCAAGTCTAG
catatgggcgcgccctcagcatcgattgaattggccaccATGGTGAGCAAGGGCGAGGAGGATAACATGGCCATCATCAAGGAGTTCATGCGCTTCA
AGGTGCACATGGAGGGCTCCGTGAACGGCCACGAGTTCGAGATCGAGGGCGAGGGCGAGGGCCGCCCCTACGAGGGCACCCAGACCGCCAAGCTGAA
GGTGACCAAGGGTGGCCCCCTGCCCTTCGCCTGGGACATCCTGTCCCCTCAGTTCATGTACGGCTCCAAGGCCTACGTGAAGCACCCCGCCGACATC
CCCGACTACTTGAAGCTGTCCTTCCCCGAGGGCTTCAAGTGGGAGCGCGTGATGAACTTCGAGGACGGCGGCGTGGTGACCGTGACCCAGGACTCCT
CCCTGCAGGACGGCGAGTTCATCTACAAGGTGAAGCTGCGCGGCACCAACTTCCCCTCCGACGGCCCCGTAATGCAGAAGAAGACCATGGGCTGGGA
GGCCTCCTCCGAGCGGATGTACCCCGAGGACGGCGCCCTGAAGGGCGAGATCAAGCAGAGGCTGAAGCTGAAGGACGGCGGCCACTACGACGCTGAG
GTCAAGACCACCTACAAGGCCAAGAAGCCCGTGCAGCTGCCCGGCGCCTACAACGTCAACATCAAGTTGGACATCACCTCCCACAACGAGGACTACA
CCATCGTGGAACAGTACGAACGCGCCGAGGGCCGCCACTCCACCGGCGGCATGGACGAGCTGTACAAGTAGgaattGGCAAGCTGCTTACATAGAAC
TCGCGGCGATTGGCATGCCGCCTTAAAATTTTTATTTTATTTTTCTTTTCTTTTCCGAATCGGATTTTGTTTTTAATATTTCAAAAAAAAAAAAAAA
AAAAAAAAAAACGCGTCGAGGGGAATTAATTCTTGAAGACGAAAGGGCCAGGTGGCACTTTTCGGGGAAATGTGCGCGGAACCCCTATTTGTTTATT
TTTCTAAATACATTCAAATATGTATCCGCTCATGAGACAATAACCCTGATAAATGCTTCAATAATATTGAAAAAGGAAGAGTATGAGTATTCAACAT
TTCCGTGTCGCCCTTATTCCCTTTTTTGCGGCATTTTGCCTTCCTGTTTTTGCTCACCCAGAAACGCTGGTGAAAGTAAAAGATGCTGAAGATCAGT
TGGGTGCACGAGTGGGTTACATCGAACTGGATCTCAACAGCGGTAAGATCCTTGAGAGTTTTCGCCCCGAAGAACGTTTTCCAATGATGAGCACTTT
TAAAGTTCTGCTATGTGGCGCGGTATTATCCCGTGTTGACGCCGGGCAAGAGCAACTCGGTCGCCGCATACACTATTCTCAGAATGACTTGGTTGAG
TACTCACCAGTCACAGAAAAGCATCTTACGGATGGCATGACAGTAAGAGAATTATGCAGTGCTGCCATAACCATGAGTGATAACACTGCGGCCAACT
TACTTCTGACAACGATCGGAGGACCGAAGGAGCTAACCGCTTTTTTGCACAACATGGGGGATCATGTAACTCGCCTTGATCGTTGGGAACCGGAGCT
GAATGAAGCCATACCAAACGACGAGCGTGACACCACGATGCCTGTAGCAATGGCAACAACGTTGCGCAAACTATTAACTGGCGAACTACTTACTCTA
GCTTCCCGGCAACAATTAATAGACTGGATGGAGGCGGATAAAGTTGCAGGACCACTTCTGCGCTCGGCCCTTCCGGCTGGCTGGTTTATTGCTGATA
AATCTGGAGCCGGTGAGCGTGGGTCTCGCGGTATCATTGCAGCACTGGGGCCAGATGGTAAGCCCTCCCGTATCGTAGTTATCTACACGACGGGGAG
TCAGGCAACTATGGATGAACGAAATAGACAGATCGCTGAGATAGGTGCCTCACTGATTAAGCATTGGTAACTGTCAGACCAAGTTTACTCATATATA
CTTTAGATTGATTTAAAACTTCATTTTTAATTTAAAAGGATCTAGGTGAAGATCCTTTTTGATAATCTCATGACCAAAATCCCTTAACGTGAGTTTT
CGTTCCACTGAGCGTCAGACCCCGTAGAAAAGATCAAAGGATCTTCTTGAGATCCTTTTTTTCTGCGCGTAATCTGCTGCTTGCAAACAAAAAAACC
ACCGCTACCAGCGGTGGTTTGTTTGCCGGATCAAGAGCTACCAACTCTTTTTCCGAAGGTAACTGGCTTCAGCAGAGCGCAGATACCAAATACTGTC
CTTCTAGTGTAGCCGTAGTTAGGCCACCACTTCAAGAACTCTGTAGCACCGCCTACATACCTCGCTCTGCTAATCCTGTTACCAGTGGCTGCTGCCA
GTGGCGATAAGTCGTGTCTTACCGGGTTGGACTCAAGACGATAGTTACCGGATAAGGCGCAGCGGTCGGGCTGAACGGGGGGTTCGTGCACACAGCC
CAGCTTGGAGCGAACGACCTACACCGAACTGAGATACCTACAGCGTGAGCATTGAGAAAGCGCCACGCTTCCCGAAGGGAGAAAGGCGGACAGGTAT

CCGGTAAGCGGCAGGGTCGGAACAGGAGAGCGCACGAGGGAGCTTCCAGGGGGAAACGCCTGGTATCTTTATAGTCCTGTCGGGTTTCGCCACCTCT
GACTTGAGCGTCGATTTTTGTGATGCTCGTCAGGGGGGCGGAGCCTATGGAAAAACGCCAGCAACGCGAGCTCTAATACGACTCACTATAG

>Ab1c1-mCherry
ATGGGCGGCGCATGAGAGAAGCCCAGACCAATTACCTACCCAAAATGGAGAAAGTTCACGTTGACATCGAGGAAGACAGCCCATTCCTCAGAGCTTT
GCAGCGGAGCTTCCCGCAGTTTGAGGTAGAAGCCAAGCAGGTCACTGATAATGACCATGCTAATGCCAGAGCGTTTTCGCATCTGGCTTCAAAACTG
ATCGAAACGGAGGTGGACCCATCCGACACGATCCTTGACATTGGAAGTGCGCCCGCCCGCAGAATGTATTCTAAGCACAAGTATCATTGTATCTGTC
CGATGAGATGTGCGGAAGATCCGGACAGATTGTATAAGTATGCAACTAAGCTGAAGAAAAACTGTAAGGAAATAACTGATAAGGAATTGGACAAGAA
AATGAAGGAGCTGGCCGCCGTCATGAGCGACCCTGACCTGGAAACTGAGACTATGTGCCTCCACGACGACGAGTCGTGTCGCTACGAAGGGCAAGTC
GCTGTTTACCAGGATGTATACGCGGTTGACGGACCGACAAGTCTCTATCACCAAGCCAATAAGGGAGTTAGAGTCGCCTACTGGATAGGCTTTGACA
CCACCCCTTTTATGTTTAAGAACTTGGCTGGAGCATATCCATCATACTCTACCAACTGGGCCGACGAAACCGTGTTAACGGCTCGTAACATAGGCCT
ATGCAGCTCTGACGTTATGGAGCGGTCACGTAGAGGGATGTCCATTCTTAGAAAGAAGTATTTGAAACCATCCAACAATGTTCTATTCTCTGTTGGC
TCGACCATCTACCACGAGAAGAGGGACTTACTGAGGAGCTGGCACCTGCCGTCTGTATTTCACTTACGTGGCAAGCAAAATTACACATGTCGGTGTG
AGACTATAGTTAGTTGCGACGGGTACGTCGTTAAAAGAATAGCTATCAGTCCAGGCCTGTATGGGAAGCCTTCAGGCTATGCTGCTACGATGCACCG
CGAGGGATTCTTGTGCTGCAAAGTGACAGACACATTGAACGGGGAGAGGGTCTCTTTTCCCGTGTGCACGTATGTGCCAGCTACATTGTGTGACCAA
ATGACTGGCATACTGGCAACAGATGTCAGTGCGGACGACGCGCAAAAACTGCTGGTTGGGCTCAACCAGCGTATAGTCGTCAACGGTCGCACCCAGA
GAAACACCAATACCATGAAAAATTACCTTTTGCCCGTAGTGGCCCAGGCATTTGCTAGGTGGGCAAAGGAATATAAGGAAGATCAAGAAGATGAAAG
GCCACTAGGACTACGAGATAGACAGTTAGTCATGGGGTGTTGTTGGGCTTTTAGAAGGCACAAGATAACATCTATTTATAAGCGCCCGGATACCCAA
ACCATCATCAAAGTGAACAGCGATTTCCACTCATTCGTGCTGCCCAGGATAGGCAGTAACACATTGGAGATCGGGCTGAGAACAAGAATCAGGAAAA
TGTTAGAGGAGCACAAGGAGCCGTCACCTCTCATTACCGCCGAGGACGTACAAGAAGCTAAGTGCGCAGCCGATGAGGCTAAGGAGGTGCGTGAAGC
CGAGGAGTTGCGCGCAGCTCTACCACCTTTGGCAGCTGATGTTGAGGAGCCCACTCTGGAGGCAGACGTCGACTTGATGTTACAAGAGGCTGGGGCC
GGCTCAGTGGAGACACCTCGTGGCTTGATAAAGGTTACCAGCTACGATGGCGAGGACAAGATCGGCTCTTACGCTGTGCTTTCTCCGCAGGCTGTAC
TCAAGAGTGAAAAATTATCTTGCATCCACCCTCTCGCTGAACAAGTCATAGTGATAACACACTCTGGCCGAAAAGGGCGTTATGCCGTGGAACCATA
CCATGGTAAAGTAGTGGTGCCAGAGGGACATGCAATACCCGTCCAGGACTTTCAAGCTCTGAGTGAAAGTGCCACCATTGTGTACAACGAACGTGAG
TTCGTAAACAGGTACCTGCACCATATTGCCACACATGGAGGAGCGCTGAACACTGATGAAGAATATTACAAAACTGTCAAGCCCAGCGAGCACGACG
GCGAATACCTGTACGACATCGACAGGAAACAGTGCGTCAAGAAAGAACTAGTCACTGGGCTAGGGCTCACAGGCGAGCTGGTGGATCCTCCCTTCCA
TGAATTCGCCTACGAGAGTCTGAGAACACGACCAGCCGCTCCTTACCAAGTACCAACCATAGGGGTGTATGGCGTGCCAGGATCAGGCAAGTCTGGC
ATCATTAAAAGCGCAGTCACCAAAAAAGATCTAGTGGTGAGCGCCAAGAAAGAAAACTGTGCAGAAATTATAAGGGACGTCAAGAAAATGAAAGGGC
TGGACGTCAATGCCAGAACTGTGGACTCAGTGCTCTTGAATGGATGCAAACACCCCGTAGAGACCCTGTATATTGACGAAGCTTTTGCTTGTCATGC
AGGTACTCTCAGAGCGCTCATAGCCATTATAAGACCTAAAAAGGCAGTGCTCTGCGGGGATCCCAAACAGTGCGGTTTTTTAACATGATGTGCCTG
AAAGTGCATTTTAACCACGAGATTTGCACACAAGTCTTCCACAAAAGCATCTCTCGCCGTTGCACTAAATCTGTGACTTCGGTCGTCTCAACCTTGT
TTTACGACAAAAAAATGAGAACGACGAATCCGAAAGAGACTAAGATTGTGATTGACACTACCGGCAGTACCAAACCTAAGCAGGACGATCTCATTCT
CACTTGTTTCAGAGGGTGGGTGAAGCAGTTGCAAATAGATTACAAAGGCAACGAAATAATGACGGCAGCTGCCTCTCAAGGGCTGACCCGTAAAGGT
GTGTATGCCGTTCGGTACAAGGTGAATGAAAATCCTCTGTACGCACCCACCTCAGAACATGTGAACGTCCTACTGACCCGCACGGAGGACCGCATCG
TGTGGAAAACACTAGCCGGCGACCCATGGATAAAAACACTGACTGCCAAGTACCCTGGGAATTTCACTGCCACGATAGAGGAGTGGCAAGCAGAGCA
TGATGCCATCATGAGGCACATCTTGGAGAGACCGGACCCTACCGACGTCTTCCAGAATAAGGCAAACGTGTGTTGGGCCAAGGCTTTAGTGCCGGTG
CTGAAGACCGCTGGCATAGACATGACCACTGAACAATGGAACACTGTGGATTATTTTGAAACGGACAAAGCTCACTCAGCAGAGATAGTATTGAACC
AACTATGCGTGAGGTTCTTTGGACTCGATCTGGACTCCGGTCTATTTTCTGCACCCACTGTTCCGTTATCCATTAGGAATAATCACTGGGATAACTC
CCCGTCGCCTAACATGTACGGGCTGAATAAAGAAGTGGTCCGTCAGCTCTCTCGCAGGTACCCACAACTGCCTCGGGCAGTTGCCACTGGAAGAGTC
TATGACATGAACACTGGTACACTGCGCAATTATGATCCGCGCATAAACCTAGTACCTGTAAACAGAAGACTGCCTCATGCTTTAGTCCTCCACCATA
ATGAACACCCACAGAGTGACTTTTCTTCATTCGTCAGCAAATTGAAGGGCAGAACTGTCCTGGTGGTCGGGGAAAAGTTGTCCGTCCCAGGCAAAAT
GGTTGACTGGTTGTCAGACCGGCCTGAGGCTACCTTCAGAGCTCGGCTGGATTTAGGCATCCCAGGTGATGTGCCCAAATATGACATAATATTTGTT
AATGTGAGGACCCCATATAAATACCATCACTATCAGCAGTGTGAAGACCATGCCATTAAGCTTAGCATGTTGACCAAGAAAGCTTGTCTGCATCTGA
ATCCCGGCGGAACCTGTGTCAGCATAGGTTATGGTTACGCTGACAGGGCCAGCGAAAGCATCATTGGTGCTATAGCGCGGCAGTTCAAGTTTTCCCG
GGTATGCAAACCGAAATCCTCACTTGAAGAGACGGAAGTTCTGTTTGTATTCATTCGGTACGATCGCAAGGCCCGTACGCACAATTCTTACAAGCTT
TCATCAACCTTGACCAACATTTATACAGGTTCCAGACTCCACGAAGCCGGATGTGCACCCTCATATCATGTGGTGCGAGGGGATATTGCCACGGCCA
CCGAAGGAGTGATTATAAATGCTGCTAACAGCAAAGGACAACCTGGCGGAGGGGTGTGCGGAGCGCTGTATAAGAAATTCCCGGAAAGCTTCGATTT
ACAGCCGATCGAAGTAGGAAAAGCGCGACTGGTCAAAGGTGCAGCTAAACATATCATTCATGCCGTAGGACCAAACTTCAACAAAGTTTCGGAGGTT
GAAGGTGACAAACAGTTGGCAGAGGCTTATGAGTCCATCGCTAAGATTGTCAACGATAACAATTACAAGTCAGTAGCGATTCCACTGTTGTCCACCG
GCATCTTTTCCGGGAACAAAGATCGACTAACCCAATCATTGAACCATTTGCTGACAGCTTTAGACACCACTGATGCAGATGTAGCCATATACTGCAG
GGACAAGAAATGGGAAATGACTCTCAAGGAAGCAGTGGCTAGGAGAGAAGCAGTGGAGGAGATATGCATATCCGACGACTCTTCAGTGACAGAACCT
GATGCAGAGCTGGTGAGGGTGCATCCGAAGAGTTCTTTGGCTGGAAGGAAGGGCTACAGCACAAGCGATGGCAAAACTTTCTCATATTTGGAAGGGA
CCAAGTTTCACCAGGCGGCCAAGGATATAGCAGAAATTAATGCCATGTGGCCCGTTGCAACGGAGGCCAATGAGCAGGTATGCATGTATATCCTCGG
AGAAGGCATGAGCAGTATTAGGTCGAAATGCCCCGTCGAAGAGTCGGAAGCCTCCACACCACCTAGCACGCTGCCTTGCTTGTGCATCCATGCCATG
ACTCCAGAAAGAGTACAGCGCCTAAAAGCCTCACGTCCAGAACAAATTACTGTGTGCTCATCCTTTCCATTGCCGAAGTATAGAATCACTGGTGTGC
AGAAGATCCAATGCTCCCAGCCTATATTGTTCTCACCGAAAGTGCCTGCGTATATTCATCCAAGGAAGTATCTCGTGGAAACACCACCGGTAGACGA
GACTCCGGAGCCATCGGCAGAGAACCAATCCACAGAGGGGACACCTGAACAACCACCACTTATAACCGAGGATGAGACCAGGACTAGAACGCCTGAG
CCGATCATCATCGAAGAGGAAGAAGAGGATAGCATAAGTTTGCTGTCAGATGGCCCGACCCACCAGGTGCTGCAAGTCGAGGCAGACATTCACGGGC
CGCCCTCTGTATCTAGCTCATCCTGGTCCATTCCTCATGCATCCGACTTTGATGTGGACAGTTTATCCATACTTGACACCCTGGAGGGAGCTAGCGT
GACCAGCGGGGCAACGTCAGCCGAGACTAACTCTTACTTCGCAAAGAGTATGGAGTTTCTGGCGCGACCGGTGCCTGCGCCTCGAACAGTATTCAGG
AACCCTCCACATCCCGCTCCGCGCACAAGAACACCGTCACTTGCACCCAGCAGGGCCTGCTCGAGAACCAGCCTAGTTTCCACCCCGCCAGGCGTGA
ATAGGGTGATCACTAGAGAGGAGCTCGAGGCGCTTACCCCGTCACGCACTCCTAGCAGGTCGGTCTCGAGAACCAGCCTGGTCTCCAACCCGCCAGG
CGTAAATAGGGTGATTACAAGAGAGGAGTTTGAGGCGTTCGTAGCACAACAACAATGACGGTTTGATGCGGGTGCATACATCTTTTCCTCCGACACC
GGTCAAGGGCATTTACAACAAAAATCAGTAAGGCAAACGGTGCTATCCGAAGTGGTGTTGGAGAGGACCGAATTGGAGATTTCGTATGCCCCGCGCC
TCGACCAAGAAAAAGAAGAATTACTACGCAAGAAATTACAGTTAAATCCCACACCTGCTAACAGAAGCAGATACCAGTCCAGGAAGGTGGAGAACAT
GAAAGCCATAACAGCTAGACGTATTCTGCAAGGCCTAGGGCATTATTTGAAGGCAGAAGGAAAAGTGGAGTGCTACCGAACCCTGCATCCTGTTCCT
TTGTATTCATCTAGTGTGAACCGTGCCTTTTCAAGCCCCAAGGTCGCAGTGGAAGCCTGTAACGCCATGTTGAAAGAGAACTTTCCGACTGTGGCTT
CTTACTGTATTATTCCAGAGTACGATGCCTATTTGGACATGGTTGACGGAGCTTCATGCTGCTTAGACACTGCCAGTTTTTGCCCTGCAAAGCTGCG
CAGCTTTCCAAAGAAACACTCCTATTTGGAACCCACAATACGATCGGCAGTGCCTTCAGCGATCCAGAACACGCTCCAGAACGTCCTGGCAGCTGCC
ACAAAAAGAAATTGCAATGTCACGCAAATGAGAGAATTGCCCGTATTGGATTCGGCGGCCTTTAATGTGGAATGCTTCAAGAAATATGCGTGTAATA
ATGAATATTGGGAAACGTTTAAAGAAAACCCCATCAGGCTTACTGAAGAAAACGTGGTAAATTACATTACCAAATTAAAAGGACCAAAAGCTGCTGC
TCTTTTTGCGAAGACACATAATTTGAATATGTTGCAGGACATACCAATGGACAGGTTTGTAATGGACTTAAAGAGAGACGTGAAAGTGACTCCAGGA

ACAAAACATACTGAAGAACGGCCCAAGGTACAGGTGATCCAGGCTGCCGATCCGCTAGCAACAGCGTATCTGTGCGGAATCCACCGAGAGCTGGTTA
GGAGATTAAATGCGGTCCTGCTTCCGAACATTCATACACTGTTTGATATGTCGGCTGAAGACTTTGACGCTATTATAGCCGAGCACTTCCAGCCTGG
GGATTGTGTTCTGGAAACTGACATCGCGTCGTTTGATAAAAGTGAGGACGACGCCATGGCTCTGACCGCGTTAATGATTCTGGAAGACTTAGGTGTG
GACGCAGAGCTGTTGACGCTGATTGAGGCGGCTTTCGGCGAAATTTCATCAATACATTTGCCCACTAAAACTAAATTTAAATTCGGAGCCATGATGA
AATCTGGAATGTTCCTCACACTGTTTGTGAACACAGTCATTAACATTGTAATCGCAAGCAGAGTGTTGAGAGAACGGCTAACCGGATCACCATGTGC
AGCATTCATTGGAGATGACAATATCGTGAAAGGAGTCAAATCGGACAAATTAATGGCAGACAGGTGCGCCACCTGGTTGAATATGGAAGTCAAGATT
ATAGATGCTGTGGTGGGCGAGAAAGCGCCTTATTTCTGTGGAGGGTTTATTTTGTGTGACTCCGTGACCGGCACAGCGTGCCGTGTGGCAGACCCCC
TAAAAAGGCTGTTTAAGCTTGGCAAACCTCTGGCAGCAGACGATGAACATGATGATGACAGGAGAAGGGCATTGCATGAAGAGTCAACACGCTGGAA
CCGAGTGGGTATTCTTTCAGAGCTGTGCAAGGCAGTAGAATCAAGGTATGAAACCGTAGGAACTTCCATCATAGTTATGGCCATGACTACTCTAGCT
AGCAGTGTTAAATCATTCAGCTACCTGAGAGGGGCCCCTATAACTCTCTACGGCTAACCTGAATGGACTACGACATAGTCTAGTCCGCCAAGTCTAG
catatgggcgcgccctcagcatcgattgaattggccaccATGGTGAGCAAGGGCGAGGAGGATAACATGGCCATCATCAAGGAGTTCATGCGCTTCA
AGGTGCACATGGAGGGCTCCGTGAACGGCCACGAGTTCGAGATCGAGGGCGAGGGCGAGGGCCGCCCCTACGAGGGCACCCAGACCGCCAAGCTGAA
GGTGACCAAGGGTGGCCCCCTGCCCTTCGCCTGGGACATCCTGTCCCCTCAGTTCATGTACGGCTCCAAGGCCTACGTGAAGCACCCCGCCGACATC
CCCGACTACTTGAAGCTGTCCTTCCCCGAGGGCTTCAAGTGGGAGCGCGTGATGAACTTCGAGGACGGCGGCGTGGTGACCGTGACCCAGGACTCCT
CCCTGCAGGACGGCGAGTTCATCTACAAGGTGAAGCTGCGCGGCACCAACTTCCCCTCCGACGGCCCCGTAATGCAGAAGAAGACCATGGGCTGGGA
GGCCTCCTCCGAGCGGATGTACCCCGAGGACGGCGCCCTGAAGGGCGAGATCAAGCAGAGGCTGAAGCTGAAGGACGGCGGCCACTACGACGCTGAG
GTCAAGACCACCTACAAGGCCAAGAAGCCCGTGCAGCTGCCCGGCGCCTACAACGTCAACATCAAGTTGGACATCACCTCCCACAACGAGGACTACA
CCATCGTGGAACAGTACGAACGCGCCGAGGGCCGCCACTCCACCGGCGGCATGGACGAGCTGTACAAGTAGgaattGGCAAGCTGCTTACATAGAAC
TCGCGGCGATTGGCATGCCGCCTTAAAATTTTTATTTTATTTTTCTTTTCTTTTCCGAATCGGATTTTGTTTTTAATATTTCAAAAAAAAAAAAAAA
AAAAAAAAAAACGCGTCGAGGGGAATTAATTCTTGAAGACGAAAGGGCCAGGTGGCACTTTTCGGGGAAATGTGCGCGGAACCCCTATTTGTTTATT
TTTCTAAATACATTCAAATATGTATCCGCTCATGAGACAATAACCCTGATAAATGCTTCAATAATATTGAAAAAGGAAGAGTATGAGTATTCAACAT
TTCCGTGTCGCCCTTATTCCCTTTTTTGCGGCATTTTGCCTTCCTGTTTTTGCTCACCCAGAAACGCTGGTGAAAGTAAAAGATGCTGAAGATCAGT
TGGGTGCACGAGTGGGTTACATCGAACTGGATCTCAACAGCGGTAAGATCCTTGAGAGTTTTCGCCCCGAAGAACGTTTTCCAATGATGAGCACTTT
TAAAGTTCTGCTATGTGGCGCGGTATTATCCCGTGTTGACGCCGGGCAAGAGCAACTCGGTCGCCGCATACACTATTCTCAGAATGACTTGGTTGAG
TACTCACCAGTCACAGAAAAGCATCTTACGGATGGCATGACAGTAAGAGAATTATGCAGTGCTGCCATAACCATGAGTGATAACACTGCGGCCAACT
TACTTCTGACAACGATCGGAGGACCGAAGGAGCTAACCGCTTTTTTGCACAACATGGGGGATCATGTAACTCGCCTTGATCGTTGGGAACCGGAGCT
GAATGAAGCCATACCAAACGACGAGCGTGACACCACGATGCCTGTAGCAATGGCAACAACGTTGCGCAAACTATTAACTGGCGAACTACTTACTCTA
GCTTCCCGGCAACAATTAATAGACTGGATGGAGGCGGATAAAGTTGCAGGACCACTTCTGCGCTCGGCCCTTCCGGCTGGCTGGTTTATTGCTGATA
AATCTGGAGCCGGTGAGCGTGGGTCTCGCGGTATCATTGCAGCACTGGGGCCAGATGGTAAGCCCTCCCGTATCGTAGTTATCTACACGACGGGGAG
TCAGGCAACTATGGATGAACGAAATAGACAGATCGCTGAGATAGGTGCCTCACTGATTAAGCATTGGTAACTGTCAGACCAAGTTTACTCATATATA
CTTTAGATTGATTTAAAACTTCATTTTTAATTTAAAAGGATCTAGGTGAAGATCCTTTTTGATAATCTCATGACCAAAATCCCTTAACGTGAGTTTT
CGTTCCACTGAGCGTCAGACCCCGTAGAAAAGATCAAAGGATCTTCTTGAGATCCTTTTTTTCTGCGCGTAATCTGCTGCTTGCAAACAAAAAAACC
ACCGCTACCAGCGGTGGTTTGTTTGCCGGATCAAGAGCTACCAACTCTTTTTCCGAAGGTAACTGGCTTCAGCAGAGCGCAGATACCAAATACTGTC
CTTCTAGTGTAGCCGTAGTTAGGCCACCACTTCAAGAACTCTGTAGCACCGCCTACATACCTCGCTCTGCTAATCCTGTTACCAGTGGCTGCTGCCA
GTGGCGATAAGTCGTGTCTTACCGGGTTGGACTCAAGACGATAGTTACCGGATAAGGCGCAGCGGTCGGGCTGAACGGGGGGTTCGTGCACACAGCC
CAGCTTGGAGCGAACGACCTACACCGAACTGAGATACCTACAGCGTGAGCATTGAGAAAGCGCCACGCTTCCCGAAGGGAGAAAGGCGGACAGGTAT
CCGGTAAGCGGCAGGGTCGGAACAGGAGAGCGCACGAGGGAGCTTCCAGGGGGAAACGCCTGGTATCTTTATAGTCCTGTCGGGTTTCGCCACCTCT
GACTTGAGCGTCGATTTTTGTGATGCTCGTCAGGGGGGCGGAGCCTATGGAAAAACGCCAGCAACGCGAGCTCTAATACGACTCACTATAG

>ab1c1-mCherry
ATGGGCGGCGCATGAGAGAAGCCCAGACCAATTACCTACCCAAAATGGAGAAAGTTCACGTTGACATCGAGGAAGACAGCCCATTCCTCAGAGCTTT
GCAGCGGAGCTTCCCGCAGTTTGAGGTAGAAGCCAAGCAGGTCACTGATAATGACCATGCTAATGCCAGAGCGTTTTCGCATCTGGCTTCAAAACTG
ATCGAAACGGAGGTGGACCCATCCGACACGATCCTTGACATTGGAAGTGCGCCCGCCCGCAGAATGTATTCTAAGCACAAGTATCATTGTATCTGTC
CGATGAGATGTGCGGAAGATCCGGACAGATTGTATAAGTATGCAACTAAGCTGAAGAAAAACTGTAAGGAAATAACTGATAAGGAATTGGACAAGAA
AATGAAGGAGCTGGCCGCCGTCATGAGCGACCCTGACCTGGAAACTGAGACTATGTGCCTCCACGACGACGAGTCGTGTCGCTACGAAGGGCAAGTC
GCTGTTTACCAGGATGTATACGCGGTTGACGGACCGACAAGTCTCTATCACCAAGCCAATAAGGGAGTTAGAGTCGCCTACTGGATAGGCTTTGACA
CCACCCCTTTTATGTTTAAGAACTTGGCTGGAGCATATCCATCATACTCTACCAACTGGGCCGACGAAACCGTGTTAACGGCTCGTAACATAGGCCT
ATGCAGCTCTGACGTTATGGAGCGGTCACGTAGAGGGATGTCCATTCTTAGAAAGAAGTATTTGAAACCATCCAACAATGTTCTATTCTCTGTTGGC
TCGACCATCTACCACGAGAAGAGGGACTTACTGAGGAGCTGGCACCTGCCGTCTGTATTTCACTTACGTGGCAAGCAAAATTACACATGTCGGTGTG
AGACTATAGTTAGTTGCGACGGGTACGTCGTTAAAAGAATAGCTATCAGTCCAGGCCTGTATGGGAAGCCTTCAGGCTATGCTGCTACGATGCACCG
CGAGGGATTCTTGTGCTGCAAAGTGACAGACACATTGAACGGGGAGAGGGTCTCTTTTCCCGTGTGCACGTATGTGCCAGCTACATTGTGTGACCAA
ATGACTGGCATACTGGCAACAGATGTCAGTGCGGACGACGCGCAAAAACTGCTGGTTGGGCTCAACCAGCGTATAGTCGTCAACGGTCGCACCCAGA
GAAACACCAATACCATGAAAAATTACCTTTTGCCCGTAGTGGCCCAGGCATTTGCTAGGTGGGCAAAGGAATATAAGGAAGATCAAGAAGATGAAAG
GCCACTAGGACTACGAGATAGACAGTTAGTCATGGGGTGTTGTTGGGCTTTTAGAAGGCACAAGATAACATCTATTTATAAGCGCCCGGATACCCAA
ACCATCATCAAAGTGAACAGCGATTTCCACTCATTCGTGCTGCCCAGGATAGGCAGTAACACATTGGAGATCGGGCTGAGAACAAGAATCAGGAAAA
TGTTAGAGGAGCACAAGGAGCCGTCACCTCTCATTACCGCCGAGGACGTACAAGAAGCTAAGTGCGCAGCCGATGAGGCTAAGGAGGTGCGTGAAGC
CGAGGAGTTGCGCGCAGCTCTACCACCTTTGGCAGCTGATGTTGAGGAGCCCACTCTGGAGGCAGACGTCGACTTGATGTTACAAGAGGCTGGGGCC
GGCTCAGTGGAGACACCTCGTGGCTTGATAAAGGTTACCAGCTACGATGGCGAGGACAAGATCGGCTCTTACGCTGTGCTTTCTCCGCAGGCTGTAC
TCAAGAGTGAAAAATTATCTTGCATCCACCCTCTCGCTGAACAAGTCATAGTGATAACACACTCTGGCCGAAAAGGGCGTTATGCCGTGGAACCATA
CCATGGTAAAGTAGTGGTGCCAGAGGGACATGCAATACCCGTCCAGGACTTTCAAGCTCTGAGTGAAAGTGCCACCATTGTGTACAACGAACGTGAG
TTCGTAAACAGGTACCTGCACCATATTGCCACACATGGGGGAGCGCTGAACACTGATGAAGAATATTACAAAACTGTCAAGCCCAGCGAGCACGACG
GCGAATACCTGTACGACATCGACAGGAAACAGTGCGTCAAGAAAGAACTAGTCACTGGGCTAGGGCTCACAGGCGAGCTGGTGGATCCTCCCTTCCA
TGAATTCGCCTACGAGAGTCTGAGAACACGACCAGCCGCTCCTTACCAAGTACCAACCATAGGGGTGTATGGCGTGCCAGGATCAGGCAAGTCTGGC
ATCATTAAAAGCGCAGTCACCAAAAAAGATCTAGTGGTGAGCGCCAAGAAAGAAAACTGTGCAGAAATTATAAGGGACGTCAAGAAAATGAAAGGGC
TGGACGTCAATGCCAGAACTGTGGACTCAGTGCTCTTGAATGGATGCAAACACCCCGTAGAGACCCTGTATATTGACGAAGCTTTTGCTTGTCATGC
AGGTACTCTCAGAGCGCTCATAGCCATTATAAGACCTAAAAAGGCAGTGCTCTGCGGGGATCCCAAACAGTGCGGTTTTTTTAACATGATGTGCCTG
AAAGTGCATTTTAACCACGAGATTTGCACACAAGTCTTCCACAAAAGCATCTCTCGCCGTTGCACTAAATCTGTGACTTCGGTCGTCTCAACCTTGT
TTTACGACAAAAAAATGAGAACGACGAATCCGAAAGAGACTAAGATTGTGATTGACACTACCGGCAGTACCAAACCTAAGCAGGACGATCTCATTCT
CACTTGTTTCAGAGGGTGGGTGAAGCAGTTGCAAATAGATTACAAAGGCAACGAAATAATGACGGCAGCTGCCTCTCAAGGGCTGACCCGTAAAGGT
GTGTATGCCGTTCGGTACAAGGTGAATGAAAATCCTCTGTACGCACCCACCTCAGAACATGTGAACGTCCTACTGACCCGCACGGAGGACCGCATCG
TGTGGAAAACACTAGCCGGCGACCCATGGATAAAAACACTGACTGCCAAGTACCCTGGGAATTTCACTGCCACGATAGAGGAGTGGCAAGCAGAGCA
TGATGCCATCATGAGGCACATCTTGGAGAGACCGGACCCTACCGACGTCTTCCAGAATAAGGCAAACGTGTGTTGGGCCAAGGCTTTAGTGCCGGTG

CTGAAGACCGCTGGCATAGACATGACCACTGAACAATGGAACACTGTGGATTATTTTGAAACGGACAAAGCTCACTCAGCAGAGATAGTATTGAACC
AACTATGCGTGAGGTTCTTTGGACTCGATCTGGACTCCGGTCTATTTTCTGCACCCACTGTTCCGTTATCCATTAGGAATAATCACTGGGATAACTC
CCCGTCGCCTAACATGTACGGGCTGAATAAAGAAGTGGTCCGTCAGCTCTCTCGCAGGTACCCACAACTGCCTCGGGCAGTTGCCACTGGAAGAGTC
TATGACATGAACACTGGTACACTGCGCAATTATGATCCGCGCATAAACCTAGTACCTGTAAACAGAAGACTGCCTCATGCTTTAGTCCTCCACCATA
ATGAACACCCACAGAGTGACTTTTCTTCATTCGTCAGCAAATTGAAGGGCAGAACTGTCCTGGTGGTCGGGGAAAAGTTGTCCGTCCCAGGCAAAAT
GGTTGACTGGTTGTCAGACCGGCCTGAGGCTACCTTCAGAGCTCGGCTGGATTTAGGCATCCCAGGTGATGTGCCCAAATATGACATAATATTTGTT
AATGTGAGGACCCCATATAAATACCATCACTATCAGCAGTGTGAAGACCATGCCATTAAGCTTAGCATGTTGACCAAGAAAGCTTGTCTGCATCTGA
ATCCCGGCGGAACCTGTGTCAGCATAGGTTATGGTTACGCTGACAGGGCCAGCGAAAGCATCATTGGTGCTATAGCGCGGCAGTTCAAGTTTTCCCG
GGTATGCAAACCGAAATCCTCACTTGAAGAGACGGAAGTTCTGTTTGTATTCATTCGGTACGATCGCAAGGCCCGTACGCACAATTCTTACAAGCTT
TCATCAACCTTGACCAACATTTATACAGGTTCCAGACTCCACGAAGCCGGATGTGCACCCTCATATCATGTGGTGCGAGGGGATATTGCCACGGCCA
CCGAAGGAGTGATTATAAATGCTGCTAACAGCAAAGGACAACCTGGCGGAGGGGTGTGCGGAGCGCTGTATAAGAAATTCCCGGAAAGCTTCGATTT
ACAGCCGATCGAAGTAGGAAAAGCGCGACTGGTCAAAGGTGCAGCTAAACATATCATTCATGCCGTAGGACCAAACTTCAACAAAGTTTCGGAGGTT
GAAGGTGACAAACAGTTGGCAGAGGCTTATGAGTCCATCGCTAAGATTGTCAACGATAACAATTACAAGTCAGTAGCGATTCCACTGTTGTCCACCG
GCATCTTTTCCGGGAACAAAGATCGACTAACCCAATCATTGAACCATTTGCTGACAGCTTTAGACACCACTGATGCAGATGTAGCCATATACTGCAG
GGACAAGAAATGGGAAATGACTCTCAAGGAAGCAGTGGCTAGGAGAGAAGCAGTGGAGGAGATATGCATATCCGACGACTCTTCAGTGACAGAACCT
GATGCAGAGCTGGTGAGGGTGCATCCGAAGAGTTCTTTGGCTGGAAGGAAGGGCTACAGCACAAGCGATGGCAAAACTTTCTCATATTTGGAAGGGA
CCAAGTTTCACCAGGCGGCCAAGGATATAGCAGAAATTAATGCCATGTGGCCCGTTGCAACGGAGGCCAATGAGCAGGTATGCATGTATATCCTCGG
AGAAGGCATGAGCAGTATTAGGTCGAAATGCCCCGTCGAAGAGTCGGAAGCCTCCACACCACCTAGCACGCTGCCTTGCTTGTGCATCCATGCCATG
ACTCCAGAAAGAGTACAGCGCCTAAAAGCCTCACGTCCAGAACAAATTACTGTGTGCTCATCCTTTCCATTGCCGAAGTATAGAATCACTGGTGTGC
AGAAGATCCAATGCTCCCAGCCTATATTGTTCTCACCGAAAGTGCCTGCGTATATTCATCCAAGGAAGTATCTCGTGGAAACACCACCGGTAGACGA
GACTCCGGAGCCATCGGCAGAGAACCAATCCACAGAGGGGACACCTGAACAACCACCACTTATAACCGAGGATGAGACCAGGACTAGAACGCCTGAG
CCGATCATCATCGAAGAGGAAGAAGAGGATAGCATAAGTTTGCTGTCAGATGGCCCGACCCACCAGGTGCTGCAAGTCGAGGCAGACATTCACGGGC
CGCCCTCTGTATCTAGCTCATCCTGGTCCATTCCTCATGCATCCGACTTTGATGTGGACAGTTTATCCATACTTGACACCCTGGAGGGAGCTAGCGT
GACCAGCGGGGCAACGTCAGCCGAGACTAACTCTTACTTCGCAAAGAGTATGGAGTTTCTGGCGCGACCGGTGCCTGCGCCTCGAACAGTATTCAGG
AACCCTCCACATCCCGCTCCGCGCACAAGAACACCGTCACTTGCACCCAGCAGGGCCTGCTCGAGAACCAGCCTAGTTTCCACCCCGCCAGGCGTGA
ATAGGGTGATCACTAGAGAGGAGCTCGAGGCGCTTACCCCGTCACGCACTCCTAGCAGGTCGGTCTCGAGAACCAGCCTGGTCTCCAACCCGCCAGG
CGTAAATAGGGTGATTACAAGAGAGGAGTTTGAGGCGTTCGTAGCACAACAACAATGACGGTTTGATGCGGGTGCATACATCTTTTCCTCCGACACC
GGTCAAGGGCATTTACAACAAAAATCAGTAAGGCAAACGGTGCTATCCGAAGTGGTGTTGGAGAGGACCGAATTGGAGATTTCGTATGCCCCGCGCC
TCGACCAAGAAAAAGAAGAATTACTACGCAAGAAATTACAGTTAAATCCCACACCTGCTAACAGAAGCAGATACCAGTCCAGGAAGGTGGAGAACAT
GAAAGCCATAACAGCTAGACGTATTCTGCAAGGCCTAGGGCATTATTTGAAGGCAGAAGGAAAAGTGGAGTGCTACCGAACCCTGCATCCTGTTCCT
TTGTATTCATCTAGTGTGAACCGTGCCTTTTCAAGCCCCAAGGTCGCAGTGGAAGCCTGTAACGCCATGTTGAAAGAGAACTTTCCGACTGTGGCTT
CTTACTGTATTATTCCAGAGTACGATGCCTATTTGGACATGGTTGACGGAGCTTCATGCTGCTTAGACACTGCCAGTTTTTGCCCTGCAAAGCTGCG
CAGCTTTCCAAAGAAACACTCCTATTTGGAACCCACAATACGATCGGCAGTGCCTTCAGCGATCCAGAACACGCTCCAGAACGTCCTGGCAGCTGCC
ACAAAAAGAAATTGCAATGTCACGCAAATGAGAGAATTGCCCGTATTGGATTCGGCGGCCTTTAATGTGGAATGCTTCAAGAAATATGCGTGTAATA
ATGAATATTGGGAAACGTTTAAAGAAAACCCCATCAGGCTTACTGAAGAAAACGTGGTAAATTACATTACCAAATTAAAAGGACCAAAAGCTGCTGC
TCTTTTTGCGAAGACACATAATTTGAATATGTTGCAGGACATACCAATGGACAGGTTTGTAATGGACTTAAAGAGAGACGTGAAAGTGACTCCAGGA
ACAAAACATACTGAAGAACGGCCCAAGGTACAGGTGATCCAGGCTGCCGATCCGCTAGCAACAGCGTATCTGTGCGGAATCCACCGAGAGCTGGTTA
GGAGATTAAATGCGGTCCTGCTTCCGAACATTCATACACTGTTTGATATGTCGGCTGAAGACTTTGACGCTATTATAGCCGAGCACTTCCAGCCTGG
GGATTGTGTTCTGGAAACTGACATCGCGTCGTTTGATAAAAGTGAGGACGACGCCATGGCTCTGACCGCGTTAATGATTCTGGAAGACTTAGGTGTG
GACGCAGAGCTGTTGACGCTGATTGAGGCGGCTTTCGGCGAAATTTCATCAATACATTTGCCCACTAAAACTAAATTTAAATTCGGAGCCATGATGA
AATCTGGAATGTTCCTCACACTGTTTGTGAACACAGTCATTAACATTGTAATCGCAAGCAGAGTGTTGAGAGAACGGCTAACCGGATCACCATGTGC
AGCATTCATTGGAGATGACAATATCGTGAAAGGAGTCAAATCGGACAAATTAATGGCAGACAGGTGCGCCACCTGGTTGAATATGGAAGTCAAGATT
ATAGATGCTGTGGTGGGCGAGAAAGCGCCTTATTTCTGTGGAGGGTTTATTTTGTGTGACTCCGTGACCGGCACAGCGTGCCGTGTGGCAGACCCCC
TAAAAAGGCTGTTTAAGCTTGGCAAACCTCTGGCAGCAGACGATGAACATGATGATGACAGGAGAAGGGCATTGCATGAAGAGTCAACACGCTGGAA
CCGAGTGGGTATTCTTTCAGAGCTGTGCAAGGCAGTAGAATCAAGGTATGAAACCGTAGGAACTTCCATCATAGTTATGGCCATGACTACTCTAGCT
AGCAGTGTTAAATCATTCAGCTACCTGAGAGGGGCCCCTATAACTCTCTACGGCTAACCTGAATGGACTACGACATAGTCTAGTCCGCCAAGTCTAG
catatgggcgcgccctcagcatcgattgaattggccaccATGGTGAGCAAGGGCGAGGAGGATAACATGGCCATCATCAAGGAGTTCATGCGCTTCA
AGGTGCACATGGAGGGCTCCGTGAACGGCCACGAGTTCGAGATCGAGGGCGAGGGCGAGGGCCGCCCCTACGAGGGCACCCAGACCGCCAAGCTGAA
GGTGACCAAGGGTGGCCCCCTGCCCTTCGCCTGGGACATCCTGTCCCCTCAGTTCATGTACGGCTCCAAGGCCTACGTGAAGCACCCCGCCGACATC
CCCGACTACTTGAAGCTGTCCTTCCCCGAGGGCTTCAAGTGGGAGCGCGTGATGAACTTCGAGGACGGCGGCGTGGTGACCGTGACCCAGGACTCCT
CCCTGCAGGACGGCGAGTTCATCTACAAGGTGAAGCTGCGCGGCACCAACTTCCCCTCCGACGGCCCCGTAATGCAGAAGAAGACCATGGGCTGGGA
GGCCTCCTCCGAGCGGATGTACCCCGAGGACGGCGCCCTGAAGGGCGAGATCAAGCAGAGGCTGAAGCTGAAGGACGGCGGCCACTACGACGCTGAG
GTCAAGACCACCTACAAGGCCAAGAAGCCCGTGCAGCTGCCCGGCGCCTACAACGTCAACATCAAGTTGGACATCACCTCCCACAACGAGGACTACA
CCATCGTGGAACAGTACGAACGCGCCGAGGGCCGCCACTCCACCGGCGGCATGGACGAGCTGTACAAGTAGgaattGGCAAGCTGCTTACATAGAAC
TCGCGGCGATTGGCATGCCGCCTTAAAATTTTTATTTTATTTTTCTTTTCTTTTCCGAATCGGATTTTGTTTTTAATATTTCAAAAAAAAAAAAAAA
AAAAAAAAAAACGCGTCGAGGGGAATTAATTCTTGAAGACGAAAGGGCCAGGTGGCACTTTTCGGGGAAATGTGCGCGGAACCCCTATTTGTTTATT
TTTCTAAATACATTCAAATATGTATCCGCTCATGAGACAATAACCCTGATAAATGCTTCAATAATATTGAAAAAGGAAGAGTATGAGTATTCAACAT
TTCCGTGTCGCCCTTATTCCCTTTTTTGCGGCATTTTGCCTTCCTGTTTTTGCTCACCCAGAAACGCTGGTGAAAGTAAAAGATGCTGAAGATCAGT
TGGGTGCACGAGTGGGTTACATCGAACTGGATCTCAACAGCGGTAAGATCCTTGAGAGTTTTCGCCCCGAAGAACGTTTTCCAATGATGAGCACTTT
TAAAGTTCTGCTATGTGGCGCGGTATTATCCCGTGTTGACGCCGGGCAAGAGCAACTCGGTCGCCGCATACACTATTCTCAGAATGACTTGGTTGAG
TACTCACCAGTCACAGAAAAGCATCTTACGGATGGCATGACAGTAAGAGAATTATGCAGTGCTGCCATAACCATGAGTGATAACACTGCGGCCAACT
TACTTCTGACAACGATCGGAGGACCGAAGGAGCTAACCGCTTTTTTGCACAACATGGGGGATCATGTAACTCGCCTTGATCGTTGGGAACCGGAGCT
GAATGAAGCCATACCAAACGACGAGCGTGACACCACGATGCCTGTAGCAATGGCAACAACGTTGCGCAAACTATTAACTGGCGAACTACTTACTCTA
GCTTCCCGGCAACAATTAATAGACTGGATGGAGGCGGATAAAGTTGCAGGACCACTTCTGCGCTCGGCCCTTCCGGCTGGCTGGTTTATTGCTGATA
AATCTGGAGCCGGTGAGCGTGGGTCTCGCGGTATCATTGCAGCACTGGGGCCAGATGGTAAGCCCTCCCGTATCGTAGTTATCTACACGACGGGGAG
TCAGGCAACTATGGATGAACGAAATAGACAGATCGCTGAGATAGGTGCCTCACTGATTAAGCATTGGTAACTGTCAGACCAAGTTTACTCATATATA
CTTTAGATTGATTTAAAACTTCATTTTTAATTTAAAAGGATCTAGGTGAAGATCCTTTTTGATAATCTCATGACCAAAATCCCTTAACGTGAGTTTT
CGTTCCACTGAGCGTCAGACCCCGTAGAAAAGATCAAAGGATCTTCTTGAGATCCTTTTTTTCTGCGCGTAATCTGCTGCTTGCAAACAAAAAAACC
ACCGCTACCAGCGGTGGTTTGTTTGCCGGATCAAGAGCTACCAACTCTTTTTCCGAAGGTAACTGGCTTCAGCAGAGCGCAGATACCAAATACTGTC
CTTCTAGTGTAGCCGTAGTTAGGCCACCACTTCAAGAACTCTGTAGCACCGCCTACATACCTCGCTCTGCTAATCCTGTTACCAGTGGCTGCTGCCA
GTGGCGATAAGTCGTGTCTTACCGGGTTGGACTCAAGACGATAGTTACCGGATAAGGCGCAGCGGTCGGGCTGAACGGGGGGTTCGTGCACACAGCC
CAGCTTGGAGCGAACGACCTACACCGAACTGAGATACCTACAGCGTGAGCATTGAGAAAGCGCCACGCTTCCCGAAGGGAGAAAGGCGGACAGGTAT

CCGGTAAGCGGCAGGGTCGGAACAGGAGAGCGCACGAGGGAGCTTCCAGGGGGAAACGCCTGGTATCTTTATAGTCCTGTCGGGTTTCGCCACCTCT
GACTTGAGCGTCGATTTTTGTGATGCTCGTCAGGGGGGCGGAGCCTATGGAAAAACGCCAGCAACGCGAGCTCTAATACGACTCACTATAG

>Ab2c1-mCherry
ATGGGCGGCGCATGAGAGAAGCCCAGACCAATTACCTACCCAAAATGGAGAAAGTTCACGTTGACATCGAGGAAGACAGCCCATTCCTCAGAGCTTT
GCAGCGGAGCTTCCCGCAGTTTGAGGTAGAAGCCAAGCAGGTCACTGATAATGACCATGCTAATGCCAGAGCGTTTTCGCATCTGGCTTCAAAACTG
ATCGAAACGGAGGTGGACCCATCCGACACGATCCTTGACATTGGAAGTGCGCCCGCCCGCAGAATGTATTCTAAGCACAAGTATCATTGTATCTGTC
CGATGAGATGTGCGGAAGATCCGGACAGATTGTATAAGTATGCAACTAAGCTGAAGAAAAACTGTAAGGAAATAACTGATAAGGAATTGGACAAGAA
AATGAAGGAGCTGGCCGCCGTCATGAGCGACCCTGACCTGGAAACTGAGACTATGTGCCTCCACGACGACGAGTCGTGTCGCTACGAAGGGCAAGTC
GCTGTTTACCAGGATGTATACGCGGTTGACGGACCGACAAGTCTCTATCACCAAGCCAATAAGGGAGTTAGAGTCGCCTACTGGATAGGCTTTGACA
CCACCCCTTTTATGTTTAAGAACTTGGCTGGAGCATATCCATCATACTCTACCAACTGGGCCGACGAAACCGTGTTAACGGCTCGTAACATAGGCCT
ATGCAGCTCTGACGTTATGGAGCGGTCACGTAGAGGGATGTCCATTCTTAGAAAGAAGTATTTGAAACCATCCAACAATGTTCTATTCTCTGTTGGC
TCGACCATCTACCACGAGAAGAGGGACTTACTGAGGAGCTGGCACCTGCCGTCTGTATTTCACTTACGTGGCAAGCAAAATTACACATGTCGGTGTG
AGACTATAGTTAGTTGCGACGGGTACGTCGTTAAAAGAATAGCTATCAGTCCAGGCCTGTATGGGAAGCCTTCAGGCTATGCTGCTACGATGCACCG
CGAGGGATTCTTGTGCTGCAAAGTGACAGACACATTGAACGGGGAGAGGGTCTCTTTTCCCGTGTGCACGTATGTGCCAGCTACATTGTGTGACCAA
ATGACTGGCATACTGGCAACAGATGTCAGTGCGGACGACGCGCAAAAACTGCTGGTTGGGCTCAACCAGCGTATAGTCGTCAACGGTCGCACCCAGA
GAAACACCAATACCATGAAAAATTACCTTTTGCCCGTAGTGGCCCAGGCATTTGCTAGGTGGGCAAAGGAATATAAGGAAGATCAAGAAGATGAAAG
GCCACTAGGACTACGAGATAGACAGTTAGTCATGGGGTGTTGTTGGGCTTTTAGAAGGCACAAGATAACATCTATTTATAAGCGCCCGGATACCCAA
ACCATCATCAAAGTGAACAGCGATTTCCACTCATTCGTGCTGCCCAGGATAGGCAGTAACACATTGGAGATCGGGCTGAGAACAAGAATCAGGAAAA
TGTTAGAGGAGCACAAGGAGCCGTCACCTCTCATTACCGCCGAGGACGTACAAGAAGCTAAGTGCGCAGCCGATGAGGCTAAGGAGGTGCGTGAAGC
CGAGGAGTTGCGCGCAGCTCTACCACCTTTGGCAGCTGATGTTGAGGAGCCCACTCTGGAGGCAGACGTCGACTTGATGTTACAAGAGGCTGGGGCC
GGCTCAGTGGAGACACCTCGTGGCTTGATAAAGGTTACCAGCTACGATGGCGAGGACAAGATCGGCTCTTACGCTGTGCTTTCTCCGCAGGCTGTAC
TCAAGAGTGAAAAATTATCTTGCATCCACCCTCTCGCTGAACAAGTCATAGTGATAACACACTCTGGCCGAAAAGGGCGTTATGCCGTGGAACCATA
CCATGGTAAAGTAGTGGTGCCAGAGGGACATGCAATACCCGTCCAGGACTTTCAAGCTCTGAGTGAAAGTGCCACCATTGTGTACAACGAACGTGAG
TTCGTAAACAGGTACCTGCACCATATTGCCACACATGGAGGAGCGCTGAACACTGATGAAGAATATTACAAAACTGTCAAGCCCAGCGAGCACGACG
GCGAATACCTGTACGACATCGACAGGAAACAGTGCGTCAAGAAAGAACTAGTCACTGGGCTAGGGCTCACAGGCGAGCTGGTGGATCCTCCCTTCCA
TGAATTCGCCTACGAGAGTCTGAGAACACGACCAGCCGCTCCTTACCAAGTACCAACCATAGGGGTGTATGGCGTGCCAGGATCAGGCAAGTCTGGC
ATCATTAAAAGCGCAGTCACCAAAAAAGATCTAGTGGTGAGCGCCAAGAAAGAAAACTGTGCAGAAATTATAAGGGACGTCAAGAAAATGAAAGGGC
TGGACGTCAATGCCAGAACTGTGGACTCAGTGCTCTTGAATGGATGCAAACACCCCGTAGAGACCCTGTATATTGACGAAGCTTTTGCTTGTCATGC
AGGTACTCTCAGAGCGCTCATAGCCATTATAAGACCTAAAAAGGCAGTGCTCTGCGGGGATCCCAAACAGTGCGGTTTTTTTAACATGATGTGCCTG
AAAGTGCATTTTAACCACGAGATTTGCACACAAGTCTTCCACAAAAGCATCTCTCGCCGTTGCACTAAATCTGTGACTTCGGTCGTCTCAACCTTGT
TTTACGACAAAAAAATGAGAACGACGAATCCGAAAGAGACTAAGATTGTGATTGACACTACCGGCAGTACCAAACCTAAGCAGGACGATCTCATTCT
CACTTGTTTCAGAGGGTGGGTGAAGCAGTTGCAAATAGATTACAAAGGCAACGAAATAATGACGGCAGCTGCCTCTCAAGGGCTGACCCGTAAAGGT
GTGTATGCCGTTCGGTACAAGGTGAATGAAAATCCTCTGTACGCACCCACCTCAGAACATGTGAACGTCCTACTGACCCGCACGGAGGACCGCATCG
TGTGGAAAACACTAGCCGGCGACCCATGGATAAAAACACTGACTGCCAAGTACCCTGGGAATTTCACTGCCACGATAGAGGAGTGGCAAGCAGAGCA
TGATGCCATCATGAGGCACATCTTGGAGAGACCGGACCCTACCGACGTCTTCCAGAATAAGGCAAACGTGTGTTGGGCCAAGGCTTTAGTGCCGGTG
CTGAAGACCGCTGGCATAGACATGACCACTGAACAATGGAACACTGTGGATTATTTTGAAACGGACAAAGCTCACTCAGCAGAGATAGTATTGAACC
AACTATGCGTGAGGTTCTTTGGACTCGATCTGGACTCCGGTCTATTTTCTGCACCCACTGTTCCGTTATCCATTAGGAATAATCACTGGGATAACTC
CCCGTCGCCTAACATGTACGGGCTGAATAAAGAAGTGGTCCGTCAGCTCTCTCGCAGGTACCCACAACTGCCTCGGGCAGTTGCCACTGGAAGAGTC
TATGACATGAACACTGGTACACTGCGCAATTATGATCCGCGCATAAACCTAGTACCTGTAAACAGAAGACTGCCTCATGCTTTAGTCCTCCACCATA
ATGAACACCCACAGAGTGACTTTTCTTCATTCGTCAGCAAATTGAAGGGCAGAACTGTCCTGGTGGTCGGGGAAAAGTTGTCCGTCCCAGGCAAAAT
GGTTGACTGGTTGTCAGACCGGCCTGAGGCTACCTTCAGAGCTCGGCTGGATTTAGGCATCCCAGGTGATGTGCCCAAATATGACATAATATTTGTT
AATGTGAGGACCCCATATAAATACCATCACTATCAGCAGTGTGAAGACCATGCCATTAAGCTTAGCATGTTGACCAAGAAAGCTTGTCTGCATCTGA
ATCCCGGCGGAACCTGTGTCAGCATAGGTTATGGTTACGCTGACAGGGCCAGCGAAAGCATCATTGGTGCTATAGCGCGGCAGTTCAAGTTTTCCCG
GGTATGCAAACCGAAATCCTCACTTGAAGAGACGGAAGTTCTGTTTGTATTCATTGGGTACGATCGCAAGGCCCGTACGCACAATTCTTACAAGCTT
TCATCAACCTTGACCAACATTTATACAGGTTCCAGACTCCACGAAGCCGGATGTGCACCCTCATATCATGTGGTGCGAGGGGATATTGCCACGGCCA
CCGAAGGAGTGATTATAAATGCTGCTAACAGCAAAGGACAACCTGGCGGAGGGGTGTGCGGAGCGCTGTATAAGAAATTCCCGGAAAGCTTCGATTT
ACAGCCGATCGAAGTAGGAAAAGCGCGACTGGTCAAAGGTGCAGCTAAACATATCATTCATGCCGTAGGACCAAACTTCAACAAAGTTTCGGAGGTT
GAAGGTGACAAACAGTTGGCAGAGGCTTATGAGTCCATCGCTGAGATTGTCAACGATAACAATTACAAGTCAGTAGCGATTCCACTGTTGTCCACCG
GCATCTTTTCCGGGAACAAAGATCGACTAACCCAATCATTGAACCATTTGCTGACAGCTTTAGACACCACTGATGCAGATGTAGCCATATACTGCAG
GGACAAGAAATGGGAAATGACTCTCAAGGAAGCAGTGGCTAGGAGAGAAGCAGTGGAGGAGATATGCATATCCGACGACTCTTCAGTGACAGAACCT
GATGCAGAGCTGGTGAGGGTGCATCCGAAGAGTTCTTTGGCTGGAAGGAAGGGCTACAGCACAAGCGATGGCAAAACTTTCTCATATTTGGAAGGGA
CCAAGTTTCACCAGGCGGCCAAGGATATAGCAGAAATTAATGCCATGTGGCCCGTTGCAACGGAGGCCAATGAGCAGGTATGCATGTATATCCTCGG
AGAAGGCATGAGCAGTATTAGGTCGAAATGCCCCGTCGAAGAGTCGGAAGCCTCCACACCACCTAGCACGCTGCCTTGCTTGTGCATCCATGCCATG
ACTCCAGAAAGAGTACAGCGCCTAAAAGCCTCACGTCCAGAACAAATTACTGTGTGCTCATCCTTTCCATTGCCGAAGTATAGAATCACTGGTGTGC
AGAAGATCCAATGCTCCCAGCCTATATTGTTCTCACCGAAAGTGCCTGCGTATATTCATCCAAGGAAGTATCTCGTGGAAACACCACCGGTAGACGA
GACTCCGGAGCCATCGGCAGAGAACCAATCCACAGAGGGGACACCTGAACAACCACCACTTATAACCGAGGATGAGACCAGGACTAGAACGCCTGAG
CCGATCATCATCGAAGAGGAAGAAGAGGATAGCATAAGTTTGCTGTCAGATGGCCCGACCCACCAGGTGCTGCAAGTCGAGGCAGACATTCACGGGC
CGCCCTCTGTATCTAGCTCATCCTGGTCCATTCCTCATGCATCCGACTTTGATGTGGACAGTTTATCCATACTTGACACCCTGGAGGGAGCTAGCGT
GACCAGCGGGGCAACGTCAGCCGAGACTAACTCTTACTTCGCAAAGAGTATGGAGTTTCTGGCGCGACCGGTGCCTGCGCCTCGAACAGTATTCAGG
AACCCTCCACATCCCGCTCCGCGCACAAGAACACCGTCACTTGCACCCAGCAGGGCCTGCTCGAGAACCAGCCTAGTTTCCACCCCGCCAGGCGTGA
ATAGGGTGATCACTAGAGAGGAGCTCGAGGCGCTTACCCCGTCACGCACTCCTAGCAGGTCGGTCTCGAGAACCAGCCTGGTCTCCAACCCGCCAGG
CGTAAATAGGGTGATTACAAGAGAGGAGTTTGAGGCGTTCGTAGCACAACAACAATGACGGTTTGATGCGGGTGCATACATCTTTTCCTCCGACACC
GGTCAAGGGCATTTACAACAAAAATCAGTAAGGCAAACGGTGCTATCCGAAGTGGTGTTGGAGAGGACCGAATTGGAGATTTCGTATGCCCCGCGCC
TCGACCAAGAAAAAGAAGAATTACTACGCAAGAAATTACAGTTAAATCCCACACCTGCTAACAGAAGCAGATACCAGTCCAGGAAGGTGGAGAACAT
GAAAGCCATAACAGCTAGACGTATTCTGCAAGGCCTAGGGCATTATTTGAAGGCAGAAGGAAAAGTGGAGTGCTACCGAACCCTGCATCCTGTTCCT
TTGTATTCATCTAGTGTGAACCGTGCCTTTTCAAGCCCCAAGGTCGCAGTGGAAGCCTGTAACGCCATGTTGAAAGAGAACTTTCCGACTGTGGCTT
CTTACTGTATTATTCCAGAGTACGATGCCTATTTGGACATGGTTGACGGAGCTTCATGCTGCTTAGACACTGCCAGTTTTTGCCCTGCAAAGCTGCG
CAGCTTTCCAAAGAAACACTCCTATTTGGAACCCACAATACGATCGGCAGTGCCTTCAGCGATCCAGAACACGCTCCAGAACGTCCTGGCAGCTGCC
ACAAAAAGAAATTGCAATGTCACGCAAATGAGAGAATTGCCCGTATTGGATTCGGCGGCCTTTAATGTGGAATGCTTCAAGAAATATGCGTGTAATA
ATGAATATTGGGAAACGTTTAAAGAAAACCCCATCAGGCTTACTGAAGAAAACGTGGTAAATTACATTACCAAATTAAAAGGACCAAAAGCTGCTGC
TCTTTTTGCGAAGACACATAATTTGAATATGTTGCAGGACATACCAATGGACAGGTTTGTAATGGACTTAAAGAGAGACGTGAAAGTGACTCCAGGA

```
ACAAAACATACTGAAGAACGGCCCAAGGTACAGGTGATCCAGGCTGCCGATCCGCTAGCAACAGCGTATCTGTGCGGAATCCACCGAGAGCTGGTTA
GGAGATTAAATGCGGTCCTGCTTCCGAACATTCATACACTGTTTGATATGTCGGCTGAAGACTTTGACGCTATTATAGCCGAGCACTTCCAGCCTGG
GGATTGTGTTCTGGAAACTGACATCGCGTCGTTTGATAAAAGTGAGGACGACGCCATGGCTCTGACCGCGTTAATGATTCTGGAAGACTTAGGTGTG
GACGCAGAGCTGTTGACGCTGATTGAGGCGGCTTTCGGCGAAATTTCATCAATACATTTGCCCACTAAAACTAAATTTAAATTCGGAGCCATGATGA
AATCTGGAATGTTCCTCACACTGTTTGTGAACACAGTCATTAACATTGTAATCGCAAGCAGAGTGTTGAGAGAACGGCTAACCGGATCACCATGTGC
AGCATTCATTGGAGATGACAATATCGTGAAAGGAGTCAAATCGGACAAATTAATGGCAGACAGGTGCGCCACCTGGTTGAATATGGAAGTCAAGATT
ATAGATGCTGTGGTGGGCGAGAAAGCGCCTTATTTCTGTGGAGGGTTTATTTTGTGTGACTCCGTGACCGGCACAGCGTGCCGTGTGGCAGACCCCC
TAAAAAGGCTGTTTAAGCTTGGCAAACCTCTGGCAGCAGACGATGAACATGATGATGACAGGAGAAGGGCATTGCATGAAGAGTCAACACGCTGGAA
CCGAGTGGGTATTCTTTCAGAGCTGTGCAAGGCAGTAGAATCAAGGTATGAAACCGTAGGAACTTCCATCATAGTTATGGCCATGACTACTCTAGCT
AGCAGTGTTAAATCATTCAGCTACCTGAGAGGGGCCCCTATAACTCTCTACGGCTAACCTGAATGGACTACGACATAGTCTAGTCCGCCAAGTCTAG
catatgggcgcgccctcagcatcgattgaattggccaccATGGTGAGCAAGGGCGAGGAGGATAACATGGCCATCATCAAGGAGTTCATGCGCTTCA
AGGTGCACATGGAGGGCTCCGTGAACGGCCACGAGTTCGAGATCGAGGGCGAGGGCGAGGGCCGCCCCTACGAGGGCACCCAGACCGCCAAGCTGAA
GGTGACCAAGGGTGGCCCCCTGCCCTTCGCCTGGGACATCCTGTCCCCTCAGTTCATGTACGGCTCCAAGGCCTACGTGAAGCACCCCGCCGACATC
CCCGACTACTTGAAGCTGTCCTTCCCCGAGGGCTTCAAGTGGGAGCGCGTGATGAACTTCGAGGACGGCGGCGTGGTGACCGTGACCCAGGACTCCT
CCCTGCAGGACGGCGAGTTCATCTACAAGGTGAAGCTGCGCGGCACCAACTTCCCCTCCGACGGCCCCGTAATGCAGAAGAAGACCATGGGCTGGGA
GGCCTCCTCCGAGCGGATGTACCCCGAGGACGGCGCCCTGAAGGGCGAGATCAAGCAGAGGCTGAAGCTGAAGGACGGCGGCCACTACGACGCTGAG
GTCAAGACCACCTACAAGGCCAAGAAGCCCGTGCAGCTGCCCGGCGCCTACAACGTCAACATCAAGTTGGACATCACCTCCCACAACGAGGACTACA
CCATCGTGGAACAGTACGAACGCGCCGAGGGCCGCCACTCCACCGGCGGCATGGACGAGCTGTACAAGTAGgaattGGCAAGCTGCTTACATAGAAC
TCGCGGCGATTGGCATGCCGCCTTAAAATTTTTATTTTATTTTTCTTTTCTTTTCCGAATCGGATTTTGTTTTTAATATTTCAAAAAAAAAAAAAAA
AAAAAAAAAAACGCGTCGAGGGGAATTAATTCTTGAAGACGAAAGGGCCAGGTGGCACTTTTCGGGGAAATGTGCGCGGAACCCCTATTTGTTTATT
TTTCTAAATACATTCAAATATGTATCCGCTCATGAGACAATAACCCTGATAAATGCTTCAATAATATTGAAAAAGGAAGAGTATGAGTATTCAACAT
TTCCGTGTCGCCCTTATTCCCTTTTTTGCGGCATTTTGCCTTCCTGTTTTTGCTCACCCAGAAACGCTGGTGAAAGTAAAAGATGCTGAAGATCAGT
TGGGTGCACGAGTGGGTTACATCGAACTGGATCTCAACAGCGGTAAGATCCTTGAGAGTTTTCGCCCCGAAGAACGTTTTCCAATGATGAGCACTTT
TAAAGTTCTGCTATGTGGCGCGGTATTATCCCGTGTTGACGCCGGGCAAGAGCAACTCGGTCGCCGCATACACTATTCTCAGAATGACTTGGTTGAG
TACTCACCAGTCACAGAAAAGCATCTTACGGATGGCATGACAGTAAGAGAATTATGCAGTGCTGCCATAACCATGAGTGATAACACTGCGGCCAACT
TACTTCTGACAACGATCGGAGGACCGAAGGAGCTAACCGCTTTTTTGCACAACATGGGGGATCATGTAACTCGCCTTGATCGTTGGGAACCGGAGCT
GAATGAAGCCATACCAAACGACGAGCGTGACACCACGATGCCTGTAGCAATGGCAACAACGTTGCGCAAACTATTAACTGGCGAACTACTTACTCTA
GCTTCCCGGCAACAATTAATAGACTGGATGGAGGCGGATAAAGTTGCAGGACCACTTCTGCGCTCGGCCCTTCCGGCTGGCTGGTTTATTGCTGATA
AATCTGGAGCCGGTGAGCGTGGGTCTCGCGGTATCATTGCAGCACTGGGGCCAGATGGTAAGCCCTCCCGTATCGTAGTTATCTACACGACGGGGAG
TCAGGCAACTATGGATGAACGAAATAGACAGATCGCTGAGATAGGTGCCTCACTGATTAAGCATTGGTAACTGTCAGACCAAGTTTACTCATATATA
CTTTAGATTGATTTAAAACTTCATTTTTAATTTAAAAGGATCTAGGTGAAGATCCTTTTTGATAATCTCATGACCAAAATCCCTTAACGTGAGTTTT
CGTTCCACTGAGCGTCAGACCCCGTAGAAAAGATCAAAGGATCTTCTTGAGATCCTTTTTTTCTGCGCGTAATCTGCTGCTTGCAAACAAAAAAACC
ACCGCTACCAGCGGTGGTTTGTTTGCCGGATCAAGAGCTACCAACTCTTTTTCCGAAGGTAACTGGCTTCAGCAGAGCGCAGATACCAAATACTGTC
CTTCTAGTGTAGCCGTAGTTAGGCCACCACTTCAAGAACTCTGTAGCACCGCCTACATACCTCGCTCTGCTAATCCTGTTACCAGTGGCTGCTGCCA
GTGGCGATAAGTCGTGTCTTACCGGGTTGGACTCAAGACGATAGTTACCGGATAAGGCGCAGCGGTCGGGCTGAACGGGGGGTTCGTGCACACAGCC
CAGCTTGGAGCGAACGACCTACACCGAACTGAGATACCTACAGCGTGAGCATTGAGAAAGCGCCACGCTTCCCGAAGGGAGAAAGGCGGACAGGTAT
CCGGTAAGCGGCAGGGTCGGAACAGGAGAGCGCACGAGGGAGCTTCCAGGGGGAAACGCCTGGTATCTTTATAGTCCTGTCGGGTTTCGCCACCTCT
GACTTGAGCGTCGATTTTTGTGATGCTCGTCAGGGGGGCGGAGCCTATGGAAAAACGCCAGCAACGCGAGCTCTAATACGACTCACTATAG

>ab2c1-mCherry
ATGGGCGGCGCATGAGAGAAGCCCAGACCAATTACCTACCCAAAATGGAGAAAGTTCACGTTGACATCGAGGAAGACAGCCCATTCCTCAGAGCTTT
GCAGCGGAGCTTCCCGCAGTTTGAGGTAGAAGCCAAGCAGGTCACTGATAATGACCATGCTAATGCCAGAGCGTTTTCGCATCTGGCTTCAAAACTG
ATCGAAACGGAGGTGGACCCATCCGACACGATCCTTGACATTGGAAGTGCGCCCGCCCGCAGAATGTATTCTAAGCACAAGTATCATTGTATCTGTC
CGATGAGATGTGCGGAAGATCCGGACAGATTGTATAAGTATGCAACTAAGCTGAAGAAAAACTGTAAGGAAATAACTGATAAGGAATTGGACAAGAA
AATGAAGGAGCTGGCCGCCGTCATGAGCGACCCTGACCTGGAAACTGAGACTATGTGCCTCCACGACGACGAGTCGTGTCGCTACGAAGGGCAAGTC
GCTGTTTACCAGGATGTATACGCGGTTGACGGACCGACAAGTCTCTATCACCAAGCCAATAAGGGAGTTAGAGTCGCCTACTGGATAGGCTTTGACA
CCACCCCTTTTATGTTTAAGAACTTGGCTGGAGCATATCCATCATACTCTACCAACTGGGCCGACGAAACCGTGTTAACGGCTCGTAACATAGGCCT
ATGCAGCTCTGACGTTATGGAGCGGTCACGTAGAGGGATGTCCATTCTTAGAAAGAAGTATTTGAAACCATCCAACAATGTTCTATTCTCTGTTGGC
TCGACCATCTACCACGAGAAGAGGGACTTACTGAGGAGCTGGCACCTGCCGTCTGTATTTCACTTACGTGGCAAGCAAAATTACACATGTCGGTGTG
AGACTATAGTTAGTTGCGACGGGTACGTCGTTAAAAGAATAGCTATCAGTCCAGGCCTGTATGGGAAGCCTTCAGGCTATGCTGCTACGATGCACCG
CGAGGGATTCTTGTGCTGCAAAGTGACAGACACATTGAACGGGGAGAGGGTCTCTTTTCCCGTGTGCACGTATGTGCCAGCTACATTGTGTGACCAA
ATGACTGGCATACTGGCAACAGATGTCAGTGCGGACGACGCGCAAAAACTGCTGGTTGGGCTCAACCAGCGTATAGTCGTCAACGGTCGCACCCAGA
GAAACACCAATACCATGAAAAATTACCTTTTGCCCGTAGTGGCCCAGGCATTTGCTAGGTGGGCAAAGGAATATAAGGAAGATCAAGAAGATGAAAG
GCCACTAGGACTACGAGATAGACAGTTAGTCATGGGGTGTTGTTGGGCTTTTAGAAGGCACAAGATAACATCTATTTATAAGCGCCCGGATACCCAA
ACCATCATCAAAGTGAACAGCGATTTCCACTCATTCGTGCTGCCCAGGATAGGCAGTAACACATTGGAGATCGGGCTGAGAACAAGAATCAGGAAAA
TGTTAGAGGAGCACAAGGAGCCGTCACCTCTCATTACCGCCGAGGACGTACAAGAAGCTAAGTGCGCAGCCGATGAGGCTAAGGAGGTGCGTGAAGC
CGAGGAGTTGCGCGCAGCTCTACCACCTTTGGCAGCTGATGTTGAGGAGCCCACTCTGGAGGCAGACGTCGACTTGATGTTACAAGAGGCTGGGGCC
GGCTCAGTGGAGACACCTCGTGGCTTGATAAAGGTTACCAGCTACGATGGCGAGGACAAGATCGGCTCTTACGCTGTGCTTTCTCCGCAGGCTGTAC
TCAAGAGTGAAAAATTATCTTGCATCCACCCTCTCGCTGAACAAGTCATAGTGATAACACACTCTGGCCGAAAAGGGCGTTATGCCGTGGAACCATA
CCATGGTAAAGTAGTGGTGCCAGAGGGACATGCAATACCCGTCCAGGACTTTCAAGCTCTGAGTGAAAGTGCCACCATTGTGTACAACGAACGTGAG
TTCGTAAACAGGTACCTGCACCATATTGCCACACATGGGGGAGCGCTGAACACTGATGAAGAATATTACAAAACTGTCAAGCCCAGCGAGCACGACG
GCGAATACCTGTACGACATCGACAGGAAACAGTGCGTCAAGAAAGAACTAGTCACTGGGCTAGGGCTCACAGGCGAGCTGGTGGATCCTCCCTTCCA
TGAATTCGCCTACGAGAGTCTGAGAACACGACCAGCCGCTCCTTACCAAGTACCAACCATAGGGGTGTATGGCGTGCCAGGATCAGGCAAGTCTGGC
ATCATTAAAAGCGCAGTCACCAAAAAAGATCTAGTGGTGAGCGCCAAGAAAGAAAACTGTGCAGAAATTATAAGGGACGTCAAGAAAATGAAAGGGC
TGGACGTCAATGCCAGAACTGTGGACTCAGTGCTCTTGAATGGATGCAAACACCCCGTAGAGACCCTGTATATTGACGAAGCTTTTGCTTGTCATGC
AGGTACTCTCAGAGCGCTCATAGCCATTATAAGACCTAAAAAGGCAGTGCTCTGCGGGGATCCCAAACAGTGCGGTTTTTTAACATGATGTGCCTG
AAAGTGCATTTTAACCACGAGATTTGCACACAAGTCTTCCACAAAAGCATCTCTCGCCGTTGCACTAAATCTGTGACTTCGGTCGTCTCAACCTTGT
TTTACGACAAAAAAATGAGAACGACGAATCCGAAAGAGACTAAGATTGTGATTGACACTACCGGCAGTACCAAACCTAAGCAGGACGATCTCATTCT
CACTTGTTTCAGAGGGTGGGTGAAGCAGTTGCAAATAGATTACAAAGGCAACGAAATAATGACGGCAGCTGCCTCTCAAGGGCTGACCCGTAAAGGT
GTGTATGCCGTTCGGTACAAGGTGAATGAAAATCCTCTGTACGCACCCACCTCAGAACATGTGAACGTCCTACTGACCCGCACGGAGGACCGCATCG
TGTGGAAAACACTAGCCGGCGACCCATGGATAAAAACACTGACTGCCAAGTACCCTGGGAATTTCACTGCCACGATAGAGGAGTGGCAAGCAGAGCA
TGATGCCATCATGAGGCACATCTTGGAGAGACCGGACCCTACCGACGTCTTCCAGAATAAGGCAAACGTGTGTTGGGCCAAGGCTTTAGTGCCGGTG
```

CTGAAGACCGCTGGCATAGACATGACCACTGAACAATGGAACACTGTGGATTATTTTGAAACGGACAAAGCTCACTCAGCAGAGATAGTATTGAACC
AACTATGCGTGAGGTTCTTTGGACTCGATCTGGACTCCGGTCTATTTTCTGCACCCACTGTTCCGTTATCCATTAGGAATAATCACTGGGATAACTC
CCCGTCGCCTAACATGTACGGGCTGAATAAAGAAGTGGTCCGTCAGCTCTCTCGCAGGTACCCACAACTGCCTCGGGCAGTTGCCACTGGAAGAGTC
TATGACATGAACACTGGTACACTGCGCAATTATGATCCGCGCATAAACCTAGTACCTGTAAACAGAAGACTGCCTCATGCTTTAGTCCTCCACCATA
ATGAACACCCACAGAGTGACTTTTCTTCATTCGTCAGCAAATTGAAGGGCAGAACTGTCCTGGTGGTCGGGGAAAAGTTGTCCGTCCCAGGCAAAAT
GGTTGACTGGTTGTCAGACCGGCCTGAGGCTACCTTCAGAGCTCGGCTGGATTTAGGCATCCCAGGTGATGTGCCCAAATATGACATAATATTTGTT
AATGTGAGGACCCCATATAAATACCATCACTATCAGCAGTGTGAAGACCATGCCATTAAGCTTAGCATGTTGACCAAGAAAGCTTGTCTGCATCTGA
ATCCCGGCGGAACCTGTGTCAGCATAGGTTATGGTTACGCTGACAGGGCCAGCGAAAGCATCATTGGTGCTATAGCGCGGCAGTTCAAGTTTTCCCG
GGTATGCAAACCGAAATCCTCACTTGAAGAGACGGAAGTTCTGTTTGTATTCATTGGGTACGATCGCAAGGCCCGTACGCACAATTCTTACAAGCTT
TCATCAACCTTGACCAACATTTATACAGGTTCCAGACTCCACGAAGCCGGATGTGCACCCTCATATCATGTGGTGCGAGGGGATATTGCCACGGCCA
CCGAAGGAGTGATTATAAATGCTGCTAACAGCAAAGGACAACCTGGCGGAGGGGTGTGCGGAGCGCTGTATAAGAAATTCCCGGAAAGCTTCGATTT
ACAGCCGATCGAAGTAGGAAAAGCGCGACTGGTCAAAGGTGCAGCTAAACATATCATTCATGCCGTAGGACCAAACTTCAACAAAGTTTCGGAGGTT
GAAGGTGACAAACAGTTGGCAGAGGCTTATGAGTCCATCGCTGAGATTGTCAACGATAACAATTACAAGTCAGTAGCGATTCCACTGTTGTCCACCG
GCATCTTTTCCGGGAACAAAGATCGACTAACCCAATCATTGAACCATTTGCTGACAGCTTTAGACACCACTGATGCAGATGTAGCCATATACTGCAG
GGACAAGAAATGGGAAATGACTCTCAAGGAAGCAGTGGCTAGGAGAGAAGCAGTGGAGGAGATATGCATATCCGACGACTCTTCAGTGACAGAACCT
GATGCAGAGCTGGTGAGGGTGCATCCGAAGAGTTCTTTGGCTGGAAGGAAGGGCTACAGCACAAGCGATGGCAAAACTTTCTCATATTTGGAAGGGA
CCAAGTTTCACCAGGCGGCCAAGGATATAGCAGAAATTAATGCCATGTGGCCCGTTGCAACGGAGGCCAATGAGCAGGTATGCATGTATATCCTCGG
AGAAGGCATGAGCAGTATTAGGTCGAAATGCCCCGTCGAAGAGTCGGAAGCCTCCACACCACCTAGCACGCTGCCTTGCTTGTGCATCCATGCCATG
ACTCCAGAAAGAGTACAGCGCCTAAAAGCCTCACGTCCAGAACAAATTACTGTGTGCTCATCCTTTCCATTGCCGAAGTATAGAATCACTGGTGTGC
AGAAGATCCAATGCTCCCAGCCTATATTGTTCTCACCGAAAGTGCCTGCGTATATTCATCCAAGGAAGTATCTCGTGGAAACACCACCGGTAGACGA
GACTCCGGAGCCATCGGCAGAGAACCAATCCACAGAGGGGACACCTGAACAACCACCACTTATAACCGAGGATGAGACCAGGACTAGAACGCCTGAG
CCGATCATCATCGAAGAGGAAGAAGAGGATAGCATAAGTTTGCTGTCAGATGGCCCGACCCACCAGGTGCTGCAAGTCGAGGCAGACATTCACGGGC
CGCCCTCTGTATCTAGCTCATCCTGGTCCATTCCTCATGCATCCGACTTTGATGTGGACAGTTTATCCATACTTGACACCCTGGAGGGAGCTAGCGT
GACCAGCGGGGCAACGTCAGCCGAGACTAACTCTTACTTCGCAAAGAGTATGGAGTTTCTGGCGCGACCGGTGCCTGCGCCTCGAACAGTATTCAGG
AACCCTCCACATCCCGCTCCGCGCACAAGAACACCGTCACTTGCACCCAGCAGGGCCTGCTCGAGAACCAGCCTAGTTTCCACCCCGCCAGGCGTGA
ATAGGGTGATCACTAGAGAGGAGCTCGAGGCGCTTACCCCGTCACGCACTCCTAGCAGGTCGGTCTCGAGAACCAGCCTGGTCTCCAACCCGCCAGG
CGTAAATAGGGTGATTACAAGAGAGGAGTTTGAGGCGTTCGTAGCACAACAACAATGACGGTTTGATGCGGGTGCATACATCTTTTCCTCCGACACC
GGTCAAGGGCATTTACAACAAAAATCAGTAAGGCAAACGGTGCTATCCGAAGTGGTGTTGGAGAGGACCGAATTGGAGATTTCGTATGCCCCGCGCC
TCGACCAAGAAAAAGAAGAATTACTACGCAAGAAATTACAGTTAAATCCCACACCTGCTAACAGAAGCAGATACCAGTCCAGGAAGGTGGAGAACAT
GAAAGCCATAACAGCTAGACGTATTCTGCAAGGCCTAGGGCATTATTTGAAGGCAGAAGGAAAAGTGGAGTGCTACCGAACCCTGCATCCTGTTCCT
TTGTATTCATCTAGTGTGAACCGTGCCTTTTCAAGCCCCAAGGTCGCAGTGGAAGCCTGTAACGCCATGTTGAAAGAGAACTTTCCGACTGTGGCTT
CTTACTGTATTATTCCAGAGTACGATGCCTATTTGGACATGGTTGACGGAGCTTCATGCTGCTTAGACACTGCCAGTTTTTGCCCTGCAAAGCTGCG
CAGCTTTCCAAAGAAACACTCCTATTTGGAACCCACAATACGATCGGCAGTGCCTTCAGCGATCCAGAACACGCTCCAGAACGTCCTGGCAGCTGCC
ACAAAAAGAAATTGCAATGTCACGCAAATGAGAGAATTGCCCGTATTGGATTCGGCGGCCTTTAATGTGGAATGCTTCAAGAAATATGCGTGTAATA
ATGAATATTGGGAAACGTTTAAAGAAAACCCCATCAGGCTTACTGAAGAAAACGTGGTAAATTACATTACCAAATTAAAAGGACCAAAAGCTGCTGC
TCTTTTTGCGAAGACACATAATTTGAATATGTTGCAGGACATACCAATGGACAGGTTTGTAATGGACTTAAAGAGAGACGTGAAAGTGACTCCAGGA
ACAAAACATACTGAAGAACGGCCCAAGGTACAGGTGATCCAGGCTGCCGATCCGCTAGCAACAGCGTATCTGTGCGGAATCCACCGAGAGCTGGTTA
GGAGATTAAATGCGGTCCTGCTTCCGAACATTCATACACTGTTTGATATGTCGGCTGAAGACTTTGACGCTATTATAGCCGAGCACTTCCAGCCTGG
GGATTGTGTTCTGGAAACTGACATCGCGTCGTTTGATAAAAGTGAGGACGACGCCATGGCTCTGACCGCGTTAATGATTCTGGAAGACTTAGGTGTG
GACGCAGAGCTGTTGACGCTGATTGAGGCGGCTTTCGGCGAAATTTCATCAATACATTTGCCCACTAAAACTAAATTTAAATTCGGAGCCATGATGA
AATCTGGAATGTTCCTCACACTGTTTGTGAACACAGTCATTAACATTGTAATCGCAAGCAGAGTGTTGAGAGAACGGCTAACCGGATCACCATGTGC
AGCATTCATTGGAGATGACAATATCGTGAAAGGAGTCAAATCGGACAAATTAATGGCAGACAGGTGCGCCACCTGGTTGAATATGGAAGTCAAGATT
ATAGATGCTGTGGTGGGCGAGAAAGCGCCTTATTTCTGTGGAGGGTTTATTTTGTGTGACTCCGTGACCGGCACAGCGTGCCGTGTGGCAGACCCCC
TAAAAAGGCTGTTTAAGCTTGGCAAACCTCTGGCAGCAGACGATGAACATGATGATGACAGGAGAAGGGCATTGCATGAAGAGTCAACACGCTGGAA
CCGAGTGGGTATTCTTTCAGAGCTGTGCAAGGCAGTAGAATCAAGGTATGAAACCGTAGGAACTTCCATCATAGTTATGGCCATGACTACTCTAGCT
AGCAGTGTTAAATCATTCAGCTACCTGAGAGGGGCCCCTATAACTCTCTACGGCTAACCTGAATGGACTACGACATAGTCTAGTCCGCCAAGTCTAG
catatgggcgcgccctcagcatcgattgaattggccaccATGGTGAGCAAGGGCGAGGAGGATAACATGGCCATCATCAAGGAGTTCATGCGCTTCA
AGGTGCACATGGAGGGCTCCGTGAACGGCCACGAGTTCGAGATCGAGGGCGAGGGCGAGGGCCGCCCCTACGAGGGCACCCAGACCGCCAAGCTGAA
GGTGACCAAGGGTGGCCCCCTGCCCTTCGCCTGGGACATCCTGTCCCCTCAGTTCATGTACGGCTCCAAGGCCTACGTGAAGCACCCCGCCGACATC
CCCGACTACTTGAAGCTGTCCTTCCCCGAGGGCTTCAAGTGGGAGCGCGTGATGAACTTCGAGGACGGCGGCGTGGTGACCGTGACCCAGGACTCCT
CCCTGCAGGACGGCGAGTTCATCTACAAGGTGAAGCTGCGCGGCACCAACTTCCCCTCCGACGGCCCCGTAATGCAGAAGAAGACCATGGGCTGGGA
GGCCTCCTCCGAGCGGATGTACCCCGAGGACGGCGCCCTGAAGGGCGAGATCAAGCAGAGGCTGAAGCTGAAGGACGGCGGCCACTACGACGCTGAG
GTCAAGACCACCTACAAGGCCAAGAAGCCCGTGCAGCTGCCCGGCGCCTACAACGTCAACATCAAGTTGGACATCACCTCCCACAACGAGGACTACA
CCATCGTGGAACAGTACGAACGCGCCGAGGGCCGCCACTCCACCGGCGGCATGGACGAGCTGTACAAGTAGgaattGGCAAGCTGCTTACATAGAAC
TCGCGGCGATTGGCATGCCGCCTTAAAATTTTTATTTTATTTTTCTTTTCTTTTCCGAATCGGATTTTGTTTTTAATATTTCAAAAAAAAAAAAAAA
AAAAAAAAAAACGCGTCGAGGGGAATTAATTCTTGAAGACGAAAGGGCCAGGTGGCACTTTTCGGGGAAATGTGCGCGGAACCCCTATTTGTTTATT
TTTCTAAATACATTCAAATATGTATCCGCTCATGAGACAATAACCCTGATAAATGCTTCAATAATATTGAAAAAGGAAGAGTATGAGTATTCAACAT
TTCCGTGTCGCCCTTATTCCCTTTTTTGCGGCATTTTGCCTTCCTGTTTTTGCTCACCCAGAAACGCTGGTGAAAGTAAAAGATGCTGAAGATCAGT
TGGGTGCACGAGTGGGTTACATCGAACTGGATCTCAACAGCGGTAAGATCCTTGAGAGTTTTCGCCCCGAAGAACGTTTTCCAATGATGAGCACTTT
TAAAGTTCTGCTATGTGGCGCGGTATTATCCCGTGTTGACGCCGGGCAAGAGCAACTCGGTCGCCGCATACACTATTCTCAGAATGACTTGGTTGAG
TACTCACCAGTCACAGAAAAGCATCTTACGGATGGCATGACAGTAAGAGAATTATGCAGTGCTGCCATAACCATGAGTGATAACACTGCGGCCAACT
TACTTCTGACAACGATCGGAGGACCGAAGGAGCTAACCGCTTTTTTGCACAACATGGGGGATCATGTAACTCGCCTTGATCGTTGGGAACCGGAGCT
GAATGAAGCCATACCAAACGACGAGCGTGACACCACGATGCCTGTAGCAATGGCAACAACGTTGCGCAAACTATTAACTGGCGAACTACTTACTCTA
GCTTCCCGGCAACAATTAATAGACTGGATGGAGGCGGATAAAGTTGCAGGACCACTTCTGCGCTCGGCCCTTCCGGCTGGCTGGTTTATTGCTGATA
AATCTGGAGCCGGTGAGCGTGGGTCTCGCGGTATCATTGCAGCACTGGGGCCAGATGGTAAGCCCTCCCGTATCGTAGTTATCTACACGACGGGGAG
TCAGGCAACTATGGATGAACGAAATAGACAGATCGCTGAGATAGGTGCCTCACTGATTAAGCATTGGTAACTGTCAGACCAAGTTTACTCATATATA
CTTTAGATTGATTTAAAACTTCATTTTTAATTTAAAAGGATCTAGGTGAAGATCCTTTTTGATAATCTCATGACCAAAATCCCTTAACGTGAGTTTT
CGTTCCACTGAGCGTCAGACCCCGTAGAAAAGATCAAAGGATCTTCTTGAGATCCTTTTTTTCTGCGCGTAATCTGCTGCTTGCAAACAAAAAAACC
ACCGCTACCAGCGGTGGTTTGTTTGCCGGATCAAGAGCTACCAACTCTTTTTCCGAAGGTAACTGGCTTCAGCAGAGCGCAGATACCAAATACTGTC
CTTCTAGTGTAGCCGTAGTTAGGCCACCACTTCAAGAACTCTGTAGCACCGCCTACATACCTCGCTCTGCTAATCCTGTTACCAGTGGCTGCTGCCA
GTGGCGATAAGTCGTGTCTTACCGGGTTGGACTCAAGACGATAGTTACCGGATAAGGCGCAGCGGTCGGGCTGAACGGGGGGTTCGTGCACACAGCC
CAGCTTGGAGCGAACGACCTACACCGAACTGAGATACCTACAGCGTGAGCATTGAGAAAGCGCCACGCTTCCCGAAGGGAGAAAGGCGGACAGGTAT

CCGGTAAGCGGCAGGGTCGGAACAGGAGAGCGCACGAGGGAGCTTCCAGGGGGAAACGCCTGGTATCTTTATAGTCCTGTCGGGTTTCGCCACCTCT
GACTTGAGCGTCGATTTTTGTGATGCTCGTCAGGGGGGCGGAGCCTATGGAAAAACGCCAGCAACGCGAGCTCTAATACGACTCACTATAG

>ABc2-mCherry
ATGGGCGGCGCATGAGAGAAGCCCAGACCAATTACCTACCCAAAATGGAGAAAGTTCACGTTGACATCGAGGAAGACAGCCCATTCCTCAGAGCTTT
GCAGCGGAGCTTCCCGCAGTTTGAGGTAGAAGCCAAGCAGGTCACTGATAATGACCATGCTAATGCCAGAGCGTTTTCGCATCTGGCTTCAAAACTG
ATCGAAACGGAGGTGGACCCATCCGACACGATCCTTGACATTGGAAGTGCGCCCGCCCGCAGAATGTATTCTAAGCACAAGTATCATTGTATCTGTC
CGATGAGATGTGCGGAAGATCCGGACAGATTGTATAAGTATGCAACTAAGCTGAAGAAAAACTGTAAGGAAATAACTGATAAGGAATTGGACAAGAA
AATGAAGGAGCTGGCCGCCGTCATGAGCGACCCTGACCTGGAAACTGAGACTATGTGCCTCCACGACGACGAGTCGTGTCGCTACGAAGGGCAAGTC
GCTGTTTACCAGGATGTATACGCGGTTGACGGACCGACAAGTCTCTATCACCAAGCCAATAAGGGAGTTAGAGTCGCCTACTGGATAGGCTTTGACA
CCACCCCTTTTATGTTTAAGAACTTGGCTGGAGCATATCCATCATACTCTACCAACTGGGCCGACGAAACCGTGTTAACGGCTCGTAACATAGGCCT
ATGCAGCTCTGACGTTATGGAGCGGTCACGTAGAGGGATGTCCATTCTTAGAAAGAAGTATTTGAAACCATCCAACAATGTTCTATTCTCTGTTGGC
TCGACCATCTACCACGAGAAGAGGGACTTACTGAGGAGCTGGCACCTGCCGTCTGTATTTCACTTACGTGGCAAGCAAAATTACACATGTCGGTGTG
AGACTATAGTTAGTTGCGACGGGTACGTCGTTAAAAGAATAGCTATCAGTCCAGGCCTGTATGGGAAGCCTTCAGGCTATGCTGCTACGATGCACCG
CGAGGGATTCTTGTGCTGCAAAGTGACAGACACATTGAACGGGGAGAGGGTCTCTTTTCCCGTGTGCACGTATGTGCCAGCTACATTGTGTGACCAA
ATGACTGGCATACTGGCAACAGATGTCAGTGCGGACGACGCGCAAAAACTGCTGGTTGGGCTCAACCAGCGTATAGTCGTCAACGGTCGCACCCAGA
GAAACACCAATACCATGAAAAATTACCTTTTGCCCGTAGTGGCCCAGGCATTTGCTAGGTGGGCAAAGGAATATAAGGAAGATCAAGAAGATGAAAG
GCCACTAGGACTACGAGATAGACAGTTAGTCATGGGGTGTTGTTGGGCTTTTAGAAGGCACAAGATAACATCTATTTATAAGCGCCCGGATACCCAA
ACCATCATCAAAGTGAACAGCGATTTCCACTCATTCGTGCTGCCCAGGATAGGCAGTAACACATTGGAGATCGGGCTGAGAACAAGAATCAGGAAAA
TGTTAGAGGAGCACAAGGAGCCGTCACCTCTCATTACCGCCGAGGACGTACAAGAAGCTAAGTGCGCAGCCGATGAGGCTAAGGAGGTGCGTGAAGC
CGAGGAGTTGCGCGCAGCTCTACCACCTTTGGCAGCTGATGTTGAGGAGCCCACTCTGGAGGCAGACGTCGACTTGATGTTACAAGAGGCTGGGGCC
GGCTCAGTGGAGACACCTCGTGGCTTGATAAAGGTTACCAGCTACGATGGCGAGGACAAGATCGGCTCTTACGCTGTGCTTTCTCCGCAGGCTGTAC
TCAAGAGTGAAAAATTATCTTGCATCCACCCTCTCGCTGAACAAGTCATAGTGATAACACACTCTGGCCGAAAAGGGCGTTATGCCGTGGAACCATA
CCATGGTAAAGTAGTGGTGCCAGAGGGACATGCAATACCCGTCCAGGACTTTCAAGCTCTGAGTGAAAGTGCCACCATTGTGTACAACGAACGTGAG
TTCGTAAACAGGTACCTGCACCATATTGCCACACATGGAGGAGCGCTGAACACTGATGAAGAATATTACAAAACTGTCAAGCCCAGCGAGCACGACG
GCGAATACCTGTACGACATCGACAGGAAACAGTGCGTCAAGAAAGAACTAGTCACTGGGCTAGGGCTCACAGGCGAGCTGGTGGATCCTCCCTTCCA
TGAATTCGCCTACGAGAGTCTGAGAACACGACCAGCCGCTCCTTACCAAGTACCAACCATAGGGGTGTATGGCGTGCCAGGATCAGGCAAGTCTGGC
ATCATTAAAAGCGCAGTCACCAAAAAAGATCTAGTGGTGAGCGCCAAGAAAGAAAACTGTGCAGAAATTATAAGGGACGTCAAGAAAATGAAAGGGC
TGGACGTCAATGCCAGAACTGTGGACTCAGTGCTCTTGAATGGATGCAAACACCCCGTAGAGACCCTGTATATTGACGAAGCTTTTGCTTGTCATGC
AGGTACTCTCAGAGCGCTCATAGCCATTATAAGACCTAAAAAGGCAGTGCTCTGCGGGGATCCCAAACAGTGCGGTTTTTTTAACATGATGTGCCTG
AAAGTGCATTTTAACCACGAGATTTGCACACAAGTCTTCCACAAAAGCATCTCTCGCCGTTGCACTAAATCTGTGACTTCGGTCGTCTCAACCTTGT
TTTACGACAAAAAAATGAGAACGACGAATCCGAAAGAGACTAAGATTGTGATTGACACTACCGGCAGTACCAAACCTAAGCAGGACGATCTCATTCT
CACTTGTTTCAGAGGGTGGGTGAAGCAGTTGCAAATAGATTACAAAGGCAACGAAATAATGACGGCAGCTGCCTCTCAAGGGCTGACCCGTAAAGGT
GTGTATGCCGTTCGGTACAAGGTGAATGAAAATCCTCTGTACGCACCCACCTCAGAACATGTGAACGTCCTACTGACCCGCACGGAGGACCGCATCG
TGTGGAAAACACTAGCCGGCGACCCATGGATAAAAACACTGACTGCCAAGTACCCTGGGAATTTCACTGCCACGATAGAGGAGTGGCAAGCAGAGCA
TGATGCCATCATGAGGCACATCTTGGAGAGACCGGACCCTACCGACGTCTTCCAGAATAAGGCAAACGTGTGTTGGGCCAAGGCTTTAGTGCCGGTG
CTGAAGACCGCTGGCATAGACATGACCACTGAACAATGGAACACTGTGGATTATTTTGAAACGGACAAAGCTCACTCAGCAGAGATAGTATTGAACC
AACTATGCGTGAGGTTCTTTGGACTCGATCTGGACTCCGGTCTATTTTCTGCACCCACTGTTCCGTTATCCATTAGGAATAATCACTGGGATAACTC
CCCGTCGCCTAACATGTACGGGCTGAATAAAGAAGTGGTCCGTCAGCTCTCTCGCAGGTACCCACAACTGCCTCGGGCAGTTGCCACTGGAAGAGTC
TATGACATGAACACTGGTACACTGCGCAATTATGATCCGCGCATAAACCTAGTACCTGTAAACAGAAGACTGCCTCATGCTTTAGTCCTCCACCATA
ATGAACACCCACAGAGTGACTTTTCTTCATTCGTCAGCAAATTGAAGGGCAGAACTGTCCTGGTGGTCGGGGAAAAGTTGTCCGTCCCAGGCAAAAT
GGTTGACTGGTTGTCAGACCGGCCTGAGGCTACCTTCAGAGCTCGGCTGGATTTAGGCATCCCAGGTGATGTGCCCAAATATGACATAATATTTGTT
AATGTGAGGACCCCATATAAATACCATCACTATCAGCAGTGTGAAGACCATGCCATTAAGCTTAGCATGTTGACCAAGAAAGCTTGTCTGCATCTGA
ATCCCGGCGGAACCTGTGTCAGCATAGGTTATGGTTACGCTGACAGGGCCAGCGAAAGCATCATTGGTGCTATAGCGCGGCAGTTCAAGTTTTCCCG
GGTATGCAAACCGAAATCCTCACTTGAAGAGACGGAAGTTCTGTTTGTATTCATTGGGTACGATCGCAAGGCCCGTACGCACAATTCTTACAAGCTT
TCATCAACCTTGACCAACATTTATACAGGTTCCAGACTCCACGAAGCCGGATGTGCACCCTCATATCATGTGGTGCGAGGGGATATTGCCACGGCCA
CCGAAGGAGTGATTATAAATGCTGCTAACAGCAAAGGACAACCTGGCGGAGGGGTGTGCGGAGCGCTGTATAAGAAATTCCCGGAAAGCTTCGATTT
ACAGCCGATCGAAGTAGGAAAAGCGCGACTGGTCAAAGGTGCAGCTAAACATATCATTCATGCCGTAGGACCAAACTTCAACAAAGTTTCGGAGGTT
GAAGGTGACAAACAGTTGGCAGAGGCTTATGAGTCCATCGCTAAGATTGTCAACGATAACAATTACAAGTCAGTAGCGATTCCACTGTTGTCCACCG
GCATCTTTTCCGGGAACAAAGATCGACTAACCCAATCATTGAACCATTTGCTGACAGCTTTAGACACCACTGATGCAGATGTAGCCATATACTGCAG
GGACAAGAAATGGGAAATGACTCTCAAGGAAGCAGTGGCTAGGAGAGAAGCAGTGGAGGAGATATGCATATCCGACGACTCTTCAGTGACAGAACCT
GATGCAGAGCTGGTGAGGGTGCATCCGAAGAGTTCTTTGGCTGGAAGGAAGGGCTACAGCACAAGCGATGGCAAAACTTTCTCATATTTGGAAGGGA
CCAAGTTTCACCAGGCGGCCAAGGATATAGCAGAAATTAATGCCATGTGGCCCGTTGCAACGGAGGCCAATGAGCAGGTATGCATGTATATCCTCGG
AGAAAGCATGAGCAGTATTAGGTCGAAATGCCCCGTCGAAGATTCGGAAGCCTCCACACCACCTAGCACGCTGCCTTGCTTGTGCATCCATGCCATG
ACTCCAGAAAGAGTACAGCGCCTAAAAGCCTCACGTCCAGAACAAATTACTGTGTGCTCATCCTTTCCATTGCCGAAGTATAGAATCACTGGTATGC
AGAAGATCCAATGCTCCCAGCCTATATTGTTCTCACCGAAAGTGCCTGCGTATATTCATCCAAGGAAGTATCTCGTGGAAACACCACCGGTAGACGA
GACTCCGGAGCCATCGGCAGAGAACCAATCCACAGAGGGGACACCTGAACAACCACCACTTATAACCGAGGATGAGACCAGGACTAGAACGCCTGAG
CCGATCATCATCGAAGAGGAAGAAGAGGATAGCATAAGTTTGCTGTCAGATGGCCCGACCCACCAGGTGCTGCAAGTCGAGGCAGACATTCACGGGC
CGCCCTCTGTATCTAGCTCATCCTGGTCCATTCCTCATGCATCCGACTTTGATGTGGACAGTTTATCCATACTTGACACCCTGGAGGGAGCTAGCGT
GACCAGCGGGGCAACGTCAGCCGAGACTAACTCTTACTTCGCAAAGAGTATGGAGTTTCTGGCGCGACCGGTGCCTGCGCCTCGAACAGTATTCAGG
AACCCTCCACATCCCGCTCCGCGCACAAGAACACCGTCACTTGCACCCAGCAGGGCCTGCTCGAGAACCAGCCTAGTTTCCACCCCGCCAGGCGTGA
ATAGGGTGATCACTAGAGAGGAGCTCGAGGCGCTTACCCCGTCACGCACTCCTAGCAGGTCGGTCTCGAGAACCAGCCTGGTCTCCAACCCGCCAGG
CGTAAATAGGGTGATTACAAGAGAGGAGTTTGAGGCGTTCGTAGCACAACAACAATGACGGTTTGATGCGGGTGCATACATCTTTTCCTCCGACACC
GGTCAAGGGCATTTACAACAAAAATCAGTAAGGCAAACGGTGCTATCCGAAGTGGTGTTGGAGAGGACCGAATTGGAGATTTCGTATGCCCCGCGCC
TCGACCAAGAAAAAGAAGAATTACTACGCAAGAAATTACAGTTAAATCCCACACCTGCTAACAGAAGCAGATACCAGTCCAGGAAGGTGGAGAACAT
GAAAGCCATAACAGCTAGACGTATTCTGCAAGGCCTAGGGCATTATTTGAAGGCAGAAGGAAAAGTGGAGTGCTACCGAACCCTGCATCCTGTTCCT
TTGTATTCATCTAGTGTGAACCGTGCCTTTTCAAGCCCCAAGGTCGCAGTGGAAGCCTGTAACGCCATGTTGAAAGAGAACTTTCCGACTGTGGCTT
CTTACTGTATTATTCCAGAGTACGATGCCTATTTGGACATGGTTGACGGAGCTTCATGCTGCTTAGACACTGCCAGTTTTTGCCCTGCAAAGCTGCG
CAGCTTTCCAAAGAAACACTCCTATTTGGAACCCACAATACGATCGGCAGTGCCTTCAGCGATCCAGAACACGCTCCAGAACGTCCTGGCAGCTGCC
ACAAAAAGAAATTGCAATGTCACGCAAATGAGAGAATTGCCCGTATTGGATTCGGCGGCCTTTAATGTGGAATGCTTCAAGAAATATGCGTGTAATA
ATGAATATTGGGAAACGTTTAAAGAAAACCCCATCAGGCTTACTGAAGAAAACGTGGTAAATTACATTACCAAATTAAAAGGACCAAAAGCTGCTGC
TCTTTTTGCGAAGACACATAATTTGAATATGTTGCAGGACATACCAATGGACAGGTTTGTAATGGACTTAAAGAGAGACGTGAAAGTGACTCCAGGA

ACAAAACATACTGAAGAACGGCCCAAGGTACAGGTGATCCAGGCTGCCGATCCGCTAGCAACAGCGTATCTGTGCGGAATCCACCGAGAGCTGGTTA
GGAGATTAAATGCGGTCCTGCTTCCGAACATTCATACACTGTTTGATATGTCGGCTGAAGACTTTGACGCTATTATAGCCGAGCACTTCCAGCCTGG
GGATTGTGTTCTGGAAACTGACATCGCGTCGTTTGATAAAAGTGAGGACGACGCCATGGCTCTGACCGCGTTAATGATTCTGGAAGACTTAGGTGTG
GACGCAGAGCTGTTGACGCTGATTGAGGCGGCTTTCGGCGAAATTTCATCAATACATTTGCCCACTAAAACTAAATTTAAATTCGGAGCCATGATGA
AATCTGGAATGTTCCTCACACTGTTTGTGAACACAGTCATTAACATTGTAATCGCAAGCAGAGTGTTGAGAGAACGGCTAACCGGATCACCATGTGC
AGCATTCATTGGAGATGACAATATCGTGAAAGGAGTCAAATCGGACAAATTAATGGCAGACAGGTGCGCCACCTGGTTGAATATGGAAGTCAAGATT
ATAGATGCTGTGGTGGGCGAGAAAGCGCCTTATTTCTGTGGAGGGTTTATTTTGTGTGACTCCGTGACCGGCACAGCGTGCCGTGTGGCAGACCCCC
TAAAAAGGCTGTTTAAGCTTGGCAAACCTCTGGCAGCAGACGATGAACATGATGATGACAGGAGAAGGGCATTGCATGAAGAGTCAACACGCTGGAA
CCGAGTGGGTATTCTTTCAGAGCTGTGCAAGGCAGTAGAATCAAGGTATGAAACCGTAGGAACTTCCATCATAGTTATGGCCATGACTACTCTAGCT
AGCAGTGTTAAATCATTCAGCTACCTGAGAGGGGCCCCTATAACTCTCTACGGCTAACCTGAATGGACTACGACATAGTCTAGTCCGCCAAGTCTAG
catatgggcgcgccctcagcatcgattgaattggccaccATGGTGAGCAAGGGCGAGGAGGATAACATGGCCATCATCAAGGAGTTCATGCGCTTCA
AGGTGCACATGGAGGGCTCCGTGAACGGCCACGAGTTCGAGATCGAGGGCGAGGGCGAGGGCCGCCCCTACGAGGGCACCCAGACCGCCAAGCTGAA
GGTGACCAAGGGTGGCCCCCTGCCCTTCGCCTGGGACATCCTGTCCCCTCAGTTCATGTACGGCTCCAAGGCCTACGTGAAGCACCCCGCCGACATC
CCCGACTACTTGAAGCTGTCCTTCCCCGAGGGCTTCAAGTGGGAGCGCGTGATGAACTTCGAGGACGGCGGCGTGGTGACCGTGACCCAGGACTCCT
CCCTGCAGGACGGCGAGTTCATCTACAAGGTGAAGCTGCGCGGCACCAACTTCCCCTCCGACGGCCCCGTAATGCAGAAGAAGACCATGGGCTGGGA
GGCCTCCTCCGAGCGGATGTACCCCGAGGACGGCGCCCTGAAGGGCGAGATCAAGCAGAGGCTGAAGCTGAAGGACGGCGGCCACTACGACGCTGAG
GTCAAGACCACCTACAAGGCCAAGAAGCCCGTGCAGCTGCCCGGCGCCTACAACGTCAACATCAAGTTGGACATCACCTCCCACAACGAGGACTACA
CCATCGTGGAACAGTACGAACGCGCCGAGGGCCGCCACTCCACCGGCGGCATGGACGAGCTGTACAAGTAGgaattGGCAAGCTGCTTACATAGAAC
TCGCGGCGATTGGCATGCCGCCTTAAAATTTTTATTTTATTTTTCTTTTCTTTTCCGAATCGGATTTTGTTTTTAATATTTCAAAAAAAAAAAAAAA
AAAAAAAAAAACGCGTCGAGGGGAATTAATTCTTGAAGACGAAAGGGCCAGGTGGCACTTTTCGGGGAAATGTGCGCGGAACCCCTATTTGTTTATT
TTTCTAAATACATTCAAATATGTATCCGCTCATGAGACAATAACCCTGATAAATGCTTCAATAATATTGAAAAAGGAAGAGTATGAGTATTCAACAT
TTCCGTGTCGCCCTTATTCCCTTTTTTGCGGCATTTTGCCTTCCTGTTTTTGCTCACCCAGAAACGCTGGTGAAAGTAAAAGATGCTGAAGATCAGT
TGGGTGCACGAGTGGGTTACATCGAACTGGATCTCAACAGCGGTAAGATCCTTGAGAGTTTTCGCCCCGAAGAACGTTTTCCAATGATGAGCACTTT
TAAAGTTCTGCTATGTGGCGCGGTATTATCCCGTGTTGACGCCGGGCAAGAGCAACTCGGTCGCCGCATACACTATTCTCAGAATGACTTGGTTGAG
TACTCACCAGTCACAGAAAAGCATCTTACGGATGGCATGACAGTAAGAGAATTATGCAGTGCTGCCATAACCATGAGTGATAACACTGCGGCCAACT
TACTTCTGACAACGATCGGAGGACCGAAGGAGCTAACCGCTTTTTTGCACAACATGGGGGATCATGTAACTCGCCTTGATCGTTGGGAACCGGAGCT
GAATGAAGCCATACCAAACGACGAGCGTGACACCACGATGCCTGTAGCAATGGCAACAACGTTGCGCAAACTATTAACTGGCGAACTACTTACTCTA
GCTTCCCGGCAACAATTAATAGACTGGATGGAGGCGGATAAAGTTGCAGGACCACTTCTGCGCTCGGCCCTTCCGGCTGGCTGGTTTATTGCTGATA
AATCTGGAGCCGGTGAGCGTGGGTCTCGCGGTATCATTGCAGCACTGGGGCCAGATGGTAAGCCCTCCCGTATCGTAGTTATCTACACGACGGGGAG
TCAGGCAACTATGGATGAACGAAATAGACAGATCGCTGAGATAGGTGCCTCACTGATTAAGCATTGGTAACTGTCAGACCAAGTTTACTCATATATA
CTTTAGATTGATTTAAAACTTCATTTTTAATTTAAAAGGATCTAGGTGAAGATCCTTTTTGATAATCTCATGACCAAAATCCCTTAACGTGAGTTTT
CGTTCCACTGAGCGTCAGACCCCGTAGAAAAGATCAAAGGATCTTCTTGAGATCCTTTTTTTCTGCGCGTAATCTGCTGCTTGCAAACAAAAAAACC
ACCGCTACCAGCGGTGGTTTGTTTGCCGGATCAAGAGCTACCAACTCTTTTTCCGAAGGTAACTGGCTTCAGCAGAGCGCAGATACCAAATACTGTC
CTTCTAGTGTAGCCGTAGTTAGGCCACCACTTCAAGAACTCTGTAGCACCGCCTACATACCTCGCTCTGCTAATCCTGTTACCAGTGGCTGCTGCCA
GTGGCGATAAGTCGTGTCTTACCGGGTTGGACTCAAGACGATAGTTACCGGATAAGGCGCAGCGGTCGGGCTGAACGGGGGGTTCGTGCACACAGCC
CAGCTTGGAGCGAACGACCTACACCGAACTGAGATACCTACAGCGTGAGCATTGAGAAAGCGCCACGCTTCCCGAAGGGAGAAAGGCGGACAGGTAT
CCGGTAAGCGGCAGGGTCGGAACAGGAGAGCGCACGAGGGAGCTTCCAGGGGGAAACGCCTGGTATCTTTATAGTCCTGTCGGGTTTCGCCACCTCT
GACTTGAGCGTCGATTTTTGTGATGCTCGTCAGGGGGGCGGAGCCTATGGAAAAACGCCAGCAACGCGAGCTCTAATACGACTCACTATAG

>aBc2-mCherry
ATGGGCGGCGCATGAGAGAAGCCCAGACCAATTACCTACCCAAAATGGAGAAAGTTCACGTTGACATCGAGGAAGACAGCCCATTCCTCAGAGCTTT
GCAGCGGAGCTTCCCGCAGTTTGAGGTAGAAGCCAAGCAGGTCACTGATAATGACCATGCTAATGCCAGAGCGTTTTCGCATCTGGCTTCAAAACTG
ATCGAAACGGAGGTGGACCCATCCGACACGATCCTTGACATTGGAAGTGCGCCCGCCCGCAGAATGTATTCTAAGCACAAGTATCATTGTATCTGTC
CGATGAGATGTGCGGAAGATCCGGACAGATTGTATAAGTATGCAACTAAGCTGAAGAAAAACTGTAAGGAAATAACTGATAAGGAATTGGACAAGAA
AATGAAGGAGCTGGCCGCCGTCATGAGCGACCCTGACCTGGAAACTGAGACTATGTGCCTCCACGACGACGAGTCGTGTCGCTACGAAGGGCAAGTC
GCTGTTTACCAGGATGTATACGCGGTTGACGGACCGACAAGTCTCTATCACCAAGCCAATAAGGGAGTTAGAGTCGCCTACTGGATAGGCTTTGACA
CCACCCCTTTTATGTTTAAGAACTTGGCTGGAGCATATCCATCATACTCTACCAACTGGGCCGACGAAACCGTGTTAACGGCTCGTAACATAGGCCT
ATGCAGCTCTGACGTTATGGAGCGGTCACGTAGAGGGATGTCCATTCTTAGAAAGAAGTATTTGAAACCATCCAACAATGTTCTATTCTCTGTTGGC
TCGACCATCTACCACGAGAAGAGGGACTTACTGAGGAGCTGGCACCTGCCGTCTGTATTTCACTTACGTGGCAAGCAAAATTACACATGTCGGTGTG
AGACTATAGTTAGTTGCGACGGGTACGTCGTTAAAAGAATAGCTATCAGTCCAGGCCTGTATGGGAAGCCTTCAGGCTATGCTGCTACGATGCACCG
CGAGGGATTCTTGTGCTGCAAAGTGACAGACACATTGAACGGGGAGAGGGTCTCTTTTCCCGTGTGCACGTATGTGCCAGCTACATTGTGTGACCAA
ATGACTGGCATACTGGCAACAGATGTCAGTGCGGACGACGCGCAAAAACTGCTGGTTGGGCTCAACCAGCGTATAGTCGTCAACGGTCGCACCCAGA
GAAACACCAATACCATGAAAAATTACCTTTTGCCCGTAGTGGCCCAGGCATTTGCTAGGTGGGCAAAGGAATATAAGGAAGATCAAGAAGATGAAAG
GCCACTAGGACTACGAGATAGACAGTTAGTCATGGGGTGTTGTTGGGCTTTTAGAAGGCACAAGATAACATCTATTTATAAGCGCCCGGATACCCAA
ACCATCATCAAAGTGAACAGCGATTTCCACTCATTCGTGCTGCCCAGGATAGGCAGTAACACATTGGAGATCGGGCTGAGAACAAGAATCAGGAAAA
TGTTAGAGGAGCACAAGGAGCCGTCACCTCTCATTACCGCCGAGGACGTACAAGAAGCTAAGTGCGCAGCCGATGAGGCTAAGGAGGTGCGTGAAGC
CGAGGAGTTGCGCGCAGCTCTACCACCTTTGGCAGCTGATGTTGAGGAGCCCACTCTGGAGGCAGACGTCGACTTGATGTTACAAGAGGCTGGGGCC
GGCTCAGTGGAGACACCTCGTGGCTTGATAAAGGTTACCAGCTACGATGGCGAGGACAAGATCGGCTCTTACGCTGTGCTTTCTCCGCAGGCTGTAC
TCAAGAGTGAAAAATTATCTTGCATCCACCCTCTCGCTGAACAAGTCATAGTGATAACACACTCTGGCCGAAAAGGGCGTTATGCCGTGGAACCATA
CCATGGTAAAGTAGTGGTGCCAGAGGGACATGCAATACCCGTCCAGGACTTTCAAGCTCTGAGTGAAAGTGCCACCATTGTGTACAACGAACGTGAG
TTCGTAAACAGGTACCTGCACCATATTGCCACACATGGGGGAGCGCTGAACACTGATGAAGAATATTACAAAACTGTCAAGCCCAGCGAGCACGACG
GCGAATACCTGTACGACATCGACAGGAAACAGTGCGTCAAGAAAGAACTAGTCACTGGGCTAGGGCTCACAGGCGAGCTGGTGGATCCTCCCTTCCA
TGAATTCGCCTACGAGAGTCTGAGAACACGACCAGCCGCTCCTTACCAAGTACCAACCATAGGGGTGTATGGCGTGCCAGGATCAGGCAAGTCTGGC
ATCATTAAAAGCGCAGTCACCAAAAAAGATCTAGTGGTGAGCGCCAAGAAAGAAAACTGTGCAGAAATTATAAGGGACGTCAAGAAAATGAAAGGGC
TGGACGTCAATGCCAGAACTGTGGACTCAGTGCTCTTGAATGGATGCAAACACCCCGTAGAGACCCTGTATATTGACGAAGCTTTTGCTTGTCATGC
AGGTACTCTCAGAGCGCTCATAGCCATTATAAGACCTAAAAAGGCAGTGCTCTGCGGGGATCCCAAACAGTGCGGTTTTTTAACATGATGTGCCTG
AAAGTGCATTTTAACCACGAGATTTGCACACAAGTCTTCCACAAAAGCATCTCTCGCCGTTGCACTAAATCTGTGACTTCGGTCGTCTCAACCTTGT
TTTACGACAAAAAAATGAGAACGACGAATCCGAAAGAGACTAAGATTGTGATTGACACTACCGGCAGTACCAAACCTAAGCAGGACGATCTCATTCT
CACTTGTTTCAGAGGGTGGGTGAAGCAGTTGCAAATAGATTACAAAGGCAACGAAATAATGACGGCAGCTGCCTCTCAAGGGCTGACCCGTAAAGGT
GTGTATGCCGTTCGGTACAAGGTGAATGAAAATCCTCTGTACGCACCCACCTCAGAACATGTGAACGTCCTACTGACCCGCACGGAGGACCGCATCG
TGTGGAAAACACTAGCCGGCGACCCATGGATAAAAACACTGACTGCCAAGTACCCTGGGAATTTCACTGCCACGATAGAGGAGTGGCAAGCAGAGCA
TGATGCCATCATGAGGCACATCTTGGAGAGACCGGACCCTACCGACGTCTTCCAGAATAAGGCAAACGTGTGTTGGGCCAAGGCTTTAGTGCCGGTG

```
CTGAAGACCGCTGGCATAGACATGACCACTGAACAATGGAACACTGTGGATTATTTTGAAACGGACAAAGCTCACTCAGCAGAGATAGTATTGAACC
AACTATGCGTGAGGTTCTTTGGACTCGATCTGGACTCCGGTCTATTTTCTGCACCCACTGTTCCGTTATCCATTAGGAATAATCACTGGGATAACTC
CCCGTCGCCTAACATGTACGGGCTGAATAAAGAAGTGGTCCGTCAGCTCTCTCGCAGGTACCCACAACTGCCTCGGGCAGTTGCCACTGGAAGAGTC
TATGACATGAACACTGGTACACTGCGCAATTATGATCCGCGCATAAACCTAGTACCTGTAAACAGAAGACTGCCTCATGCTTTAGTCCTCCACCATA
ATGAACACCCACAGAGTGACTTTTCTTCATTCGTCAGCAAATTGAAGGGCAGAACTGTCCTGGTGGTCGGGGAAAAGTTGTCCGTCCCAGGCAAAAT
GGTTGACTGGTTGTCAGACCGGCCTGAGGCTACCTTCAGAGCTCGGCTGGATTTAGGCATCCCAGGTGATGTGCCCAAATATGACATAATATTTGTT
AATGTGAGGACCCCATATAAATACCATCACTATCAGCAGTGTGAAGACCATGCCATTAAGCTTAGCATGTTGACCAAGAAAGCTTGTCTGCATCTGA
ATCCCGGCGGAACCTGTGTCAGCATAGGTTATGGTTACGCTGACAGGGCCAGCGAAAGCATCATTGGTGCTATAGCGCGGCAGTTCAAGTTTTCCCG
GGTATGCAAACCGAAATCCTCACTTGAAGAGACGGAAGTTCTGTTTGTATTCATTGGGTACGATCGCAAGGCCCGTACGCACAATTCTTACAAGCTT
TCATCAACCTTGACCAACATTTATACAGGTTCCAGACTCCACGAAGCCGGATGTGCACCCTCATATCATGTGGTGCGAGGGGATATTGCCACGGCCA
CCGAAGGAGTGATTATAAATGCTGCTAACAGCAAAGGACAACCTGGCGGAGGGGTGTGCGGAGCGCTGTATAAGAAATTCCCGGAAAGCTTCGATTT
ACAGCCGATCGAAGTAGGAAAAGCGCGACTGGTCAAAGGTGCAGCTAAACATATCATTCATGCCGTAGGACCAAACTTCAACAAAGTTTCGGAGGTT
GAAGGTGACAAACAGTTGGCAGAGGCTTATGAGTCCATCGCTAAGATTGTCAACGATAACAATTACAAGTCAGTAGCGATTCCACTGTTGTCCACCG
GCATCTTTTCCGGGAACAAAGATCGACTAACCCAATCATTGAACCATTTGCTGACAGCTTTAGACACCACTGATGCAGATGTAGCCATATACTGCAG
GGACAAGAAATGGGAAATGACTCTCAAGGAAGCAGTGGCTAGGAGAGAAGCAGTGGAGGAGATATGCATATCCGACGACTCTTCAGTGACAGAACCT
GATGCAGAGCTGGTGAGGGTGCATCCGAAGAGTTCTTTGGCTGGAAGGAAGGGCTACAGCACAAGCGATGGCAAAACTTTCTCATATTTGGAAGGGA
CCAAGTTTCACCAGGCGGCCAAGGATATAGCAGAAATTAATGCCATGTGGCCCGTTGCAACGGAGGCCAATGAGCAGGTATGCATGTATATCCTCGG
AGAAAGCATGAGCAGTATTAGGTCGAAATGCCCCGTCGAAGATTCGGAAGCCTCCACACCACCTAGCACGCTGCCTTGCTTGTGCATCCATGCCATG
ACTCCAGAAAGAGTACAGCGCCTAAAAGCCTCACGTCCAGAACAAATTACTGTGTGCTCATCCTTTCCATTGCCGAAGTATAGAATCACTGGTATGC
AGAAGATCCAATGCTCCCAGCCTATATTGTTCTCACCGAAAGTGCCTGCGTATATTCATCCAAGGAAGTATCTCGTGGAAACACCACCGGTAGACGA
GACTCCGGAGCCATCGGCAGAGAACCAATCCACAGAGGGGACACCTGAACAACCACCACTTATAACCGAGGATGAGACCAGGACTAGAACGCCTGAG
CCGATCATCATCGAAGAGGAAGAAGAGGATAGCATAAGTTTGCTGTCAGATGGCCCGACCCACCAGGTGCTGCAAGTCGAGGCAGACATTCACGGGC
CGCCCTCTGTATCTAGCTCATCCTGGTCCATTCCTCATGCATCCGACTTTGATGTGGACAGTTTATCCATACTTGACACCCTGGAGGGAGCTAGCGT
GACCAGCGGGGCAACGTCAGCCGAGACTAACTCTTACTTCGCAAAGAGTATGGAGTTTCTGGCGCGACCGGTGCCTGCGCCTCGAACAGTATTCAGG
AACCCTCCACATCCCGCTCCGCGCACAAGAACACCGTCACTTGCACCCAGCAGGGCCTGCTCGAGAACCAGCCTAGTTTCCACCCCGCCAGGCGTGA
ATAGGGTGATCACTAGAGAGGAGCTCGAGGCGCTTACCCCGTCACGCACTCCTAGCAGGTCGGTCTCGAGAACCAGCCTGGTCTCCAACCCGCCAGG
CGTAAATAGGGTGATTACAAGAGAGGAGTTTGAGGCGTTCGTAGCACAACAACAATGACGGTTTGATGCGGGTGCATACATCTTTTCCTCCGACACC
GGTCAAGGGCATTTACAACAAAAATCAGTAAGGCAAACGGTGCTATCCGAAGTGGTGTTGGAGAGGACCGAATTGGAGATTTCGTATGCCCCGCGCC
TCGACCAAGAAAAAGAAGAATTACTACGCAAGAAATTACAGTTAAATCCCACACCTGCTAACAGAAGCAGATACCAGTCCAGGAAGGTGGAGAACAT
GAAAGCCATAACAGCTAGACGTATTCTGCAAGGCCTAGGGCATTATTTGAAGGCAGAAGGAAAAGTGGAGTGCTACCGAACCCTGCATCCTGTTCCT
TTGTATTCATCTAGTGTGAACCGTGCCTTTTCAAGCCCCAAGGTCGCAGTGGAAGCCTGTAACGCCATGTTGAAAGAGAACTTTCCGACTGTGGCTT
CTTACTGTATTATTCCAGAGTACGATGCCTATTTGGACATGGTTGACGGAGCTTCATGCTGCTTAGACACTGCCAGTTTTTGCCCTGCAAAGCTGCG
CAGCTTTCCAAAGAAACACTCCTATTTGGAACCCACAATACGATCGGCAGTGCCTTCAGCGATCCAGAACACGCTCCAGAACGTCCTGGCAGCTGCC
ACAAAAAGAAATTGCAATGTCACGCAAATGAGAGAATTGCCCGTATTGGATTCGGCGGCCTTTAATGTGGAATGCTTCAAGAAATATGCGTGTAATA
ATGAATATTGGGAAACGTTTAAAGAAAACCCCATCAGGCTTACTGAAGAAAACGTGGTAAATTACATTACCAAATTAAAAGGACCAAAAGCTGCTGC
TCTTTTTGCGAAGACACATAATTTGAATATGTTGCAGGACATACCAATGGACAGGTTTGTAATGGACTTAAAGAGAGACGTGAAAGTGACTCCAGGA
ACAAAACATACTGAAGAACGGCCCAAGGTACAGGTGATCCAGGCTGCCGATCCGCTAGCAACAGCGTATCTGTGCGGAATCCACCGAGAGCTGGTTA
GGAGATTAAATGCGGTCCTGCTTCCGAACATTCATACACTGTTTGATATGTCGGCTGAAGACTTTGACGCTATTATAGCCGAGCACTTCCAGCCTGG
GGATTGTGTTCTGGAAACTGACATCGCGTCGTTTGATAAAAGTGAGGACGACGCCATGGCTCTGACCGCGTTAATGATTCTGGAAGACTTAGGTGTG
GACGCAGAGCTGTTGACGCTGATTGAGGCGGCTTTCGGCGAAATTTCATCAATACATTTGCCCACTAAAACTAAATTTAAATTCGGAGCCATGATGA
AATCTGGAATGTTCCTCACACTGTTTGTGAACACAGTCATTAACATTGTAATCGCAAGCAGAGTGTTGAGAGAACGGCTAACCGGATCACCATGTGC
AGCATTCATTGGAGATGACAATATCGTGAAAGGAGTCAAATCGGACAAATTAATGGCAGACAGGTGCGCCACCTGGTTGAATATGGAAGTCAAGATT
ATAGATGCTGTGGTGGGCGAGAAAGCGCCTTATTTCTGTGGAGGGTTTATTTTGTGTGACTCCGTGACCGGCACAGCGTGCCGTGTGGCAGACCCCC
TAAAAAGGCTGTTTAAGCTTGGCAAACCTCTGGCAGCAGACGATGAACATGATGATGACAGGAGAAGGGCATTGCATGAAGAGTCAACACGCTGGAA
CCGAGTGGGTATTCTTTCAGAGCTGTGCAAGGCAGTAGAATCAAGGTATGAAACCGTAGGAACTTCCATCATAGTTATGGCCATGACTACTCTAGCT
AGCAGTGTTAAATCATTCAGCTACCTGAGAGGGGCCCCTATAACTCTCTACGGCTAACCTGAATGGACTACGACATAGTCTAGTCCGCCAAGTCTAG
catatgggcgcgccctcagcatcgattgaattggccaccATGGTGAGCAAGGGCGAGGAGGATAACATGGCCATCATCAAGGAGTTCATGCGCTTCA
AGGTGCACATGGAGGGCTCCGTGAACGGCCACGAGTTCGAGATCGAGGGCGAGGGCGAGGGCCGCCCCTACGAGGGCACCCAGACCGCCAAGCTGAA
GGTGACCAAGGGTGGCCCCCTGCCCTTCGCCTGGGACATCCTGTCCCCTCAGTTCATGTACGGCTCCAAGGCCTACGTGAAGCACCCCGCCGACATC
CCCGACTACTTGAAGCTGTCCTTCCCCGAGGGCTTCAAGTGGGAGCGCGTGATGAACTTCGAGGACGGCGGCGTGGTGACCGTGACCCAGGACTCCT
CCCTGCAGGACGGCGAGTTCATCTACAAGGTGAAGCTGCGCGGCACCAACTTCCCCTCCGACGGCCCCGTAATGCAGAAGAAGACCATGGGCTGGGA
GGCCTCCTCCGAGCGGATGTACCCCGAGGACGGCGCCCTGAAGGGCGAGATCAAGCAGAGGCTGAAGCTGAAGGACGGCGGCCACTACGACGCTGAG
GTCAAGACCACCTACAAGGCCAAGAAGCCCGTGCAGCTGCCCGGCGCCTACAACGTCAACATCAAGTTGGACATCACCTCCCACAACGAGGACTACA
CCATCGTGGAACAGTACGAACGCGCCGAGGGCCGCCACTCCACCGGCGGCATGGACGAGCTGTACAAGTAGgaattGGCAAGCTGCTTACATAGAAC
TCGCGGCGATTGGCATGCCGCCTTAAAATTTTTATTTTATTTTTCTTTTCTTTTCCGAATCGGATTTTGTTTTTAATATTTCAAAAAAAAAAAAAAA
AAAAAAAAAAACGCGTCGAGGGGAATTAATTCTTGAAGACGAAAGGGCCAGGTGGCACTTTTCGGGGAAATGTGCGCGGAACCCCTATTTGTTTATT
TTTCTAAATACATTCAAATATGTATCCGCTCATGAGACAATAACCCTGATAAATGCTTCAATAATATTGAAAAAGGAAGAGTATGAGTATTCAACAT
TTCCGTGTCGCCCTTATTCCCTTTTTTGCGGCATTTTGCCTTCCTGTTTTTGCTCACCCAGAAACGCTGGTGAAAGTAAAAGATGCTGAAGATCAGT
TGGGTGCACGAGTGGGTTACATCGAACTGGATCTCAACAGCGGTAAGATCCTTGAGAGTTTTCGCCCCGAAGAACGTTTTCCAATGATGAGCACTTT
TAAAGTTCTGCTATGTGGCGCGGTATTATCCCGTGTTGACGCCGGGCAAGAGCAACTCGGTCGCCGCATACACTATTCTCAGAATGACTTGGTTGAG
TACTCACCAGTCACAGAAAAGCATCTTACGGATGGCATGACAGTAAGAGAATTATGCAGTGCTGCCATAACCATGAGTGATAACACTGCGGCCAACT
TACTTCTGACAACGATCGGAGGACCGAAGGAGCTAACCGCTTTTTTGCACAACATGGGGGATCATGTAACTCGCCTTGATCGTTGGGAACCGGAGCT
GAATGAAGCCATACCAAACGACGAGCGTGACACCACGATGCCTGTAGCAATGGCAACAACGTTGCGCAAACTATTAACTGGCGAACTACTTACTCTA
GCTTCCCGGCAACAATTAATAGACTGGATGGAGGCGGATAAAGTTGCAGGACCACTTCTGCGCTCGGCCCTTCCGGCTGGCTGGTTTATTGCTGATA
AATCTGGAGCCGGTGAGCGTGGGTCTCGCGGTATCATTGCAGCACTGGGGCCAGATGGTAAGCCCTCCCGTATCGTAGTTATCTACACGACGGGGAG
TCAGGCAACTATGGATGAACGAAATAGACAGATCGCTGAGATAGGTGCCTCACTGATTAAGCATTGGTAACTGTCAGACCAAGTTTACTCATATATA
CTTTAGATTGATTTAAAACTTCATTTTTAATTTAAAAGGATCTAGGTGAAGATCCTTTTTGATAATCTCATGACCAAAATCCCTTAACGTGAGTTTT
CGTTCCACTGAGCGTCAGACCCCGTAGAAAAGATCAAAGGATCTTCTTGAGATCCTTTTTTTCTGCGCGTAATCTGCTGCTTGCAAACAAAAAAACC
ACCGCTACCAGCGGTGGTTTGTTTGCCGGATCAAGAGCTACCAACTCTTTTTCCGAAGGTAACTGGCTTCAGCAGAGCGCAGATACCAAATACTGTC
CTTCTAGTGTAGCCGTAGTTAGGCCACCACTTCAAGAACTCTGTAGCACCGCCTACATACCTCGCTCTGCTAATCCTGTTACCAGTGGCTGCTGCCA
GTGGCGATAAGTCGTGTCTTACCGGGTTGGACTCAAGACGATAGTTACCGGATAAGGCGCAGCGGTCGGGCTGAACGGGGGGTTCGTGCACACAGCC
CAGCTTGGAGCGAACGACCTACACCGAACTGAGATACCTACAGCGTGAGCATTGAGAAAGCGCCACGCTTCCCGAAGGGAGAAAGGCGGACAGGTAT
```

CCGGTAAGCGGCAGGGTCGGAACAGGAGAGCGCACGAGGGAGCTTCCAGGGGGAAACGCCTGGTATCTTTATAGTCCTGTCGGGTTTCGCCACCTCT
GACTTGAGCGTCGATTTTTGTGATGCTCGTCAGGGGGGCGGAGCCTATGGAAAAACGCCAGCAACGCGAGCTCTAATACGACTCACTATAG

>Ab1c2-mCherry
ATGGGCGGCGCATGAGAGAAGCCCAGACCAATTACCTACCCAAAATGGAGAAAGTTCACGTTGACATCGAGGAAGACAGCCCATTCCTCAGAGCTTT
GCAGCGGAGCTTCCCGCAGTTTGAGGTAGAAGCCAAGCAGGTCACTGATAATGACCATGCTAATGCCAGAGCGTTTTCGCATCTGGCTTCAAAACTG
ATCGAAACGGAGGTGGACCCATCCGACACGATCCTTGACATTGGAAGTGCGCCCGCCCGCAGAATGTATTCTAAGCACAAGTATCATTGTATCTGTC
CGATGAGATGTGCGGAAGATCCGGACAGATTGTATAAGTATGCAACTAAGCTGAAGAAAAACTGTAAGGAAATAACTGATAAGGAATTGGACAAGAA
AATGAAGGAGCTGGCCGCCGTCATGAGCGACCCTGACCTGGAAACTGAGACTATGTGCCTCCACGACGACGAGTCGTGTCGCTACGAAGGGCAAGTC
GCTGTTTACCAGGATGTATACGCGGTTGACGGACCGACAAGTCTCTATCACCAAGCCAATAAGGGAGTTAGAGTCGCCTACTGGATAGGCTTTGACA
CCACCCCTTTTATGTTTAAGAACTTGGCTGGAGCATATCCATCATACTCTACCAACTGGGCCGACGAAACCGTGTTAACGGCTCGTAACATAGGCCT
ATGCAGCTCTGACGTTATGGAGCGGTCACGTAGAGGGATGTCCATTCTTAGAAAGAAGTATTTGAAACCATCCAACAATGTTCTATTCTCTGTTGGC
TCGACCATCTACCACGAGAAGAGGGACTTACTGAGGAGCTGGCACCTGCCGTCTGTATTTCACTTACGTGGCAAGCAAAATTACACATGTCGGTGTG
AGACTATAGTTAGTTGCGACGGGTACGTCGTTAAAAGAATAGCTATCAGTCCAGGCCTGTATGGGAAGCCTTCAGGCTATGCTGCTACGATGCACCG
CGAGGGATTCTTGTGCTGCAAAGTGACAGACACATTGAACGGGGAGAGGGTCTCTTTTCCCGTGTGCACGTATGTGCCAGCTACATTGTGTGACCAA
ATGACTGGCATACTGGCAACAGATGTCAGTGCGGACGACGCGCAAAAACTGCTGGTTGGGCTCAACCAGCGTATAGTCGTCAACGGTCGCACCCAGA
GAAACACCAATACCATGAAAAATTACCTTTTGCCCGTAGTGGCCCAGGCATTTGCTAGGTGGGCAAAGGAATATAAGGAAGATCAAGAAGATGAAAG
GCCACTAGGACTACGAGATAGACAGTTAGTCATGGGGTGTTGTTGGGCTTTTAGAAGGCACAAGATAACATCTATTTATAAGCGCCCGGATACCCAA
ACCATCATCAAAGTGAACAGCGATTTCCACTCATTCGTGCTGCCCAGGATAGGCAGTAACACATTGGAGATCGGGCTGAGAACAAGAATCAGGAAAA
TGTTAGAGGAGCACAAGGAGCCGTCACCTCTCATTACCGCCGAGGACGTACAAGAAGCTAAGTGCGCAGCCGATGAGGCTAAGGAGGTGCGTGAAGC
CGAGGAGTTGCGCGCAGCTCTACCACCTTTGGCAGCTGATGTTGAGGAGCCCACTCTGGAGGCAGACGTCGACTTGATGTTACAAGAGGCTGGGGCC
GGCTCAGTGGAGACACCTCGTGGCTTGATAAAGGTTACCAGCTACGATGGCGAGGACAAGATCGGCTCTTACGCTGTGCTTTCTCCGCAGGCTGTAC
TCAAGAGTGAAAAATTATCTTGCATCCACCCTCTCGCTGAACAAGTCATAGTGATAACACACTCTGGCCGAAAAGGGCGTTATGCCGTGGAACCATA
CCATGGTAAAGTAGTGGTGCCAGAGGGACATGCAATACCCGTCCAGGACTTTCAAGCTCTGAGTGAAAGTGCCACCATTGTGTACAACGAACGTGAG
TTCGTAAACAGGTACCTGCACCATATTGCCACACATGGAGGAGCGCTGAACACTGATGAAGAATATTACAAAACTGTCAAGCCCAGCGAGCACGACG
GCGAATACCTGTACGACATCGACAGGAAACAGTGCGTCAAGAAAGAACTAGTCACTGGGCTAGGGCTCACAGGCGAGCTGGTGGATCCTCCCTTCCA
TGAATTCGCCTACGAGAGTCTGAGAACACGACCAGCCGCTCCTTACCAAGTACCAACCATAGGGGTGTATGGCGTGCCAGGATCAGGCAAGTCTGGC
ATCATTAAAAGCGCAGTCACCAAAAAAGATCTAGTGGTGAGCGCCAAGAAAGAAAACTGTGCAGAAATTATAAGGGACGTCAAGAAAATGAAAGGGC
TGGACGTCAATGCCAGAACTGTGGACTCAGTGCTCTTGAATGGATGCAAACACCCCGTAGAGACCCTGTATATTGACGAAGCTTTTGCTTGTCATGC
AGGTACTCTCAGAGCGCTCATAGCCATTATAAGACCTAAAAAGGCAGTGCTCTGCGGGGATCCCAAACAGTGCGGTTTTTTTAACATGATGTGCCTG
AAAGTGCATTTTAACCACGAGATTTGCACACAAGTCTTCCACAAAAGCATCTCTCGCCGTTGCACTAAATCTGTGACTTCGGTCGTCTCAACCTTGT
TTTACGACAAAAAAATGAGAACGACGAATCCGAAAGAGACTAAGATTGTGATTGACACTACCGGCAGTACCAAACCTAAGCAGGACGATCTCATTCT
CACTTGTTTCAGAGGGTGGGTGAAGCAGTTGCAAATAGATTACAAAGGCAACGAAATAATGACGGCAGCTGCCTCTCAAGGGCTGACCCGTAAAGGT
GTGTATGCCGTTCGGTACAAGGTGAATGAAAATCCTCTGTACGCACCCACCTCAGAACATGTGAACGTCCTACTGACCCGCACGGAGGACCGCATCG
TGTGGAAAACACTAGCCGGCGACCCATGGATAAAAACACTGACTGCCAAGTACCCTGGGAATTTCACTGCCACGATAGAGGAGTGGCAAGCAGAGCA
TGATGCCATCATGAGGCACATCTTGGAGAGACCGGACCCTACCGACGTCTTCCAGAATAAGGCAAACGTGTGTTGGGCCAAGGCTTTAGTGCCGGTG
CTGAAGACCGCTGGCATAGACATGACCACTGAACAATGGAACACTGTGGATTATTTTGAAACGGACAAAGCTCACTCAGCAGAGATAGTATTGAACC
AACTATGCGTGAGGTTCTTTGGACTCGATCTGGACTCCGGTCTATTTTCTGCACCCACTGTTCCGTTATCCATTAGGAATAATCACTGGGATAACTC
CCCGTCGCCTAACATGTACGGGCTGAATAAAGAAGTGGTCCGTCAGCTCTCTCGCAGGTACCCACAACTGCCTCGGGCAGTTGCCACTGGAAGAGTC
TATGACATGAACACTGGTACACTGCGCAATTATGATCCGCGCATAAACCTAGTACCTGTAAACAGAAGACTGCCTCATGCTTTAGTCCTCCACCATA
ATGAACACCCACAGAGTGACTTTTCTTCATTCGTCAGCAAATTGAAGGGCAGAACTGTCCTGGTGGTCGGGGAAAAGTTGTCCGTCCCAGGCAAAAT
GGTTGACTGGTTGTCAGACCGGCCTGAGGCTACCTTCAGAGCTCGGCTGGATTTAGGCATCCCAGGTGATGTGCCCAAATATGACATAATATTTGTT
AATGTGAGGACCCCATATAAATACCATCACTATCAGCAGTGTGAAGACCATGCCATTAAGCTTAGCATGTTGACCAAGAAAGCTTGTCTGCATCTGA
ATCCCGGCGGAACCTGTGTCAGCATAGGTTATGGTTACGCTGACAGGGCCAGCGAAAGCATCATTGGTGCTATAGCGCGGCAGTTCAAGTTTTCCCG
GGTATGCAAACCGAAATCCTCACTTGAAGAGACGGAAGTTCTGTTTGTATTCATTCGGTACGATCGCAAGGCCCGTACGCACAATTCTTACAAGCTT
TCATCAACCTTGACCAACATTTATACAGGTTCCAGACTCCACGAAGCCGGATGTGCACCCTCATATCATGTGGTGCGAGGGGATATTGCCACGGCCA
CCGAAGGAGTGATTATAAATGCTGCTAACAGCAAAGGACAACCTGGCGGAGGGGTGTGCGGAGCGCTGTATAAGAAATTCCCGGAAAGCTTCGATTT
ACAGCCGATCGAAGTAGGAAAAGCGCGACTGGTCAAAGGTGCAGCTAAACATATCATTCATGCCGTAGGACCAAACTTCAACAAAGTTTCGGAGGTT
GAAGGTGACAAACAGTTGGCAGAGGCTTATGAGTCCATCGCTAAGATTGTCAACGATAACAATTACAAGTCAGTAGCGATTCCACTGTTGTCCACCG
GCATCTTTTCCGGGAACAAAGATCGACTAACCCAATCATTGAACCATTTGCTGACAGCTTTAGACACCACTGATGCAGATGTAGCCATATACTGCAG
GGACAAGAAATGGGAAATGACTCTCAAGGAAGCAGTGGCTAGGAGAGAAGCAGTGGAGGAGATATGCATATCCGACGACTCTTCAGTGACAGAACCT
GATGCAGAGCTGGTGAGGGTGCATCCGAAGAGTTCTTTGGCTGGAAGGAAGGGCTACAGCACAAGCGATGGCAAAACTTTCTCATATTTGGAAGGGA
CCAAGTTTCACCAGGCGGCCAAGGATATAGCAGAAATTAATGCCATGTGGCCCGTTGCAACGGAGGCCAATGAGCAGGTATGCATGTATATCCTCGG
AGAAAGCATGAGCAGTATTAGGTCGAAATGCCCCGTCGAAGATTCGGAAGCCTCCACACCACCTAGCACGCTGCCTTGCTTGTGCATCCATGCCATG
ACTCCAGAAAGAGTACAGCGCCTAAAAGCCTCACGTCCAGAACAAATTACTGTGTGCTCATCCTTTCCATTGCCGAAGTATAGAATCACTGGTATGC
AGAAGATCCAATGCTCCCAGCCTATATTGTTCTCACCGAAAGTGCCTGCGTATATTCATCCAAGGAAGTATCTCGTGGAAACACCACCGGTAGACGA
GACTCCGGAGCCATCGGCAGAGAACCAATCCACAGAGGGGACACCTGAACAACCACCACTTATAACCGAGGATGAGACCAGGACTAGAACGCCTGAG
CCGATCATCATCGAAGAGGAAGAAGAGGATAGCATAAGTTTGCTGTCAGATGGCCCGACCCACCAGGTGCTGCAAGTCGAGGCAGACATTCACGGGC
CGCCCTCTGTATCTAGCTCATCCTGGTCCATTCCTCATGCATCCGACTTTGATGTGGACAGTTTATCCATACTTGACACCCTGGAGGGAGCTAGCGT
GACCAGCGGGGCAACGTCAGCCGAGACTAACTCTTACTTCGCAAAGAGTATGGAGTTTCTGGCGCGACCGGTGCCTGCGCCTCGAACAGTATTCAGG
AACCCTCCACATCCCGCTCCGCGCACAAGAACACCGTCACTTGCACCCAGCAGGGCCTGCTCGAGAACCAGCCTAGTTTCCACCCCGCCAGGCGTGA
ATAGGGTGATCACTAGAGAGGAGCTCGAGGCGCTTACCCCGTCACGCACTCCTAGCAGGTCGGTCTCGAGAACCAGCCTGGTCTCCAACCCGCCAGG
CGTAAATAGGGTGATTACAAGAGAGGAGTTTGAGGCGTTCGTAGCACAACAACAATGACGGTTTGATGCGGGTGCATACATCTTTTCCTCCGACACC
GGTCAAGGGCATTTACAACAAAAATCAGTAAGGCAAACGGTGCTATCCGAAGTGGTGTTGGAGAGGACCGAATTGGAGATTTCGTATGCCCCGCGCC
TCGACCAAGAAAAAGAAGAATTACTACGCAAGAAATTACAGTTAAATCCCACACCTGCTAACAGAAGCAGATACCAGTCCAGGAAGGTGGAGAACAT
GAAAGCCATAACAGCTAGACGTATTCTGCAAGGCCTAGGGCATTATTTGAAGGCAGAAGGAAAAGTGGAGTGCTACCGAACCCTGCATCCTGTTCCT
TTGTATTCATCTAGTGTGAACCGTGCCTTTTCAAGCCCCAAGGTCGCAGTGGAAGCCTGTAACGCCATGTTGAAAGAGAACTTTCCGACTGTGGCTT
CTTACTGTATTATTCCAGAGTACGATGCCTATTTGGACATGGTTGACGGAGCTTCATGCTGCTTAGACACTGCCAGTTTTTGCCCTGCAAAGCTGCG
CAGCTTTCCAAAGAAACACTCCTATTTGGAACCCACAATACGATCGGCAGTGCCTTCAGCGATCCAGAACACGCTCCAGAACGTCCTGGCAGCTGCC
ACAAAAAGAAATTGCAATGTCACGCAAATGAGAGAATTGCCCGTATTGGATTCGGCGGCCTTTAATGTGGAATGCTTCAAGAAATATGCGTGTAATA
ATGAATATTGGGAAACGTTTAAAGAAAACCCCATCAGGCTTACTGAAGAAAACGTGGTAAATTACATTACCAAATTAAAAGGACCAAAAGCTGCTGC
TCTTTTTGCGAAGACACATAATTTGAATATGTTGCAGGACATACCAATGGACAGGTTTGTAATGGACTTAAAGAGAGACGTGAAAGTGACTCCAGGA

ACAAAACATACTGAAGAACGGCCCAAGGTACAGGTGATCCAGGCTGCCGATCCGCTAGCAACAGCGTATCTGTGCGGAATCCACCGAGAGCTGGTTA
GGAGATTAAATGCGGTCCTGCTTCCGAACATTCATACACTGTTTGATATGTCGGCTGAAGACTTTGACGCTATTATAGCCGAGCACTTCCAGCCTGG
GGATTGTGTTCTGGAAACTGACATCGCGTCGTTTGATAAAAGTGAGGACGACGCCATGGCTCTGACCGCGTTAATGATTCTGGAAGACTTAGGTGTG
GACGCAGAGCTGTTGACGCTGATTGAGGCGGCTTTCGGCGAAATTTCATCAATACATTTGCCCACTAAAACTAAATTTAAATTCGGAGCCATGATGA
AATCTGGAATGTTCCTCACACTGTTTGTGAACACAGTCATTAACATTGTAATCGCAAGCAGAGTGTTGAGAGAACGGCTAACCGGATCACCATGTGC
AGCATTCATTGGAGATGACAATATCGTGAAAGGAGTCAAATCGGACAAATTAATGGCAGACAGGTGCGCCACCTGGTTGAATATGGAAGTCAAGATT
ATAGATGCTGTGGTGGGCGAGAAAGCGCCTTATTTCTGTGGAGGGTTTATTTTGTGTGACTCCGTGACCGGCACAGCGTGCCGTGTGGCAGACCCCC
TAAAAAGGCTGTTTAAGCTTGGCAAACCTCTGGCAGCAGACGATGAACATGATGATGACAGGAGAAGGGCATTGCATGAAGAGTCAACACGCTGGAA
CCGAGTGGGTATTCTTTCAGAGCTGTGCAAGGCAGTAGAATCAAGGTATGAAACCGTAGGAACTTCCATCATAGTTATGGCCATGACTACTCTAGCT
AGCAGTGTTAAATCATTCAGCTACCTGAGAGGGGCCCCTATAACTCTCTACGGCTAACCTGAATGGACTACGACATAGTCTAGTCCGCCAAGTCTAG
catatgggcgcgccctcagcatcgattgaattggccaccATGGTGAGCAAGGGCGAGGAGGATAACATGGCCATCATCAAGGAGTTCATGCGCTTCA
AGGTGCACATGGAGGGCTCCGTGAACGGCCACGAGTTCGAGATCGAGGGCGAGGGCGAGGGCCGCCCCTACGAGGGCACCCAGACCGCCAAGCTGAA
GGTGACCAAGGGTGGCCCCCTGCCCTTCGCCTGGGACATCCTGTCCCCTCAGTTCATGTACGGCTCCAAGGCCTACGTGAAGCACCCCGCCGACATC
CCCGACTACTTGAAGCTGTCCTTCCCCGAGGGCTTCAAGTGGGAGCGCGTGATGAACTTCGAGGACGGCGGCGTGGTGACCGTGACCCAGGACTCCT
CCCTGCAGGACGGCGAGTTCATCTACAAGGTGAAGCTGCGCGGCACCAACTTCCCCTCCGACGGCCCCGTAATGCAGAAGAAGACCATGGGCTGGGA
GGCCTCCTCCGAGCGGATGTACCCCGAGGACGGCGCCCTGAAGGGCGAGATCAAGCAGAGGCTGAAGCTGAAGGACGGCGGCCACTACGACGCTGAG
GTCAAGACCACCTACAAGGCCAAGAAGCCCGTGCAGCTGCCCGGCGCCTACAACGTCAACATCAAGTTGGACATCACCTCCCACAACGAGGACTACA
CCATCGTGGAACAGTACGAACGCGCCGAGGGCCGCCACTCCACCGGCGGCATGGACGAGCTGTACAAGTAGgaattGGCAAGCTGCTTACATAGAAC
TCGCGGCGATTGGCATGCCGCCTTAAAATTTTTATTTTATTTTTCTTTTCTTTTCCGAATCGGATTTTGTTTTTAATATTTCAAAAAAAAAAAAAAA
AAAAAAAAAAACGCGTCGAGGGGAATTAATTCTTGAAGACGAAAGGGCCAGGTGGCACTTTTCGGGGAAATGTGCGCGGAACCCCTATTTGTTTATT
TTTCTAAATACATTCAAATATGTATCCGCTCATGAGACAATAACCCTGATAAATGCTTCAATAATATTGAAAAAGGAAGAGTATGAGTATTCAACAT
TTCCGTGTCGCCCTTATTCCCTTTTTTGCGGCATTTTGCCTTCCTGTTTTTGCTCACCCAGAAACGCTGGTGAAAGTAAAAGATGCTGAAGATCAGT
TGGGTGCACGAGTGGGTTACATCGAACTGGATCTCAACAGCGGTAAGATCCTTGAGAGTTTTCGCCCCGAAGAACGTTTTCCAATGATGAGCACTTT
TAAAGTTCTGCTATGTGGCGCGGTATTATCCCGTGTTGACGCCGGGCAAGAGCAACTCGGTCGCCGCATACACTATTCTCAGAATGACTTGGTTGAG
TACTCACCAGTCACAGAAAAGCATCTTACGGATGGCATGACAGTAAGAGAATTATGCAGTGCTGCCATAACCATGAGTGATAACACTGCGGCCAACT
TACTTCTGACAACGATCGGAGGACCGAAGGAGCTAACCGCTTTTTTGCACAACATGGGGGATCATGTAACTCGCCTTGATCGTTGGGAACCGGAGCT
GAATGAAGCCATACCAAACGACGAGCGTGACACCACGATGCCTGTAGCAATGGCAACAACGTTGCGCAAACTATTAACTGGCGAACTACTTACTCTA
GCTTCCCGGCAACAATTAATAGACTGGATGGAGGCGGATAAAGTTGCAGGACCACTTCTGCGCTCGGCCCTTCCGGCTGGCTGGTTTATTGCTGATA
AATCTGGAGCCGGTGAGCGTGGGTCTCGCGGTATCATTGCAGCACTGGGGCCAGATGGTAAGCCCTCCCGTATCGTAGTTATCTACACGACGGGGAG
TCAGGCAACTATGGATGAACGAAATAGACAGATCGCTGAGATAGGTGCCTCACTGATTAAGCATTGGTAACTGTCAGACCAAGTTTACTCATATATA
CTTTAGATTGATTTAAAACTTCATTTTTAATTTAAAAGGATCTAGGTGAAGATCCTTTTTGATAATCTCATGACCAAAATCCCTTAACGTGAGTTTT
CGTTCCACTGAGCGTCAGACCCCGTAGAAAAGATCAAAGGATCTTCTTGAGATCCTTTTTTTCTGCGCGTAATCTGCTGCTTGCAAACAAAAAAACC
ACCGCTACCAGCGGTGGTTTGTTTGCCGGATCAAGAGCTACCAACTCTTTTTCCGAAGGTAACTGGCTTCAGCAGAGCGCAGATACCAAATACTGTC
CTTCTAGTGTAGCCGTAGTTAGGCCACCACTTCAAGAACTCTGTAGCACCGCCTACATACCTCGCTCTGCTAATCCTGTTACCAGTGGCTGCTGCCA
GTGGCGATAAGTCGTGTCTTACCGGGTTGGACTCAAGACGATAGTTACCGGATAAGGCGCAGCGGTCGGGCTGAACGGGGGGTTCGTGCACACAGCC
CAGCTTGGAGCGAACGACCTACACCGAACTGAGATACCTACAGCGTGAGCATTGAGAAAGCGCCACGCTTCCCGAAGGGAGAAAGGCGGACAGGTAT
CCGGTAAGCGGCAGGGTCGGAACAGGAGAGCGCACGAGGGAGCTTCCAGGGGGAAACGCCTGGTATCTTTATAGTCCTGTCGGGTTTCGCCACCTCT
GACTTGAGCGTCGATTTTTGTGATGCTCGTCAGGGGGGCGGAGCCTATGGAAAAACGCCAGCAACGCGAGCTCTAATACGACTCACTATAG

>ab1c2-mCherry
ATGGGCGGCGCATGAGAGAAGCCCAGACCAATTACCTACCCAAAATGGAGAAAGTTCACGTTGACATCGAGGAAGACAGCCCATTCCTCAGAGCTTT
GCAGCGGAGCTTCCCGCAGTTTGAGGTAGAAGCCAAGCAGGTCACTGATAATGACCATGCTAATGCCAGAGCGTTTTCGCATCTGGCTTCAAAACTG
ATCGAAACGGAGGTGGACCCATCCGACACGATCCTTGACATTGGAAGTGCGCCCGCCCGCAGAATGTATTCTAAGCACAAGTATCATTGTATCTGTC
CGATGAGATGTGCGGAAGATCCGGACAGATTGTATAAGTATGCAACTAAGCTGAAGAAAAACTGTAAGGAAATAACTGATAAGGAATTGGACAAGAA
AATGAAGGAGCTGGCCGCCGTCATGAGCGACCCTGACCTGGAAACTGAGACTATGTGCCTCCACGACGACGAGTCGTGTCGCTACGAAGGGCAAGTC
GCTGTTTACCAGGATGTATACGCGGTTGACGGACCGACAAGTCTCTATCACCAAGCCAATAAGGGAGTTAGAGTCGCCTACTGGATAGGCTTTGACA
CCACCCCTTTTATGTTTAAGAACTTGGCTGGAGCATATCCATCATACTCTACCAACTGGGCCGACGAAACCGTGTTAACGGCTCGTAACATAGGCCT
ATGCAGCTCTGACGTTATGGAGCGGTCACGTAGAGGGATGTCCATTCTTAGAAAGAAGTATTTGAAACCATCCAACAATGTTCTATTCTCTGTTGGC
TCGACCATCTACCACGAGAAGAGGGACTTACTGAGGAGCTGGCACCTGCCGTCTGTATTTCACTTACGTGGCAAGCAAAATTACACATGTCGGTGTG
AGACTATAGTTAGTTGCGACGGGTACGTCGTTAAAAGAATAGCTATCAGTCCAGGCCTGTATGGGAAGCCTTCAGGCTATGCTGCTACGATGCACCG
CGAGGGATTCTTGTGCTGCAAAGTGACAGACACATTGAACGGGGAGAGGGTCTCTTTTCCCGTGTGCACGTATGTGCCAGCTACATTGTGTGACCAA
ATGACTGGCATACTGGCAACAGATGTCAGTGCGGACGACGCGCAAAAACTGCTGGTTGGGCTCAACCAGCGTATAGTCGTCAACGGTCGCACCCAGA
GAAACACCAATACCATGAAAAATTACCTTTTGCCCGTAGTGGCCCAGGCATTTGCTAGGTGGGCAAAGGAATATAAGGAAGATCAAGAAGATGAAAG
GCCACTAGGACTACGAGATAGACAGTTAGTCATGGGGTGTTGTTGGGCTTTTAGAAGGCACAAGATAACATCTATTTATAAGCGCCCGGATACCCAA
ACCATCATCAAAGTGAACAGCGATTTCCACTCATTCGTGCTGCCCAGGATAGGCAGTAACACATTGGAGATCGGGCTGAGAACAAGAATCAGGAAAA
TGTTAGAGGAGCACAAGGAGCCGTCACCTCTCATTACCGCCGAGGACGTACAAGAAGCTAAGTGCGCAGCCGATGAGGCTAAGGAGGTGCGTGAAGC
CGAGGAGTTGCGCGCAGCTCTACCACCTTTGGCAGCTGATGTTGAGGAGCCCACTCTGGAGGCAGACGTCGACTTGATGTTACAAGAGGCTGGGGCC
GGCTCAGTGGAGACACCTCGTGGCTTGATAAAGGTTACCAGCTACGATGGCGAGGACAAGATCGGCTCTTACGCTGTGCTTTCTCCGCAGGCTGTAC
TCAAGAGTGAAAAATTATCTTGCATCCACCCTCTCGCTGAACAAGTCATAGTGATAACACACTCTGGCCGAAAAGGGCGTTATGCCGTGGAACCATA
CCATGGTAAAGTAGTGGTGCCAGAGGGACATGCAATACCCGTCCAGGACTTTCAAGCTCTGAGTGAAAGTGCCACCATTGTGTACAACGAACGTGAG
TTCGTAAACAGGTACCTGCACCATATTGCCACACATGGGGGAGCGCTGAACACTGATGAAGAATATTACAAAACTGTCAAGCCCAGCGAGCACGACG
GCGAATACCTGTACGACATCGACAGGAAACAGTGCGTCAAGAAAGAACTAGTCACTGGGCTAGGGCTCACAGGCGAGCTGGTGGATCCTCCCTTCCA
TGAATTCGCCTACGAGAGTCTGAGAACACGACCAGCCGCTCCTTACCAAGTACCAACCATAGGGGTGTATGGCGTGCCAGGATCAGGCAAGTCTGGC
ATCATTAAAAGCGCAGTCACCAAAAAAGATCTAGTGGTGAGCGCCAAGAAAGAAAACTGTGCAGAAATTATAAGGGACGTCAAGAAAATGAAAGGGC
TGGACGTCAATGCCAGAACTGTGGACTCAGTGCTCTTGAATGGATGCAAACACCCCGTAGAGACCCTGTATATTGACGAAGCTTTTGCTTGTCATGC
AGGTACTCTCAGAGCGCTCATAGCCATTATAAGACCTAAAAAGGCAGTGCTCTGCGGGGATCCCAAACAGTGCGGTTTTTTTAACATGATGTGCCTG
AAAGTGCATTTTAACCACGAGATTTGCACACAAGTCTTCCACAAAAGCATCTCTCGCCGTTGCACTAAATCTGTGACTTCGGTCGTCTCAACCTTGT
TTTACGACAAAAAAATGAGAACGACGAATCCGAAAGAGACTAAGATTGTGATTGACACTACCGGCAGTACCAAACCTAAGCAGGACGATCTCATTCT
CACTTGTTTCAGAGGGTGGGTGAAGCAGTTGCAAATAGATTACAAAGGCAACGAAATAATGACGGCAGCTGCCTCTCAAGGGCTGACCCGTAAAGGT
GTGTATGCCGTTCGGTACAAGGTGAATGAAAATCCTCTGTACGCACCCACCTCAGAACATGTGAACGTCCTACTGACCCGCACGGAGGACCGCATCG
TGTGGAAAACACTAGCCGGCGACCCATGGATAAAAACACTGACTGCCAAGTACCCTGGGAATTTCACTGCCACGATAGAGGAGTGGCAAGCAGAGCA
TGATGCCATCATGAGGCACATCTTGGAGAGACCGGACCCTACCGACGTCTTCCAGAATAAGGCAAACGTGTGTTGGGCCAAGGCTTTAGTGCCGGTG

CTGAAGACCGCTGGCATAGACATGACCACTGAACAATGGAACACTGTGGATTATTTTGAAACGGACAAAGCTCACTCAGCAGAGATAGTATTGAACC
AACTATGCGTGAGGTTCTTTGGACTCGATCTGGACTCCGGTCTATTTTCTGCACCCACTGTTCCGTTATCCATTAGGAATAATCACTGGGATAACTC
CCCGTCGCCTAACATGTACGGGCTGAATAAAGAAGTGGTCCGTCAGCTCTCTCGCAGGTACCCACAACTGCCTCGGGCAGTTGCCACTGGAAGAGTC
TATGACATGAACACTGGTACACTGCGCAATTATGATCCGCGCATAAACCTAGTACCTGTAAACAGAAGACTGCCTCATGCTTTAGTCCTCCACCATA
ATGAACACCCACAGAGTGACTTTTCTTCATTCGTCAGCAAATTGAAGGGCAGAACTGTCCTGGTGGTCGGGGAAAAGTTGTCCGTCCCAGGCAAAAT
GGTTGACTGGTTGTCAGACCGGCCTGAGGCTACCTTCAGAGCTCGGCTGGATTTAGGCATCCCAGGTGATGTGCCCAAATATGACATAATATTTGTT
AATGTGAGGACCCCATATAAATACCATCACTATCAGCAGTGTGAAGACCATGCCATTAAGCTTAGCATGTTGACCAAGAAAGCTTGTCTGCATCTGA
ATCCCGGCGGAACCTGTGTCAGCATAGGTTATGGTTACGCTGACAGGGCCAGCGAAAGCATCATTGGTGCTATAGCGCGGCAGTTCAAGTTTTCCCG
GGTATGCAAACCGAAATCCTCACTTGAAGAGACGGAAGTTCTGTTTGTATTCATTCGGTACGATCGCAAGGCCCGTACGCACAATTCTTACAAGCTT
TCATCAACCTTGACCAACATTTATACAGGTTCCAGACTCCACGAAGCCGGATGTGCACCCTCATATCATGTGGTGCGAGGGGATATTGCCACGGCCA
CCGAAGGAGTGATTATAAATGCTGCTAACAGCAAAGGACAACCTGGCGGAGGGGTGTGCGGAGCGCTGTATAAGAAATTCCCGGAAAGCTTCGATTT
ACAGCCGATCGAAGTAGGAAAAGCGCGACTGGTCAAAGGTGCAGCTAAACATATCATTCATGCCGTAGGACCAAACTTCAACAAAGTTTCGGAGGTT
GAAGGTGACAAACAGTTGGCAGAGGCTTATGAGTCCATCGCTAAGATTGTCAACGATAACAATTACAAGTCAGTAGCGATTCCACTGTTGTCCACCG
GCATCTTTTCCGGGAACAAAGATCGACTAACCCAATCATTGAACCATTTGCTGACAGCTTTAGACACCACTGATGCAGATGTAGCCATATACTGCAG
GGACAAGAAATGGGAAATGACTCTCAAGGAAGCAGTGGCTAGGAGAGAAGCAGTGGAGGAGATATGCATATCCGACGACTCTTCAGTGACAGAACCT
GATGCAGAGCTGGTGAGGGTGCATCCGAAGAGTTCTTTGGCTGGAAGGAAGGGCTACAGCACAAGCGATGGCAAAACTTTCTCATATTTGGAAGGGA
CCAAGTTTCACCAGGCGGCCAAGGATATAGCAGAAATTAATGCCATGTGGCCCGTTGCAACGGAGGCCAATGAGCAGGTATGCATGTATATCCTCGG
AGAAAGCATGAGCAGTATTAGGTCGAAATGCCCCGTCGAAGATTCGGAAGCCTCCACACCACCTAGCACGCTGCCTTGCTTGTGCATCCATGCCATG
ACTCCAGAAAGAGTACAGCGCCTAAAAGCCTCACGTCCAGAACAAATTACTGTGTGCTCATCCTTTCCATTGCCGAAGTATAGAATCACTGGTATGC
AGAAGATCCAATGCTCCCAGCCTATATTGTTCTCACCGAAAGTGCCTGCGTATATTCATCCAAGGAAGTATCTCGTGGAAACACCACCGGTAGACGA
GACTCCGGAGCCATCGGCAGAGAACCAATCCACAGAGGGGACACCTGAACAACCACCACTTATAACCGAGGATGAGACCAGGACTAGAACGCCTGAG
CCGATCATCATCGAAGAGGAAGAAGAGGATAGCATAAGTTTGCTGTCAGATGGCCCGACCCACCAGGTGCTGCAAGTCGAGGCAGACATTCACGGGC
CGCCCTCTGTATCTAGCTCATCCTGGTCCATTCCTCATGCATCCGACTTTGATGTGGACAGTTTATCCATACTTGACACCCTGGAGGGAGCTAGCGT
GACCAGCGGGGCAACGTCAGCCGAGACTAACTCTTACTTCGCAAAGAGTATGGAGTTTCTGGCGCGACCGGTGCCTGCGCCTCGAACAGTATTCAGG
AACCCTCCACATCCCGCTCCGCGCACAAGAACACCGTCACTTGCACCCAGCAGGGCCTGCTCGAGAACCAGCCTAGTTTCCACCCCGCCAGGCGTGA
ATAGGGTGATCACTAGAGAGGAGCTCGAGGCGCTTACCCCGTCACGCACTCCTAGCAGGTCGGTCTCGAGAACCAGCCTGGTCTCCAACCCGCCAGG
CGTAAATAGGGTGATTACAAGAGAGGAGTTTGAGGCGTTCGTAGCACAACAACAATGACGGTTTGATGCGGGTGCATACATCTTTTCCTCCGACACC
GGTCAAGGGCATTTACAACAAAAATCAGTAAGGCAAACGGTGCTATCCGAAGTGGTGTTGGAGAGGACCGAATTGGAGATTTCGTATGCCCCGCGCC
TCGACCAAGAAAAAGAAGAATTACTACGCAAGAAATTACAGTTAAATCCCACACCTGCTAACAGAAGCAGATACCAGTCCAGGAAGGTGGAGAACAT
GAAAGCCATAACAGCTAGACGTATTCTGCAAGGCCTAGGGCATTATTTGAAGGCAGAAGGAAAAGTGGAGTGCTACCGAACCCTGCATCCTGTTCCT
TTGTATTCATCTAGTGTGAACCGTGCCTTTTCAAGCCCCAAGGTCGCAGTGGAAGCCTGTAACGCCATGTTGAAAGAGAACTTTCCGACTGTGGCTT
CTTACTGTATTATTCCAGAGTACGATGCCTATTTGGACATGGTTGACGGAGCTTCATGCTGCTTAGACACTGCCAGTTTTTGCCCTGCAAAGCTGCG
CAGCTTTCCAAAGAAACACTCCTATTTGGAACCCACAATACGATCGGCAGTGCCTTCAGCGATCCAGAACACGCTCCAGAACGTCCTGGCAGCTGCC
ACAAAAAGAAATTGCAATGTCACGCAAATGAGAGAATTGCCCGTATTGGATTCGGCGGCCTTTAATGTGGAATGCTTCAAGAAATATGCGTGTAATA
ATGAATATTGGGAAACGTTTAAAGAAAACCCCATCAGGCTTACTGAAGAAAACGTGGTAAATTACATTACCAAATTAAAAGGACCAAAAGCTGCTGC
TCTTTTTGCGAAGACACATAATTTGAATATGTTGCAGGACATACCAATGGACAGGTTTGTAATGGACTTAAAGAGAGACGTGAAAGTGACTCCAGGA
ACAAAACATACTGAAGAACGGCCCAAGGTACAGGTGATCCAGGCTGCCGATCCGCTAGCAACAGCGTATCTGTGCGGAATCCACCGAGAGCTGGTTA
GGAGATTAAATGCGGTCCTGCTTCCGAACATTCATACACTGTTTGATATGTCGGCTGAAGACTTTGACGCTATTATAGCCGAGCACTTCCAGCCTGG
GGATTGTGTTCTGGAAACTGACATCGCGTCGTTTGATAAAAGTGAGGACGACGCCATGGCTCTGACCGCGTTAATGATTCTGGAAGACTTAGGTGTG
GACGCAGAGCTGTTGACGCTGATTGAGGCGGCTTTCGGCGAAATTTCATCAATACATTTGCCCACTAAAACTAAATTTAAATTCGGAGCCATGATGA
AATCTGGAATGTTCCTCACACTGTTTGTGAACACAGTCATTAACATTGTAATCGCAAGCAGAGTGTTGAGAGAACGGCTAACCGGATCACCATGTGC
AGCATTCATTGGAGATGACAATATCGTGAAAGGAGTCAAATCGGACAAATTAATGGCAGACAGGTGCGCCACCTGGTTGAATATGGAAGTCAAGATT
ATAGATGCTGTGGTGGGCGAGAAAGCGCCTTATTTCTGTGGAGGGTTTATTTTGTGTGACTCCGTGACCGGCACAGCGTGCCGTGTGGCAGACCCCC
TAAAAAGGCTGTTTAAGCTTGGCAAACCTCTGGCAGCAGACGATGAACATGATGATGACAGGAGAAGGGCATTGCATGAAGAGTCAACACGCTGGAA
CCGAGTGGGTATTCTTTCAGAGCTGTGCAAGGCAGTAGAATCAAGGTATGAAACCGTAGGAACTTCCATCATAGTTATGGCCATGACTACTCTAGCT
AGCAGTGTTAAATCATTCAGCTACCTGAGAGGGGCCCCTATAACTCTCTACGGCTAACCTGAATGGACTACGACATAGTCTAGTCCGCCAAGTCTAG
catatgggcgcgccctcagcatcgattgaattggccaccATGGTGAGCAAGGGCGAGGAGGATAACATGGCCATCATCAAGGAGTTCATGCGCTTCA
AGGTGCACATGGAGGGCTCCGTGAACGGCCACGAGTTCGAGATCGAGGGCGAGGGCGAGGGCCGCCCCTACGAGGGCACCCAGACCGCCAAGCTGAA
GGTGACCAAGGGTGGCCCCCTGCCCTTCGCCTGGGACATCCTGTCCCCTCAGTTCATGTACGGCTCCAAGGCCTACGTGAAGCACCCCGCCGACATC
CCCGACTACTTGAAGCTGTCCTTCCCCGAGGGCTTCAAGTGGGAGCGCGTGATGAACTTCGAGGACGGCGGCGTGGTGACCGTGACCCAGGACTCCT
CCCTGCAGGACGGCGAGTTCATCTACAAGGTGAAGCTGCGCGGCACCAACTTCCCCTCCGACGGCCCCGTAATGCAGAAGAAGACCATGGGCTGGGA
GGCCTCCTCCGAGCGGATGTACCCCGAGGACGGCGCCCTGAAGGGCGAGATCAAGCAGAGGCTGAAGCTGAAGGACGGCGGCCACTACGACGCTGAG
GTCAAGACCACCTACAAGGCCAAGAAGCCCGTGCAGCTGCCCGGCGCCTACAACGTCAACATCAAGTTGGACATCACCTCCCACAACGAGGACTACA
CCATCGTGGAACAGTACGAACGCGCCGAGGGCCGCCACTCCACCGGCGGCATGGACGAGCTGTACAAGTAGgaattGGCAAGCTGCTTACATAGAAC
TCGCGGCGATTGGCATGCCGCCTTAAAATTTTTATTTTATTTTTCTTTTCTTTTCCGAATCGGATTTTGTTTTTAATATTTCAAAAAAAAAAAAAAA
AAAAAAAAAAACGCGTCGAGGGGAATTAATTCTTGAAGACGAAAGGGCCAGGTGGCACTTTTCGGGGAAATGTGCGCGGAACCCCTATTTGTTTATT
TTTCTAAATACATTCAAATATGTATCCGCTCATGAGACAATAACCCTGATAAATGCTTCAATAATATTGAAAAAGGAAGAGTATGAGTATTCAACAT
TTCCGTGTCGCCCTTATTCCCTTTTTTGCGGCATTTTGCCTTCCTGTTTTTGCTCACCCAGAAACGCTGGTGAAAGTAAAAGATGCTGAAGATCAGT
TGGGTGCACGAGTGGGTTACATCGAACTGGATCTCAACAGCGGTAAGATCCTTGAGAGTTTTCGCCCCGAAGAACGTTTTCCAATGATGAGCACTTT
TAAAGTTCTGCTATGTGGCGCGGTATTATCCCGTGTTGACGCCGGGCAAGAGCAACTCGGTCGCCGCATACACTATTCTCAGAATGACTTGGTTGAG
TACTCACCAGTCACAGAAAAGCATCTTACGGATGGCATGACAGTAAGAGAATTATGCAGTGCTGCCATAACCATGAGTGATAACACTGCGGCCAACT
TACTTCTGACAACGATCGGAGGACCGAAGGAGCTAACCGCTTTTTTGCACAACATGGGGGATCATGTAACTCGCCTTGATCGTTGGGAACCGGAGCT
GAATGAAGCCATACCAAACGACGAGCGTGACACCACGATGCCTGTAGCAATGGCAACAACGTTGCGCAAACTATTAACTGGCGAACTACTTACTCTA
GCTTCCCGGCAACAATTAATAGACTGGATGGAGGCGGATAAAGTTGCAGGACCACTTCTGCGCTCGGCCCTTCCGGCTGGCTGGTTTATTGCTGATA
AATCTGGAGCCGGTGAGCGTGGGTCTCGCGGTATCATTGCAGCACTGGGGCCAGATGGTAAGCCCTCCCGTATCGTAGTTATCTACACGACGGGGAG
TCAGGCAACTATGGATGAACGAAATAGACAGATCGCTGAGATAGGTGCCTCACTGATTAAGCATTGGTAACTGTCAGACCAAGTTTACTCATATATA
CTTTAGATTGATTTAAAACTTCATTTTTAATTTAAAAGGATCTAGGTGAAGATCCTTTTTGATAATCTCATGACCAAAATCCCTTAACGTGAGTTTT
CGTTCCACTGAGCGTCAGACCCCGTAGAAAAGATCAAAGGATCTTCTTGAGATCCTTTTTTTCTGCGCGTAATCTGCTGCTTGCAAACAAAAAAACC
ACCGCTACCAGCGGTGGTTTGTTTGCCGGATCAAGAGCTACCAACTCTTTTTCCGAAGGTAACTGGCTTCAGCAGAGCGCAGATACCAAATACTGTC
CTTCTAGTGTAGCCGTAGTTAGGCCACCACTTCAAGAACTCTGTAGCACCGCCTACATACCTCGCTCTGCTAATCCTGTTACCAGTGGCTGCTGCCA
GTGGCGATAAGTCGTGTCTTACCGGGTTGGACTCAAGACGATAGTTACCGGATAAGGCGCAGCGGTCGGGCTGAACGGGGGGTTCGTGCACACAGCC
CAGCTTGGAGCGAACGACCTACACCGAACTGAGATACCTACAGCGTGAGCATTGAGAAAGCGCCACGCTTCCCGAAGGGAGAAAGGCGGACAGGTAT

CCGGTAAGCGGCAGGGTCGGAACAGGAGAGCGCACGAGGGAGCTTCCAGGGGGAAACGCCTGGTATCTTTATAGTCCTGTCGGGTTTCGCCACCTCT
GACTTGAGCGTCGATTTTTGTGATGCTCGTCAGGGGGGCGGAGCCTATGGAAAAACGCCAGCAACGCGAGCTCTAATACGACTCACTATAG

>Ab2c2-mCherry
ATGGGCGGCGCATGAGAGAAGCCCAGACCAATTACCTACCCAAAATGGAGAAAGTTCACGTTGACATCGAGGAAGACAGCCCATTCCTCAGAGCTTT
GCAGCGGAGCTTCCCGCAGTTTGAGGTAGAAGCCAAGCAGGTCACTGATAATGACCATGCTAATGCCAGAGCGTTTTCGCATCTGGCTTCAAAACTG
ATCGAAACGGAGGTGGACCCATCCGACACGATCCTTGACATTGGAAGTGCGCCCGCCCGCAGAATGTATTCTAAGCACAAGTATCATTGTATCTGTC
CGATGAGATGTGCGGAAGATCCGGACAGATTGTATAAGTATGCAACTAAGCTGAAGAAAAACTGTAAGGAAATAACTGATAAGGAATTGGACAAGAA
AATGAAGGAGCTGGCCGCCGTCATGAGCGACCCTGACCTGGAAACTGAGACTATGTGCCTCCACGACGACGAGTCGTGTCGCTACGAAGGGCAAGTC
GCTGTTTACCAGGATGTATACGCGGTTGACGGACCGACAAGTCTCTATCACCAAGCCAATAAGGGAGTTAGAGTCGCCTACTGGATAGGCTTTGACA
CCACCCCTTTTATGTTTAAGAACTTGGCTGGAGCATATCCATCATACTCTACCAACTGGGCCGACGAAACCGTGTTAACGGCTCGTAACATAGGCCT
ATGCAGCTCTGACGTTATGGAGCGGTCACGTAGAGGGATGTCCATTCTTAGAAAGAAGTATTTGAAACCATCCAACAATGTTCTATTCTCTGTTGGC
TCGACCATCTACCACGAGAAGAGGGACTTACTGAGGAGCTGGCACCTGCCGTCTGTATTTCACTTACGTGGCAAGCAAAATTACACATGTCGGTGTG
AGACTATAGTTAGTTGCGACGGGTACGTCGTTAAAAGAATAGCTATCAGTCCAGGCCTGTATGGGAAGCCTTCAGGCTATGCTGCTACGATGCACCG
CGAGGGATTCTTGTGCTGCAAAGTGACAGACACATTGAACGGGGAGAGGGTCTCTTTTCCCGTGTGCACGTATGTGCCAGCTACATTGTGTGACCAA
ATGACTGGCATACTGGCAACAGATGTCAGTGCGGACGACGCGCAAAAACTGCTGGTTGGGCTCAACCAGCGTATAGTCGTCAACGGTCGCACCCAGA
GAAACACCAATACCATGAAAAATTACCTTTTGCCCGTAGTGGCCCAGGCATTTGCTAGGTGGGCAAAGGAATATAAGGAAGATCAAGAAGATGAAAG
GCCACTAGGACTACGAGATAGACAGTTAGTCATGGGGTGTTGTTGGGCTTTTAGAAGGCACAAGATAACATCTATTTATAAGCGCCCGGATACCCAA
ACCATCATCAAAGTGAACAGCGATTTCCACTCATTCGTGCTGCCCAGGATAGGCAGTAACACATTGGAGATCGGGCTGAGAACAAGAATCAGGAAAA
TGTTAGAGGAGCACAAGGAGCCGTCACCTCTCATTACCGCCGAGGACGTACAAGAAGCTAAGTGCGCAGCCGATGAGGCTAAGGAGGTGCGTGAAGC
CGAGGAGTTGCGCGCAGCTCTACCACCTTTGGCAGCTGATGTTGAGGAGCCCACTCTGGAGGCAGACGTCGACTTGATGTTACAAGAGGCTGGGGCC
GGCTCAGTGGAGACACCTCGTGGCTTGATAAAGGTTACCAGCTACGATGGCGAGGACAAGATCGGCTCTTACGCTGTGCTTTCTCCGCAGGCTGTAC
TCAAGAGTGAAAAATTATCTTGCATCCACCCTCTCGCTGAACAAGTCATAGTGATAACACACTCTGGCCGAAAAGGGCGTTATGCCGTGGAACCATA
CCATGGTAAAGTAGTGGTGCCAGAGGGACATGCAATACCCGTCCAGGACTTTCAAGCTCTGAGTGAAAGTGCCACCATTGTGTACAACGAACGTGAG
TTCGTAAACAGGTACCTGCACCATATTGCCACACATGGAGGAGCGCTGAACACTGATGAAGAATATTACAAAACTGTCAAGCCCAGCGAGCACGACG
GCGAATACCTGTACGACATCGACAGGAAACAGTGCGTCAAGAAAGAACTAGTCACTGGGCTAGGGCTCACAGGCGAGCTGGTGGATCCTCCCTTCCA
TGAATTCGCCTACGAGAGTCTGAGAACACGACCAGCCGCTCCTTACCAAGTACCAACCATAGGGGTGTATGGCGTGCCAGGATCAGGCAAGTCTGGC
ATCATTAAAAGCGCAGTCACCAAAAAAGATCTAGTGGTGAGCGCCAAGAAAGAAAACTGTGCAGAAATTATAAGGGACGTCAAGAAAATGAAAGGGC
TGGACGTCAATGCCAGAACTGTGGACTCAGTGCTCTTGAATGGATGCAAACACCCCGTAGAGACCCTGTATATTGACGAAGCTTTTGCTTGTCATGC
AGGTACTCTCAGAGCGCTCATAGCCATTATAAGACCTAAAAAGGCAGTGCTCTGCGGGGATCCCAAACAGTGCGGTTTTTTAACATGATGTGCCTG
AAAGTGCATTTTAACCACGAGATTTGCACACAAGTCTTCCACAAAAGCATCTCTCGCCGTTGCACTAAATCTGTGACTTCGGTCGTCTCAACCTTGT
TTTACGACAAAAAAATGAGAACGACGAATCCGAAAGAGACTAAGATTGTGATTGACACTACCGGCAGTACCAAACCTAAGCAGGACGATCTCATTCT
CACTTGTTTCAGAGGGTGGGTGAAGCAGTTGCAAATAGATTACAAAGGCAACGAAATAATGACGGCAGCTGCCTCTCAAGGGCTGACCCGTAAAGGT
GTGTATGCCGTTCGGTACAAGGTGAATGAAAATCCTCTGTACGCACCCACCTCAGAACATGTGAACGTCCTACTGACCCGCACGGAGGACCGCATCG
TGTGGAAAACACTAGCCGGCGACCCATGGATAAAAACACTGACTGCCAAGTACCCTGGGAATTTCACTGCCACGATAGAGGAGTGGCAAGCAGAGCA
TGATGCCATCATGAGGCACATCTTGGAGAGACCGGACCCTACCGACGTCTTCCAGAATAAGGCAAACGTGTGTTGGGCCAAGGCTTTAGTGCCGGTG
CTGAAGACCGCTGGCATAGACATGACCACTGAACAATGGAACACTGTGGATTATTTTGAAACGGACAAAGCTCACTCAGCAGAGATAGTATTGAACC
AACTATGCGTGAGGTTCTTTGGACTCGATCTGGACTCCGGTCTATTTTCTGCACCCACTGTTCCGTTATCCATTAGGAATAATCACTGGGATAACTC
CCCGTCGCCTAACATGTACGGGCTGAATAAAGAAGTGGTCCGTCAGCTCTCTCGCAGGTACCCACAACTGCCTCGGGCAGTTGCCACTGGAAGAGTC
TATGACATGAACACTGGTACACTGCGCAATTATGATCCGCGCATAAACCTAGTACCTGTAAACAGAAGACTGCCTCATGCTTTAGTCCTCCACCATA
ATGAACACCCACAGAGTGACTTTTCTTCATTCGTCAGCAAATTGAAGGGCAGAACTGTCCTGGTGGTCGGGGAAAAGTTGTCCGTCCCAGGCAAAAT
GGTTGACTGGTTGTCAGACCGGCCTGAGGCTACCTTCAGAGCTCGGCTGGATTTAGGCATCCCAGGTGATGTGCCCAAATATGACATAATATTTGTT
AATGTGAGGACCCCATATAAATACCATCACTATCAGCAGTGTGAAGACCATGCCATTAAGCTTAGCATGTTGACCAAGAAAGCTTGTCTGCATCTGA
ATCCCGGCGGAACCTGTGTCAGCATAGGTTATGGTTACGCTGACAGGGCCAGCGAAAGCATCATTGGTGCTATAGCGCGGCAGTTCAAGTTTTCCCG
GGTATGCAAACCGAAATCCTCACTTGAAGAGACGGAAGTTCTGTTTGTATTCATTGGGTACGATCGCAAGGCCCGTACGCACAATTCTTACAAGCTT
TCATCAACCTTGACCAACATTTATACAGGTTCCAGACTCCACGAAGCCGGATGTGCACCCTCATATCATGTGGTGCGAGGGGATATTGCCACGGCCA
CCGAAGGAGTGATTATAAATGCTGCTAACAGCAAAGGACAACCTGGCGGAGGGGTGTGCGGAGCGCTGTATAAGAAATTCCCGGAAAGCTTCGATTT
ACAGCCGATCGAAGTAGGAAAAGCGCGACTGGTCAAAGGTGCAGCTAAACATATCATTCATGCCGTAGGACCAAACTTCAACAAAGTTTCGGAGGTT
GAAGGTGACAAACAGTTGGCAGAGGCTTATGAGTCCATCGCTGAGATTGTCAACGATAACAATTACAAGTCAGTAGCGATTCCACTGTTGTCCACCG
GCATCTTTTCCGGGAACAAAGATCGACTAACCCAATCATTGAACCATTTGCTGACAGCTTTAGACACCACTGATGCAGATGTAGCCATATACTGCAG
GGACAAGAAATGGGAAATGACTCTCAAGGAAGCAGTGGCTAGGAGAGAAGCAGTGGAGGAGATATGCATATCCGACGACTCTTCAGTGACAGAACCT
GATGCAGAGCTGGTGAGGGTGCATCCGAAGAGTTCTTTGGCTGGAAGGAAGGGCTACAGCACAAGCGATGGCAAAACTTTCTCATATTTGGAAGGGA
CCAAGTTTCACCAGGCGGCCAAGGATATAGCAGAAATTAATGCCATGTGGCCCGTTGCAACGGAGGCCAATGAGCAGGTATGCATGTATATCCTCGG
AGAAAGCATGAGCAGTATTAGGTCGAAATGCCCCGTCGAAGATTCGGAAGCCTCCACACCACCTAGCACGCTGCCTTGCTTGTGCATCCATGCCATG
ACTCCAGAAAGAGTACAGCGCCTAAAAGCCTCACGTCCAGAACAAATTACTGTGTGCTCATCCTTTCCATTGCCGAAGTATAGAATCACTGGTATGC
AGAAGATCCAATGCTCCCAGCCTATATTGTTCTCACCGAAAGTGCCTGCGTATATTCATCCAAGGAAGTATCTCGTGGAAACACCACCGGTAGACGA
GACTCCGGAGCCATCGGCAGAGAACCAATCCACAGAGGGGACACCTGAACAACCACCACTTATAACCGAGGATGAGACCAGGACTAGAACGCCTGAG
CCGATCATCATCGAAGAGGAAGAAGAGGATAGCATAAGTTTGCTGTCAGATGGCCCGACCCACCAGGTGCTGCAAGTCGAGGCAGACATTCACGGGC
CGCCCTCTGTATCTAGCTCATCCTGGTCCATTCCTCATGCATCCGACTTTGATGTGGACAGTTTATCCATACTTGACACCCTGGAGGGAGCTAGCGT
GACCAGCGGGGCAACGTCAGCCGAGACTAACTCTTACTTCGCAAAGAGTATGGAGTTTCTGGCGCGACCGGTGCCTGCGCCTCGAACAGTATTCAGG
AACCCTCCACATCCCGCTCCGCGCACAAGAACACCGTCACTTGCACCCAGCAGGGCCTGCTCGAGAACCAGCCTAGTTTCCACCCCGCCAGGCGTGA
ATAGGGTGATCACTAGAGAGGAGCTCGAGGCGCTTACCCCGTCACGCACTCCTAGCAGGTCGGTCTCGAGAACCAGCCTGGTCTCCAACCCGCCAGG
CGTAAATAGGGTGATTACAAGAGAGGAGTTTGAGGCGTTCGTAGCACAACAACAATGACGGTTTGATGCGGGTGCATACATCTTTTCCTCCGACACC
GGTCAAGGGCATTTACAACAAAAATCAGTAAGGCAAACGGTGCTATCCGAAGTGGTGTTGGAGAGGACCGAATTGGAGATTTCGTATGCCCCGCGCC
TCGACCAAGAAAAAGAAGAATTACTACGCAAGAAATTACAGTTAAATCCCACACCTGCTAACAGAAGCAGATACCAGTCCAGGAAGGTGGAGAACAT
GAAAGCCATAACAGCTAGACGTATTCTGCAAGGCCTAGGGCATTATTTGAAGGCAGAAGGAAAAGTGGAGTGCTACCGAACCCTGCATCCTGTTCCT
TTGTATTCATCTAGTGTGAACCGTGCCTTTTCAAGCCCCAAGGTCGCAGTGGAAGCCTGTAACGCCATGTTGAAAGAGAACTTTCCGACTGTGGCTT
CTTACTGTATTATTCCAGAGTACGATGCCTATTTGGACATGGTTGACGGAGCTTCATGCTGCTTAGACACTGCCAGTTTTTGCCCTGCAAAGCTGCG
CAGCTTTCCAAAGAAACACTCCTATTTGGAACCCACAATACGATCGGCAGTGCCTTCAGCGATCCAGAACACGCTCCAGAACGTCCTGGCAGCTGCC
ACAAAAAGAAATTGCAATGTCACGCAAATGAGAGAATTGCCCGTATTGGATTCGGCGGCCTTTAATGTGGAATGCTTCAAGAAATATGCGTGTAATA
ATGAATATTGGGAAACGTTTAAAGAAAACCCCATCAGGCTTACTGAAGAAAACGTGGTAAATTACATTACCAAATTAAAAGGACCAAAAGCTGCTGC
TCTTTTTGCGAAGACACATAATTTGAATATGTTGCAGGACATACCAATGGACAGGTTTGTAATGGACTTAAAGAGAGACGTGAAAGTGACTCCAGGA

ACAAAACATACTGAAGAACGGCCCAAGGTACAGGTGATCCAGGCTGCCGATCCGCTAGCAACAGCGTATCTGTGCGGAATCCACCGAGAGCTGGTTA
GGAGATTAAATGCGGTCCTGCTTCCGAACATTCATACACTGTTTGATATGTCGGCTGAAGACTTTGACGCTATTATAGCCGAGCACTTCCAGCCTGG
GGATTGTGTTCTGGAAACTGACATCGCGTCGTTTGATAAAAGTGAGGACGACGCCATGGCTCTGACCGCGTTAATGATTCTGGAAGACTTAGGTGTG
GACGCAGAGCTGTTGACGCTGATTGAGGCGGCTTTCGGCGAAATTTCATCAATACATTTGCCCACTAAAACTAAATTTAAATTCGGAGCCATGATGA
AATCTGGAATGTTCCTCACACTGTTTGTGAACACAGTCATTAACATTGTAATCGCAAGCAGAGTGTTGAGAGAACGGCTAACCGGATCACCATGTGC
AGCATTCATTGGAGATGACAATATCGTGAAAGGAGTCAAATCGGACAAATTAATGGCAGACAGGTGCGCCACCTGGTTGAATATGGAAGTCAAGATT
ATAGATGCTGTGGTGGGCGAGAAAGCGCCTTATTTCTGTGGAGGGTTTATTTTGTGTGACTCCGTGACCGGCACAGCGTGCCGTGTGGCAGACCCCC
TAAAAAGGCTGTTTAAGCTTGGCAAACCTCTGGCAGCAGACGATGAACATGATGATGACAGGAGAAGGGCATTGCATGAAGAGTCAACACGCTGGAA
CCGAGTGGGTATTCTTTCAGAGCTGTGCAAGGCAGTAGAATCAAGGTATGAAACCGTAGGAACTTCCATCATAGTTATGGCCATGACTACTCTAGCT
AGCAGTGTTAAATCATTCAGCTACCTGAGAGGGGCCCCTATAACTCTCTACGGCTAACCTGAATGGACTACGACATAGTCTAGTCCGCCAAGTCTAG
catatgggcgcgccctcagcatcgattgaattggccaccATGGTGAGCAAGGGCGAGGAGGATAACATGGCCATCATCAAGGAGTTCATGCGCTTCA
AGGTGCACATGGAGGGCTCCGTGAACGGCCACGAGTTCGAGATCGAGGGCGAGGGCGAGGGCCGCCCCTACGAGGGCACCCAGACCGCCAAGCTGAA
GGTGACCAAGGGTGGCCCCCTGCCCTTCGCCTGGGACATCCTGTCCCCTCAGTTCATGTACGGCTCCAAGGCCTACGTGAAGCACCCCGCCGACATC
CCCGACTACTTGAAGCTGTCCTTCCCCGAGGGCTTCAAGTGGGAGCGCGTGATGAACTTCGAGGACGGCGGCGTGGTGACCGTGACCCAGGACTCCT
CCCTGCAGGACGGCGAGTTCATCTACAAGGTGAAGCTGCGCGGCACCAACTTCCCCTCCGACGGCCCCGTAATGCAGAAGAAGACCATGGGCTGGGA
GGCCTCCTCCGAGCGGATGTACCCCGAGGACGGCGCCCTGAAGGGCGAGATCAAGCAGAGGCTGAAGCTGAAGGACGGCGGCCACTACGACGCTGAG
GTCAAGACCACCTACAAGGCCAAGAAGCCCGTGCAGCTGCCCGGCGCCTACAACGTCAACATCAAGTTGGACATCACCTCCCACAACGAGGACTACA
CCATCGTGGAACAGTACGAACGCGCCGAGGGCCGCCACTCCACCGGCGGCATGGACGAGCTGTACAAGTAGgaattGGCAAGCTGCTTACATAGAAC
TCGCGGCGATTGGCATGCCGCCTTAAAATTTTTATTTTATTTTTCTTTTCTTTTCCGAATCGGATTTTGTTTTTAATATTTCAAAAAAAAAAAAAA
AAAAAAAAAAACGCGTCGAGGGGAATTAATTCTTGAAGACGAAAGGGCCAGGTGGCACTTTTCGGGGAAATGTGCGCGGAACCCCTATTTGTTTATT
TTTCTAAATACATTCAAATATGTATCCGCTCATGAGACAATAACCCTGATAAATGCTTCAATAATATTGAAAAAGGAAGAGTATGAGTATTCAACAT
TTCCGTGTCGCCCTTATTCCCTTTTTTGCGGCATTTTGCCTTCCTGTTTTTGCTCACCCAGAAACGCTGGTGAAAGTAAAAGATGCTGAAGATCAGT
TGGGTGCACGAGTGGGTTACATCGAACTGGATCTCAACAGCGGTAAGATCCTTGAGAGTTTTCGCCCCGAAGAACGTTTTCCAATGATGAGCACTTT
TAAAGTTCTGCTATGTGGCGCGGTATTATCCCGTGTTGACGCCGGGCAAGAGCAACTCGGTCGCCGCATACACTATTCTCAGAATGACTTGGTTGAG
TACTCACCAGTCACAGAAAAGCATCTTACGGATGGCATGACAGTAAGAGAATTATGCAGTGCTGCCATAACCATGAGTGATAACACTGCGGCCAACT
TACTTCTGACAACGATCGGAGGACCGAAGGAGCTAACCGCTTTTTTGCACAACATGGGGGATCATGTAACTCGCCTTGATCGTTGGGAACCGGAGCT
GAATGAAGCCATACCAAACGACGAGCGTGACACCACGATGCCTGTAGCAATGGCAACAACGTTGCGCAAACTATTAACTGGCGAACTACTTACTCTA
GCTTCCCGGCAACAATTAATAGACTGGATGGAGGCGGATAAAGTTGCAGGACCACTTCTGCGCTCGGCCCTTCCGGCTGGCTGGTTTATTGCTGATA
AATCTGGAGCCGGTGAGCGTGGGTCTCGCGGTATCATTGCAGCACTGGGGCCAGATGGTAAGCCCTCCCGTATCGTAGTTATCTACACGACGGGGAG
TCAGGCAACTATGGATGAACGAAATAGACAGATCGCTGAGATAGGTGCCTCACTGATTAAGCATTGGTAACTGTCAGACCAAGTTTACTCATATATA
CTTTAGATTGATTTAAAACTTCATTTTTAATTTAAAAGGATCTAGGTGAAGATCCTTTTTGATAATCTCATGACCAAAATCCCTTAACGTGAGTTTT
CGTTCCACTGAGCGTCAGACCCCGTAGAAAAGATCAAAGGATCTTCTTGAGATCCTTTTTTTCTGCGCGTAATCTGCTGCTTGCAAACAAAAAAACC
ACCGCTACCAGCGGTGGTTTGTTTGCCGGATCAAGAGCTACCAACTCTTTTTCCGAAGGTAACTGGCTTCAGCAGAGCGCAGATACCAAATACTGTC
CTTCTAGTGTAGCCGTAGTTAGGCCACCACTTCAAGAACTCTGTAGCACCGCCTACATACCTCGCTCTGCTAATCCTGTTACCAGTGGCTGCTGCCA
GTGGCGATAAGTCGTGTCTTACCGGGTTGGACTCAAGACGATAGTTACCGGATAAGGCGCAGCGGTCGGGCTGAACGGGGGGTTCGTGCACACAGCC
CAGCTTGGAGCGAACGACCTACACCGAACTGAGATACCTACAGCGTGAGCATTGAGAAAGCGCCACGCTTCCCGAAGGGAGAAAGGCGGACAGGTAT
CCGGTAAGCGGCAGGGTCGGAACAGGAGAGCGCACGAGGGAGCTTCCAGGGGGAAACGCCTGGTATCTTTATAGTCCTGTCGGGTTTCGCCACCTCT
GACTTGAGCGTCGATTTTTGTGATGCTCGTCAGGGGGGCGGAGCCTATGGAAAAACGCCAGCAACGCGAGCTCTAATACGACTCACTATAG

>ab2c2-mCherry
ATGGGCGGCGCATGAGAGAAGCCCAGACCAATTACCTACCCAAAATGGAGAAAGTTCACGTTGACATCGAGGAAGACAGCCCATTCCTCAGAGCTTT
GCAGCGGAGCTTCCCGCAGTTTGAGGTAGAAGCCAAGCAGGTCACTGATAATGACCATGCTAATGCCAGAGCGTTTTCGCATCTGGCTTCAAAACTG
ATCGAAACGGAGGTGGACCCATCCGACACGATCCTTGACATTGGAAGTGCGCCCGCCCGCAGAATGTATTCTAAGCACAAGTATCATTGTATCTGTC
CGATGAGATGTGCGGAAGATCCGGACAGATTGTATAAGTATGCAACTAAGCTGAAGAAAAACTGTAAGGAAATAACTGATAAGGAATTGGACAAGAA
AATGAAGGAGCTGGCCGCCGTCATGAGCGACCCTGACCTGGAAACTGAGACTATGTGCCTCCACGACGACGAGTCGTGTCGCTACGAAGGGCAAGTC
GCTGTTTACCAGGATGTATACGCGGTTGACGGACCGACAAGTCTCTATCACCAAGCCAATAAGGGAGTTAGAGTCGCCTACTGGATAGGCTTTGACA
CCACCCCTTTTATGTTTAAGAACTTGGCTGGAGCATATCCATCATACTCTACCAACTGGGCCGACGAAACCGTGTTAACGGCTCGTAACATAGGCCT
ATGCAGCTCTGACGTTATGGAGCGGTCACGTAGAGGGATGTCCATTCTTAGAAAGAAGTATTTGAAACCATCCAACAATGTTCTATTCTCTGTTGGC
TCGACCATCTACCACGAGAAGAGGGACTTACTGAGGAGCTGGCACCTGCCGTCTGTATTTCACTTACGTGGCAAGCAAAATTACACATGTCGGTGTG
AGACTATAGTTAGTTGCGACGGGTACGTCGTTAAAAGAATAGCTATCAGTCCAGGCCTGTATGGGAAGCCTTCAGGCTATGCTGCTACGATGCACCG
CGAGGGATTCTTGTGCTGCAAAGTGACAGACACATTGAACGGGGAGAGGGTCTCTTTTCCCGTGTGCACGTATGTGCCAGCTACATTGTGTGACCAA
ATGACTGGCATACTGGCAACAGATGTCAGTGCGGACGACGCGCAAAAACTGCTGGTTGGGCTCAACCAGCGTATAGTCGTCAACGGTCGCACCCAGA
GAAACACCAATACCATGAAAAATTACCTTTTGCCCGTAGTGGCCCAGGCATTTGCTAGGTGGGCAAAGGAATATAAGGAAGATCAAGAAGATGAAAG
GCCACTAGGACTACGAGATAGACAGTTAGTCATGGGGTGTTGTTGGGCTTTTAGAAGGCACAAGATAACATCTATTTATAAGCGCCCGGATACCCAA
ACCATCATCAAAGTGAACAGCGATTTCCACTCATTCGTGCTGCCCAGGATAGGCAGTAACACATTGGAGATCGGGCTGAGAACAAGAATCAGGAAAA
TGTTAGAGGAGCACAAGGAGCCGTCACCTCTCATTACCGCCGAGGACGTACAAGAAGCTAAGTGCGCAGCCGATGAGGCTAAGGAGGTGCGTGAAGC
CGAGGAGTTGCGCGCAGCTCTACCACCTTTGGCAGCTGATGTTGAGGAGCCCACTCTGGAGGCAGACGTCGACTTGATGTTACAAGAGGCTGGGGCC
GGCTCAGTGGAGACACCTCGTGGCTTGATAAAGGTTACCAGCTACGATGGCGAGGACAAGATCGGCTCTTACGCTGTGCTTTCTCCGCAGGCTGTAC
TCAAGAGTGAAAAATTATCTTGCATCCACCCTCTCGCTGAACAAGTCATAGTGATAACACACTCTGGCCGAAAAGGGCGTTATGCCGTGGAACCATA
CCATGGTAAAGTAGTGGTGCCAGAGGGACATGCAATACCCGTCCAGGACTTTCAAGCTCTGAGTGAAAGTGCCACCATTGTGTACAACGAACGTGAG
TTCGTAAACAGGTACCTGCACCATATTGCCACACATGGGGGAGCGCTGAACACTGATGAAGAATATTACAAAACTGTCAAGCCCAGCGAGCACGACG
GCGAATACCTGTACGACATCGACAGGAAACAGTGCGTCAAGAAAGAACTAGTCACTGGGCTAGGGCTCACAGGCGAGCTGGTGGATCCTCCCTTCCA
TGAATTCGCCTACGAGAGTCTGAGAACACGACCAGCCGCTCCTTACCAAGTACCAACCATAGGGGTGTATGGCGTGCCAGGATCAGGCAAGTCTGGC
ATCATTAAAAGCGCAGTCACCAAAAAAGATCTAGTGGTGAGCGCCAAGAAAGAAAACTGTGCAGAAATTATAAGGGACGTCAAGAAAATGAAAGGGC
TGGACGTCAATGCCAGAACTGTGGACTCAGTGCTCTTGAATGGATGCAAACACCCCGTAGAGACCCTGTATATTGACGAAGCTTTTGCTTGTCATGC
AGGTACTCTCAGAGCGCTCATAGCCATTATAAGACCTAAAAAGGCAGTGCTCTGCGGGGATCCCAAACAGTGCGGTTTTTTAACATGATGTGCCTG
AAAGTGCATTTTAACCACGAGATTTGCACACAAGTCTTCCACAAAAGCATCTCTCGCCGTTGCACTAAATCTGTGACTTCGGTCGTCTCAACCTTGT
TTTACGACAAAAAAATGAGAACGACGAATCCGAAAGAGACTAAGATTGTGATTGACACTACCGGCAGTACCAAACCTAAGCAGGACGATCTCATTCT
CACTTGTTTCAGAGGGTGGGTGAAGCAGTTGCAAATAGATTACAAAGGCAACGAAATAATGACGGCAGCTGCCTCTCAAGGGCTGACCCGTAAAGGT
GTGTATGCCGTTCGGTACAAGGTGAATGAAAATCCTCTGTACGCACCCACCTCAGAACATGTGAACGTCCTACTGACCCGCACGGAGGACCGCATCG
TGTGGAAAACACTAGCCGGCGACCCATGGATAAAAACACTGACTGCCAAGTACCCTGGGAATTTCACTGCCACGATAGAGGAGTGGCAAGCAGAGCA
TGATGCCATCATGAGGCACATCTTGGAGAGACCGGACCCTACCGACGTCTTCCAGAATAAGGCAAACGTGTGTTGGGCCAAGGCTTTAGTGCCGGTG

CTGAAGACCGCTGGCATAGACATGACCACTGAACAATGGAACACTGTGGATTATTTTGAAACGGACAAAGCTCACTCAGCAGAGATAGTATTGAACC
AACTATGCGTGAGGTTCTTTGGACTCGATCTGGACTCCGGTCTATTTTCTGCACCCACTGTTCCGTTATCCATTAGGAATAATCACTGGGATAACTC
CCCGTCGCCTAACATGTACGGGCTGAATAAAGAAGTGGTCCGTCAGCTCTCTCGCAGGTACCCACAACTGCCTCGGGCAGTTGCCACTGGAAGAGTC
TATGACATGAACACTGGTACACTGCGCAATTATGATCCGCGCATAAACCTAGTACCTGTAAACAGAAGACTGCCTCATGCTTTAGTCCTCCACCATA
ATGAACACCCACAGAGTGACTTTTCTTCATTCGTCAGCAAATTGAAGGGCAGAACTGTCCTGGTGGTCGGGGAAAAGTTGTCCGTCCCAGGCAAAAT
GGTTGACTGGTTGTCAGACCGGCCTGAGGCTACCTTCAGAGCTCGGCTGGATTTAGGCATCCCAGGTGATGTGCCCAAATATGACATAATATTTGTT
AATGTGAGGACCCCATATAAATACCATCACTATCAGCAGTGTGAAGACCATGCCATTAAGCTTAGCATGTTGACCAAGAAAGCTTGTCTGCATCTGA
ATCCCGGCGGAACCTGTGTCAGCATAGGTTATGGTTACGCTGACAGGGCCAGCGAAAGCATCATTGGTGCTATAGCGCGGCAGTTCAAGTTTTCCCG
GGTATGCAAACCGAAATCCTCACTTGAAGAGACGGAAGTTCTGTTTGTATTCATTGGGTACGATCGCAAGGCCCGTACGCACAATTCTTACAAGCTT
TCATCAACCTTGACCAACATTTATACAGGTTCCAGACTCCACGAAGCCGGATGTGCACCCTCATATCATGTGGTGCGAGGGGATATTGCCACGGCCA
CCGAAGGAGTGATTATAAATGCTGCTAACAGCAAAGGACAACCTGGCGGAGGGGTGTGCGGAGCGCTGTATAAGAAATTCCCGGAAAGCTTCGATTT
ACAGCCGATCGAAGTAGGAAAAGCGCGACTGGTCAAAGGTGCAGCTAAACATATCATTCATGCCGTAGGACCAAACTTCAACAAAGTTTCGGAGGT
GAAGGTGACAAACAGTTGGCAGAGGCTTATGAGTCCATCGCTGAGATTGTCAACGATAACAATTACAAGTCAGTAGCGATTCCACTGTTGTCCACCG
GCATCTTTTCCGGGAACAAAGATCGACTAACCCAATCATTGAACCATTTGCTGACAGCTTTAGACACCACTGATGCAGATGTAGCCATATACTGCAG
GGACAAGAAATGGGAAATGACTCTCAAGGAAGCAGTGGCTAGGAGAGAAGCAGTGGAGGAGATATGCATATCCGACGACTCTTCAGTGACAGAACCT
GATGCAGAGCTGGTGAGGGTGCATCCGAAGAGTTCTTTGGCTGGAAGGAAGGGCTACAGCACAAGCGATGGCAAAACTTTCTCATATTTGGAAGGGA
CCAAGTTTCACCAGGCGGCCAAGGATATAGCAGAAATTAATGCCATGTGGCCCGTTGCAACGGAGGCCAATGAGCAGGTATGCATGTATATCCTCGG
AGAAAGCATGAGCAGTATTAGGTCGAAATGCCCCGTCGAAGATTCGGAAGCCTCCACACCACCTAGCACGCTGCCTTGCTTGTGCATCCATGCCATG
ACTCCAGAAAGAGTACAGCGCCTAAAAGCCTCACGTCCAGAACAAATTACTGTGTGCTCATCCTTTCCATTGCCGAAGTATAGAATCACTGGTATGC
AGAAGATCCAATGCTCCCAGCCTATATTGTTCTCACCGAAAGTGCCTGCGTATATTCATCCAAGGAAGTATCTCGTGGAAACACCACCGGTAGACGA
GACTCCGGAGCCATCGGCAGAGAACCAATCCACAGAGGGGACACCTGAACAACCACCACTTATAACCGAGGATGAGACCAGGACTAGAACGCCTGAG
CCGATCATCATCGAAGAGGAAGAAGAGGATAGCATAAGTTTGCTGTCAGATGGCCCGACCCACCAGGTGCTGCAAGTCGAGGCAGACATTCACGGGC
CGCCCTCTGTATCTAGCTCATCCTGGTCCATTCCTCATGCATCCGACTTTGATGTGGACAGTTTATCCATACTTGACACCCTGGAGGGAGCTAGCGT
GACCAGCGGGGCAACGTCAGCCGAGACTAACTCTTACTTCGCAAAGAGTATGGAGTTTCTGGCGCGACCGGTGCCTGCGCCTCGAACAGTATTCAGG
AACCCTCCACATCCCGCTCCGCGCACAAGAACACCGTCACTTGCACCCAGCAGGGCCTGCTCGAGAACCAGCCTAGTTTCCACCCCGCCAGGCGTGA
ATAGGGTGATCACTAGAGAGGAGCTCGAGGCGCTTACCCCGTCACGCACTCCTAGCAGGTCGGTCTCGAGAACCAGCCTGGTCTCCAACCCGCCAGG
CGTAAATAGGGTGATTACAAGAGAGGAGTTTGAGGCGTTCGTAGCACAACAACAATGACGGTTTGATGCGGGTGCATACATCTTTTCCTCCGACACC
GGTCAAGGGCATTTACAACAAAAATCAGTAAGGCAAACGGTGCTATCCGAAGTGGTGTTGGAGAGGACCGAATTGGAGATTTCGTATGCCCCGCGCC
TCGACCAAGAAAAAGAAGAATTACTACGCAAGAAATTACAGTTAAATCCCACACCTGCTAACAGAAGCAGATACCAGTCCAGGAAGGTGGAGAACAT
GAAAGCCATAACAGCTAGACGTATTCTGCAAGGCCTAGGGCATTATTTGAAGGCAGAAGGAAAAGTGGAGTGCTACCGAACCCTGCATCCTGTTCCT
TTGTATTCATCTAGTGTGAACCGTGCCTTTTCAAGCCCCAAGGTCGCAGTGGAAGCCTGTAACGCCATGTTGAAAGAGAACTTTCCGACTGTGGCTT
CTTACTGTATTATTCCAGAGTACGATGCCTATTTGGACATGGTTGACGGAGCTTCATGCTGCTTAGACACTGCCAGTTTTTGCCCTGCAAAGCTGCG
CAGCTTTCCAAAGAAACACTCCTATTTGGAACCCACAATACGATCGGCAGTGCCTTCAGCGATCCAGAACACGCTCCAGAACGTCCTGGCAGCTGCC
ACAAAAAGAAATTGCAATGTCACGCAAATGAGAGAATTGCCCGTATTGGATTCGGCGGCCTTTAATGTGGAATGCTTCAAGAAATATGCGTGTAATA
ATGAATATTGGGAAACGTTTAAAGAAAACCCCATCAGGCTTACTGAAGAAAACGTGGTAAATTACATTACCAAATTAAAAGGACCAAAAGCTGCTGC
TCTTTTTGCGAAGACACATAATTTGAATATGTTGCAGGACATACCAATGGACAGGTTTGTAATGGACTTAAAGAGAGACGTGAAAGTGACTCCAGGA
ACAAAACATACTGAAGAACGGCCCAAGGTACAGGTGATCCAGGCTGCCGATCCGCTAGCAACAGCGTATCTGTGCGGAATCCACCGAGAGCTGGTTA
GGAGATTAAATGCGGTCCTGCTTCCGAACATTCATACACTGTTTGATATGTCGGCTGAAGACTTTGACGCTATTATAGCCGAGCACTTCCAGCCTGG
GGATTGTGTTCTGGAAACTGACATCGCGTCGTTTGATAAAAGTGAGGACGACGCCATGGCTCTGACCGCGTTAATGATTCTGGAAGACTTAGGTGTG
GACGCAGAGCTGTTGACGCTGATTGAGGCGGCTTTCGGCGAAATTTCATCAATACATTTGCCCACTAAAACTAAATTTAAATTCGGAGCCATGATGA
AATCTGGAATGTTCCTCACACTGTTTGTGAACACAGTCATTAACATTGTAATCGCAAGCAGAGTGTTGAGAGAACGGCTAACCGGATCACCATGTGC
AGCATTCATTGGAGATGACAATATCGTGAAAGGAGTCAAATCGGACAAATTAATGGCAGACAGGTGCGCCACCTGGTTGAATATGGAAGTCAAGATT
ATAGATGCTGTGGTGGGCGAGAAAGCGCCTTATTTCTGTGGAGGGTTTATTTTGTGTGACTCCGTGACCGGCACAGCGTGCCGTGTGGCAGACCCCC
TAAAAAGGCTGTTTAAGCTTGGCAAACCTCTGGCAGCAGACGATGAACATGATGATGACAGGAGAAGGGCATTGCATGAAGAGTCAACACGCTGGAA
CCGAGTGGGTATTCTTTCAGAGCTGTGCAAGGCAGTAGAATCAAGGTATGAAACCGTAGGAACTTCCATCATAGTTATGGCCATGACTACTCTAGCT
AGCAGTGTTAAATCATTCAGCTACCTGAGAGGGGCCCCTATAACTCTCTACGGCTAACCTGAATGGACTACGACATAGTCTAGTCCGCCAAGTCTAG
catatgggcgcgccctcagcatcgattgaattggccaccATGGTGAGCAAGGGCGAGGAGGATAACATGGCCATCATCAAGGAGTTCATGCGCTTCA
AGGTGCACATGGAGGGCTCCGTGAACGGCCACGAGTTCGAGATCGAGGGCGAGGGCGAGGGCCGCCCCTACGAGGGCACCCAGACCGCCAAGCTGAA
GGTGACCAAGGGTGGCCCCCTGCCCTTCGCCTGGGACATCCTGTCCCCTCAGTTCATGTACGGCTCCAAGGCCTACGTGAAGCACCCCGCCGACATC
CCCGACTACTTGAAGCTGTCCTTCCCCGAGGGCTTCAAGTGGGAGCGCGTGATGAACTTCGAGGACGGCGGCGTGGTGACCGTGACCCAGGACTCCT
CCCTGCAGGACGGCGAGTTCATCTACAAGGTGAAGCTGCGCGGCACCAACTTCCCCTCCGACGGCCCCGTAATGCAGAAGAAGACCATGGGCTGGGA
GGCCTCCTCCGAGCGGATGTACCCCGAGGACGGCGCCCTGAAGGGCGAGATCAAGCAGAGGCTGAAGCTGAAGGACGGCGGCCACTACGACGCTGAG
GTCAAGACCACCTACAAGGCCAAGAAGCCCGTGCAGCTGCCCGGCGCCTACAACGTCAACATCAAGTTGGACATCACCTCCCACAACGAGGACTACA
CCATCGTGGAACAGTACGAACGCGCCGAGGGCCGCCACTCCACCGGCGGCATGGACGAGCTGTACAAGTAGgaattGGCAAGCTGCTTACATAGAAC
TCGCGGCGATTGGCATGCCGCCTTAAAATTTTTATTTTATTTTTCTTTTCTTTTCCGAATCGGATTTTGTTTTTAATATTTCAAAAAAAAAAAAAAA
AAAAAAAAAAAACGCGTCGAGGGGAATTAATTCTTGAAGACGAAAGGGCCAGGTGGCACTTTTCGGGGAAATGTGCGCGGAACCCCTATTTGTTTATT
TTTCTAAATACATTCAAATATGTATCCGCTCATGAGACAATAACCCTGATAAATGCTTCAATAATATTGAAAAAGGAAGAGTATGAGTATTCAACAT
TTCCGTGTCGCCCTTATTCCCTTTTTTGCGGCATTTTGCCTTCCTGTTTTTGCTCACCCAGAAACGCTGGTGAAAGTAAAAGATGCTGAAGATCAGT
TGGGTGCACGAGTGGGTTACATCGAACTGGATCTCAACAGCGGTAAGATCCTTGAGAGTTTTCGCCCCGAAGAACGTTTTCCAATGATGAGCACTTT
TAAAGTTCTGCTATGTGGCGCGGTATTATCCCGTGTTGACGCCGGGCAAGAGCAACTCGGTCGCCGCATACACTATTCTCAGAATGACTTGGTTGAG
TACTCACCAGTCACAGAAAAGCATCTTACGGATGGCATGACAGTAAGAGAATTATGCAGTGCTGCCATAACCATGAGTGATAACACTGCGGCCAACT
TACTTCTGACAACGATCGGAGGACCGAAGGAGCTAACCGCTTTTTTGCACAACATGGGGGATCATGTAACTCGCCTTGATCGTTGGGAACCGGAGCT
GAATGAAGCCATACCAAACGACGAGCGTGACACCACGATGCCTGTAGCAATGGCAACAACGTTGCGCAAACTATTAACTGGCGAACTACTTACTCTA
GCTTCCCGGCAACAATTAATAGACTGGATGGAGGCGGATAAAGTTGCAGGACCACTTCTGCGCTCGGCCCTTCCGGCTGGCTGGTTTATTGCTGATA
AATCTGGAGCCGGTGAGCGTGGGTCTCGCGGTATCATTGCAGCACTGGGGCCAGATGGTAAGCCCTCCCGTATCGTAGTTATCTACACGACGGGGAG
TCAGGCAACTATGGATGAACGAAATAGACAGATCGCTGAGATAGGTGCCTCACTGATTAAGCATTGGTAACTGTCAGACCAAGTTTACTCATATATA
CTTTAGATTGATTTAAAACTTCATTTTTAATTTAAAAGGATCTAGGTGAAGATCCTTTTTGATAATCTCATGACCAAAATCCCTTAACGTGAGTTTT
CGTTCCACTGAGCGTCAGACCCCGTAGAAAAGATCAAAGGATCTTCTTGAGATCCTTTTTTTCTGCGCGTAATCTGCTGCTTGCAAACAAAAAAACC
ACCGCTACCAGCGGTGGTTTGTTTGCCGGATCAAGAGCTACCAACTCTTTTTCCGAAGGTAACTGGCTTCAGCAGAGCGCAGATACCAAATACTGTC
CTTCTAGTGTAGCCGTAGTTAGGCCACCACTTCAAGAACTCTGTAGCACCGCCTACATACCTCGCTCTGCTAATCCTGTTACCAGTGGCTGCTGCCA
GTGGCGATAAGTCGTGTCTTACCGGGTTGGACTCAAGACGATAGTTACCGGATAAGGCGCAGCGGTCGGGCTGAACGGGGGGTTCGTGCACACAGCC
CAGCTTGGAGCGAACGACCTACACCGAACTGAGATACCTACAGCGTGAGCATTGAGAAAGCGCCACGCTTCCCGAAGGGAGAAAGGCGGACAGGTAT

CCGGTAAGCGGCAGGGTCGGAACAGGAGAGCGCACGAGGGAGCTTCCAGGGGGAAACGCCTGGTATCTTTATAGTCCTGTCGGGTTTCGCCACCTCT
GACTTGAGCGTCGATTTTTGTGATGCTCGTCAGGGGGGCGGAGCCTATGGAAAAACGCCAGCAACGCGAGCTCTAATACGACTCACTATAG

>pTW064MM
TCGCGCGTTTCGGTGATGACGGTGAAAACCTCTGACACATGCAGCTCCCGGAGACGGTCACAGCTTGTCTGTAAGCGGATGCCGGGAGCAGACAAGC
CCGTCAGGGCGCGTCAGCGGGTGTTGGCGGGTGTCGGGGCTGGCTTAACTATGCGGCATCAGAGCAGATTGTACTGAGAGTGCACCATATGCGGTGT
GAAATACCGCACAGATGCGTAAGGAGAAAATACCGCATCAGGCGCCATTCGCCATTCAGGCTGCGCAACTGTTGGGAAGGGCGATCGGTGCGGGCCT
CTTCGCTATTACGCCAGCTGGCGAAAGGGGGATGTGCTGCAAGGCGATTAAGTTGGGTAACGCCAGGGTTTTCCCAGTCACGACGTTGTAAAACGAC
GGCCAGTGAATTCGAGCTCGGTACCCGGGGATCCTCTAGAGTCGACCTGCAGGCATGCAAGCTTGGCGTAATCATGGTCATAGCTGTTTCCTGTGTG
AAATTGTTATCCGCTCACAATTCCACACAACATACGAGCCGGAAGCATAAAGTGTAAAGCCTGGGGTGCCTAATGAGTGAGCTAACTCACATTAATT
GCGTTGCGCTCACTGCCCGCTTTCCAGTCGGGAAACCTGTCGTGCCAGCTGCATTAATGAATCGGCCAACGCGCGGGGAGAGGCGGTTTGCGTATTG
GGCGCTCTTCCGCTGGTCTCACACCGTCGACCGACAGCCTTCCAAATGTTCTTCGGGTGATGCTGCCAACTTAGTCGACCGACAGCCTTCCAAATGT
TCTTCTCAAACGGAATCGTCGTATCCAGCCTACTCGCTATTGTCCTCAATGCCGTATTAAATCATAAAAAGAAATAAGAAAAAGAGGTGCGAGCCTC
TTTTTTGTGTGACAAAATAAAAACATCTACCTATTCATATACGCTAGTGTCATAGTCCTGAAAATCATCTGCATCAAGAACAATTTCACAACTCTTA
TACTTTTCTCTTACAAGTCGTTCGGCTTCATCTGGATTTTCAGCCTCTATACTTACTAAACGTGATAAAGTTTCTGTAATTTCTACTGTATCGACCT
GCAGACTGGCTGTGTATAAGGGAGCCTGACATTTATATTCCCCAGAACATCAGGTTAATGGCGTTTTTGATGTCATTTTCGCGGTGGCTGAGATCAG
CCACTTCTTCCCCGATAACGGACACCGGCACACTGGCCATATCGGTGGTCATCATGCGCCAGCTTTCATCCCCGATATGCACCACCGGGTAAAGTTC
ACGGGAGACTTTATCTGACAGCAGACGTGCACTGGCCAGGGGGATCACCATCCGTCGCCCGGGCGTGTCAATAATATCACTCTGTACATCCACAAAC
AGACGATAACGGCTCTCTCTTTTATAGGTGTAAACCTTAAACTGCATTTCACCAGCCCCTGTTCTCGTCAGCAAAAGAGCCGTTCATTTCAATAAAC
CGGGCGACCTCAGCCATCCCTTCCTGATTTTCCGCTTTCCAGCGTTCGGCACGCAGACGACGGGCTTCATTCTGCATGGTTGTGCTTACCAGACCGG
AGATATTGACATCATATATGCCTTGAGCAACTGATAGCTGTCGCTGTCAACTGTCACTGTAATACGCTGCTTCATAGCATACCTCTTTTTGACATAC
TTCGGGTATACATATCAGTATATATTCTTATACCGCAAAAATCAGCGCGCAAATACGCATACTGTTATCTGGCTTTTAGTAAGCCGGATCCACGCGG
CGTTTACGCCCCCCCTGCCACTCATCGCAGTACTGTTGTAATTCATTAAGCATTCTGCCGACATGGAAGCCATCACAAACGGCATGATGAACCTGAA
TCGCCAGCGGCATCAGCACCTTGTCGCCTTGCGTATAATATTTGCCCATGGTGAAAACGGGGGCGAAGAAGTTGTCCATATTGGCCACGTTTAAATC
AAAACTGGTGAAACTCACCCAGGGATTGGCTGAGACGAAAAACATATTCTCAATAAACCCTTTAGGGAAATAGGCCAGGTTTTCACCGTAACACGCC
ACATCTTGCGAATATATGTGTAGAAACTGCCGGAAATCGTCGTGGTATTCACTCCAGAGCGATGAAAACGTTTCAGTTTGCTCATGGAAAACGGTGT
AACAAGGGTGAACACTATCCCATATCACCAGCTCACCGTCTTTCATTGCCATACGGAATTCCGGATGAGCATTCATCAGGCGGGCAAGAATGTGAAT
AAAGGCCGGATAAAACTTGTGCTTATTTTTCTTTACGGTCTTTAAAAAGGCCGTAATATCCAGCTGAACGGTCTGGTTATAGGTACATTGAGCAACT
GACTGAAATGCCTCAAAATGTTCTTTACGATGCCATTGGGATATATCAACGGTGGTATATCCAGTGATTTTTTTCTCCATTTTAGCTTCCTTAGCTC
CTGAAAATCTCGATAACTCAAAAAATACGCCCGGTAGTGATCTTATTTCATTATGGTGAAAGTTGGAACCTCTTACGTGCCGATCAACGTCTCATTT
TCGCCAAAAGTTGGCCCAGGGCTTCCCGGTATCAACAGGGACACCAGGATTTATTTATTCTGCGAAGTGATCTTCCGTCACAGGTATTTATTCGGCG
CAAAGTGCGTCGGGTGATGCTGCCAACTTAGTCGACATAATGAGACCGCTTCCTCGCTCACTGACTCGCTGCGCTCGGTCGTTCGGCTGCGGCGAGC
GGTATCAGCTCACTCAAAGGCGGTAATACGGTTATCCACAGAATCAGGGGATAACGCAGGAAAGAACATGTGAGCAAAAGGCCAGCAAAAGGCCAGG
AACCGTAAAAAGGCCGCGTTGCTGGCGTTTTTCCATAGGCTCCGCCCCCCTGACGAGCATCACAAAAATCGACGCTCAAGTCAGAGGTGGCGAAACC
CGACAGGACTATAAAGATACCAGGCGTTTCCCCCTGGAAGCTCCCTCGTGCGCTCTCCTGTTCCGACCCTGCCGCTTACCGGATACCTGTCCGCCTT
TCTCCCTTCGGGAAGCGTGGCGCTTTCTCATAGCTCACGCTGTAGGTATCTCAGTTCGGTGTAGGTCGTTCGCTCCAAGCTGGGCTGTGTGCACGAA
CCCCCCGTTCAGCCCGACCGCTGCGCCTTATCCGGTAACTATCGTCTTGAGTCCAACCCGGTAAGACACGACTTATCGCCACTGGCAGCAGCCACTG
GTAACAGGATTAGCAGAGCGAGGTATGTAGGCGGTGCTACAGAGTTCTTGAAGTGGTGGCCTAACTACGGCTACACTAGAAGAACAGTATTTGGTAT
CTGCGCTCTGCTGAAGCCAGTTACCTTCGGAAAAAGAGTTGGTAGCTCTTGATCCGGCAAACAAACCACCGCTGGTAGCGGTGGTTTTTTTGTTTGC
AAGCAGCAGATTACGCGCAGAAAAAAAGGATCTCAAGAAGATCCTTTGATCTTTTCTACGGGGTCTGACGCTCAGTGGAACGAAAACTCACGTTAAG
GGATTTTGGTCATGAGATTATCAAAAAGGATCTTCACCTAGATCCTTTTAAATTAAAAATGAAGTTTTAAATCAATCTAAAGTATATATGAGTAAAC
TTGGTCTGACAGTTACCAATGCTTAATCAGTGAGGCACCTATCTCAGCGATCTGTCTATTTCGTTCATCCATAGTTGCCTGACTCCCCGTCGTGTAG
ATAACTACGATACGGGAGGGCTTACCATCTGGCCCCAGTGCTGCAATGATACCGCGAGATCCACGCTCACCGGCTCCAGATTTATCAGCAATAAACC
AGCCAGCCGGAAGGGCCGAGCGCAGAAGTGGTCCTGCAACTTTATCCGCCTCCATCCAGTCTATTAATTGTTGCCGGGAAGCTAGAGTAAGTAGTTC
GCCAGTTAATAGTTTGCGCAACGTTGTTGCCATTGCTACAGGCATCGTGGTGTCACGCTCGTCGTTTGGTATGGCTTCATTCAGCTCCGGTTCCCAA
CGATCAAGGCGAGTTACATGATCCCCCATGTTGTGCAAAAAAGCGGTTAGCTCCTTCGGTCCTCCGATCGTTGTCAGAAGTAAGTTGGCCGCAGTGT
TATCACTCATGGTTATGGCAGCACTGCATAATTCTCTTACTGTCATGCCATCCGTAAGATGCTTTTCTGTGACTGGTGAGTACTCAACCAAGTCATT
CTGAGAATAGTGTATGCGGCGACCGAGTTGCTCTTGCCCGGCGTCAATACGGGATAATACCGCGCCACATAGCAGAACTTTAAAAGTGCTCATCATT
GGAAAACGTTCTTCGGGGCGAAAACTCTCAAGGATCTTACCGCTGTTGAGATCCAGTTCGATGTAACCCACTCGTGCACCCAACTGATCTTCAGCAT
CTTTTACTTTCACCAGCGTTTCTGGGTGAGCAAAAACAGGAAGGCAAAATGCCGCAAAAAAGGGAATAAGGGCGACACGGAAATGTTGAATACTCAT
ACTCTTCCTTTTTCAATATTATTGAAGCATTTATCAGGGTTATTGTCTCATGAGCGGATACATATTTGAATGTATTTAGAAAAATAAACAAATAGGG
GTTCCGCGCACATTTCCCCGAAAAGTGCCACCTGACGTCTAAGAAACCATTATTATCATGACATTAACCTATAAAAATAGGCGTATCACGAGGCCCT
TTCGTC

>pBD059
ATGGGCGGCGCATGAGAGAAGCCCAGACCAATTACCTACCCAAAATGGAGAAAGTTCACGTTGACATCGAGGAAGACAGCCCATTCCTCAGAGCTTT
GCAGCGGAGCTTCCCGCAGTTTGAGGTAGAAGCCAAGCAGGTCACTGATAATGACCATGCTAATGCCAGAGCGTTTTCGCATCTGGCTTCAAAACTG
ATCGAAACGGAGGTGGACCCATCCGACACGATCCTTGACATTGGAAGTGCGCCCGCCCGCAGAATGTATTCTAAGCACAAGTATCATTGTATCTGTC
CGATGAGATGTGCGGAAGATCCGGACAGATTGTATAAGTATGCAACTAAGCTGAAGAAAAACTGTAAGGAAATAACTGATAAGGAATTGGACAAGAA
AATGAAGGAGCTCGCCGCCGTCATGAGCGACCCTGACCTGGAAACTGAGACTATGTGCCTCCACGACGACGAGTCGTGTCGCTACGAAGGGCAAGTC
GCTGTTTACCAGGATGTATACGCGGTTGACGGACCGACAAGTCTCTATCACCAAGCCAATAAGGGAGTTAGAGTCGCCTACTGGATAGGCTTTGACA
CCACCCCTTTTATGTTTAAGAACTTGGCTGGAGCATATCCATCATACTCTACCAACTGGGCCGACGAAACCGTGTTAACGGCTCGTAACATAGGCCT
ATGCAGCTCTGACGTTATGGAGCGGTCACGTAGAGGGATGTCCATTCTTAGAAAGAAGTATTTGAAACCATCCAACAATGTTCTATTCTCTGTTGGC
TCGACCATCTACCACGAGAAGAGGGACTTACTGAGGAGCTGGCACCTGCCGTCTGTATTTCACTTACGTGGCAAGCAAAATTACACATGTCGGTGTG
AGACTATAGTTAGTTGCGACGGGTACGTCGTTAAAAGAATAGCTATCAGTCCAGGCCTGTATGGGAAGCCTTCAGGCTATGCTGCTACGATGCACCG
CGAGGGATTCTTGTGCTGCAAAGTGACAGACACATTGAACGGGGAGAGGGTCTCTTTTCCCGTGTGCACGTATGTGCCAGCTACATTGTGTGACCAA
ATGACTGGCATACTGGCAACAGATGTCAGTGCGGACGACGCGCAAAAACTGCTGGTTGGGCTCAACCAGCGTATAGTCGTCAACGGTCGCACCCAGA
GAAACACCAATACCATGAAAAATTACCTTTTGCCCGTAGTGGCCCAGGCATTTGCTAGGTGGGCAAAGGAATATAAGGAAGATCAAGAAGATGAAAG
GCCACTAGGACTACGAGATAGACAGTTAGTCATGGGGTGTTGTTGGGCTTTTAGAAGGCACAAGATAACATCTATTTATAAGCGCCCGGATACCCAA
ACCATCATCAAAGTGAACAGCGATTTCCACTCATTCGTGCTGCCCAGGATAGGCAGTAACACATTGGAGATCGGGCTGAGAACAAGAATCAGGAAAA
TGTTAGAGGAGCACAAGGAGCCGTCACCTCTCATTACCGCCGAGGACGTACAAGAAGCTAAGTGCGCAGCCGATGAGGCTAAGGAGGTGCGTGAAGC
CGAGGAGTTGCGCGCAGCTCTACCACCTTTGGCAGCTGATGTTGAGGAGCCCACTCTGGAAGCCGATGTCGACTTGATGTTACAAGAGGCTGGGGCC

GGCTCAGTGGAGACACCTCGTGGCTTGATAAAGGTTACCAGCTACGATGGCGAGGACAAGATCGGCTCTTACGCTGTGCTTTCTCCGCAGGCTGTAC
TCAAGAGTGAAAAATTATCTTGCATCCACCCTCTCGCTGAACAAGTCATAGTGATAACACACTCTGGCCGAAAAGGGCGTTATGCCGTGGAACCATA
CCATGGTAAAGTAGTGGTGCCAGAGGGACATGCAATACCCGTCCAGGACTTTCAAGCTCTGAGTGAAAGTGCCACCATTGTGTACAACGAACGTGAG
TTCGTAAACAGGTACCTGCACCATATTGCCACACATGGAGGAGCGCTGAACACTGATGAAGAATATTACAAAACTGTCAAGCCCAGCGAGCACGACG
GCGAATACCTGTACGACATCGACAGGAAACAGTGCGTCAAGAAAGAACTAGTCACTGGGCTAGGGCTCACAGGCGAGCTGGTGGATCCTCCCTTCCA
TGAATTCGCCTACGAGAGTCTGAGAACACGACCAGCCGCTCCTTACCAAGTACCAACCATAGGGGTGTATGGCGTGCCAGGATCAGGCAAGTCTGGC
ATCATTAAAAGCGCAGTCACCAAAAAAGATCTAGTGGTGAGCGCCAAGAAAGAAAACTGTGCAGAAATTATAAGGGACGTCAAGAAAATGAAAGGGC
TGGACGTCAATGCCAGAACTGTGGACTCAGTGCTCTTGAATGGATGCAAACACCCCGTAGAGACCCTGTATATTGACGAAGCTTTTGCTTGTCATGC
AGGTACTCTCAGAGCGCTCATAGCCATTATAAGACCTAAAAAGGCAGTGCTCTGCGGGGATCCCAAACAGTGCGGTTTTTTTAACATGATGTGCCTG
AAAGTGCATTTTAACCACGAGATTTGCACACAAGTCTTCCACAAAAGCATCTCTCGCCGTTGCACTAAATCTGTGACTTCGGTCGTCTCAACCTTGT
TTTACGACAAAAAAATGAGAACGACGAATCCGAAAGAGACTAAGATTGTGATTGACACTACCGGCAGTACCAAACCTAAGCAGGACGATCTCATTCT
CACTTGTTTCAGAGGGTGGGTGAAGCAGTTGCAAATAGATTACAAAGGCAACGAAATAATGACGGCAGCTGCCTCTCAAGGGCTGACCCGTAAAGGT
GTGTATGCCGTTCGGTACAAGGTGAATGAAAATCCTCTGTACGCACCCACCTCAGAACATGTGAACGTCCTACTGACCCGCACGGAGGACCGCATCG
TGTGGAAAACACTAGCCGGCGACCCATGGATAAAAACACTGACTGCCAAGTACCCTGGGAATTTCACTGCCACGATAGAGGAGTGGCAAGCAGAGCA
TGATGCCATCATGAGGCACATCTTGGAGAGACCGGACCCTACCGACGTCTTCCAGAATAAGGCAAACGTGTGTTGGGCCAAGGCTTTAGTGCCGGTG
CTGAAGACCGCTGGCATAGACATGACCACTGAACAATGGAACACTGTGGATTATTTTGAAACGGACAAAGCTCACTCAGCAGAGATAGTATTGAACC
AACTATGCGTGAGGTTCTTTGGACTCGATCTGGACTCCGGTCTATTTTCTGCACCCACTGTTCCGTTATCCATTAGGAATAATCACTGGGATAACTC
CCCGTCGCCTAACATGTACGGGCTGAATAAAGAAGTGGTCCGTCAGCTCTCTCGCAGGTACCCACAACTGCCTCGGGCAGTTGCCACTGGAAGAGTC
TATGACATGAACACTGGTACACTGCGCAATTATGATCCGCGCATAAACCTAGTACCTGTAAACAGAAGACTGCCTCATGCTTTAGTCCTCCACCATA
ATGAACACCCACAGAGTGACTTTTCTTCATTCGTCAGCAAATTGAAGGGCAGAACTGTCCTGGTGGTCGGGGAAAAGTTGTCCGTCCCAGGCAAAAT
GGTTGACTGGTTGTCAGACCGGCCTGAGGCTACCTTCAGAGCTCGGCTGGATTTAGGCATCCCAGGTGATGTGCCCAAATATGACATAATATTTGTT
AATGTGAGGACCCCATATAAATACCATCACTATCAGCAGTGTGAAGACCATGCCATTAAGCTTAGCATGTTGACCAAGAAAGCTTGTCTGCATCTGA
ATCCCGGCGGAACCTGTGTCAGCATAGGTTATGGTTACGCTGACAGGGCCAGCGAAAGCATCATTGGTGCTATAGCGCGGCTGTTCAAGTTTTCCCG
GGTATGCAAACCGAAATCCTCACTTGAAGAGACGGAAGTTCTGTTTGTATTCATTGGGTACGATCGCAAGGCCCGTACGCACAATCCTTACAAGCTT
TCATCAACCTTGACCAACATTTATACAGGTTCCAGACTCCACGAAGCCGGATGTGCACCCTCATATCATGTGGTGCGAGGGGATATTGCCACGGCCA
CCGAAGGAGTGATTATAAATGCTGCTAACAGCAAAGGACAACCTGGCGGAGGGGTGTGCGGAGCGCTGTATAAGAAATTCCCGGAAAGCTTCGATTT
ACAGCCGATCGAAGTAGGAAAAGCGCGACTGGTCAAAGGTGCAGCTAAACATATCATTCATGCCGTAGGACCAAACTTCAACAAAGTTTCGGAGGTT
GAAGGTGACAAACAGTTGGCAGAGGCTTATGAGTCCATCGCTAAGATTGTCAACGATAACAATTACAAGTCAGTAGCGATTCCACTGTTGTCCACCG
GCATCTTTTCCGGGAACAAAGATCGACTAACCCAATCATTGAACCATTTGCTGACAGCTTTAGACACCACTGATGCAGATGTAGCCATATACTGCAG
GGACAAGAAATGGGAAATGACTCTCAAGGAAGCAGTGGCTAGGAGAGAAGCAGTGGAGGAGATATGCATATCCGACGACTCTTCAGTGACAGAACCT
GATGCAGAGCTGGTGAGGGTGCATCCGAAGAGTTCTTTGGCTGGAAGGAAGGGCTACAGCACAAGCGATGGCAAAACTTTCTCATATTTGGAAGGGA
CCAAGTTTCACCAGGCGGCCAAGGATATAGCAGAAATTAATGCCATGTGGCCCGTTGCAACGGAGGCCAATGAGCAGGTATGCATGTATATCCTCGG
AGAAAGCATGAGCAGTATTAGGTCGAAATGCCCCGTCGAAGAGTCGGAAGCCTCCACACCACCTAGCACGCTGCCTTGCTTGTGCATCCATGCCATG
ACTCCAGAAAGAGTACAGCGCCTAAAAGCCTCACGTCCAGAACAAATTACTGTGTGCTCATCCTTTCCATTGCCGAAGTATAGAATCACTGGTGTGC
AGAAGATCCAATGCTCCCAGCCTATATTGTTCTCACCGAAAGTGCCTGCGTATATTCATCCAAGGAAGTATCTCGTGGAAACACCACCGGTAGACGA
GACTCCGGAGCCATCGGCAGAGAACCAATCCACAGAGGGGACACCTGAACAACCACCACTTATAACCGAGGATGAGACCAGGACTAGAACGCCTGAG
CCGATCATCATCGAAGAGGAAGAAGAGGATAGCATAAGTTTGCTGTCAGATGGCCCGACCCACCAGGTGCTGCAAGTCGAGGCAGACATTCACGGGC
CGCCCTCTGTATCTAGCTCATCCTGGTCCATTCCTCATGCATCCGACTTTGATGTGGACAGTTTATCCATACTTGACACCCTGGAGGGAGCTAGCGT
GACCAGCGGGGCAACGTCAGCCGAGACTAACTCTTACTTCGCAAAGAGTATGGAGTTTCTGGCGCGACCGGTGCCTGCGCCTCGAACAGTATTCAGG
AACCCTCCACATCCCGCTCCGCGCACAAGAACACCGTCACTTGCACCCAGCAGGGCCTGCTCGAGAACCAGCCTAGTTCCACCCCGCCAGGCGTGA
ATAGGGTGATCACTAGAGAGGAGCTCGAGGCGCTTACCCCGTCACGCACTCCTAGCAGGTCGGTCTCGAGAACCAGCCTGGTCTCCAACCCGCCAGG
CGTAAATAGGGTGATTACAAGAGAGGAGTTTGAGGCGTTCGTAGCACAACAACAATGACGGTTTGATGCGGGTGCATACATCTTTTCCTCCGACACC
GGTCAAGGGCATTTACAACAAAAATCAGTAAGGCAAACGGTGCTATCCGAAGTGGTGTTGGAGAGGACCGAATTGGAGATTTCGTATGCCCCGCGCC
TCGACCAAGAAAAAGAAGAATTACTACGCAAGAAATTACAGTTAAATCCCACACCTGCTAACAGAAGCAGATACCAGTCCAGGAAGGTGGAGAACAT
GAAAGCCATAACAGCTAGACGTATTCTGCAAGGCCTAGGGCATTATTTGAAGGCAGAAGGAAAAGTGGAGTGCTACCGAACCCTGCATCCTGTTCCT
TTGTATTCATCTAGTGTGAACCGTGCCTTTTCAAGCCCCAAGGTCGCAGTGGAAGCCTGTAACGCCATGTTGAAAGAGAACTTTCCGACTGTGGCTT
CTTACTGTATTATTCCAGAGTACGATGCCTATTTGGACATGGTTGACGGAGCTTCATGCTGCTTAGACACTGCCAGTTTTTGCCCTGCAAAGCTGCG
CAGCTTTCCAAAGAAACACTCCTATTTGGAACCCACAATACGATCGGCAGTGCCTTCAGCGATCCAGAACACGCTCCAGAACGTCCTGGCAGCTGCC
ACAAAAAGAAATTGCAATGTCACGCAAATGAGAGAATTGCCCGTATTGGATTCGGCGGCCTTTAATGTGGAATGCTTCAAGAAATATGCGTGTAATA
ATGAATATTGGGAAACGTTTAAAGAAAACCCCATCAGGCTTACTGAAGAAAACGTGGTAAATTACATTACCAAATTAAAAGGACCAAAAGCTGCTGC
TCTTTTTGCGAAGACACATAATTTGAATATGTTGCAGGACATACCAATGGACAGGTTTGTAATGGACTTAAAGAGAGACGTGAAAGTGACTCCAGGA
ACAAAACATACTGAAGAACGGCCCAAGGTACAGGTGATCCAGGCTGCCGATCCGCTAGCAACAGCGTATCTGTGCGGAATCCACCGAGAGCTGGTTA
GGAGATTAAATGCGGTCCTGCTTCCGAACATTCATACACTGTTTGATATGTCGGCTGAAGACTTTGACGCTATTATAGCCGAGCACTTCCAGCCTGG
GGATTGTGTTCTGGAAACTGACATCGCGTCGTTTGATAAAAGTGAGGACGACGCCATGGCTCTGACCGCGTTAATGATTCTGGAAGACTTAGGTGTG
GACGCAGAGCTGTTGACGCTGATTGAGGCGGCTTTCGGCGAAATTTCATCAATACATTTGCCCACTAAAACTAAATTTAAATTCGGAGCCATGATGA
AATCTGGAATGTTCCTCACACTGTTTGTGAACACAGTCATTAACATTGTAATCGCAAGCAGAGTGTTGAGAGAACGGCTAACCGGATCACCATGTGC
AGCATTCATTGGAGATGACAATATCGTGAAAGGAGTCAAATCGGACAAATTAATGGCAGACAGGTGCGCCACCTGGTTGAATATGGAAGTCAAGATT
ATAGATGCTGTGGTGGGCGAGAAAGCGCCTTATTTCTGTGGAGGGTTTATTTTGTGTGACTCCGTGACCGGCACAGCGTGCCGTGTGGCAGACCCCC
TAAAAAGGCTGTTTAAGCTTGGCAAACCTCTGGCAGCAGACGATGAACATGATGATGACAGGAGAAGGGCATTGCATGAAGAGTCAACACGCTGGAA
CCGAGTGGGTATTCTTTCAGAGCTGTGCAAGGCAGTAGAATCAAGGTATGAAACCGTAGGAACTTCCATCATAGTTATGGCCATGACTACTCTAGCT
AGCAGTGTTAAATCATTCAGCTACCTGAGAGGGGCCCCTATAACTCTCTACGGCTAACCTGAATGGACTACGACATAGTCTAGTCCGCCAAGGCCAC
CATGGAGGACGCTAAAAATATCAAGAAGGGTCCCGCTCCTTTCTATCCACTCGAGGACGGTACCGCTGGTGAGCAACTGCACAAGGCTATGAAACGG
TATGCTCTTGTGCCAGGCACCATAGCTTTCACCGACGCCCACATAGAGGTGGACATTACTTACGCAGAGTACTTTGAGATGTCTGTCCGGCTGGCAG
AGGCAATGAAAAGATATGGCCTCAACACAAACCATAGGATAGTGGTTTGCAGCGAGAATTCTCTTCAGTTTTTTATGCCCGTCCTCGGGGCTCTTTT
CATCGGCGTGGCTGTCGCCCCCGCCAACGACATATACAACGAGCGAGAGCTGCTGAACAGCATGGGAATCAGTCAGCCTACAGTGGTTTTTGTATCA
AAGAAGGGCTTGCAAAAGATCCTTAACGTACAGAAAAAGCTCCCTATTATTCAGAAGATCATTATAATGGATTCAAAGACCGACTATCAGGGATTCC
AGTCAATGTATACTTTCGTCACTTCCCACCTTCCTCCTGGGTTTAATGAATACGACTTCGTTCCCGAAAGTTTCGATCGGGACAAAACTATTGCACT
CATCATGAACTCCTCTGGGTCCACTGGGCTGCCTAAAGGTGTCGCACTTCCCCACAGGACTGCATGTGTAAGGTTCTCTCACGCACGAGATCCCATA
TTCGGTAACCAAATCATTCCTGATACCGCTATCCTCTCTGTAGTGCCTTTCCACCATGGATTCGGCATGTTCACCACTCTGGGTTACTTGATATGTG
GGTTCCGCGTGGTCCTTATGTACAGATTTGAAGAGGAGCTCTTTTTGAGGAGCTTGCAGGACTACAAAATCCAAAGTGCTCTCCTCGTCCCCACATT
GTTCTCATTCTTCGCCAAATCTACTCTCATCGACAAGTACGATTTGAGCAACTTGCACGAAATTGCTTCTGGGGGGGCTCCACTCTCCAAAGAAGTC
GGCGAAGCCGTTGCTAAAAGGTTCCACTTGCCAGGCATTCGCCAGGGGTATGGCCTTACTGAGACAACCTCAGCTATTCTCATTACCCCTGAGGGTG

ACGATAAGCCTGGTGCCGTCGGTAAGGTAGTACCATTTTTCGAGGCAAAGGTGGTCGACTTGGATACCGGAAAAACCCTCGGTGTGAATCAGAGAGG
CGAATTGTGTGTCAGGGGTCCAATGATCATGAGCGGTTACGTCAATAATCCCGAGGCCACTAACGCCTTGATTGATAAAGATGGTTGGCTTCATTCA
GGTGATATAGCATACTGGGATGAGGATGAACATTTCTTCATTGTCGATAGGCTCAAAAGCCTGATCAAGTATAAGGGTTACCAAGTGGCCCCTGCCG
AACTCGAATCTATACTGCTGCAACATCCAAACATTTTTGACGCTGGCGTGGCCGGACTGCCCGATGATGATGCTGGTGAACTCCCTGCCGCCGTTGT
CGTCCTGGAACATGGTAAGACCATGACAGAAAAGGAGATTGTAGATTACGTGGCTTCACAAGTGACTACAGCTAAGAAATTGCGCGGGGGTGTCGTT
TTCGTGGACGAGGTACCCAAGGGCCTCACTGGGAAGCTCGACGCCAGAAAAATCCGAGAAATCCTGATTAAAGCAAAGAAGGGCGGAAAGATTGCAG
TGTAATAAATACAGCAGCAATTGGCAAGCTGCTTACATAGAACTCGCGGCGATTGGCATGCCGCCTTAAAATTTTTATTTTATTTTTCTTTTCTTTT
CCGAATCGGATTTTGTTTTTAATATTTCAAAAAAAAAAAAAAAAAAAAAAAAAAAAAAAAAAAAAAAAAAATAGGGATAACAGGGTAATTGAGCAAAAGG
CCAGCAAAAGGCCAGGAACCGTAAAAAGGCCGCGTTGCTGGCGTTTTTCCATAGGCTCCGCCCCCCTGACGAGCATCACAAAAATCGACGCTCAAGT
CAGAGGTGGCGAAACCCGACAGGACTATAAAGATACCAGGCGTTTCCCCCTGGAAGCTCCCTCGTGCGCTCTCCTGTTCCGACCCTGCCGCTTACCG
GATACCTGTCCGCCTTTCTCCCTTCGGGAAGCGTGGCGCTTTCTCATAGCTCACGCTGTAGGTATCTCAGTTCGGTGTAGGTCGTTCGCTCCAAGCT
GGGCTGTGTGCACGAACCCCCCGTTCAGCCCGACCGCTGCGCCTTATCCGGTAACTATCGTCTTGAGTCCAACCCGGTAAGACACGACTTATCGCCA
CTGGCAGCAGCCACTGGTAACAGGATTAGCAGAGCGAGGTATGTAGGCGGTGCTACAGAGTTCTTGAAGTGGTGGCCTAACTACGGCTACACTAGAA
GAACAGTATTTGGTATCTGCGCTCTGCTGAAGCCAGTTACCTTCGGAAAAAGAGTTGGTAGCTCTTGATCCGGCAAACAAACCACCGCTGGTAGCGG
TGGTTTTTTTGTTTGCAAGCAGCAGATTACGCGCAGAAAAAAAGGATCTCAAGAAGATCCTTTGATCTTTTCTACGGGGTCTGACGCTCAGTGGAAC
GAAAACTCACGTTAAGGGATTTTGGTCATGAGATTATCAAAAAGGATCTTCACCTAGATCCTTTTAAATTAAAAATGAAGTTTTAAATCAATCTAAA
GTATATATGAGTAAACTTGGTCTGACAGTTACCAATGCTTAATCAGTGAGGCACCTATCTCAGCGATCTGTCTATTTCGTTCATCCATAGTTGCCTG
ACTCCCCGTCGTGTAGATAACTACGATACGGGAGGGCTTACCATCTGGCCCCAGTGCTGCAATGATACCGCGAGACCCACGCTCACCGGCTCCAGAT
TTATCAGCAATAAACCAGCCAGCCGGAAGGGCCGAGCGCAGAAGTGGTCCTGCAACTTTATCCGCCTCCATCCAGTCTATTAATTGTTGCCGGGAAG
CTAGAGTAAGTAGTTCGCCAGTTAATAGTTTGCGCAACGTTGTTGCCATTGCTACAGGCATCGTGGTGTCACGCTCGTCGTTTGGTATGGCTTCATT
CAGCTCCGGTTCCCAACGATCAAGGCGAGTTACATGATCCCCCATGTTGTGCAAAAAAGCGGTTAGCTCCTTCGGTCCTCCGATCGTTGTCAGAAGT
AAGTTGGCCGCAGTGTTATCACTCATGGTTATGGCAGCACTGCATAATTCTCTTACTGTCATGCCATCCGTAAGATGCTTTTCTGTGACTGGTGAGT
ACTCAACCAAGTCATTCTGAGAATAGTGTATGCGGCGACCGAGTTGCTCTTGCCCGGCGTCAATACGGGATAATACCGCGCCACATAGCAGAACTTT
AAAAGTGCTCATCATTGGAAAACGTTCTTCGGGGCGAAAACTCTCAAGGATCTTACCGCTGTTGAGATCCAGTTCGATGTAACCCACTCGTGCACCC
AACTGATCTTCAGCATCTTTTACTTTCACCAGCGTTTCTGGGTGAGCAAAAACAGGAAGGCAAAATGCCGCAAAAAAGGGAATAAGGGCGACACGGA
AATGTTGAATACTCATACTCTTCCTTTTTCAATATTATTGAAGCATTTATCAGGGTTATTGTCTCATGAGCGGATACATATTTGAATGTATTTAGAA
AAATAAACAAATAGGGGTTCCGCGCACATTTCCCCGAAAAGTGCCACCTGACGTTAGGGATAACAGGGTAATTAATACGACTCACTATAG

>pYL026
ATGGGCGGCGCATGAGAGAAGCCCAGACCAATTACCTACCCAAAATGGAGAAAGTTCACGTTGACATCGAGGAAGACAGCCCATTCCTCAGAGCTTT
GCAGCGGAGCTTCCCGCAGTTTGAGGTAGAAGCCAAGCAGGTCACTGATAATGACCATGCTAATGCCAGAGCGTTTTCGCATCTGGCTTCAAAACTG
ATCGAAACGGAGGTGGACCCATCCGACACGATCCTTGACATTGGAAGTGCGCCCGCCCGCAGAATGTATTCTAAGCACAAGTATCATTGTATCTGTC
CGATGAGATGTGCGGAAGATCCGGACAGATTGTATAAGTATGCAACTAAGCTGAAGAAAAACTGTAAGGAAATAACTGATAAGGAATTGGACAAGAA
AATGAAGGAGCTCGCCGCCGTCATGAGCGACCCTGACCTGGAAACTGAGACTATGTGCCTCCACGACGACGAGTCGTGTCGCTACGAAGGGCAAGTC
GCTGTTTACCAGGATGTATACGCGGTTGACGGACCGACAAGTCTCTATCACCAAGCCAATAAGGGAGTTAGAGTCGCCTACTGGATAGGCTTTGACA
CCACCCCTTTTATGTTTAAGAACTTGGCTGGAGCATATCCATCATACTCTACCAACTGGGCCGACGAAACCGTGTTAACGGCTCGTAACATAGGCCT
ATGCAGCTCTGACGTTATGGAGCGGTCACGTAGAGGGATGTCCATTCTTAGAAAGAAGTATTTGAAACCATCCAACAATGTTCTATTCTCTGTTGGC
TCGACCATCTACCACGAGAAGAGGGACTTACTGAGGAGCTGGCACCTGCCGTCTGTATTTCACTTACGTGGCAAGCAAAATTACACATGTCGGTGTG
AGACTATAGTTAGTTGCGACGGGTACGTCGTTAAAAGAATAGCTATCAGTCCAGGCCTGTATGGGAAGCCTTCAGGCTATGCTGCTACGATGCACCG
CGAGGGATTCTTGTGCTGCAAAGTGACAGACACATTGAACGGGGAGAGGGTCTCTTTTCCCGTGTGCACGTATGTGCCAGCTACATTGTGTGACCAA
ATGACTGGCATACTGGCAACAGATGTCAGTGCGGACGACGCGCAAAAACTGCTGGTTGGGCTCAACCAGCGTATAGTCGTCAACGGTCGCACCCAGA
GAAACACCAATACCATGAAAAATTACCTTTTGCCCGTAGTGGCCCAGGCATTTGCTAGGTGGGCAAAGGAATATAAGGAAGATCAAGAAGATGAAAG
GCCACTAGGACTACGAGATAGACAGTTAGTCATGGGGTGTTGTTGGGCTTTTAGAAGGCACAAGATAACATCTATTTATAAGCGCCCGGATACCCAA
ACCATCATCAAAGTGAACAGCGATTTCCACTCATTCGTGCTGCCCAGGATAGGCAGTAACACATTGGAGATCGGGCTGAGAACAAGAATCAGGAAAA
TGTTAGAGGAGCACAAGGAGCCGTCACCTCTCATTACCGCCGAGGACGTACAAGAAGCTAAGTGCGCAGCCGATGAGGCTAAGGAGGTGCGTGAAGC
CGAGGAGTTGCGCGCAGCTCTACCACCTTTGGCAGCTGATGTTGAGGAGCCCACTCTGGAAGCCGATGTCGACTTGATGTTACAAGAGGCTGGGGCC
GGCTCAGTGGAGACACCTCGTGGCTTGATAAAGGTTACCAGCTACGATGGCGAGGACAAGATCGGCTCTTACGCTGTGCTTTCTCCGCAGGCTGTAC
TCAAGAGTGAAAAATTATCTTGCATCCACCCTCTCGCTGAACAAGTCATAGTGATAACACACTCTGGCCGAAAAGGGCGTTATGCCGTGGAACCATA
CCATGGTAAAGTAGTGGTGCCAGAGGGACATGCAATACCCGTCCAGGACTTTCAAGCTCTGAGTGAAAGTGCCACCATTGTGTACAACGAACGTGAG
TTCGTAAACAGGTACCTGCACCATATTGCCACACATGGAGGAGCGCTGAACACTGATGAAGAATATTACAAAACTGTCAAGCCCAGCGAGCACGACG
GCGAATACCTGTACGACATCGACAGGAAACAGTGCGTCAAGAAAGAACTAGTCACTGGGCTAGGGCTCACAGGCGAGCTGGTGGATCCTCCCTTCCA
TGAATTCGCCTACGAGAGTCTGAGAACACGACCAGCCGCTCCTTACCAAGTACCAACCATAGGGGTGTATGGCGTGCCAGGATCAGGCAAGTCTGGC
ATCATTAAAAGCGCAGTCACCAAAAAAGATCTAGTGGTGAGCGCCAAGAAAGAAAACTGTGCAGAAATTATAAGGGACGTCAAGAAAATGAAAGGGC
TGGACGTCAATGCCAGAACTGTGGACTCAGTGCTCTTGAATGGATGCAAACACCCCGTAGAGACCCTGTATATTGACGAAGCTTTTGCTTGTCATGC
AGGTACTCTCAGAGCGCTCATAGCCATTATAAGACCTAAAAAGGCAGTGCTCTGCGGGGATCCCAAACAGTGCGGTTTTTTTAACATGATGTGCCTG
AAAGTGCATTTTAACCACGAGATTTGCACACAAGTCTTCCACAAAAGCATCTCTCGCCGTTGCACTAAATCTGTGACTTCGGTCGTCTCAACCTTGT
TTTACGACAAAAAAATGAGAACGACGAATCCGAAAGAGACTAAGATTGTGATTGACACTACCGGCAGTACCAAACCTAAGCAGGACGATCTCATTCT
CACTTGTTTCAGAGGGTGGGTGAAGCAGTTGCAAATAGATTACAAAGGCAACGAAATAATGACGGCAGCTGCCTCTCAAGGGCTGACCCGTAAAGGT
GTGTATGCCGTTCGGTACAAGGTGAATGAAAATCCTCTGTACGCACCCACCTCAGAACATGTGAACGTCCTACTGACCCGCACGGAGGACCGCATCG
TGTGGAAAACACTAGCCGGCGACCCATGGATAAAAACACTGACTGCCAAGTACCCTGGGAATTTCACTGCCACGATAGAGGAGTGGCAAGCAGAGCA
TGATGCCATCATGAGGCACATCTTGGAGAGACCGGACCCTACCGACGTCTTCCAGAATAAGGCAAACGTGTGTTGGGCCAAGGCTTTAGTGCCGGTG
CTGAAGACCGCTGGCATAGACATGACCACTGAACAATGGAACACTGTGGATTATTTTGAAACGGACAAAGCTCACTCAGCAGAGATAGTATTGAACC
AACTATGCGTGAGGTTCTTTGGACTCGATCTGGACTCCGGTCTATTTTCTGCACCCACTGTTCCGTTATCCATTAGGAATAATCACTGGGATAACTC
CCCGTCGCCTAACATGTACGGGCTGAATAAAGAAGTGGTCCGTCAGCTCTCTCGCAGGTACCCACAACTGCCTCGGGCAGTTGCCACTGGAAGAGTC
TATGACATGAACACTGGTACACTGCGCAATTATGATCCGCGCATAAACCTAGTACCTGTAAACAGAAGACTGCCTCATGCTTTAGTCCTCCACCATA
ATGAACACCCACAGAGTGACTTTTCTTCATTCGTCAGCAAATTGAAGGGCAGAACTGTCCTGGTGGTCGGGGAAAAGTTGTCCGTCCCAGGCAAAAT
GGTTGACTGGTTGTCAGACCGGCCTGAGGCTACCTTCAGAGCTCGGCTGGATTTAGGCATCCCAGGTGATGTGCCCAAATATGACATAATATTTGTT
AATGTGAGGACCCCATATAAATACCATCACTATCAGCAGTGTGAAGACCATGCCATTAAGCTTAGCATGTTGACCAAGAAAGCTTGTCTGCATCTGA
ATCCCGGCGGAACCTGTGTCAGCATAGGTTATGGTTACGCTGACAGGGCCAGCGAAAGCATCATTGGTGCTATAGCGCGGCTGTTCAAGTTTTCCCG
GGTATGCAAACCGAAATCCTCACTTGAAGAGACGGAAGTTCTGTTTGTATTCATTGGGTACGATCGCAAGGCCCGTACGCACAATCCTTACAAGCTT
TCATCAACCTTGACCAACATTTATACAGGTTCCAGACTCCACGAAGCCGGATGTGCACCCTCATATCATGTGGTGCGAGGGGATATTGCCACGGCCA
CCGAAGGAGTGATTATAAATGCTGCTAACAGCAAAGGACAACCTGGCGGAGGGGTGTGCGGAGCGCTGTATAAGAAATTCCCGGAAAGCTTCGATTT

ACAGCCGATCGAAGTAGGAAAAGCGCGACTGGTCAAAGGTGCAGCTAAACATATCATTCATGCCGTAGGACCAAACTTCAACAAAGTTTCGGAGGTT
GAAGGTGACAAACAGTTGGCAGAGGCTTATGAGTCCATCGCTAAGATTGTCAACGATAACAATTACAAGTCAGTAGCGATTCCACTGTTGTCCACCG
GCATCTTTTCCGGGAACAAAGATCGACTAACCCAATCATTGAACCATTTGCTGACAGCTTTAGACACCACTGATGCAGATGTAGCCATATACTGCAG
GGACAAGAAATGGGAAATGACTCTCAAGGAAGCAGTGGCTAGGAGAGAAGCAGTGGAGGAGATATGCATATCCGACGACTCTTCAGTGACAGAACCT
GATGCAGAGCTGGTGAGGGTGCATCCGAAGAGTTCTTTGGCTGGAAGGAAGGGCTACAGCACAAGCGATGGCAAAACTTTCTCATATTTGGAAGGGA
CCAAGTTTCACCAGGCGGCCAAGGATATAGCAGAAATTAATGCCATGTGGCCCGTTGCAACGGAGGCCAATGAGCAGGTATGCATGTATATCCTCGG
AGAAAGCATGAGCAGTATTAGGTCGAAATGCCCCGTCGAAGAGTCGGAAGCCTCCACACCACCTAGCACGCTGCCTTGCTTGTGCATCCATGCCATG
ACTCCAGAAAGAGTACAGCGCCTAAAAGCCTCACGTCCAGAACAAATTACTGTGTGCTCATCCTTTCCATTGCCGAAGTATAGAATCACTGGTGTGC
AGAAGATCCAATGCTCCCAGCCTATATTGTTCTCACCGAAAGTGCCTGCGTATATTCATCCAAGGAAGTATCTCGTGGAAACACCACCGGTAGACGA
GACTCCGGAGCCATCGGCAGAGAACCAATCCACAGAGGGGACACCTGAACAACCACCACTTATAACCGAGGATGAGACCAGGACTAGAACGCCTGAG
CCGATCATCATCGAAGAGGAAGAAGAGGATAGCATAAGTTTGCTGTCAGATGGCCCGACCCACCAGGTGCTGCAAGTCGAGGCAGACATTCACGGGC
CGCCCTCTGTATCTAGCTCATCCTGGTCCATTCCTCATGCATCCGACTTTGATGTGGACAGTTTATCCATACTTGACACCCTGGAGGGAGCTAGCGT
GACCAGCGGGGCAACGTCAGCCGAGACTAACTCTTACTTCGCAAAGAGTATGGAGTTTCTGGCGCGACCGGTGCCTGCGCCTCGAACAGTATTCAGG
AACCCTCCACATCCCGCTCCGCGCACAAGAACACCGTCACTTGCACCCAGCAGGGCCTGCTCGAGAACCAGCCTAGTTTCCACCCCGCCAGGCGTGA
ATAGGGTGATCACTAGAGAGGAGCTCGAGGCGCTTACCCCGTCACGCACTCCTAGCAGGTCGGTCTCGAGAACCAGCCTGGTCTCCAACCCGCCAGG
CGTAAATAGGGTGATTACAAGAGAGGAGTTTGAGGCGTTCGTAGCACAACAACAATGACGGTTTGATGCGGGTGCATACATCTTTTCCTCCGACACC
GGTCAAGGGCATTTACAACAAAAATCAGTAAGGCAAACGGTGCTATCCGAAGTGGTGTTGGAGAGGACCGAATTGGAGATTTCGTATGCCCCGCGCC
TCGACCAAGAAAAAGAAGAATTACTACGCAAGAAATTACAGTTAAATCCCACACCTGCTAACAGAAGCAGATACCAGTCCAGGAAGGTGGAGAACAT
GAAAGCCATAACAGCTAGACGTATTCTGCAAGGCCTAGGGCATTATTTGAAGGCAGAAGGAAAAGTGGAGTGCTACCGAACCCTGCATCCTGTTCCT
TTGTATTCATCTAGTGTGAACCGTGCCTTTTCAAGCCCCAAGGTCGCAGTGGAAGCCTGTAACGCCATGTTGAAAGAGAACTTTCCGACTGTGGCTT
CTTACTGTATTATTCCAGAGTACGATGCCTATTTGGACATGGTTGACGGAGCTTCATGCTGCTTAGACACTGCCAGTTTTTGCCCTGCAAAGCTGCG
CAGCTTTCCAAAGAAACACTCCTATTTGGAACCCACAATACGATCGGCAGTGCCTTCAGCGATCCAGAACACGCTCCAGAACGTCCTGGCAGCTGCC
ACAAAAAGAAATTGCAATGTCACGCAAATGAGAGAATTGCCCGTATTGGATTCGGCGGCCTTTAATGTGGAATGCTTCAAGAAATATGCGTGTAATA
ATGAATATTGGGAAACGTTTAAAGAAAACCCCATCAGGCTTACTGAAGAAAACGTGGTAAATTACATTACCAAATTAAAAGGACCAAAAGCTGCTGC
TCTTTTTGCGAAGACACATAATTTGAATATGTTGCAGGACATACCAATGGACAGGTTTGTAATGGACTTAAAGAGAGACGTGAAAGTGACTCCAGGA
ACAAAACATACTGAAGAACGGCCCAAGGTACAGGTGATCCAGGCTGCCGATCCGCTAGCAACAGCGTATCTGTGCGGAATCCACCGAGAGCTGGTTA
GGAGATTAAATGCGGTCCTGCTTCCGAACATTCATACACTGTTTGATATGTCGGCTGAAGACTTTGACGCTATTATAGCCGAGCACTTCCAGCCTGG
GGATTGTGTTCTGGAAACTGACATCGCGTCGTTTGATAAAAGTGAGGACGACGCCATGGCTCTGACCGCGTTAATGATTCTGGAAGACTTAGGTGTG
GACGCAGAGCTGTTGACGCTGATTGAGGCGGCTTTCGGCGAAATTTCATCAATACATTTGCCCACTAAAACTAAATTTAAATTCGGAGCCATGATGA
AATCTGGAATGTTCCTCACACTGTTTGTGAACACAGTCATTAACATTGTAATCGCAAGCAGAGTGTTGAGAGAACGGCTAACCGGATCACCATGTGC
AGCATTCATTGGAGATGACAATATCGTGAAAGGAGTCAAATCGGACAAATTAATGGCAGACAGGTGCGCCACCTGGTTGAATATGGAAGTCAAGATT
ATAGATGCTGTGGTGGGCGAGAAAGCGCCTTATTTCTGTGGAGGGTTTATTTTGTGTGACTCCGTGACCGGCACAGCGTGCCGTGTGGCAGACCCCC
TAAAAAGGCTGTTTAAGCTTGGCAAACCTCTGGCAGCAGACGATGAACATGATGATGACAGGAGAAGGGCATTGCATGAAGAGTCAACACGCTGGAA
CCGAGTGGGTATTCTTTCAGAGCTGTGCAAGGCAGTAGAATCAAGGTATGAAACCGTAGGAacttccatcatagttatggccatgactactctagct
agcagtgttaaatcattcagctacctgagaggggcccctataactctctacggctaacctgaatggactacgacatagtctagtccgccaaggcCAC
CATGTACAGCATGCAGCTCGCATCCTGTGTCACATTGACACTTGTGCTCCTTGTCAACAGCGCACCCACTTCAAGCTCCACTTCAAGCTCTACAGCG
GAAGCACAGCAGCAGCAGCAGCAGCAGCAGCAGCAGCAGCAGCACCTGGAGCAGCTGTTGATGGACCTACAGGAGCTCCTGAGCAGGATGGAGAATT
ACAGGAACCTGAAACTCCCCAGGATGCTCACCTTCAAATTTTACTTGCCCAAGCAGGCCACAGAATTGAAAGATCTTCAGTGCCTAGAAGATGAACT
TGGACCTCTGCGGCATGTTCTGGATTTGACTCAAAGCAAAAGCTTTCAATTGGAAGATGCTGAGAATTTCATCAGCAATATCAGAGTAACTGTTGTA
AAACTAAAGGGCTCTGACAACACATTTGAGTGCCAATTCGATGATGAGTCAGCAACTGTGGTGGACTTTCTGAGGAGATGGATAGCCTTCTGTCAAA
GCATCATCTCAACAAGCCCTCAAGGATCCGGCGCAACAAACTTCTCTCTGCTGAAACAAGCCGGAGATGTCGAAGAGAATCCTGGACCGATGGTGAG
CAAGGGCGAGGAGGATAACATGGCCATCATCAAGGAGTTCATGCGCTTCAAGGTGCACATGGAGGGCTCCGTGAACGGCCACGAGTTCGAGATCGAG
GGCGAGGGCGAGGGCCGCCCCTACGAGGGCACCCAGACCGCCAAGCTGAAGGTGACCAAGGGTGGCCCCCTGCCCTTCGCCTGGGACATCCTGTCCC
CTCAGTTCATGTACGGCTCCAAGGCCTACGTGAAGCACCCCGCCGACATCCCCGACTACTTGAAGCTGTCCTTCCCCGAGGGCTTCAAGTGGGAGCG
CGTGATGAACTTCGAGGACGGCGGCGTGGTGACCGTGACCCAGGACTCCTCCCTGCAGGACGGCGAGTTCATCTACAAGGTGAAGCTGCGCGGCACC
AACTTCCCCTCCGACGGCCCCGTAATGCAGAAGAAGACCATGGGCTGGGAGGCCTCCTCCGAGCGGATGTACCCCGAGGACGGCGCCCTGAAGGGCG
AGATCAAGCAGAGGCTGAAGCTGAAGGACGGCGGCCACTACGACGCTGAGGTCAAGACCACCTACAAGGCCAAGAAGCCCGTGCAGCTGCCCGGCGC
CTACAACGTCAACATCAAGTTGGACATCACCTCCCACAACGAGGACTACACCATCGTGGAACAGTACGAACGCGCCGAGGGCCGCCACTCCACCGGC
GGCATGGACGAGCTGTACAAGTAataaatacagcagcaattggcaagctgcttacatagaactcgcggcgattggcatgccgccttaaaatttttat
tttatttttcttttcttttccgaatcggattttgtttttaatatTTCAAAAAAAAAAAAAAAAAAAAAAAAAAAAAAAAAAAAAAAAAAAATAGGGATAAC
AGGGTAATTGAGCAAAAGGCCAGCAAAAGGCCAGGAACCGTAAAAAGGCCGCGTTGCTGGCGTTTTTCCATAGGCTCCGCCCCCCTGACGAGCATCA
CAAAAATCGACGCTCAAGTCAGAGGTGGCGAAACCCGACAGGACTATAAAGATACCAGGCGTTTCCCCCTGGAAGCTCCCTCGTGCGCTCTCCTGTT
CCGACCCTGCCGCTTACCGGATACCTGTCCGCCTTTCTCCCTTCGGGAAGCGTGGCGCTTTCTCATAGCTCACGCTGTAGGTATCTCAGTTCGGTGT
AGGTCGTTCGCTCCAAGCTGGGCTGTGTGCACGAACCCCCCGTTCAGCCCGACCGCTGCGCCTTATCCGGTAACTATCGTCTTGAGTCCAACCCGGT
AAGACACGACTTATCGCCACTGGCAGCAGCCACTGGTAACAGGATTAGCAGAGCGAGGTATGTAGGCGGTGCTACAGAGTTCTTGAAGTGGTGGCCT
AACTACGGCTACACTAGAAGAACAGTATTTGGTATCTGCGCTCTGCTGAAGCCAGTTACCTTCGGAAAAAGAGTTGGTAGCTCTTGATCCGGCAAAC
AAACCACCGCTGGTAGCGGTGGTTTTTTTGTTTGCAAGCAGCAGATTACGCGCAGAAAAAAAGGATCTCAAGAAGATCCTTTGATCTTTTCTACGGG
GTCTGACGCTCAGTGGAACGAAAACTCACGTTAAGGGATTTTGGTCATGAGATTATCAAAAAGGATCTTCACCTAGATCCTTTTAAATTAAAAATGA
AGTTTTAAATCAATCTAAAGTATATATGAGTAAACTTGGTCTGACAGTTACCAATGCTTAATCAGTGAGGCACCTATCTCAGCGATCTGTCTATTTC
GTTCATCCATAGTTGCCTGACTCCCCGTCGTGTAGATAACTACGATACGGGAGGGCTTACCATCTGGCCCCAGTGCTGCAATGATACCGCGAGACCC
ACGCTCACCGGCTCCAGATTTATCAGCAATAAACCAGCCAGCCGGAAGGGCCGAGCGCAGAAGTGGTCCTGCAACTTTATCCGCCTCCATCCAGTCT
ATTAATTGTTGCCGGGAAGCTAGAGTAAGTAGTTCGCCAGTTAATAGTTTGCGCAACGTTGTTGCCATTGCTACAGGCATCGTGGTGTCACGCTCGT
CGTTTGGTATGGCTTCATTCAGCTCCGGTTCCCAACGATCAAGGCGAGTTACATGATCCCCCATGTTGTGCAAAAAAGCGGTTAGCTCCTTCGGTCC
TCCGATCGTTGTCAGAAGTAAGTTGGCCGCAGTGTTATCACTCATGGTTATGGCAGCACTGCATAATTCTCTTACTGTCATGCCATCCGTAAGATGC
TTTTCTGTGACTGGTGAGTACTCAACCAAGTCATTCTGAGAATAGTGTATGCGGCGACCGAGTTGCTCTTGCCCGGCGTCAATACGGGATAATACCG
CGCCACATAGCAGAACTTTAAAAGTGCTCATCATTGGAAAACGTTCTTCGGGGCGAAAACTCTCAAGGATCTTACCGCTGTTGAGATCCAGTTCGAT
GTAACCCACTCGTGCACCCAACTGATCTTCAGCATCTTTTACTTTCACCAGCGTTTCTGGGTGAGCAAAAACAGGAAGGCAAAATGCCGCAAAAAAG
GGAATAAGGGCGACACGGAAATGTTGAATACTCATACTCTTCCTTTTTCAATATTATTGAAGCATTTATCAGGGTTATTGTCTCATGAGCGGATACA
TATTTGAATGTATTTAGAAAAATAAACAAATAGGGGTTCCGCGCACATTTCCCCGAAAAGTGCCACCTGACGTTAGGGATAACAGGGTAATTAATAC
GACTCACTATAG
